# Offsetting Low-Affinity Carbohydrate Binding with Covalency to Engage Sugar-Specific Proteins for Tumor-Immune Proximity Induction

**DOI:** 10.1021/acscentsci.3c01052

**Published:** 2023-11-03

**Authors:** Benjamin P. M. Lake, Anthony F. Rullo

**Affiliations:** ^†^Department of Medicine, McMaster Immunology Research Center, Center for Discovery in Cancer Research, ^‡^Department of Biochemistry and Biomedical Sciences, and ^§^Department of Chemistry and Chemical Biology, McMaster University, 1280 Main Street West, Hamilton Ontario, Canada

## Abstract

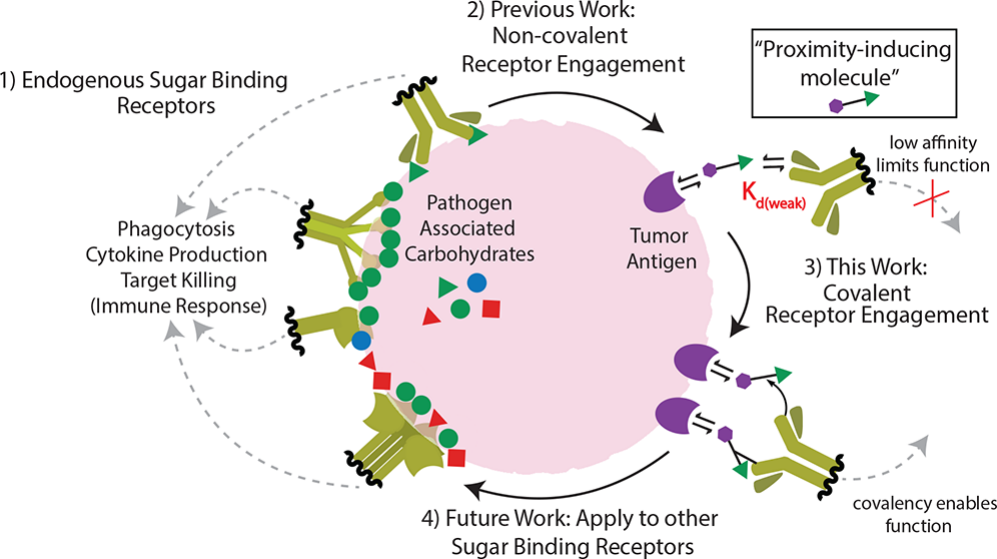

Carbohydrate-binding receptors are often used by the innate immune system to potentiate inflammation, target endocytosis/destruction, and adaptive immunity (e.g., CD206, DC-SIGN, MBL, and anticarbohydrate antibodies). To access this class of receptors for cancer immunotherapy, a growing repertoire of bifunctional proximity-inducing therapeutics use high-avidity multivalent carbohydrate binding domains to offset the intrinsically low affinity associated with monomeric carbohydrate–protein binding interactions (*K*_d_ ≈ 10^–3^–10^–6^ M). For applications aimed at recruiting anticarbohydrate antibodies to tumor cells, large synthetic scaffolds are used that contain both a tumor-binding domain (TBD) and a multivalent antibody-binding domain (ABD) comprising multiple l-rhamnose monosaccharides. This allows for stable bridging between tumor cells and antibodies, which activates tumoricidal immune function. Problematically, such multivalent macromolecules can face limitations including synthetic and/or structural complexity and the potential for off-target immune engagement. We envisioned that small bifunctional “proximity-inducing” molecules containing a low-affinity monovalent ABD could efficiently engage carbohydrate-binding receptors for tumor-immune proximity by coupling weak binding with covalent engagement. Typical covalent drugs and electrophilic chimeras use high-affinity ligands to promote the fast covalent engagement of target proteins (i.e., large *k*_inact_/*K*_I_), driven by a favorably small *K*_I_ for binding. We hypothesized the much less favorable *K*_I_ associated with carbohydrate–protein binding interactions can be offset by a favorably large *k*_inact_ for the covalent labeling step. In the current study, we test this hypothesis in the context of a model system that uses rhamnose-specific antibodies to induce tumor-immune proximity and tumoricidal function. We discovered that synthetic chimeric molecules capable of preorganizing an optimal electrophile (i.e., SuFEx vs activated ester) for protein engagement can rapidly covalently engage natural sources of antirhamnose antibody using only a single low-affinity rhamnose monosaccharide ABD. Strikingly, we observe chimeric molecules lacking an electrophile, which can only noncovalently bind the antibody, completely lack tumoricidal function. This is in stark contrast to previous work targeting small molecule hapten and peptide-specific antibodies. Our findings underscore the utility of covalency as a strategy to engage low-affinity carbohydrate-specific proteins for tumor-immune proximity induction.

## Abstract 2

We demonstate that heterobifunctional electrophilic molecules can covalently engage carbohydrate-specific proteins for tumor immunotherapy, leveraging only simple low-affinity sugars.

## Introduction

Carbohydrates play an essential role in many immunological functions through the specific engagement of receptors like lectins and anti-carbohydrate antibodies. For example, the mannose receptor (CD206) on macrophages recognizes mannose/fucose on pathogenic targets, leading to phagocytosis, and is a marker for macrophage polarization (CD206 overexpressed on M2-like tumor-associated macrophages).^1−4^ Dectin 1/2 receptors largely recognize β-glucans and mannose residues, respectively, to potentiate inflammatory antipathogenic responses.^5−12^ Furthermore, dendritic cell-specific ICAM-3 grabbing nonintegrin (DC-SIGN), largely expressed on dendritic cells, is associated with antigen uptake and subsequent MHC presentation for the potentiation of adaptive immune responses.^13−17^ Alternatively, soluble carbohydrate binding proteins such as mannose-binding lectin and anti-carbohydrate antibodies play critical roles in immune defenses against pathogens.^18−21^ For example, the PPV23 vaccine induces anticarbohydrate antibodies to protect against a variety of streptococcus pneumonia serotypes (e.g., induction of α-l-rhamnose specific antibodies recognizing the 23F serotype).^22−24^

Several bifunctional “proximity-inducing”^25,26^ therapeutic strategies function through the highly stabilized engagement of carbohydrate binding receptors. One strategy uses lysosome targeting chimeras (LYTACs), which integrate a tumor-targeting antibody with a multivalent array of GalNac and M6Pn sugars.^27,28^ The simultaneous engagement of ASGPR or CI-M6PR and the target leads to endocytosis and lysosome accumulation/degradation of the cell surface or soluble target proteins. Notably, analogous nonantibody-based approaches have also been reported.^29,30^

A second strategy uses monoclonal antibodies (mAbs) which function by simultaneously binding both tumor antigens (via their Fab domain) and sugar-specific immune receptors (via their glycosylated Fc domain).^31−35^ Upon reaching a critical threshold of antibodies localized to the target surface (opsonization), subsequent engagement of immune components (i.e. tumor-immune proximity induction) can affect target elimination via complement-dependent cytotoxicity (CDC), antibody-dependent cellular cytotoxicity (ADCC), or antibody-dependent cellular phagocytosis (ADCP). In fact, glyco-engineering the mAb glycan at position Asn297 can uniquely increase antitumor function by increasing antibody glycan binding affinity for Fc receptors.^36−41^ For example, defucosylation can stabilize glycan CD16α interactions, leading to enhanced ADCC.^42,43^

**Scheme 1:**
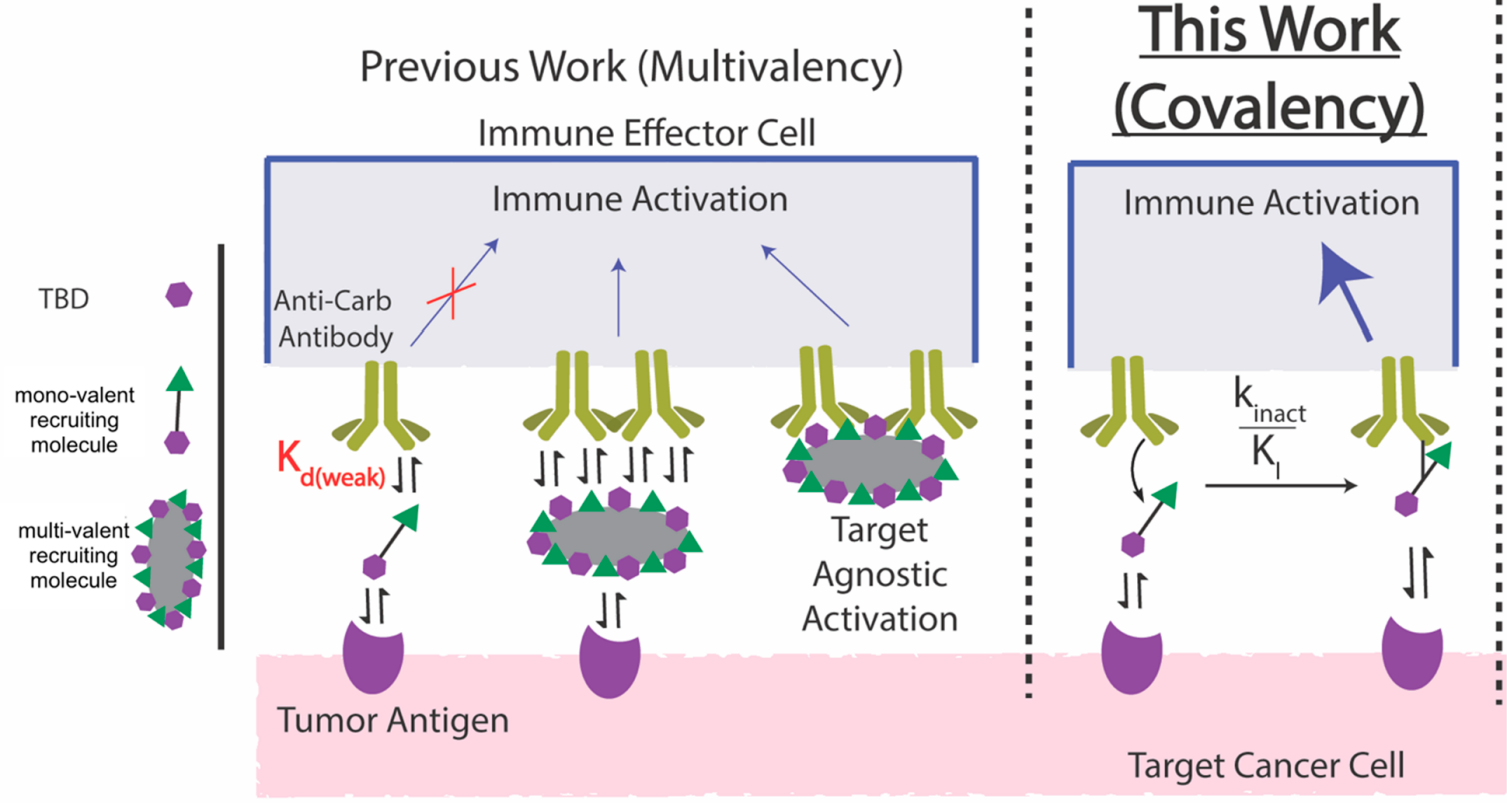
Established and Current/Emerging Chemical Strategies to Induce Tumor-Immune Proximity via Carbohydrate-Specific Antibodies Left panel: figure legend plus previous work using non-covalent monovalent and multivalent “chimera/recruiter/engager” approaches. Depicted is the limited capacity of the monovalent rhamnose chimera to induce tumor-immune proximity due to weak protein-carbohydrate binding affinity. This function is partially rescued via multivalency; however, this approach can compromise specificity due to target agnostic activation. Right panel: Current study leveraging covalency to enhance chimeric molecule engagement of carbohydrate-specific receptors (covalent linkage to antibody depicted by a thin black connecting line). Chimeric molecule covalently engaged with antirhamnose antibody can induce immune activation.

A third strategy uses multivalent macromolecular chimeric scaffolds to engage endogenous serum antirhamnose antibodies for tumor-immune proximity induction (Scheme 1). Here a synthetic scaffold is equipped with both a tumor binding domain (TBD) and a multivalent array of rhamnose sugars, serving as an antibody binding domain (ABD). For example, Coen et al. used a rhamnose-decorated polymer lipid anchored into cells, while Liet et al. demonstrated that dendrimeric rhamnose conjugates can bridge antirhamnose antibodies with target cells.^44,45^ Alternatively, Sheridan et al., Jakobsche et al., and Goyard et al. demonstrated antirhamnose-induced CDC of target cells through artificial multivalent rhamnose arrays on target cells (via lipid-anchored rhamnose, rhamnose-NHS-mediated cell surface labeling, or metabolic labeling to incorporate dendrimeric rhamnose, respectively).^46−48^ Interestingly, Li et al. used rhamnose- and folic acid-functionalized liposomes to induce targeted CDC in vitro and tumor regression in vivo.^49^

Intrinsically weak carbohydrate–protein binding affinity (*K*_d_ ≈ 10^–3^–10^–6^ M, typically defined by rapid *k*_off_) limits probe and therapeutic pursuits to target sugar binding receptors.^50−52^ The engagement strategies discussed above (e.g., LYTACs, antirhamnose recruiters) used multivalent carbohydrate modalities to enhance the apparent binding affinity for sugar-binding receptors. Such modalities leverage rapid microscopic rebinding *k*_on_ events to offset rapid microscopic dissociation *k*_off_ events. This reduces the overall dissociation of the bound therapeutic into the bulk solvent (increased *K*_dapp_). Multivalent immune-binding modalities, however, have been shown to cluster and activate immune proteins/receptors before localization to the target.^53−58^ In the context of tumor immunotherapy, this phenomenon would complicate therapeutic pharmacokinetics and potentially limit applications to direct tumor injection. Additionally, due to rapid microscopic dissociation events, these multivalent modalities may insufficiently stabilize immune cell–tumor cell proximity and synapse formation, to enforce anticancer immune function (e.g., ADCC and ADCP).^59,60^ Indeed, the effects of rapid microscopic dissociation and rebinding effects on the efficiency of immune receptor activation are currently not well-defined and may not be as effective as a slow microscopic dissociation event (i.e., small *k*_off_).^61^ Furthermore, the function of multivalent engagers has shown a subtle dependence on the spatial optimization of binding ligands, complicating design.^62−65^ Taken together, while conventional multivalent approaches to engage sugar-binding receptors can enhance *K*_dapp_, they are associated with the potential for off-target immune activation and complex design requirements, and may not optimally promote tumor-immune proximity induction.

As an alternative to multivalent engagement, we sought to develop a small bifunctional molecule approach that uses a minimal monovalent carbohydrate affinity ligand to engage sugar binding receptors with high apparent affinity. We hypothesized this could be achieved by coupling reversible carbohydrate binding with irreversible covalent engagement. This would kinetically stabilize monovalent carbohydrate–receptor interactions and potentially enhance therapeutic function (effectively infinitely increasing *K*_dapp_ through the elimination of *k*_off_). We previously developed covalent antibody recruiting molecules (cARMs) which can covalently engage endogenous small molecule and peptide-specific antibodies within a ternary complex.^66^ cARMs complement “chemically programmed” monoclonal antibodies and “reactive immunization” technologies by expanding access to endogenous protein receptors.^67,68^ Electrophilic chimeras such as cARMs and covalent drugs in general typically use high-affinity ligands to promote fast labeling kinetics with the target protein (i.e., large *k*_inact_/*K*_I_ due to small *K*_I_).^69,70^ This has traditionally limited access to engaging sugar binding receptors using these therapeutic modalities due to a weak (large) *K*_I_ for binding. We hypothesized that optimal preorganization of a key electrophile proximal to the carbohydrate, however, could offset an intrinsically less favorable *K*_I_ through significant increases in *k*_inact_.^70−72^

As a proof of concept, in the current study we set out to develop cARMs with the goal of engaging natural rhamnose-specific serum antibodies to promote tumor-immune proximity induction (monovalent recruiting molecule, Scheme 1). Antirhamnose antibody recruitment, provides an ideal model system for covalent engagement of sugar binding receptors using chimeric molecules for the following reasons: (1) the rhamnose binding affinity for antirhamnose is low and comparable to other sugar–protein receptor binding interactions, (2) human serum is a natural source of abundant antirhamnose antibodies, and (3) the precedence for successful tumor-immune proximity induction using multivalent scaffolds has been well established as highlighted above. In addition to serving as a general proof of concept, the development of cARMs targeting antirhamnose antibodies represents a potentially powerful addition to the current immune recruiting immunotherapeutic armamentarium.

cARMs were equipped with a monomeric rhamnose ABD to bind serum antirhamnose antibodies, an acyl imidazole electrophile, and an established glutamate urea (GU) TBD to target prostate-specific membrane antigen (PSMA, overexpressed 100–1000-fold in prostate cancer, GU ≈ *K*_d_ < 10^–8^ M).^59,73−77^ In this work, we observed that cARMs equipped with only a single monovalent rhamnose ligand can selectively covalently engage low-affinity rhamnose-specific serum antibodies. We also demonstrate that substitutions at the anomeric center (α-O vs α-S/β-S) are tolerated by these polyclonal antibodies. Additional kinetic studies with a commercial monoclonal antibody (anti-23F), derived from patients vaccinated with PPV23, show a uniquely distinct preference for the α-glycosidic configuration and reveal a potential for novel therapeutic synergy between antirhamnose recruitment chemical strategies and bacterial vaccinations.^24^ The discovery that a bacterial vaccine-induced antibody recognizes monovalent α-*O*-rhamnose cARMs provided a key model monoclonal antibody to optimize cARM covalent kinetics (*k*_inact_). We found that the weak *K*_I_ associated with binding rhamnose-specific antibodies could be offset by substituting the acyl imidazole electrophile on cARM, with an optimally pre-organized aryl sulfonyl fluoride electrophile. This substitution enabled dramatic increases in *k*_inact_ and subsequently the second-order labeling rate constant *k*_inact_/*K*_I._ Finally, in functional tumor-immune proximity induction assays, we observed that covalency is critically required for cARM dependent immune function, with analogous noncovalent ARMs completely lacking immunotherapeutic efficacy. This represents the most striking example of covalent enhancement in tumor-immune proximity induction in the literature. This work also contributes a rare example of an efficacious electrophilic bifunctional molecule/chimera that uses a weak affinity (μM) binding ligand for proximity induction. This discovery further increases the accessibility of sugar-binding immune receptors for synthetic immunotherapy using small chimeric molecules.

## Results and Discussion

### Probing Effects of Glycosidic Configuration on Recognition by Rhamnose-Specific Antibodies

The most efficient synthetic route for incorporating a rhamnose binding ABD into cARM molecules requires elaboration from the glycosidic linkage. This allows facile incorporation of both a covalent electrophile and TBD. Functionalization at the anomeric center, however, can significantly reduce/block the affinity for anti-rhamnose, which will decrease/inhibit covalent engagement kinetics (*k*_inact_/*K*_I_). This is particularly problematic since the monomeric rhamnose binding affinity for specific antibodies is substantially weaker than the typical range of ligand affinities (∼nM) commonly used for covalent drug development. For these reasons, we sought to test how structural perturbations to the glycosidic linkage (e.g., O vs S and α vs β glycoside) impact recognition by anti-rhamnose serum antibodies. This has yet to be systematically explored in the antibody recruiting literature.

**Figure 1:**
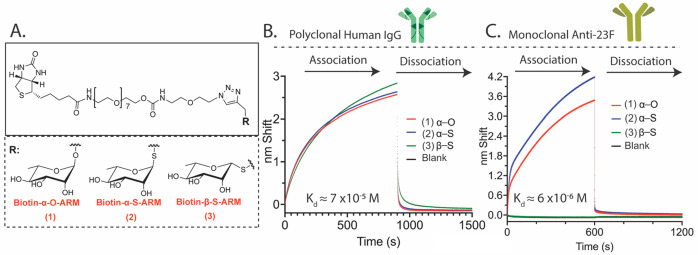
Affinity determination of endogenous and monoclonal antibody sources for l-rhamnose glycoside variants. A. Chemical structures of rhamnose affinity probes. B/C. Approximation of antirhamnose antibody binding affinity for probes **1**–**3** using biolayer interferometry. B. Purified human serum IgG is used to estimate the affinity of endogenous polyclonal serum antirhamnose antibodies for each affinity probe **1**–**3**. C. Monoclonal anti-23F antibody is used to estimate the potential affinity of vaccine-induced antirhamnose antibodies for each affinity probe **1**–**3**. Appropriate rhamnose affinity probes were preloaded onto streptavidin biosensors, followed by submersion in human IgG (26.25 μM, B) or anti-23F antibody (125 nM, C) solutions. Association was monitored for 900 (B) or 600 (C) seconds. The loaded biosensor probe was then dipped into a dissociation solution of 100 mM l-rhamnose for 600 s. Association curves were fit to a two-phase association model in GraphPad Prism using *K*_fast_ to calculate *k*_on_. Dissociation curves were similarly fit to a two-phase decay using *K*_fast_ to calculate *k*_off_.

We began by synthesizing antirhamnose affinity probes consisting of α-O/α-S and β-S rhamnose glycosides functionalized with a propargyl group for CuAAC-mediated coupling to a biotin TBD (compounds **1**–**3**, Figure 1A and Scheme S1). This enabled rhamnose immobilization onto streptavidin surfaces and subsequent antibody binding studies. We used biolayer interferometry (BLI) to estimate and compare the apparent affinity of serum polyclonal antirhamnose antibodies for each rhamnose affinity probe (Figure 1B). Each probe (**1**–**3**) was loaded onto streptavidin-coated biosensors and submerged in a solution of human IgG (26.25 μM) isolated from pooled human serum, followed by measurement of the association kinetics (Figure 1B, Association). Next, biosensors were submerged in a buffer containing free rhamnose competitor, and dissociation kinetics were measured (Figure 1B, Dissociation). The dissociation buffer contained 100 mM rhamnose competitor to inhibit avidity-based antibody rebinding, which interferes with *k*_off_ measurements. Each rhamnose glycosidic variant demonstrated comparable capacity to bind anti-rhamnose compared to blank probes (Blank probe, Figure 1B). Overall, each rhamnose glycosidic variant demonstrated comparably weak *K*_d_ values (∼70 μM, Table S1). In the context of proximity-inducing molecule development, this low monovalent sugar-binding affinity will ultimately limit ternary complex formation. A parallel ELISA study demonstrated similar results in the context of whole human serum (Figure S1). These results demonstrate that rhamnose-specific serum antibodies tolerate structural permutations about the rhamnose glycosidic linkage. The confirmation of low affinity binding also supports why multivalency has been historically required to efficiently engage antirhamnose antibodies for tumor-immune proximity induction and engage sugar binding receptors in general.

The advancement of synthetic immunotherapeutic modalities targeting anti-rhamnose antibodies has been complicated by a lack of model monoclonal antibody to enable medicinal chemistry optimization. Previous studies relied on polyclonal antirhamnose sources (e.g., human/rabbit serum) for validation studies. We hypothesized that antibodies induced by vaccination against the 23F serotype of streptococcus pneumonia (which contains α-rhamnose) would serve as a source of novel monoclonal antibodies. FDA-approved vaccines (such as PPV23) can induce anti-23F antibodies, with patient-derived monoclonal anti-23F antibody sequences commercially available.^22−24^ We proceeded to study anti-23F in BLI assays as done in Figure 1B (Figure 1C, association phase = 125 nM anti-23F). Here, we observed that anti-23F is uniquely specific for α-glycoside variants (compounds **1** and **2**). Notably, the calculated affinity of anti-23F antibody is ∼10× stronger than that for polyclonal serum antirhamnose, predominantly through increases in *k*_on_ (Tables S1 and S2). Collectively, our data support the use of anti-23F as a model monoclonal antirhamnose antibody and suggest a potential novel therapeutic synergy between PPV23 vaccination and antirhamnose recruitment chemical strategies (i.e., antirhamnose antibodies can be induced by vaccination prior to antirhamnose recruitment to disease targets via cARMs). Furthermore, given the efficient recognition of α-rhamnose by both endogenous human polyclonal and monoclonal antirhamnose sources, it was selected as the ABD for subsequent cARM development.

### Carbohydrate-Directed Covalent Engagement of Anti-rhamnose Antibodies–cARM Labeling Studies

**Figure 2:**
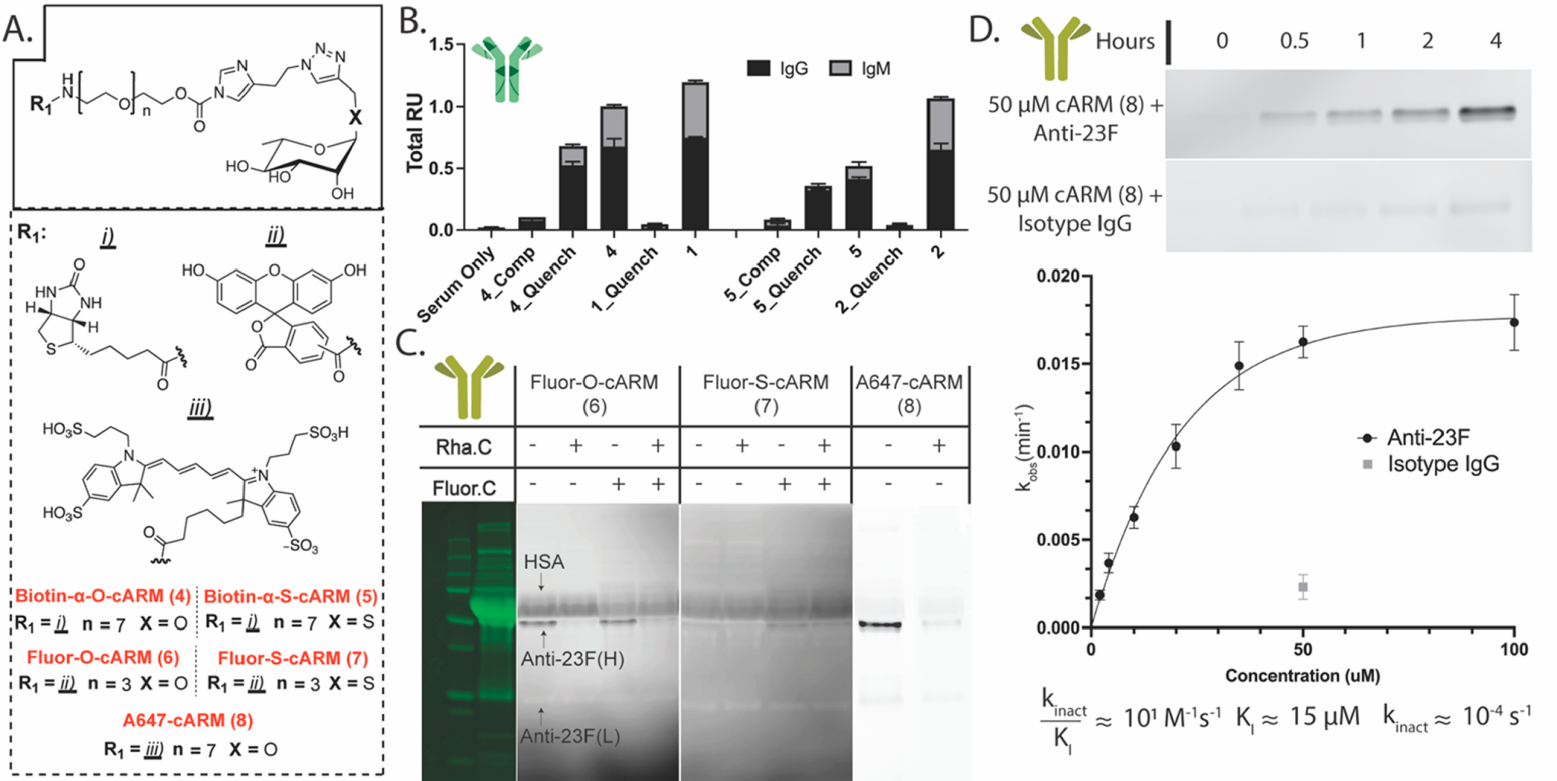
Capacity of Rha-cARMs to specifically covalently engage monoclonal anti-23F and serum anti-rhamnose. A. Chemical structures of the molecules investigated. B. Assessment of covalent vs noncovalent (cARM/ARM) engagement of human serum antirhamnose antibody via an ELISA assay. Each ARM/cARM (2 μM) was incubated with 2% human serum for 48 h and subsequently incubated directly on a streptavidin ELISA plate. Recruitment was evaluated by the addition of anti-IgG or anti-IgM HRP-conjugated secondary antibody with subsequent chromogenic substrate addition. “Comp” represents the addition of competitor rhamnose (500 mM) during 48 h of incubation. “Quench” refers to the addition of competitor rhamnose (100 mM) prior to ELISA plate binding but after 48 h of reaction incubation. C. SDS-PAGE fluorescent image of anti-23F antibody covalently labeled by fluorophore substituted Rha-cARMs (**6**–**8**). A representative protein stain associated with each gel is included on the left side of the fluorescent gel images. In all cases, 2 μM anti-23F antibody is incubated with 4 μM cARM (2 equiv) for 24 h in PBS spiked with 10% human serum. Where noted, l-rhamnose (100 mM, Rha. C) was used during incubation as a competitor for specific covalent labeling. Where noted, a free fluoresceine competitor (2.5 mM, Fluor. C) was used to distinguish fluorescein-promoted covalent labeling from rhamnose-promoted. The darker background in gels corresponding to cARM **6**, **7** is attributed to the differing capacity of free cARM-fluorophore (fluoroscein vs A647) to be retained in gel, as well as an alternative imaging channel (Cy2 for fluorescein, Cy5 for A647). Bands corresponding to labeled HSA and the anti-23F heavy chain are annotated. D. Kinetic SDS-PAGE fluorescence imaging experiment to determine Rha-AI-cARM/anti-23F antibody labeling kinetics using A647-cARM (**8**). cARM **8** (2–100 μM) was incubated with anti-23F antibody (2 μM) for up to 4 h. A representative fluorescence gel image is included corresponding to compound **8** (50 μM) incubated with either anti-23F antibody or isotype human IgG. Densitometry analysis with ImageJ was used to quantify relative band intensities. The calculation of kinetic constants is described in the Supporting Information. The error is calculated by alternative fits of initial rates data (all within the linear labeling range).

cARM design was guided by in-silico docking using anti-23F. Here a flexible PEG spacer tethered to the ABD was used to preorganize an appended acyl imidazole (AI) group, with K104 on the antibody heavy chain (Figure S2). An initial set of cARM tool compounds were synthesized to test if the cognate low-affinity monovalent rhamnose ABD can direct selective labeling of antirhamnose antibodies (compounds **4**–**8**, Figure 2A, Schemes S1 and S2). If successful, this would represent a rare example of leveraging weak binding sugar ligands to affinity label endogenous protein receptors.^85^ cARM chemical synthesis requires careful consideration of both the glycosidic linkage and acylimidazole activated ester reactivities. Given the lability of these functionalities to both pH and nucleophiles, we developed a convergent synthetic strategy that connects a preassembled electrophilic building block with an α-rhamnose glycoside fragment, via late-stage CuAAc coupling (Scheme S1).

Next, we tested if cARM tool compounds can selectively label endogenous human polyclonal antirhamnose antibodies via ELISA (**4** α-O rhamnose or **5** α-S rhamnose) (Figure 2B). cARMs (**4**, **5**) and noncovalent analog ARMs (**1**, **2**) were incubated with 2% human serum for 48 h followed by addition to a streptavidin-ELISA plate. Antibody recruitment to the plate surface was measured via the addition of HRP-conjugated anti-IgG or anti-IgM secondary antibody, followed by a chromogenic substrate. To assess labeling selectivity, the rhamnose competitor (Comp) was added during the incubation of cARM with antibody. The competitor will inhibit only ABD binding-dependent covalent labeling and antibody recruitment to the plate, but not nonspecific labeling. In the absence of a competitor, we observed robust absorbance signal in samples incubated with cARM. In the presence of a competitor, however, the signal was largely attenuated or abolished, consistent with selective covalent labeling. To discern covalent versus noncovalent antibody engagement, this experiment was repeated with rhamnose competitor added after the 48 h incubation to quench the selective labeling reaction, prior to addition to the ELISA plate. This quench control showed a baseline signal for each ARM compound (**1** and **2**) as anticipated since covalent engagement is not possible. The quench control for cARM compounds **4** and **5**, however, still exhibited significant antibody binding signal, supporting covalent engagement of anti-rhamnose. Comparing cARM compounds **4** and **5**, we observed that the α-O rhamnose cARM covalently engages more antibody compared to the α-S variant. Since the results in Figure 1 suggested serum antirhamnose recognizes these glycosidic variants similarly, this difference in labeling suggests differences in binding modes which are associated with different labeling efficiencies. In conclusion, these results support Rha-cARMs are capable of covalently engaging endogenous antirhamnose antibodies (both IgG and IgM) directly in human serum and stabilizing proximity with a target of interest.

To investigate cARM covalent engagement of higher-affinity monoclonal anti-23F, fluorescent cARMs were synthesized and evaluated using SDS-PAGE (Figure 2A/C, **6** fluorescein-α-O rhamnose, **7** fluorescein-α-S rhamnose, or **8** A647-α-O rhamnose). Each compound (4 μM) was incubated with anti-23F (2 μM) for 24 h directly in 10% human serum and analyzed via reducing SDS-PAGE. Covalently engaged proteins were visualized via fluorescent gel imaging. cARM compound **7** demonstrated significantly reduced capacity for labeling anti-23F as compared to compound **6**. This was largely attributed to the hydrolytic instability of the acylimidazole on **7** (Figure S4). In line with docking studies, cARM compound **6** exclusively labels the heavy chain of anti-23F. Consistent with the results in Figure 2B, the inclusion of a rhamnose competitor during the reaction (Rha.C) significantly reduces antibody labeling, demonstrating high selectivity. In these assays, we also observed the labeling of a single abundant off-target protein, HSA, which we hypothesized was promoted by fluorescein hydrophobic binding. To test this, we repeated the labeling reaction in the presence of fluorescein competitor (Fluor.C). In support of our hypothesis, HSA labeling by compound **6** was significantly reduced in the presence of fluorescein competitor. To further test this hypothesis, we repeated the labeling experiment with cARMs that substitute fluorescein with the more hydrophilic A647 fluorophore (compound **8**), connected with a longer peg linker. Compound **8** showed significantly reduced HSA labeling while retaining the ability to selectively label anti-23F antibody.

Next, we used SDS-PAGE to characterize anti-23F labeling kinetics. Compound **8** (2–100 μM) was incubated with anti-23F (2 μM) for up to 4 h. Densitometry analysis of fluorescently labeled protein bands was used to quantify antibody labeling (<10% product formation at *t* = 4 h for all cARM concentrations, Figure S5). Low product conversion allowed for an initial rates analysis to approximate both *k*_inact_ and *K*_I_, (Figure 2D, Figure S5, and Table S3). Labeling selectivity was demonstrated by incubating cARM with isotype IgG, which led to a significantly lower fluorescence band signal (even at high 50 μM cARM). Furthermore, labeling selectivity is supported by the observation of a plateau in a plot of *k*_obs_ vs concentration. This is inconsistent with a nonspecific bimolecular reaction that occurs independently of a pre-equilibrium binding step. The extraction of *K*_I_ from Figure 2D enabled an approximation of *K*_d_ (since *k*_off_ ≫ *k*_inact_) and is comparable to data obtained in BLI studies (Figure 1D). While the *k*_inact_/*K*_I_ describing cARM labeling is relatively small at ∼10^1^ M^–1^ s^–1^, our approach achieves specific measurable covalent antibody engagement. These slow kinetics, however, illustrate the difficulty associated with developing covalent chimeras targeting low-affinity sugar binding receptors.

### Proximity-Induced Antitumor Immune Function via Rhamnose-Specific Antibodies Requires Covalent Stabilization

**Figure 3:**
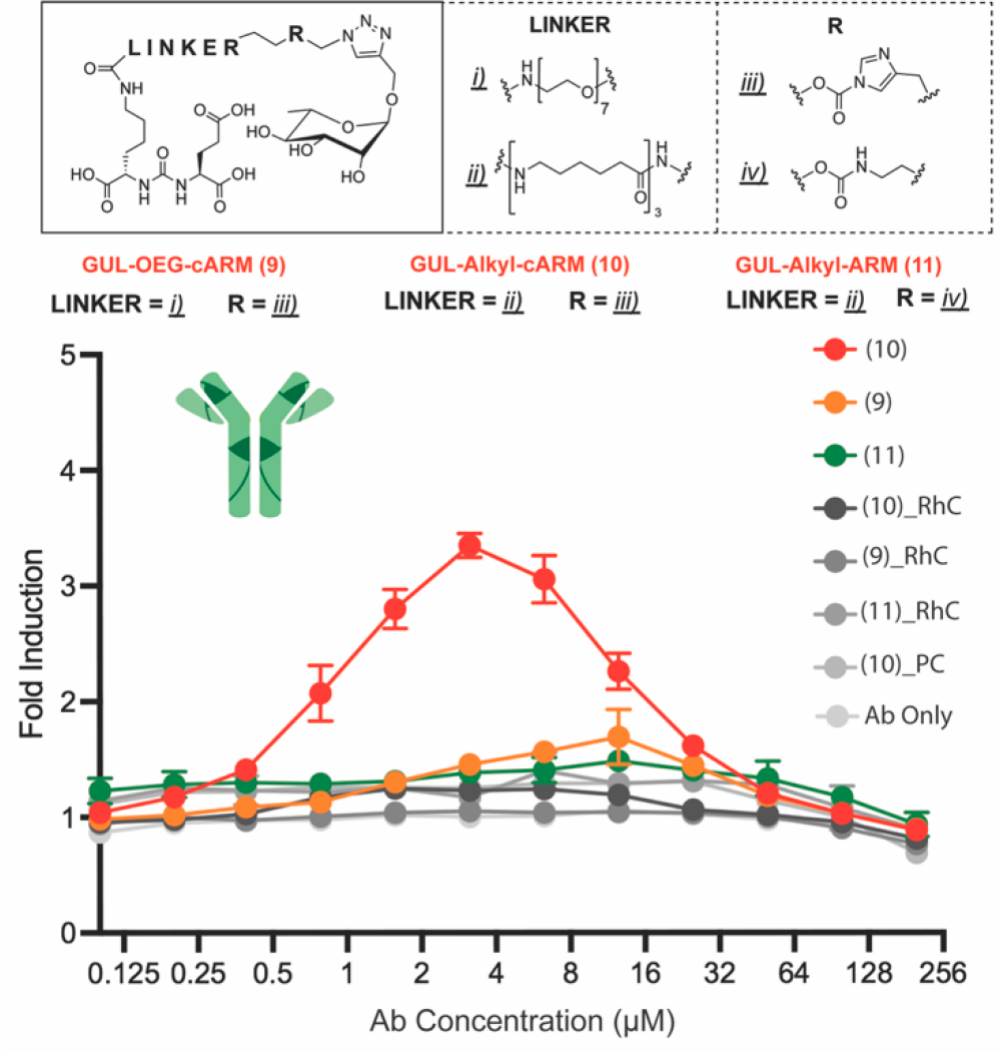
ADCC reporter assay to evaluate cARM vs ARM immune function using polyclonal anti-rhamnose antibodies. Top: chemical structures of compounds evaluated. Bottom: ADCC assay. Each cARM/ARM (50 μM) was incubated with polyclonal human IgG (230 μM) for 72 h and passed through a Millipore centrifugation column. Antibody solutions were then resuspended to experimental concentrations and added to preplated Hek-P cells (50,000 cells). To this cell solution, luciferase-expressing jurkat cells were added and incubated for 24 h. Activation of jurkat was determined as a measure of luciferase expression with a BioGlo assay kit.

Previous studies using high-affinity haptens or peptides to direct covalent antibody engagement revealed functional enhancements compared to noncovalent analogs. Given the high binding affinity interaction, however, noncovalent analogs also demonstrate varying degrees of function.^59,66,78^ Next, we investigated the potential ability of rhamnose cARMs and noncovalent ARM analogs to induce immune proximity with cancer targets. Using our convergent synthetic strategy outlined above, we synthesized PSMA targeting (GU containing) cARMs (compound **9**, flexible PEG linker; compound **10**, alkyl linker) and a comparable rhamnose-ARM (compound **11**, Schemes S3 and S4). These compounds were then evaluated for their capacity to activate immune cells against PSMA cancer targets (Hek-PSMA cells) in an ADCC reporter assay (Figure 3). Briefly, each compound (50 μM) was incubated with polyclonal human IgG (230 μM, ∼3× native serum concentration) for 72 h followed by a column washing step to model in vivo clearance (see Supporting Information). Antibody mixtures were then added directly to Hek-PSMA cells followed by the addition of CD16α expressing jurkat cells and incubation (24 h, 37 °C, 5% CO_2_). cARM **9** with a flexible PEG spacer and ARM **11** failed to induce immune activation. Interestingly, cARM **10** with an alkyl spacer successfully mediated specific immune activation. The inclusion of rhamnose competitor during the antibody labeling reaction (“RhC”) ablated signal, demonstrating the specificity of anti-rhamnose labeling vs abundant off-target antibodies in the sample. Use of a specific PSMA inhibitor (2-PMPA, “PC”) as well as isogenic PSMA(−) Hek cell line controls (Figure 3, Figure S6) showed no immune activation, demonstrating target specificity. Additional studies indicated that the loss of cARM **9** function compared to cARM **10** was due to an impaired ability to covalently label anti-23F (Figure S7). We hypothesize that the incorporation of the GU TBD and rhamnose ABD allows for intramolecular H-bonding between GU −COO^–^ and rhamnose −OH groups forming an inactive intramolecular complex unable to efficiently bind and label antibody. The formation of this complex may be prevented by the alkyl linker, which could adopt a fixed conformation via hydrophobic collapse. Taken together, these results show that both covalency and linker properties are critical to stabilizing ternary complexes with sugar binding receptors for proximity induction.

### Increasing *k*_inact_/*K*_I_ to Offset Weak Carbohydrate Binding Affinity: Incorporation of SuFEx Electrophiles into cARMs Enables Dramatic Enhancements in Covalent Labeling Kinetics

Rationally designed rhamnose-cARMs demonstrated specific covalent engagement of polyclonal and model monoclonal antirhamnose antibodies; however, the second-order labeling rate constant (*k*_inact_/*K*_I_) is relatively small to function efficiently directly in vivo. cARMs would thus require high concentrations and long incubation times for immune function. To function efficiently in vivo, antibody labeling kinetics must occur on a time scale of minutes to hours to compete with fast renal excretion. Strategies to enhance covalent chimera/inhibitor protein labeling kinetics typically include optimizing electrophile preorganization or enhancing the ligand binding affinity. For targeting sugar binding receptors using cARMs, however, these approaches are likely to require extensive medicinal chemistry to yield only modest improvements that are applicable to only a subset of polyclonal antirhamnose antibodies. Given the significant labeling rate enhancements observed in our laboratory using SuFEx chemistry combined with its versatile amino acid reactivity,^78−84^ we redesigned rhamnose cARMs to substitute the acyl imidazole for an aryl sulfonyl fluoride (SO_2_F) electrophile.

**Figure 4:**
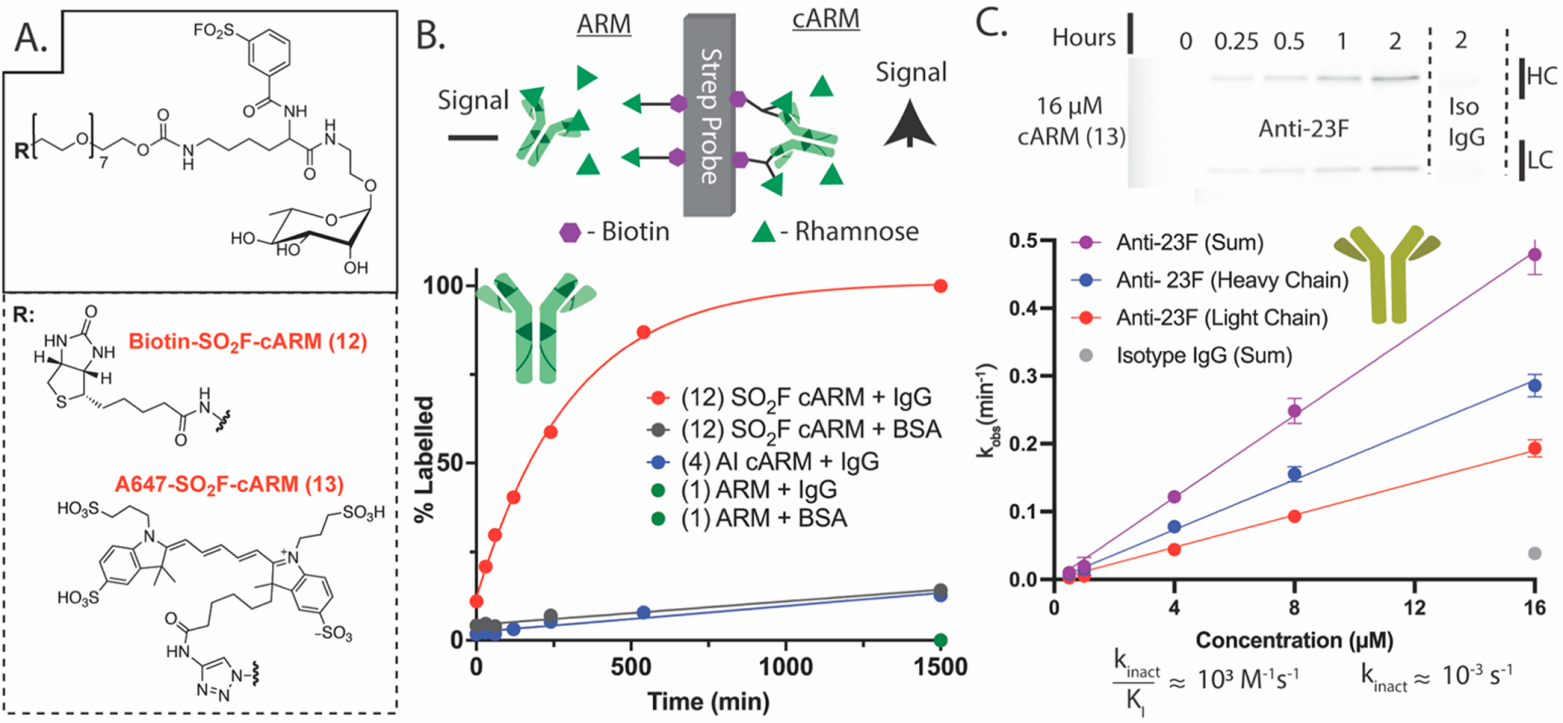
Selectivity and kinetic analysis of optimized Rha-SO_2_F-cARMs A. Chemical structures of tool compounds used in this study. B. cARM **12** (10 μM) was incubated with human IgG or BSA (230 μM) then diluted 8× with 100 mM competitor rhamnose spiked kinetics buffer and loaded onto streptavidin probes in BLI. The association plateau was plotted against time to yield the % labeled vs time covalent labeling curve C. Kinetic fluorescence SDS-PAGE assay similar to that in Figure 2D. Varying concentrations of cARM **13** were incubated with monoclonal anti-23F antibody (2 μM) for up to 2 h. Top: Representative gel demonstrating increased antibody labeling with time (cARM = 16 μM). Bottom: Plot of initial rates based on gel densitometry analysis as described in the Supporting Information.

The design of rhamnose-SO_2_F-cARM was again supported by in-silico docking and predicted to preorganize electrophilic SO_2_F proximal to lysine 104 and serine 53 of the anti-23F heavy chain as well as tyrosine 32 of the light chain (Figure S3). To evaluate the capacity of rhamnose-SO_2_F-cARMs to specifically label polyclonal antirhamnose antibodies, a tool cARM with a biotin TBD was synthesized (compound **12**, Figure 4A, Scheme S5). As an orthogonal kinetics assay to fluorescent gels, tool compound **12** labeling kinetics was first evaluated using an in-house BLI assay and compared to acyl imidazole cARM and ARM analogs. Here, each biotin containing cARM **12** or **4**, or comparable ARM **1** (10 μM), was incubated with human IgG (230 μM) for up to 25 h, followed by 8× dilution with rhamnose competitor (100 mM final rhamnose competitor). Solutions were immediately loaded onto streptavidin probes (Figure 4B, Figure S8). In this assay, rhamnose competition blocks noncovalent antirhamnose recruitment, thus the signal amplitude is directly proportional to the covalently labeled antibody product. The signal amplitude (labeled antibody) from BLI biosensor data (Figure S8) was plotted as a function of reaction time (Figure 4B), showing that novel cARM SO_2_F analog **12** reaches a plateau by 10 h while acyl imidazole containing cARM **4** is still in the linear phase of the reaction. Incubation of cARM **12** with BSA in lieu of IgG results in minimal loading/labeling, supporting high target protein selectivity.

As a reciprocal method to quantitatively compare rhamnose-SO_2_F-cARM vs rhamnose-AI-cARM antibody labeling kinetics, Alexa647-containing cARM compound **13** was synthesized and studied in fluorescent SDS-PAGE assays as described in Figure 2D (Figure 4C). Here, monoclonal anti-23F (2 μM) was incubated with compound **13** (0.5–16 μM) for up to 2 h and run directly on reducing SDS-PAGE. Initial rates kinetic analyses were performed as in Figure 2D and described in the Supporting Information (Figure S9). Once again, minimal isotype IgG labeling was observed, supporting high target antibody labeling selectivity. Consistent with in-silico studies and the capacity of SO_2_F to label multiple amino acids, compound **13** labeled both heavy and light chains of anti-23F. Extraction of *k*_inact_/*K*_I_ using initial rates (Figure 4C, anti-23F (sum)) combined with equation S1 enabled calculation of *k*_inact_ ≈ 10^–3^ s^–1^ (∼50× faster than comparable AI cARMs, Tables S3 and S4). The significantly improved labeling rate suggests that SO_2_F cARMs can also function more effectively in targeted immune activation assays and potentially in vivo. This significant increase in *k*_inact_ for SuFEx versus AI is striking, given that both electrophiles are comparably preorganized for reaction with the antibody, and suggests that features of the antibody binding pocket uniquely enhance SuFEx reactivity compared to that of other electrophiles used for affinity labeling applications such as acylimidazoles. This increase in *k*_inact_ also serves to uniquely offset the weak *K*_I_ for monovalent carbohydrate–protein receptor binding, i.e., antirhamnose, to afford rapid second-order reaction kinetics.

To further confirm selectivity, an MS study was conducted to determine the site of labeling (Figures S10 and S11). Rhamnose-SO_2_F-cARM was found to specifically label lysine 104 of the heavy chain, in line with in-silico studies. No other labeling site was detected on the light chain; however, the lability of the SO_2_F-serine/tyrosine linkage to MS sample preparation protocols likely limited complete labeling site coverage. Based on dual heavy/light chain labeling observed in SDS-PAGE (Figure 4C) and suggested by in-silico studies (Figure S3), it is highly likely that rhamnose-SO_2_F-cARM labels other binding pocket proximal residues, which is ideal for engaging a structurally heterogenic protein target such as serum antirhamnose antibodies. Due to the low labeling efficiency of rhamnose-AI-cARM, no labeling site could be identified. Collectively, these results demonstrate the kinetic advantage of using SO_2_F vs acylimidazole electrophiles for covalently stabilizing carbohydrate–protein interactions using bifunctional molecules. These results also support high selectivity for the covalent engagement of rhamnose specific antibodies versus off-target antibodies and serum proteins. Determining the selectivity for antirhamnose antibodies in the presence of off-target cell surface proteins will be the focus of future in vivo studies.

### SuFEx cARMs Outperform Acyl Imidazole cARMs in Functional Immune Assays

**Figure 5:**
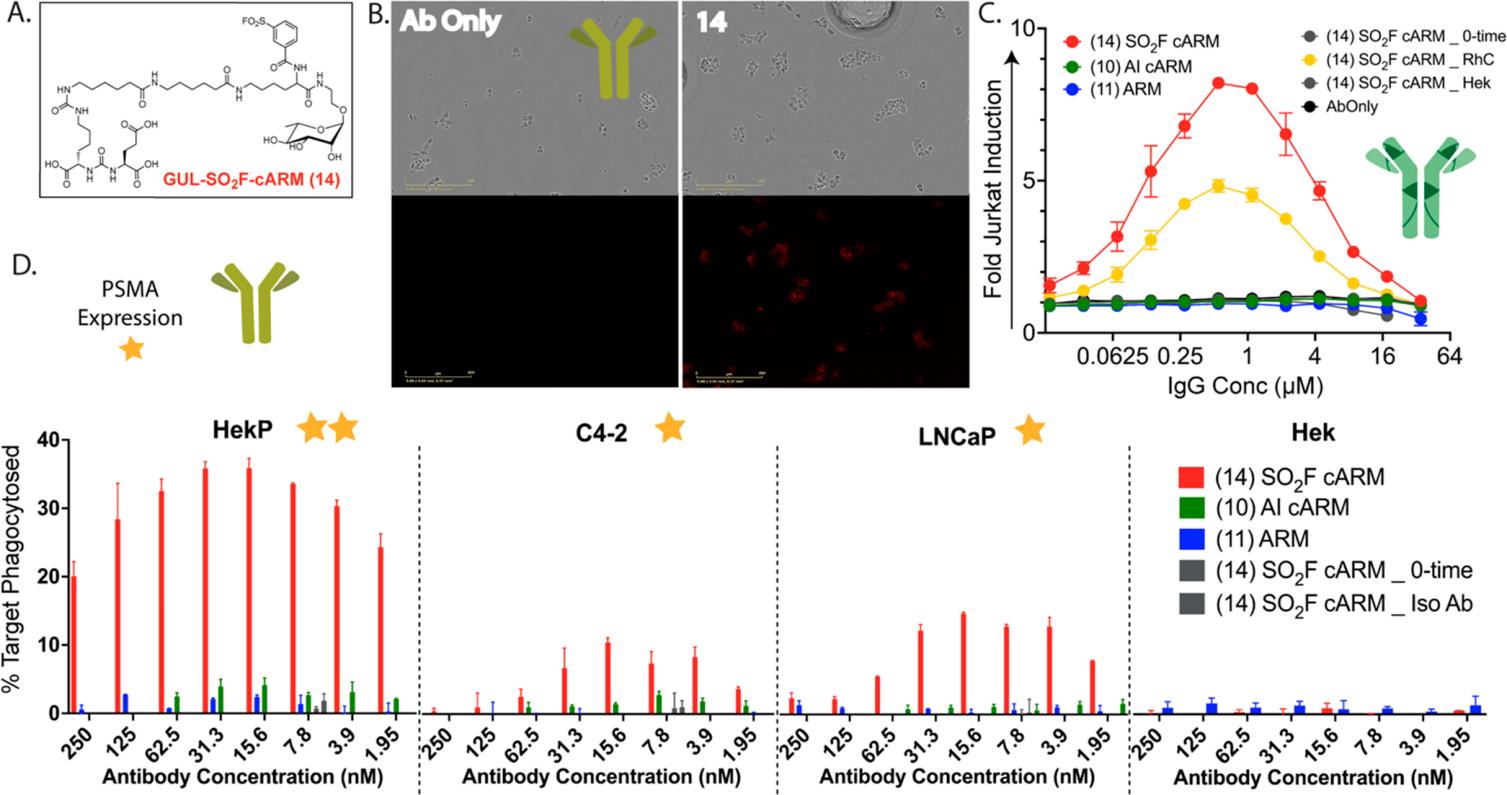
Functional analysis of PSMA-targeting SuFEx cARMs. A. Chemical structure of the functional GU cARM molecule used for PSMA targeting. B. Confocal (top) and fluorescent imaging (bottom) of Hek-PSMA cells treated with anti-23F only or anti-23F antibody pre-incubated with rhamnose-SO_2_F-cARM **14**. C. Jurkat activation assay evaluation of **14** directly in polyclonal human IgG. Each cARM/ARM (9 μM) was incubated with polyclonal human IgG (230 μM) overnight and then added to pre-plated Hek-P cells (50,000 cells). To this cell solution, luciferase expressing jurkat cells were added and incubated for 24 hours. Activation of jurkat cells was determined by measuring luciferase expression with a BioGlo assay kit. D. ADCP assays using various PSMA-expressing model and bonafide cancer cell lines. Each cARM/ARM (8 μM) was incubated with anti-23F (4 μM) overnight followed by addition to target cells (150,000 cells). Next, IFN-γ activated monocytes (150,000 cells) were added and incubated for 1 hour at 37 °C. ADCP was determined via flow cytometry.

To test the antitumor immune function of rhamnose SO_2_F cARMs, GU was again incorporated as a TBD for PSMA targeting (compound **14**, Figure 5A, Scheme S5). The ability to selectively induce opsonization of model prostate cancer cells via monoclonal anti-23F was first evaluated using fluorescence microscopy (Figure 5B, Figure S12). Compound **14** (8 μM) was incubated with anti-23F (4 μM) for 24 h followed by addition to PSMA expressing Hek-PSMA cells. Following a 20 min incubation, fluorescent secondary antibody was added. After an additional 20 min incubation, cells were fixed and imaged using an incucyte imager. cARM compound **14** was observed to induce target opsonization in contrast to antibody alone through specific PSMA-dependent targeting (Figure 5B, Figure S12). The addition of rhamnose competitor during the antibody labeling reaction again inhibited covalent antibody engagement and opsonization of target cells (Figure S12). Interestingly, comparable ARM **11**, which allows for noncovalent engagement of antirhamnose antibody, failed to opsonize target cells despite high affinity binding to PSMA on Hek-PSMA cells and high PSMA expression (∼1 × 10^6^ PSMA per cell, Figure S12).^54^ This is likely due to low affinity binding interactions between rhamnose and antirhamnose antibody (*K*_d_ ≈ 15 μM) which limits ternary complex formation. This result illustrates the power of covalency for the proximity induction of carbohydrate binding receptors using a low-affinity sugar ligand.

Next, the capacity of alkyl-AI-cARM **10**, alkyl-ARM **11**, and SO_2_F-cARM **14** to direct immune activation via polyclonal anti-rhamnose antibodies was evaluated using a modified ADCC reporter assay (Figure 5C). ADCC represents a major mode of antitumor NK cell immunotherapeutic function, common to many FDA-approved monoclonal antibody therapies. In these assays, each compound (9 μM) was incubated with human IgG (230 μM) for 24 h followed by direct addition to PSMA expressing Hek-PSMA cells and the addition of jurkat effector cells. Here, significant jurkat activation is observed using cARM **14** with milder labeling conditions as compared to those required for cARM **10** function (∼5× lower cARM concentration with an ∼3× shorter incubation and no column step, compared to Figure 3). Even in the absence of a column step (used to model in vivo clearance of the compound), noncovalent ARM **11** and AI-ARM **10** failed to direct immune activation. Inclusion of rhamnose competition (RhC) during incubation significantly attenuated immune activation; however, some antibody labeling and jurkat activation was observed. Since labeling selectivity is well established in reciprocal assays described above, we hypothesize that this is due to the rapid labeling kinetics of **14** and weak binding affinity (rapid *k*_off_) of the rhamnose competitor (which attenuates its competitive efficacy). Addition of cARM **14** directly to antibody and the target without prior incubation (0-time, before covalent engagement is established) leads to a baseline signal. This demonstrates the necessity of covalent engagement for functional immune activation. Repeating the experiment with isogenic PSMA(−) Hek cell lines failed to induce immune activation, demonstrating target specificity. A similar experiment using monoclonal anti-23F in lieu of polyclonal antibody directed analogous immune activation (Figure S13).

Finally, the capacity of Rha-cARMs to induce ADCP of multiple target cell lines (varying in PSMA expression) was evaluated. ADCP represents a major mode of antitumor monocyte/macrophage immunotherapeutic function, also invoked by several FDA-approved monoclonal antibody therapies. In these assays, each compound was incubated with monoclonal anti-23F for 24 h followed by addition to target cells (stained with DiO cell dye) and IFNγ activated U937 monocytes (stained with DiD cell dye). These mixtures were incubated (1 h, 37 °C), and ADCP was measured via flow cytometry. cARM compound **14** induced phagocytosis of all PSMA-expressing target cancer cells in a PSMA-dependent manner (Hek-PSMA ≈ 1 × 10^6^ PSMA per cell, LNCaP/C4-2∼2 × 10^5^ PSMA per cell, Hek is PSMA(−)).^54,74^ Failure of compound **14** to induce ADCP with isotype antibody supports high covalent labeling selectivity. Consistent with previous data, AI-cARM **10** is nonfunctional even against very high target expressing Hek-PSMA cells with affinity matured anti-23F antibody. The failure of ARM **11** as well as **14** at time zero (no covalent linkage formed) to induce ADCP demonstrates functional dependence on covalent antibody engagement. These results also demonstrate the inability of monovalent ARMs to induce efficacious ADCP/ADCC with low-affinity monovalent carbohydrate ligands. Additionally, we show that optimized SuFEx cARMs can covalently stabilize these low-affinity antirhamnose antibody–sugar binding interactions more efficiently than previous cARMs that used activated ester electrophiles to further enhance tumor-immune proximity induction.

## Conclusions

We report a proof of concept covalent proximity induction strategy for carbohydrate-specific protein engagement, with applications in glyco-immunotherapeutic development. Structure–activity relationship analysis surrounding the rhamnose glycosidic linkage, combined with the discovery of a model monoclonal anti-rhamnose antibody (anti-23F), contributes new knowledge and toolsets to aid the future development of antibody recruitment technology. We also reveal a potential therapeutic synergy between antirhamnose recruitment and FDA-approved antibacterial vaccines. Substituting activated ester electrophiles with SuFEx enabled striking increases in *k*_inact_ to afford the first covalent chimera that efficiently engages a sugar-specific protein and overcomes the obstacle posed by intrinsically weak carbohydrate binding affinity (i.e., large *K*_I_). cARM covalent chimeras also uniquely stabilize carbohydrate–protein interactions to rescue the loss of tumoricidal function associated with noncovalent ARM analogs. Our covalent glyco-chimeric strategy also presents a potential alternative to multivalency to engage sugar-specific receptors for proxmity induction using simple available monovalent carbohydrate ligands.

## Supporting Information Available

Additional material and method details, synthetic characterization (NMR spectra and mass spectrometry), ELISA, SDS-PAGE, BLI, microscopy, ADCC reporter assay, and flow cytometry data characterization (PDF)
