# Supplementary material for: Offsetting Low-Affinity Carbohydrate Binding with Covalency to Engage Sugar-Specific Proteins for Tumor-Immune Proximity Induction
Source: ACS Cent Sci. 2023 Nov 3;9(11):2064–75. doi: 10.1021/acscentsci.3c01052 (PMC10683482; doi:10.1021/acscentsci.3c01052)
Supplement: Supplementary file 1 — oc3c01052_si_001.pdf [file oc3c01052_si_001.pdf]

Title: Off-setting Low Affinity Carbohydrate Binding with Covalency, To Engage Sugar Specific Proteins For Tumor-Immune Proximity Induction

Authors: Benjamin P.M. Lake[⊥§], Anthony F. Rullo[∝⊥§*]

Affiliations: [⊥]Department of Medicine, McMaster Immunology Research Center, Center for Discovery in Cancer Research, [∝]Department of Biochemistry and Biomedical Sciences, [§]Department of Chemistry and Chemical Biology. McMaster University, 1280 Main street west, Hamilton Ontario, Canada.

*Corresponding author: Anthony F. Rullo, rulloa@mcmaster.ca

Total Pages: 125, Total Figures: 148 figures, 5 schemes, 4 tables

Off-setting Low Affinity Carbohydrate Binding with Covalency, To Engage Sugar Specific Proteins For Tumor-Immune Proximity Induction

Benjamin P.M. Lake[⊥§], Anthony F. Rullo[∝⊥§*]

Affiliations: [⊥]Department of Medicine, McMaster Immunology Research Center, Center for Discovery in Cancer Research, [∝]Department of Biochemistry and Biomedical Sciences, [§]Department of Chemistry and Chemical Biology. McMaster University, 1280 Main Street West, Hamilton Ontario, Canada.

*Corresponding author: Anthony F. Rullo, email: rulloa@mcmaster.ca

KEYWORDS (Glyco-covalent therapeutics, proximity inducing carbohydrates, covalent chimera, glycoconjugates, synthetic glyco-immunotherapy).

ABSTRACT:

Carbohydrate-binding proteins "xCarbs" are often used by the innate immune system to potentiate inflammation, target endocytosis/destruction, and adaptive immunity (e.g. CD206, DC-SIGN, MBL, anti-carbohydrate antibodies). To access xCarbs for cancer immunotherapy, a growing repertoire of bi-functional proximity inducing therapeutics use high avidity multivalent carbohydrate binding domains to off-set the intrinsically low affinity associated with monomeric carbohydrate : xCarb binding interactions (K_d ~10^{-3}-10^{-6} M). For applications aimed at recruiting anti-carbohydrate antibodies to tumor cells, large synthetic scaffolds are used that contain both a tumor-binding-domain (TBD) and a multivalent antibody binding domain "ABD" comprising multiple L-rhamnose monosaccharides. This allows for stable bridging between tumor cells and xCarbs which activates tumoricidal immune function. Problematically, such multivalent macromolecules can face limitations including synthetic and/or structural complexity, and the potential for off-target immune-engagement. We envisioned small bi-functional molecules containing a low affinity monovalent ABD could promote xCarb dependent tumor-immune proximity, by coupling xCarb binding *with covalent engagement*. Typical covalent drugs and electrophilic chimeras use high affinity ligands to promote fast covalent engagement of target proteins (i.e. large k_{inact}/K_I), driven by a favorably small K_I for binding. We hypothesized the much less favorable K_I associated with xCarb engagement of sugars can be off-set by a favorably large k_{inact} for the covalent labeling step. In the current study, we test this hypothesis and explore covalent "xCarb" engagement in the context of a model system that uses rhamnose specific antibodies to induce tumor-immune proximity and tumoricidal function. We discovered that synthetic chimeric molecules capable of pre-organizing an optimal electrophile (i.e. SuFEx vs. activated ester) for xCarb engagement, can rapidly covalently engage natural sources of anti-rhamnose antibody, *using only a single low affinity rhamnose monosaccharide ABD*. Strikingly, we observe chimeric molecules lacking an electrophile which can only non-covalently bind xCarbs, completely lack tumoricidal function. This is in stark contrast to previous work targeting small molecule hapten and peptide specific antibodies. Our findings underscore the utility of covalency as a strategy to engage low affinity carbohydrate specific proteins for tumor-immune proximity induction.

Table of Contents:

Synthesis Procedures	S1-24
General Notes and Materials	S25-26
Experimental Procedures (Complementing Main Text)	S27-40
Spectra and Characterization Data	S41-123
References	S123

Synthetic Procedures

General materials and methods for organic synthesis and instrumentation

All chemical reagents and solvents were obtained from commercial suppliers (Sigma Aldrich, Fisher Scientific, Chem-Impex, Broadpharm) and used without further purification. All reactions were conducted at room temperature unless otherwise stated. Thin layer chromatography (TLC) was performed on silica gel precoated aluminium sheets (Silicycle) and visualized by fluorescence, ninhydrin, and potassium permanganate staining. All column chromatography purification was conducted using a Buchi Pure C-810 Flash purification system using normal phase silica gel (Buchi) or reverse phase C18 columns (Buchi). ^{1}H, ^{13}C, and ^{1}H-^{1}H COSY NMR spectra were all recorded a Bruker 400 or 700 MHz spectrometer. ^{1}H NMR spectra was calibrated to solvent peak (($CD_2H)_2SO$, δ 2.50), (CD_3OD, δ 3.31), (D_2O, δ 4.79) or ($CDCl_3$, δ 7.26). ^{1}C NMR spectra was calibrated to solvent peak (($CD_2H)_2SO$, δ 39.52), (CD_3OD, δ 49.00) or ($CDCl_3$, δ 77.16). Electrospray ionization quadrupole Fourier transform mass spectroscopy (ESI-MS) data was obtained using a Waters QUATTRO mass spectrometer, while LCMS data was obtained on an Agilent-Sciex QTRAP system or an LTQ Orbitrap XL system using a gradient of 95:5 to 5:95 water (0.1% formic acid):ACN (0.1% formic acid). HRMS-ESI was obtained with a BRUKER MicroTOF II mass spectrometer. Where indicated, a ThermoFisher DIONEX UltiMate 3000 UHPLC+ was used for HPLC purification with a gradient of 95:5 to 5:95 water (0.1% formic acid):ACN (0.1% formic acid).

Note: Proton assignments for ^{1}H NMR spectra (Beyond figure S14) are numbered sequentially from downfield (1) to upfield.

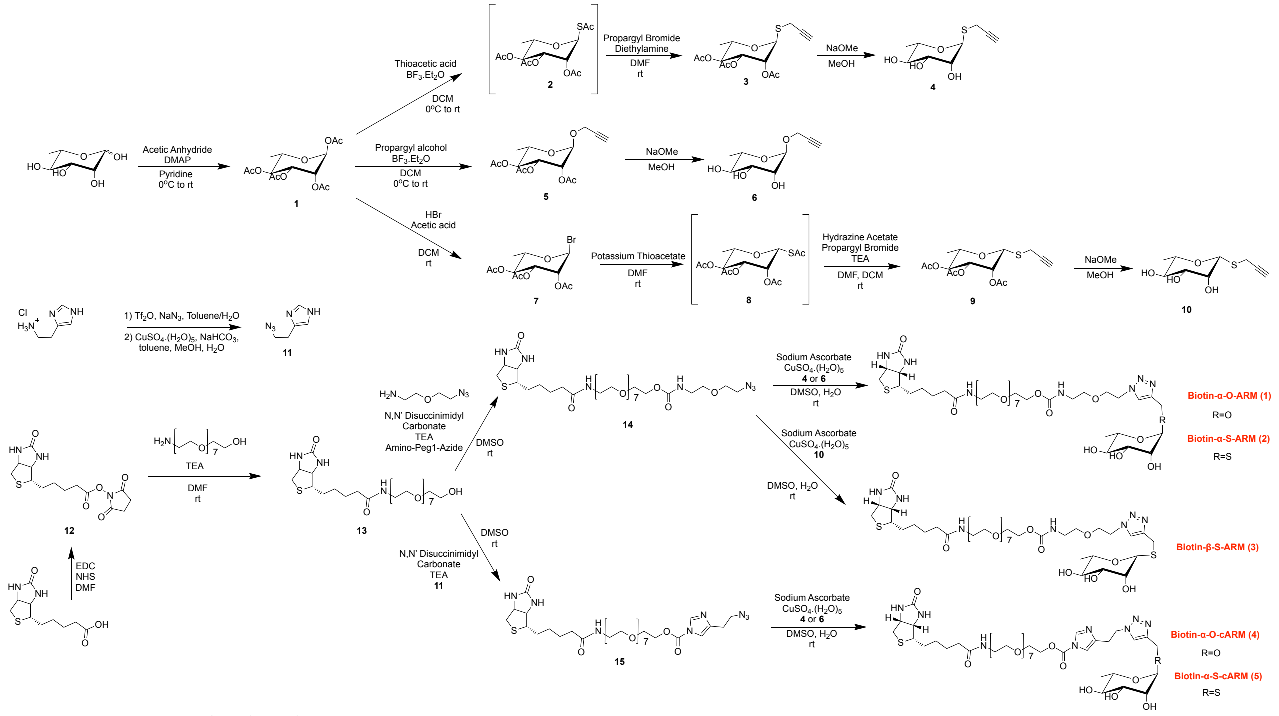

Scheme S1. Synthesis of **Rha-Biotin cARMs/ARMs (1-5)**

Note: Synthesis of Compound 11 adapted from previous work.[1]

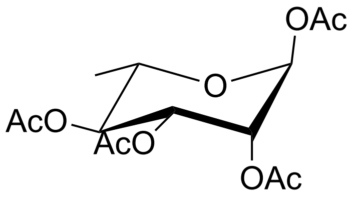

Peracetylated L-Rhamnose (1). L-rhamnose (9 g, 54.8 mmol) was added to a vacuum dried flask. The flask was capped with a rubber septum and maintained under a nitrogen atmosphere. To this flask, pyridine (40 mL, 494.6 mmol) was added followed by DMAP (187 mg, 1.5 mmol). This solution was placed in an ice bath for 15 minutes followed by the addition of acetic anhydride (31 mL, 328.6 mmol) dropwise over 15 minutes with stirring. This mixture was removed from the ice bath and allowed to stir, warming to room temperature overnight. The reaction mixture was dried under air flow, and resuspended in 300 mL EtOAc. This solution was washed 3X with each of 1M HCl, sat. sodium bicarbonate, and finally brine. The organic layer was dried over Mg_2SO_4 and concentrated to dryness under reduced pressure yielding **1** with partially acetylated impurity (14.224 g, 78% yield, about 20% partially acetylated impurity). ^{1}H NMR (700 MHz, $CDCl_3$) δ 6.00 (d, J = 1.5 Hz, 1H), 5.29 (dd, J = 9.2, 3.5 Hz, 1H), 5.24 (m, 1H), 5.11 (dd, J = 10.0, 10.0 Hz, 1H), 3.93 (dq, J = 9.9, 6.2 Hz, 1H), 2.16 (s, 3H), 2.14 (s, 3H), 2.05 (s, 3H), 1.99 (s, 3H), 1.22 (d, J = 6.2 Hz, 3H). ^{13}C NMR (176 MHz, $CDCl_3$) δ 170.08, 169.82, 168.38, 90.64, 70.47, 68.72, 20.91, 17.44.

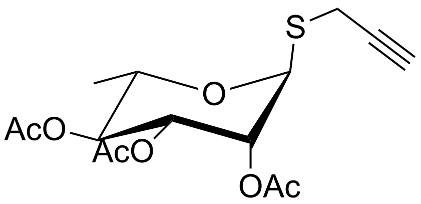

Peracetyl-α-S-Propargyl Rhamnose (3). Into a vacuum dried flask was added **1** (492 mg, 1.5 mmol). The flask was capped with a rubber septum and maintained under an argon atmosphere. To this flask anhydrous DCM (5 mL) was added followed by thioacetic acid (130 µL, 1.7 mmol). This mixture was placed in an ice bath for 15 minutes followed by the addition of $BF_3.Et_2O$ (460 µL, 3.7 mmol) dropwise over 5 minutes with stirring. This solution was allowed to stir in the ice bath overnight, slowly warming to room temperature. This mixture was quenched with 60 mL sat. sodium bicarbonate (added portion wise) and stirred until bubbling subsided. This solution was extracted with 50 mL EtOAc. The organic layer was washed 2X with sat. sodium bicarbonate followed by three washes with sat. brine. The organic layer was dried with Mg_2SO_4 and solvent removed under reduced pressure to yield crude **2** which was used directly in the next step.

Into a vacuum dried flask **2** (186 mg, 0.5 mmol) was added and dissolved in anhydrous DMF (2.5 mL). Propargyl bromide (48 µL, x 0.4 mmoL) was then added under argon atmosphere followed by DEA (92 µL, 0.9 mmol). The reaction was stirred for 1 hour, then the solvent was removed under reduced pressure. This crude product was resuspended in DCM and washed 2X with 0.1M HCl and 3X with sat. brine. The organic layer was dried with Mg_2SO_4 and dried under reduced pressure. The resulting crude product was purified via flash chromatography (isocratic 3:1, Hexanes:EtOAc) to yield **3** (67.2 mg, 26.3% over two steps). ^{1}H NMR (700 MHz, $CDCl_3$) δ 5.41 (s, J = 1 Hz, 1H), 5.38 (m, 1H), 5.19 (dd, J = 10.1, 3.3 Hz, 1H), 5.12 (dd, J = 9.9, 9.8 Hz, 1H), 4.20 (dq, J = 9.5, 6.1 Hz, 1H), 3.32 (dd, J = 113.3, 16.7 Hz, 2H), 2.26 (m, 1H), 2.17 (s, 3H), 2.05 (s, 3H), 1.98 (s, 3H), 1.25 (d, *J* = 6.2 Hz, 3H). ^{13}C NMR (176 MHz, $CDCl_3$) δ 169.8, 81.2, 78.6, 71.7, 71.0, 70.7, 69.4, 67.3, 20.8, 18.1, 17.1.

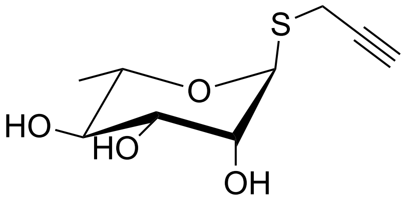

α-S-Propargyl Rhamnose (4). **3** (67.2 mg, 0.2 mmol) was dried under vacuum, then resuspended in MeOH (5 mL) followed by addition of sodium methoxide solution (280 µL, 25 wt% in MeOH). The solution was stirred for 20 minutes then diluted with 10 mL water and quenched to pH 7 with a 0.1 M HCl solution. The solution was flash frozen and lyophilized to yield **4** with a benign methyl acetate impurity in quantitative yields (100%). ^{1}H NMR (700 MHz, MeOD) δ 5.39 (d, *J* = 0.9 Hz, 1H), 3.94 (dd, J = 1.4, 3.4 Hz, 1H), 3.90 (qd, J = 6.2, 9.4 Hz, 1H), 3.58 (dd, J = 3.5, 9.5 Hz 1H), 3.44 (dd, J = 9.5, 9.5 Hz, 1H), 3.35 (s, 1H), 3.32 (dd, J = 16.8, 99.0 Hz, 2H), 1.89 (s, 1H), 1.29 (d, *J* = 6.2 Hz, 3H). ^{13}C NMR (176 MHz, MeOD) δ 179.1, 84.1, 72.7, 72.0, 70.8, 69.2, 22.9, 17.1, 16.5.

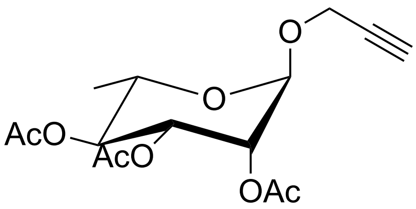

Peracetyl α-O-Propargyl Rhamnose (5). Into a vacuum dried flask was added **1** (500 mg, 1.5 mmol). The flask was capped with a rubber septum and maintained under a nitrogen atmosphere. To this flask anhydrous DCM (5 mL) was added followed by propargyl alcohol (105 µL, 1.8 mmol). This solution was placed in an ice bath for 15 minutes followed by the addition of $BF_3.Et_2O$ (460 µL, 3.7 mmol) dropwise over 5 minutes with stirring. This mixture was allowed to stir in the ice bath overnight, slowly warming to room temperature. This mixture was quenched with 60 mL sat. sodium bicarbonate (added portion wise) and stirred until bubbling subsided. This solution was extracted with 50 mL EtOAc. This organic layer was washed 2X more with sat. sodium bicarbonate followed by 3X with sat. brine. The organic layer was dried with Mg_2SO_4 and solvent removed under reduced pressure. The crude product was purified via flash chromatography (isocratic 3:1, Hexanes:EtOAc) to yield **5** (234.2 mg, 47.6%). ^{1}H NMR (700 MHz, $CDCl_3$) δ 5.28 (dd, J = 3.5, 10 Hz, 1H), 5.26 (dd, J = 1.8, 3.4 Hz, 1H), 5.07 (dd, J = 9.9, 9.9 Hz, 1H), 4.93 (d, *J* = 1.5 Hz, 1H), 4.25 (m, 2H), 3.89 (dq, J = 9.7, 6.2 Hz, 1H), 2.45 (t, *J* = 2.4 Hz, 1H), 2.15 (s, 3H), 2.04 (s, 3H), 1.98 (s, 3H), 1.22 (d, *J* = 6.3 Hz, 3H). ^{13}C NMR (176 MHz, $CDCl_3$) δ 170.00, 96.11, 78.22, 75.25, 70.95, 69.66, 68.98, 66.90, 54.71, 20.81, 17.30.

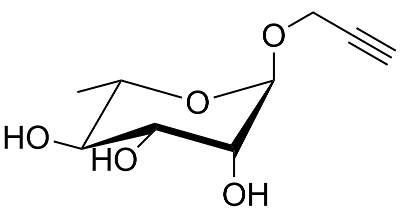

α-O-Propargyl Rhamnose (6). **5** (234.2 mg, 0.7 mmol) was dried under vacuum, then resuspended in MeOH (5 mL) followed by addition of sodium methoxide solution (280 µL, 25 wt% in MeOH). The solution was stirred for 20 minutes then diluted with 10 mL water and quenched to pH 7 with a 0.1 M HCl solution. The solution was flash frozen and lyophilized to yield **6** with a benign methyl acetate impurity in quantitative yields (100%). ^{1}H NMR (700 MHz, D_2O) δ 4.99 (d, J = 1.3 Hz, 1H), 4.34 (dd, *J* = 21.1, 16.0 Hz, 2H), 3.97 (dd, J = 3.3, 1.6 Hz, 1H), 3.77 (m, 2H), 3.48 (dd, J = 9.7, 9.7 Hz, 1H), 2.75 (s, 3H), 1.94 (s, 3H), 1.32 (d, *J* = 6.3 Hz, 3H). ^{13}C NMR (176 MHz, D_2O) δ 181.5, 98.9, 78.4, 75.9, 71.9, 70.1, 69.1, 54.7, 38.8, 23.3, 16.5.

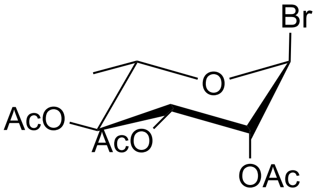

α-O-Bromo Rhamnose (7). To a flame dried flask, **1** (2 g, 6.02 mmol) was dissolved in anhydrous DCM (50 mL). The flask was placed in a room temperature water bath and shielded from light. To this flask, HBr was added (7.5 mL, 33% in acetic acid, 30.6 mmol). This solution was stirred in the dark for 2 hours, then added portion wise to ice cold sodium bicarbonate solution (10 g in 75 mL water) while stirring. When the bubbling subsided, the solution was moved to a separatory funnel for isolation of organic layer. The separated aqueous layer was extracted with DCM (25 mL). Combined organic layers were dried over Na_2SO_4, and concentrated under reduced pressure to yield **7** to be used directly in the next step (1.043 g, 49% yield). ^{1}H NMR (700 MHz, $CDCl_3$) δ 6.25 (d, J = 1.5 Hz, 1H), 5.66 (dd, 10.2, 3.4 Hz, 1H), 5.44 (dd, J = 3.4, 1.5 Hz, 1H), 5.15 (dd, J = 10.1, 10.1 Hz, 1H), 4.10 (dq, J = 9.9, 6.2 Hz, 1H), 2.16 (s, 3H), 2.07 (s,

3H), 2.00 (s, 3H), 1.28 (d, J = 6.3 Hz, 1H). ^{13}C NMR (176 MHz, $CDCl_3$) δ 169.8, 83.7, 72.5, 71.1, 70.3, 67.9, 20.8, 17.0.

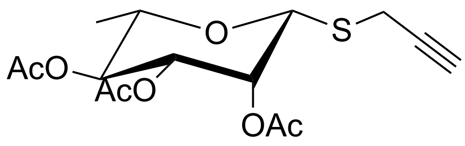

Peracetyl β-S-Propargyl Rhamnose (9). **7** (261 mg, 0.74 mmol) was dissolved in anhydrous DMF (1.7 mL) with potassium thioacetate (564 mg, 4.94 mmol), stirred 2 hours, then evaporated under a stream of nitrogen. The resulting oil was dissolved in EtOAc and washed 3x with 50% saturated brine. The organic layer was dried over Na_2SO_4 and concentrated under reduced pressure to yield a crude oil. The oil was resuspended in DCM and purified via flash chromatography (98:2 to 95:5, DCM:EtOAc) to give semi-pure product (intermediate **8)** used directly in the next reaction (62.5 mg, 24.3% crude yield). ^{1}H NMR (700 MHz, $CDCl_3$) δ 5.46 (m, 2H), 5.05 (m, 2H), 3.67 (m, 1H), 2.34 (s, 3H), 2.16 (s, 3H), 2.04 (s, 3H), 1.95 (s, 3H), 1.24 (m, 3H).

Intermediate **8** (62.5 mg, 0.18 mmoL) was dissolved in anhydrous DMF (650 µL) and anhydrous DCM (650 µL), followed by addition of hydrazine acetate (18.7 mg, 0.2 mmol). This solution was stirred for 1 hour followed by addition of propargyl bromide (32 mg, 0.27 mmol) and TEA (35 µL, 0.25 mmol). This solution was stirred overnight, then quenched with acetic acid solution (100 µL acetic acid in 50 mL EtOAc). This was washed 3x with brine, the organic layer dried over Na_2SO_4 , followed by evaporation under reduced pressure. The resulting crude oil was purified via flash chromatography (20:80 to 25:75 EtOAc : Hexanes) to yield **9** (15.1 mg, 24.5% yield). ^{1}H NMR (700 MHz, $CDCl_3$) δ 5.54 (dd, J = 3.2, 1.1 Hz, 1H), 5.10 (m, 2H), 5.04 (d, J = 1.1 Hz, 1H), 3.61 (dq, J = 9.0, 6.2 Hz, 1H), 3.42 (ddd, J = 213.9, 16,8, 2.6 Hz, 2H), 2.30 (t, J = 2.6 Hz, 1H), 2.19 (s, 3H), 2.06 (s, 3H), 1.99 (s, 3H), 1.31 (d, J = 6.2 Hz, 1H). ^{13}C NMR (176 MHz, $CDCl_3$) δ 170.17, 80.34, 78.96, 75.08, 71.94, 70.45, 20.63, 18.24, 17.65.

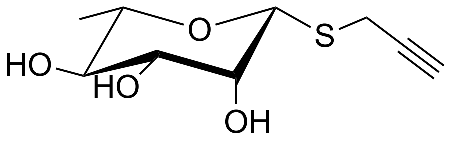

β-S-Propargyl Rhamnose (10). **9** (15.1 mg, 0.04 mmol) was dried under vacuum, then resuspended in MeOH (5 mL) followed by addition of sodium methoxide solution (280 µL, 25 wt% in MeOH). The solution was stirred for 20 minutes then diluted with 10 mL water and quenched to pH 7 with a 0.1 M HCl solution. The solution was flash frozen and lyophilized to yield semi-pure **10** with a benign methyl acetate impurity, in quantitative yields (100%). ^{1}H NMR (700 MHz, D_2O) δ 5.04 (d, J = 0.5 Hz, 1H), 4.07 (dd, J = 3.5, 0.5 Hz, 1H), 3.66 (dd, J = 9.7, 3.6 Hz, 1H), 3.51 (dd, J = 92.5, 17.1 Hz, 2H), 3.47 (m, 1H), 3.40 (m, 1H), 1.32 (d, J = 6.1 Hz, 1H).

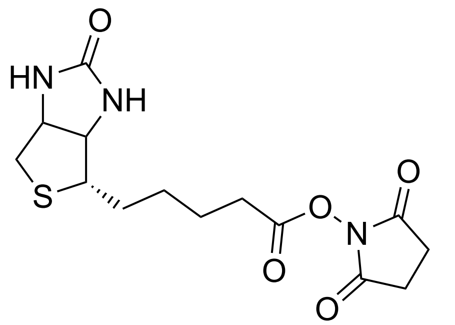

Biotin-NHS (12). Biotin (335.4 mg, 1.37 mmol), EDC.HCl (283.1 mg, 1.48 mmol), and NHS (241.5 mg, 2.1 mmol) were dissolved together in DMF (3 mL). This solution was stirred and heated with a heat gun until all components fully dissolved into a clear colourless solution. The mixture was stirred overnight, yielding an opaque white solution. Precipitate was collected via vacuum filtration, washing with ice cold water, and further dried under reduced pressure to yield **12** (219.3 mg, 46.9% yield). ^{1}H NMR (700 MHz, DMSO) δ 6.41 (s, 1H), 6.36 (s, 1H), 4.30 (m, 1H), 4.14 (m, 1H), 3.10 (m, 1H), 2.81 (m, 4H), 2.71 (m, 2H), 2.67 (m, 2H), 1.66 (m, 2H), 1.57 (m, 2H), 1.42 (m, 2H). ^{13}C NMR (176 MHz, DMSO) δ 170.15, 168.82, 162.55, 60.87, 59.05, 55.11, 29.88, 27.72, 27.46, 25.33, 24.19.

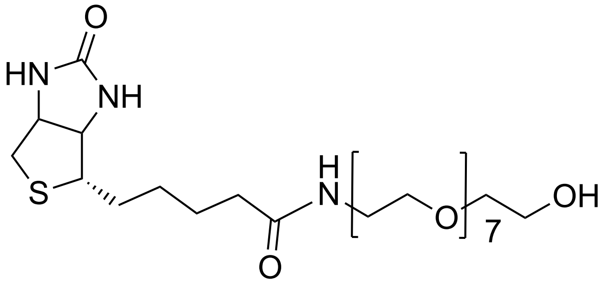

Biotin-Peg8-Hydroxy (13). Biotin (466 mg, 1.91 mmol), EDC.HCl (399 mg, 2.08 mmol), and NHS (356 mg, 3.09 mmol) were dissolved in DMF (4.2 mL). This solution was stirred and heated with a heat gun until all components were fully dissolved. The solution was allowed to stir overnight, then added directly to amino-peg8-OH (792 mg, 2.1 mmol) followed by TEA. (799 µL, 5.73 mmol). This solution was stirred overnight, then precipitated out of cold ether (45 mL) followed by centrifugation. The pellet was collected, resuspended in DMF, and precipitated out of cold ether followed by centrifugation. The pellet was purified by HPLC to yield **13** (447.1 mg, 39.3% yield over two steps). ^{1}H NMR (700 MHz, DMSO) δ 7.82 (t, J = 5.6 Hz, 1H), 6.41 (m, 1H), 6.34 (s, 1H), 4.56 (m, 1H), 4.30 (m, 1H), 4.12 (m, 1H), 3.48 (m, 2H), 3.41 (t, J = 5.3 Hz, 2H), 3.39 (t, J = 5.9 Hz, 2H), 3.18 (m, 2H), 3.10 (m, 1H), 2.70 (m, 2H), 2.13 (m, 2H), 1.53 (m, 2H), 1.50 (m, 2H), 1.31 (m, 2H). ^{13}C NMR (176 MHz, DMSO) δ 174.91, 172.56, 163.16, 72.81, 70.25, 69.64, 61.50, 60.68, 59.65, 55.89, 38.91, 35.56, 33.97, 28.58, 25.73, 25.01. HRMS-ESI $[M+H]^{+}$ *m/z* calc for $[C_{26}H_{50}N_{3}0_{10}S^{+}]$ 596.3211, found 596.4440.

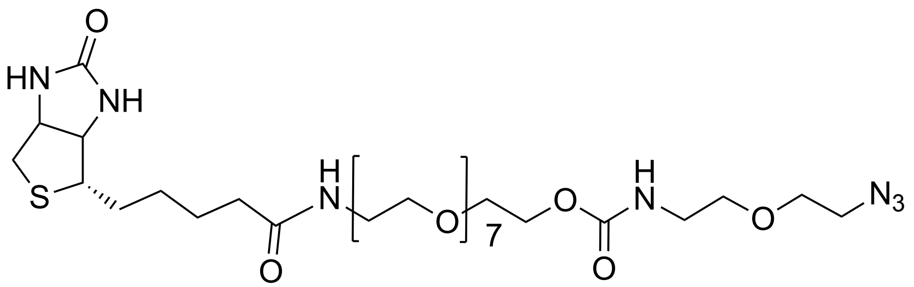

Biotin-Peg8-Peg1-Azide (14). **13** (100 mg, 0.17 mmol) was dissolved in DMSO (1 mL) with N,N'-disuccinimidyl carbonate (51.6 mg, 0.2 mmol) and TEA (32.8 µL, 0.24 mmol). This solution was stirred for 4 hours, then crashed in cold ether. The supernatant was removed, and the reaction mixture was crashed in cold ether again. The supernatant was removed, and the remaining ether was removed under reduced pressure. To the crude solution, DMSO (1 mL) was added followed by amino-peg1-azide (35.7 mg, 0.27 mmol) and TEA (46.8 µL, 0.34 mmol). This solution was stirred overnight, then purified via HPLC to yield **14** (46.4 mg, 36.7% yield). ^{1}H NMR (700 MHz, DMSO) δ 7.82 (t, *J* = 5.6 Hz, 1H), 7.21 (t, *J* = 5.5 Hz, 1H), 6.41 (s, 1H), 6.35 (s, 1H), 4.30 (m, 1H), 4.13 (m, 1H), 4.04 (t, *J* = 4.7 Hz, 2H), 3.58 (t, *J* = 5.0 Hz, 2H), 3.56 (t, *J* = 4.7 Hz, 2H), 3.50 (m, 24H), 3.44 (m, 2H), 3.39 (m, 4H), 3.18 (m, 2H), 3.14 (m, 2H), 3.10 (m, 1H), 2.70 (m, 2H), 2.06 (t, *J* = 7.5 Hz, 2H), 1.53 (m, 2H), 1.51 (m, 2H), 1.30 (m, 2H). ^{13}C NMR (176 MHz, DMSO) δ 172.13, 162.72, 156.26, 69.81, 69.21, 68.97, 68.95, 63.19, 61.06, 59.21, 55.45, 50.00, 38.47, 35.12, 28.23, 28.07, 25.29. HRMS-ESI $[M+H]^+$ *m/z* calc for $[C_{31}H_{58}N_{7}O_{12}S^{+}]$ 752.3859, found 752.6223.

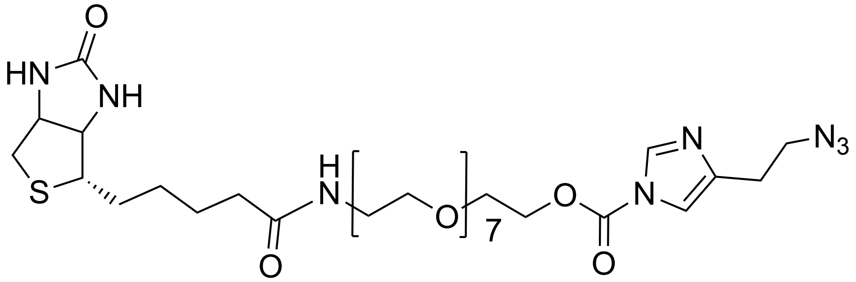

Biotin-Peg8-AI-Azide (15). **13** (71.8 mg, 0.12 mmol) was dissolved in DMSO (1 mL) with N,N'-disuccinimidyl carbonate (37 mg, 0.14 mmol) and TEA (23.5 µL, 0.17 mmol). This solution was stirred for 4 hours, then crashed in cold ether. The supernatant was removed, and the reaction mixture was crashed in cold ether again. The supernatant was removed, and the remaining ether was removed under reduced pressure. To the crude solution, DMSO (300 µL) was added with **11** (26.4 mg, 0.19 mmol) and pyridine (39 µL, 0.482 mmol). This solution was stirred for 2 hours, then purified via HPLC to yield **15** (20.9 mg, 23% yield). ^{1}H NMR (700 MHz, DMSO) δ 8.20 (d, *J* = 1.2 Hz, 1H), 7.82 (t, *J* = 5.6 Hz, 1H), 7.43 (d, *J* = 1.1 Hz, 1H), 6.40 (s, 1H), 6.34 (s, 1H), 4.48 (m, 2H), 4.30 (m, 1H), 4.12 (m, 1H), 3.76 (m, 2H), 3.59 (m, 2H), 3.50 (m, 24H), 3.39 (t, *J* = 6.0 Hz, 2H), 3.18 (m, 2H), 3.09 (m, 1H), 2.77 (m, 2H), 2.70 (m, 2H), 2.06 (t, *J* = 7.5 Hz, 2H), 1.53 (m, 2H), 1.51 (m, 2H), 1.30 (m, 2H). ^{13}C NMR (176 MHz, DMSO) δ 171.98, 162.57, 148.12, 140.14, 136.83, 114.09, 69.66, 69.45, 69.06, 67.74, 67.02, 60.91, 59.07, 55.30, 49.33, 38.32, 34.97, 28.08, 27.12, 25.14. HRMS-ESI $[M+H]^+$ *m/z* calc for $[C_{32}H_{55}N_{8}O_{11}S^{+}]$ 759.3706, found 759.7325.

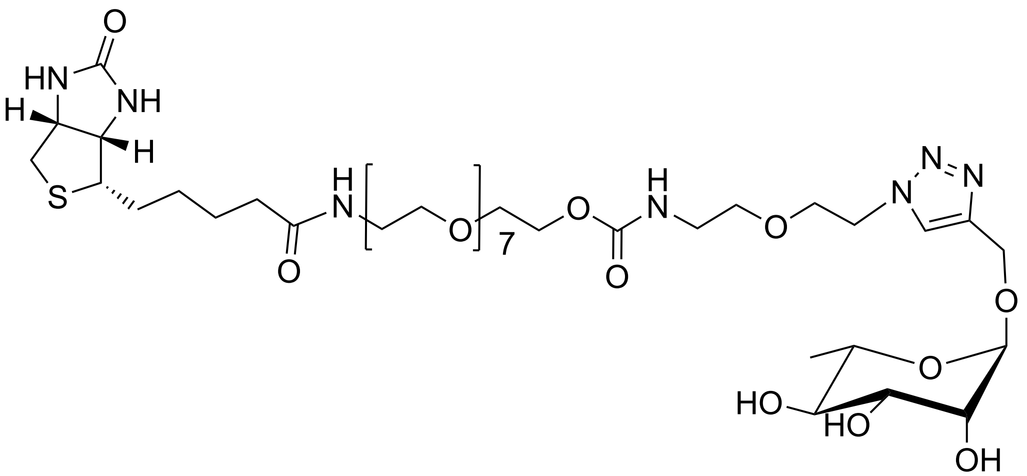

Biotin-α-O-ARM (1). **14** (14.1 mg, 0.02mmol) was dissolved in DMSO (200 μL). To this solution, **6** (12.1 mg, 0.06 mmol) was added with sodium ascorbate (18.6 mg, 0.09 mmol), $CuSO_4$ (4.7 mg, 0.02 mmol) and water (215 μL). After 1 hour of stirring, the crude solution was purified via HPLC to yield pure product (9.8 mg, 54.8% yield). ^{1}H NMR (700 MHz, D_2O_salt) δ 8.14 (s, 1H), 4.96 (s, 1H), 4.86 (m, 2H), 4.69 (d, *J* = 4.9 Hz, 2H), 4.67 (m, 1H), 4.48 (m, 1H), 4.25 (t, *J* = 4.4 Hz, 2H), 4.01 (t, *J* = 4.9 Hz, 2H), 3.98 (m, 1H), 3.80 (m, 2H), 3.78 (m, 1H), 3.75 (m, 24H), 3.68 (m, 3H), 3.61 (t, 2H), 3.49 (m, 1H), 3.44 (t, *J* = 5.3 Hz, 2H), 3.39 (m, 1H), 3.31 (m, 2H), 2.94 (m, 2H), 2.33 (t, *J* = 7.3 Hz, 2H), 1.70 (m, 2H), 1.70 (m, 2H), 1.46 (m, 2H), 1.28 (d, *J* = 6.2 Hz, 3H). ^{13}C NMR (176 MHz, D_2O_salt) δ 176.95, 165.37, 143.72, 125.55, 99.76, 72.05, 70.23, 70.14, 69.64, 69.48, 68.93, 68.81, 68.61, 64.21, 62.13, 60.30, 60.17, 55.41, 50.16, 39.78, 38.97, 35.54, 30.38, 27.95, 27.76, 25.21, 16.66. HRMS-ESI $[M+H]^+$ *m/z* calc for $[C_{40}H_{72}N_{7}0_{17}S^+]$ 954.4700, found 954.6917.

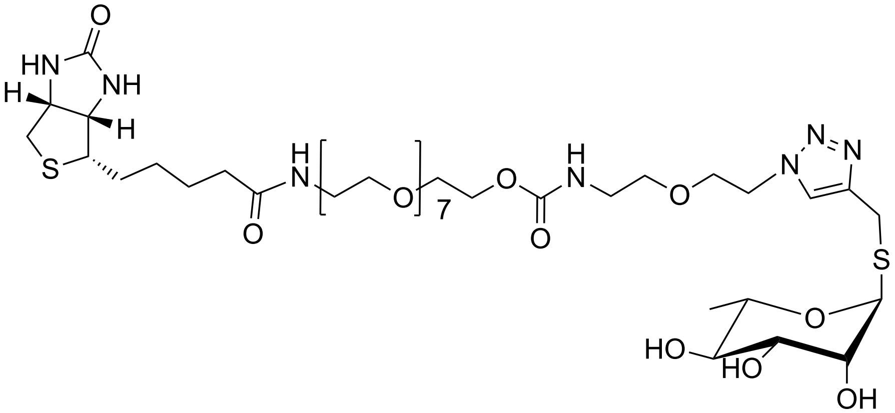

Biotin-α-S-ARM (2). **14** (14.1 mg, 0.02 mmol) was dissolved in DMSO (200 μL). To this solution, **4** (13.1 mg, 0.06 mmol) was added with sodium ascorbate (18.6 mg, 0.09 mmol), $CuSO_4$ (4.7 mg, 0.02 mmol) and water (262 μL). After 1 hour of stirring, the crude solution was purified via HPLC to yield pure product (10.7 mg, 58.8% yield). ^{1}H NMR (700 MHz, D_2O_salt) δ 8.04 (s, 1H), 5.24 (s, 1H), 4.67 (m, 3H), 4.49 (m, 1H), 4.26 (m, 2H), 4.06 (m, 1H), 4.00 (m, 4H), 3.95 (m, 1H), 3.82 (m, 2H), 3.77 (m, 1H), 3.75 (m, 24H), 3.69 (t, *J* = 5.3 Hz, 2H), 3.61 (t, *J* = 5.2 Hz, 2H), 3.50 (m, 1H), 3.45 (t, *J* = 5.3 Hz, 2H), 3.39 (m, 1H), 3.33 (m, 2H), 2.95 (m, 2H), 2.34 (t, *J* = 7.3 Hz, 2H), 1.71 (m, 2H), 1.71 (m, 2H), 1.47 (m, 2H), 1.22 (d, *J* = 6.2 Hz, 3H). ^{13}C NMR (176 MHz, D_2O_salt) δ 176.95, 165.37, 145.02, 124.54, 84.72, 72.45, 71.70, 70.94, 69.76, 69.65, 69.49, 69.15, 68.94, 68.60, 64.24, 62.14, 60.31, 55.41, 50.15, 40.07, 39.79, 38.98, 35.55, 27.96, 27.78, 25.21, 24.54, 16.63. HRMS-ESI $[M+H]^+$ *m/z* calc for $[C_{40}H_{72}N_{7}0_{16}S_2^+]$ 970.4471, found 970.6523.

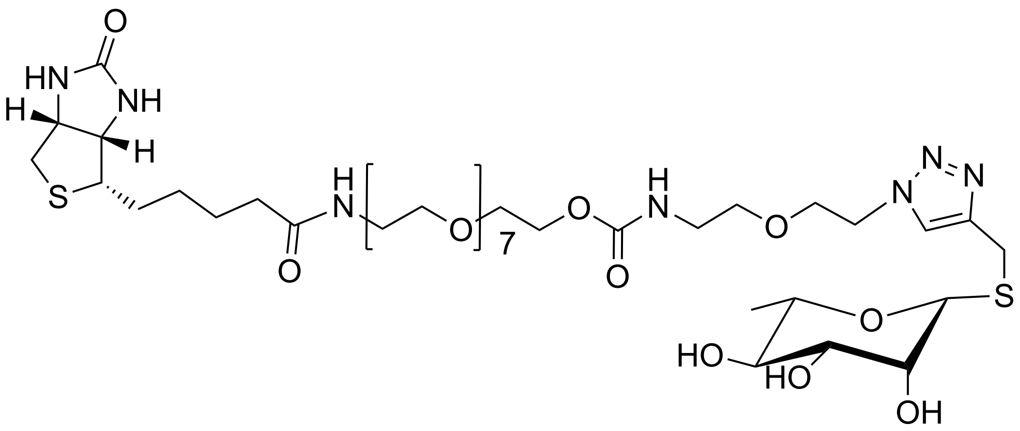

Biotin-β-S-ARM (3), **14** (5 mg, 0.007 mmol) was dissolved in DMSO (90 µL). To this solution, **10** (6.4 mg, 0.03 mmol) was added with sodium ascorbate (18.6 mg, 0.09 mmol), $CuSO_4$ (4.7 mg, 0.02 mmol) and water (200 µL). After 1 hour of stirring, the crude solution was purified via HPLC to yield pure product (2.1 mg, 32.3% yield). ^{1}H NMR (700 MHz, D_2O_salt) δ 8.00 (s, 1H), 4.72 (s, 1H), 4.63 (m, 3H), 4.45 (m, 1H), 4.22 (t, J = 4.4 Hz, 2H), 4.04 (m, 2H), 3.98 (m, 3H), 3.78 (t, J = 4.4 Hz, 2H), 3.72 (m, 24H), 3.65 (t, J = 5.3 Hz, 2H), 3.58 (m, 3H), 3.41 (t, J = 5.3 Hz, 2H), 3.36 (m, 3H), 3.29 (m, 2H), 2.91 (m, 2H), 2.30 (t, J = 7.3 Hz, 2H), 1.68 (m, 2H), 1.67 (m, 2H), 1.43 (m, 2H), 1.26 (d, J = 5.9 Hz, 3H). ^{13}C NMR (176 MHz, D_2O_salt) δ 176.91, 165.34, 145.24, 124.49, 83.73, 76.38, 73.51, 72.14, 71.88, 69.62, 69.44, 68.90, 68.57, 64.18, 62.10, 60.27, 55.37, 50.12, 39.99, 39.73, 38.93, 35.49, 27.91, 25.16, 24.47, 16.98. HRMS-ESI $[M+H]^+$ m/z calc for $[C_{40}H_{72}N_7 0_{16}S_2{}^+]$ 970.4471, found 970.6455.

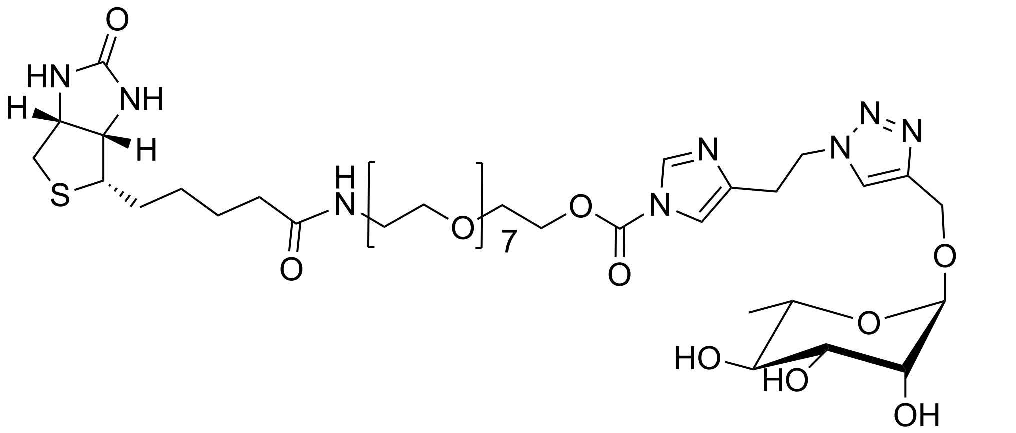

Biotin-α-O-cARM (4). **15** (9.4 mg, 0.01 mmol) was dissolved in DMSO (200 µL). To this solution, **6** (8 mg, 0.04 mmol) was added with sodium ascorbate (12.3 mg, 0.06 mmol), $CuSO_4$ (3.1 mg, 0.01 mmol) and water (215 µL). After 1 hour of stirring, the crude solution was purified via HPLC and pH neutralized (pH ~7) prior to lyophilization to yield pure product (4.5 mg, 37.7% yield). HRMS-ESI $[M+H]^+$ m/z calc for $[C_{41}H_{69}N_8 0_{16}S^+]$ 961.4547, found 961.5432.

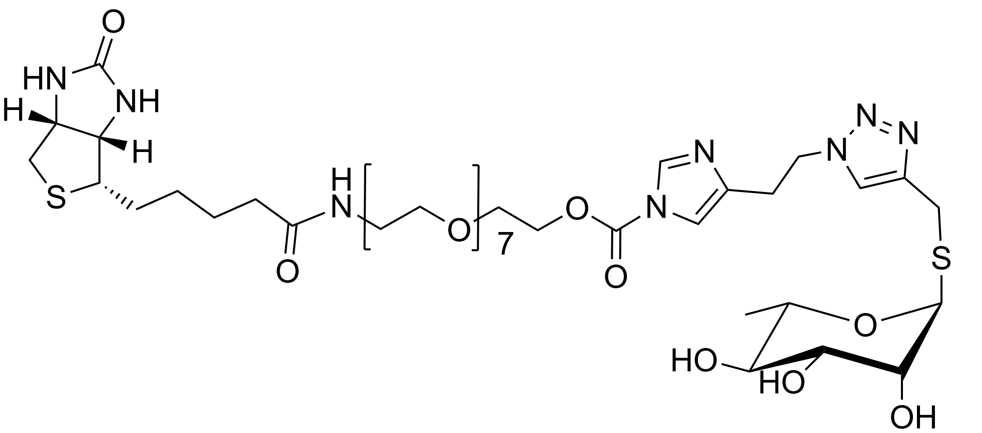

Biotin-α-S-cARM (5). **15** (9.4 mg, 0.01 mmol) was dissolved in DMSO (200 µL). To this solution, **4** (10.8 mg, 0.05 mmol) was added with sodium ascorbate (12.3 mg, 0.06 mmol), $CuSO_4$ (3.1 mg, 0.01 mmol) and water (215 µL). After 1 hour of stirring, the crude solution was purified via HPLC and pH neutralized (pH ~7) prior to lyophilization to yield pure product (0.2 mg, 1.5% yield). HRMS-ESI $[M+H]^+$ *m/z* calc for $[C_{41}H_{69}N_80_{15}S_2^+]$ 977.4318, found 977.7661.

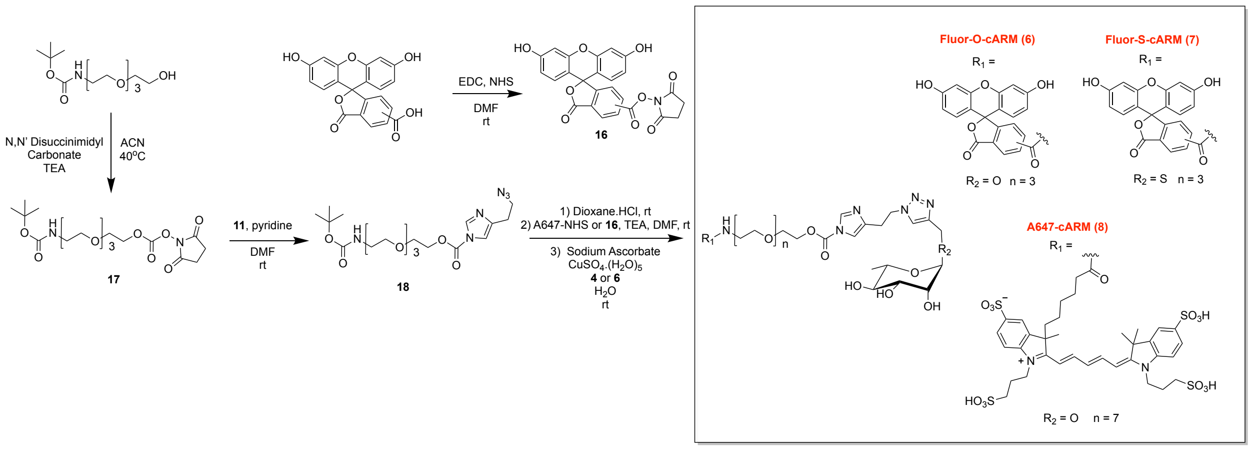

Scheme S2. Synthesis of Fluorescent **cARMs (6-8)**

Note: Compounds 16 and 17 adapted from previous work.[2]

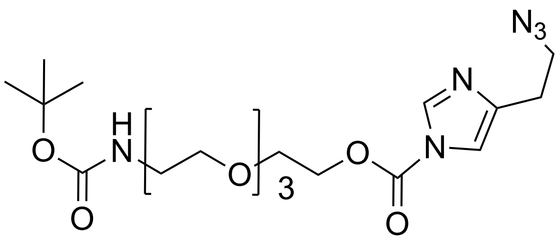

Boc-Peg4-AI-Azide (18). 17 (67 mg, 0.15 mmol) was dried under vacuum, then dissolved in anhydrous DMF (1 mL). To this solution, **11** (30 mg, 0.22 mmol) was added with pyridine (44 µL, 0.55 mmol). This mixture was stirred for 2 hours, followed by purification via flash chromatography (95:5 to 5:95, Water : ACN) to yield **18** (33 mg, 46.9% yield). ^1H NMR (700 MHz, $CDCl_3$) δ 8.11 (s, 1H), 7.27 (m, 1H), 5.10 (s,

1H), 4.55 (t, $J = 4.6$ Hz, 2H), 3.84 (t, $J = 4.6$ Hz, 2H), 3.65 (m, 10H), 3.54 (t, $J = 5.1$ Hz, 2H), 3.31 (m, 2H), 2.85 (t, $J = 6.8$ Hz, 2H), 1.44 (s, 9H). ^{13}C NMR (176 MHz, $CDCl_3$) δ 156.00, 148.52, 140.47, 138.52, 137.08, 130.35, 114.20, 79.19, 70.62, 68.64, 67.06, 50.16, 40.36, 30.94, 28.43, 27.93. HRMS-ESI $[M+H]^+$ *m/z* calc for $[C_{19}H_{33}N_{6}0_{7}^{+}]$ 457.2405, found 457.2032.

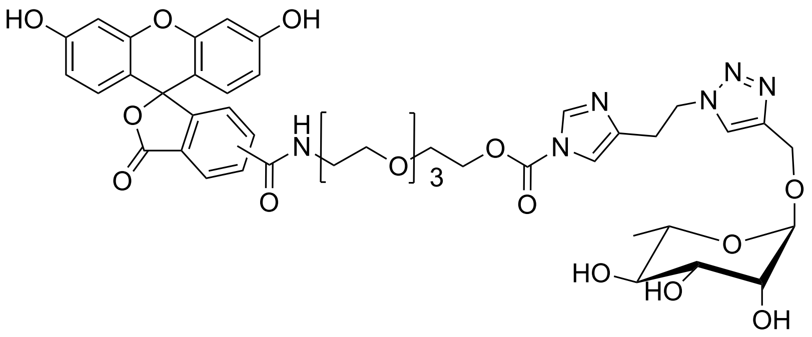

Fluor-O-cARM (6). **18** (8.3 mg, 0.02 mmol) was dried under vacuum, and resuspended Dioxane.HCl (2 mL, 4 M solution) under argon atmosphere. This was stirred for 2 hours, then crashed in cold ether. The resulting solution was centrifuged to pellet precipitate and decanted. The pellet was further dried under reduced pressure, then resuspended in anhydrous DMF (500 µL). To this solution, **16** (17.1 mg, 0.04 mmol) was added with TEA (3 µL, 0.02 mmol). The resulting solution was stirred for 3 hours, upon which more TEA was added (3 µL, 0.02 mmol) followed by 1 hour of additional stirring. The solution was precipitated in cold ether, and pellet collected by centrifugation. The supernatant was decanted, and pellet dried further under reduced pressure. To the pellet, **6** (11 mg, 0.05 mmol) was added with sodium ascorbate (8.9 mg, 0.05 mmol), $CuSO_4$ (2.3 mg, 0.01 mmol) and water (255 µL). Following 1 hour of stirring at room temperature, this solution was purified via HPLC and pH neutralized (pH ~7) prior to lyophilization to yield product (3.3 mg, 18% yield over 3 steps). HRMS-ESI $[M+H]^+$ *m/z* calc for $[C_{44}H_{49}N_{6}0_{16}^{+}]$ 917.3200, found 917.6854.

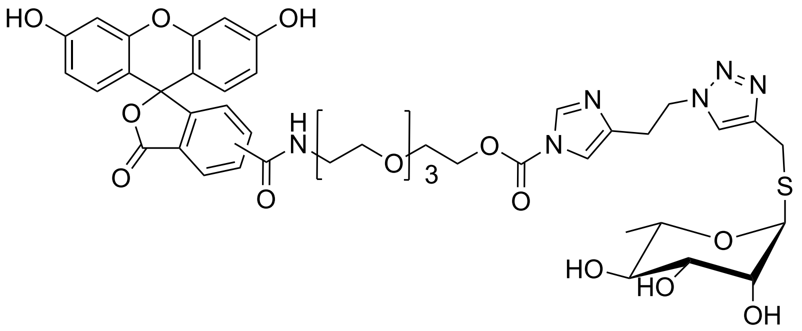

Fluor-S-cARM (7). **18** (8.3 mg, 0.02 mmol) was dried under vacuum, and resuspended Dioxane.HCl (2 mL, 4 M solution) under argon atmosphere. This was stirred for 2 hours, then crashed in cold ether. The resulting solution was centrifuged to pellet precipitate and decanted. The pellet was further dried under reduced pressure, then resuspended in anhydrous DMF (500 µL). To this solution, **16** (17.1 mg, 0.04 mmol) was added with TEA (3 µL, 0.02 mmol). The resulting solution was stirred for 3 hours, upon which more TEA was added (3 µL, 0.02 mmol) followed by 1 hour of additional stirring. The solution was precipitated in cold ether, and pellet collected by centrifugation. The supernatant was decanted, and pellet dried further under reduced pressure. To the pellet, **4** (11.8 mg, 0.05 mmol) was added with sodium ascorbate (8.9 mg, 0.05 mmol), $CuSO_4$ (2.3 mg, 0.01 mmol) and water (255 µL). Following 1 hour of stirring, this solution was purified via HPLC and pH neutralized (pH ~7) prior to lyophilization to yield product (0.84 mg, 4.5% yield over 3 steps). HRMS-ESI $[M+H]^+$ *m/z* calc for $[C_{44}H_{49}N_{6}0_{15}S^{+}]$ 933.2971, found 933.5457.

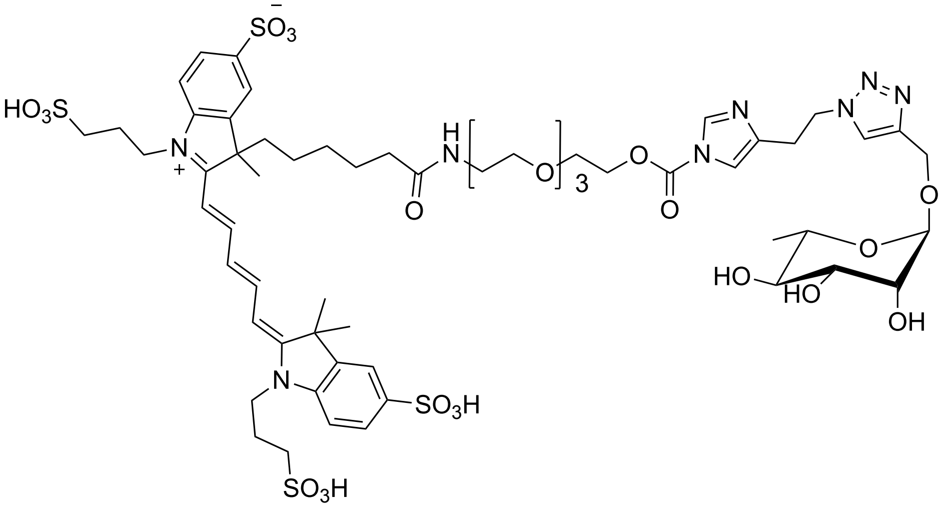

A647-cARM (8). **18** (3.6 mg, 0.008 mmol) was dried under vacuum, and resuspended in Dioxane.HCl (100 µL, 4 M solution) under argon atmosphere. This was stirred for 2 hours, then crashed in cold ether. The resulting solution was centrifuged to pellet precipitate and decanted. The pellet was resuspended in ACN (100 µL) and crashed again in cold ether followed by centrifugation. This solution was decanted, and the pellet was further dried under reduced pressure. The pellet was then resuspended in anhydrous DMF (100 µL). To this solution, Alexa 647-NHS (5 mg, 0.005 mmol) was added with DIPEA (4.6 µL, 0.026 mmol). The resulting solution was stirred for 2 hours. Directly to this solution, **6** (4.2 mg, 0.021 mmol) was added with sodium ascorbate (2.6 mg, 0.013 mmol), $CuSO_4$ (0.65 mg, 0.003 mmol) and water (100 µL). Following 30 minutes of stirring, this solution was purified via HPLC (10 mM, pH 4 ammonium formate buffer - 95:5 to 5:95 water:MeOH), and pH neutralized (pH ~7) prior to lyophilization to yield product (0.44 mg, 4% yield over 3 steps). HRMS-ESI $[M+2H]^{2+}$ *m/z* calc for $[C_{59}H_{84}N_8O_{23}S_4^{2+}]$ 700.2261, found 700.5951.

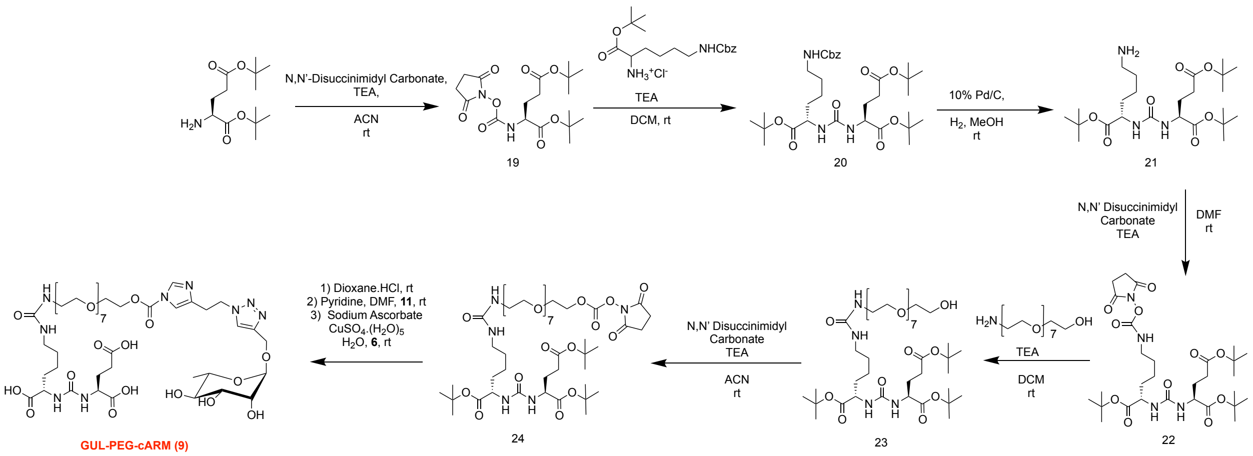

Scheme S3. Synthesis of **Rha-AI-PEG-GUL cARM (9)**

Note: Synthesis of compounds **19-24** adapted from previous work.[1,2]

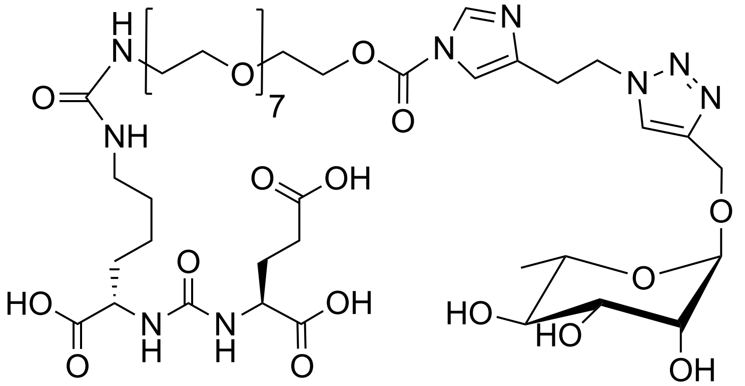

GUL-PEG-cARM (9). **24** (55 mg, 0.05 mmol) was dried under reduced pressure, then resuspended in Dioxane.HCl (1 mL, 4 M solution) and stirred for 2 hours. The solution was then crashed in cold ether and centrifuged to pellet precipitate. The supernatant was decanted, and the pellet was resuspended in ACN (1 mL) and crashed again in cold ether followed by centrifugation. This solution was decanted, and the pellet was further dried under reduced pressure. The pellet was then resuspended in anhydrous DMF (500 µL) followed by addition of **11** (9.6 mg, 0.07 mmol) and pyridine (31.1 µL, 0.39 mmol). Following 2 hours of stirring, directly to this solution **6** (39.1 mg, 0.19 mmol) was added with sodium ascorbate (47.9 mg, 0.24 mmol), $CuSO_4$ (12.1 mg, 0.05 mmol) and water (500 µL). This solution was stirred for 30 minutes followed by purification via HPLC, and pH neutralized (pH ~7) prior to lyophilization to yield product (5 mg, 8.7% yield). HRMS-ESI $[M+H]^+$ *m/z* calc for $[C_{44}H_{74}N_9O_{22}^+]$ 1080.4943, found 1080.7874.

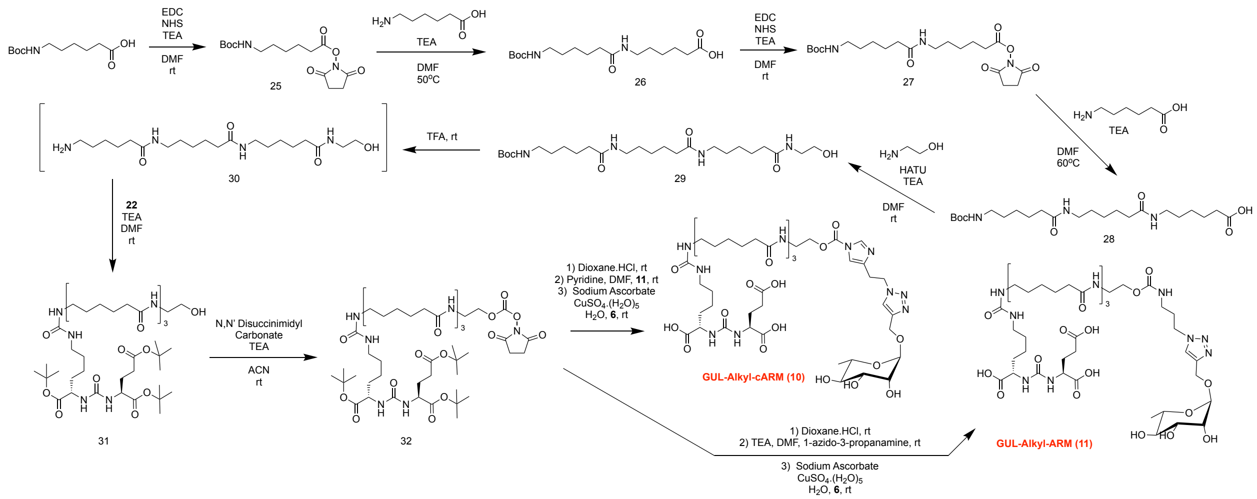

Scheme S4. Synthesis of compounds **Rha-Alkyl-GUL cARM/ARM (10-11)**

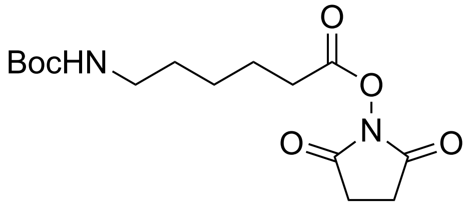

<u>BocNH-Hexanoic-NHS ester (25).</u> BocNH-Hexanoic acid (300 mg, 1.3 mmol) was dissolved in DMF (3 mL) followed by addition of NHS (179 mg, 1.6 mmol), TEA (200 µL, 1.4 mmol), and EDC.HCl (274 mg, 1.4 mmol). This solution was stirred overnight, followed by dilution with EtOAc and extraction with saturated sodium bicarbonate solution once. The organic layer was then washed 3X with brine. The organic layer was stirred over Na_2SO_4, the dried under reduced pressure to yield **25** (388.6 mg, 91% yield). ^{1}H NMR (400 MHz, $CDCl_3$) δ 4.58 (s, 1H), 3.12 (m, 2H), 2.83 (s, 4H), 2.61 (t, *J* = 7.4 Hz, 2H), 1.76 (m, 2H), 1.52 (m, 2H), 1.43 (m, 11H). ^{13}C NMR (101 MHz, $CDCl_3$) δ 169.35, 168.69, 156.19, 40.44, 31.07, 29.76, 28.63, 25.95, 24.47.

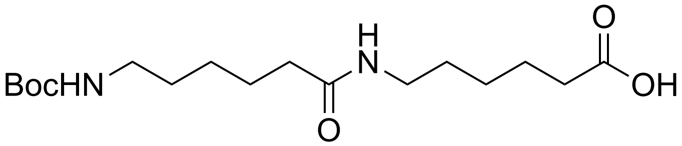

<u>BocNH-(Hexanoic)$_2$-Carboxylic Acid (26).</u> **25** (359.9 mg, 1.1 mmol) was dissolved in DMF (8 mL) with 6-aminohexanoic acid (228 mg, 1.7 mmol) and TEA (520 µL, 3.7 mmol). The solution was stirred at 50°C overnight then the solvent was evaporated under reduced pressure. The resulting oil was purified via flash chromatography (100:0 to 80:20, DCM:MeOH), to yield **26** (261.1 mg, 68.9% yield). ^{1}H NMR (700 MHz, $CDCl_3$) δ 5.96 (m, 1H), 5.57 (m, 1H), 3.29 (s, 2H), 3.12 (s, 2H), 2.36 (t, *J* = 6.7 Hz, 2H), 2.21 (t, *J* = 7.1 Hz, 2H), 1.64 (m, 4H), 1.51 (m, 4H), 1.44 (m, 9H), 1.36 (m, 4H). ^{13}C NMR (176 MHz, $CDCl_3$) δ 171.87, 38.80, 33.20, 28.31, 25.28, 23.58. HRMS-ESI $[M+H]^+$ *m/z* calc for $[C_{17}H_{33}N_20_5^+]$ 345.2384, found 345.1043.

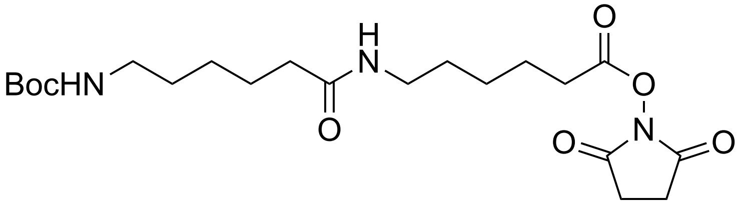

<u>BocNH-(Hexanoic)$_2$-NHS Ester (27)</u>. **26** (251 mg, 0.7 mmol) was dissolved in DMF (5 mL) with EDC.HCl (154 mg, 0.8 mmol), NHS (103 mg, 0.9 mmol), and TEA (112 µL, 0.8 mmol). The solution was stirred overnight, then diluted with EtOAc and extracted with 50% sat. sodium bicarbonate solution once. The organic layer was washed 3x with brine, then stirred with Na_2SO_4 and dried under reduced pressure for **27** (153.6 mg, 47.7% yield). ^{1}H NMR (700 MHz, $CDCl_3$) δ 5.69 (s, 1H), 4.55 (s, 1H), 3.27 (m, 2H), 3.11 (m, 2H), 2.85 (m, 4H), 2.63 (t, *J* = 7.1 Hz, 2H), 2.17 (t, *J* = 7.5 Hz, 2H), 1.78 (m, 2H), 1.65 (m, 2H), 1.55 (m, 2H), 1.44 (m, 13H), 1.34 (m, 2H). ^{13}C NMR (176 MHz, $CDCl_3$) δ 172.93, 169.23, 168.50, 38.92, 36.55, 30.89, 28.88, 28.44, 26.42, 25.74, 25.62, 25.34, 24.22. HRMS-ESI $[M+H]^+$ *m/z* calc for $[C_{21}H_{36}N_30_7^+]$ 442.2548, found 442.1275.

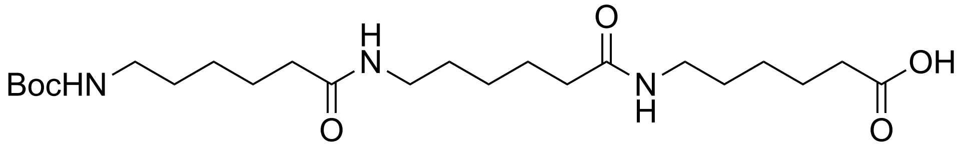

BocNH-(Hexanoic)$_3$-Carboxylic Acid (28). **27** (145 mg, 0.3 mmol) was dissolved in DMF (3 mL) with 6-aminohexanoic acid (70 mg, 0.5 mmol) and TEA (180 µL, 1.3 mmol). The solution was stirred overnight at 60°C, then solvent removed under reduced pressure. The resulting oil was purified via flash chromatography (100:0 to 80:20, DCM:MeOH), to yield **28** (143.5 mg, 95.6% yield). ^{1}H NMR (700 MHz, $CDCl_3$) δ 6.06 (s, 1H), 5.96 (s, 1H), 4.67 (s, 1H), 3.28 (m, 4H), 3.10 (m, 2H), 2.35 (t, J = 6.6 Hz, 2H), 2.19 (m, 4H), 1.65 (m, 6H), 1.52 (m, 4H), 1.48 (m, 2H), 1.43 (s, 9H), 1.39 (m, 2H), 1.34 (m, 4H). HRMS-ESI $[M+H]^+$ m/z calc for $[C_{23}H_{44}N_3O_6^+]$ 458.3225, found 458.3107.

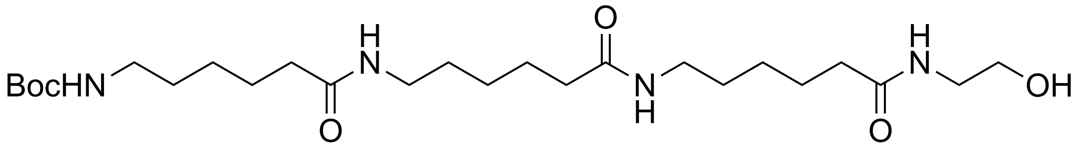

BocNH-(Hexanoic)$_3$-OH (29). **28** (28 mg, 0.06 mmol) was dissolved in DMF (500 µL) with TEA (180 µL, 1.3 mmol), and HATU (28 mg, 0.7 mmol). After 20 minutes 2-aminoethanol (5.6 mg, 0.09 mmol) was added, and the solution was stirred overnight. The solution was purified via HPLC to yield **29** (7.6 mg, 24.8% yield). ^{1}H NMR (700 MHz, MeOD) δ 3.58 (t, J = 5.8 Hz, 2H), 3.29 (t, J = 5.8 Hz, 2H), 3.16 (m, 4H), 3.02 (t, J = 7.1 Hz, 2H), 2.21 (t, J = 7.5 Hz, 2H), 2.17 (m, 4H), 1.62 (m, 6H), 1.49 (m, 6H), 1.43 (s, 9H), 1.34 (m, 6H). ^{13}C NMR (176 MHz, MeOD) δ 176.33, 175.94, 158.49, 79.73, 61.56, 42.87, 41.13, 40.12, 36.86, 30.60, 30.06, 28.72, 27.49, 27.48, 26.65, 26.53. HRMS-ESI $[M+H]^+$ m/z calc for $[C_{25}H_{49}N_4O_6^+]$ 501.3647, found 501.3138.

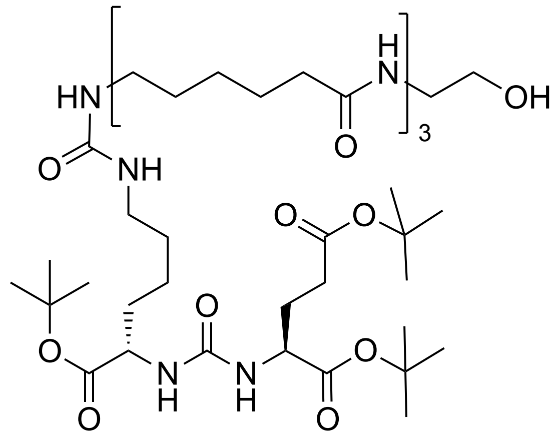

Tri-t-butyl-GUL-Alkyl-OH (31). **29** (15.5 mg, 0.03 mmol) was resuspended in TFA (1 mL) and stirred for 2 hours. The solvent was then removed under air stream. The resulting oil was resuspended in DCM (4 mL) and subsequently dried under air stream 3X. The resulting oil was resuspended in DMF (500 µL) with TEA (13 µL, 0.09 mmol) and added to **22** (10 mg, 0.02 mmol). This solution was stirred for 2 hours, then purified via HPLC to yield **31** (2 mg, 14.6% yield). ^{1}H NMR (700 MHz, $CDCl_3$) δ 6.79 (s, 1H), 6.39 (m, 1H), 6.18 (m, 1H), 5.96 (m, 1H), 5.92 (m, 1H), 5.29 (s, 1H), 5.11 (s, 1H), 4.31 (m, 1H), 4.27 (m, 1H), 4.04 (s, 1H), 3.70 (t, J = 4.6 Hz, 2H), 3.41 (m, 2H), 3.26 (m, 6H), 3.09 (m, 2H), 2.34 (m, 2H), 2.20 (m, 6H),

1.95 (m, 2H), 1.71 (m, 2H), 1.65 (m, 6H), 1.50 (m, 2H), 1.45 (m, 33H), 1.33 (m, 8H). ^{13}C NMR (176 MHz, $CDCl_3$) δ 174.37, 173.52, 172.46, 159.30, 157.82, 82.68, 81.83, 80.98, 62.30, 53.17, 42.56, 40.01, 39.17, 36.62, 31.74, 29.83, 28.98, 28.13, 26.43, 25.37, 21.76. HRMS-ESI $[M+H]^+$ *m/z* calc for $[C_{45}H_{84}N_7O_{12}^+]$ 914.6172, found 914.7940.

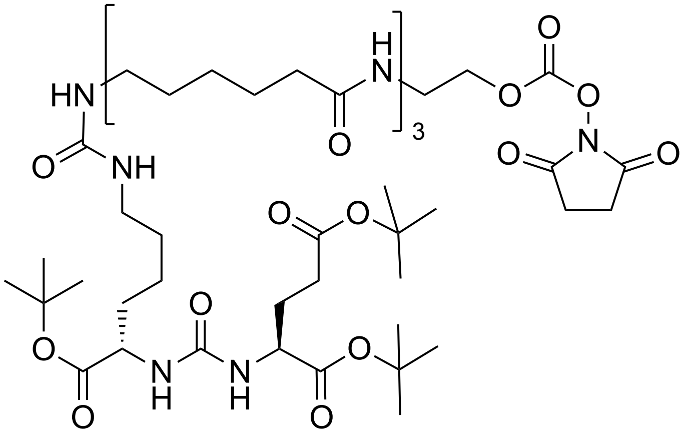

Tri-t-butyl-GUL-Alkyl-NHS (32). **31** (2 mg, 0.002 mmol) was dissolved in anhydrous ACN (100 µL). To this solution, N,N'-disuccinimidyl carbonate (2.2 mg, 0.009 mmol) was added followed by TEA (0.4 µL, 0.003mmol). This solution was stirred for 3 hours, then purified via HPLC to yield **32** (1.4 mg , 60% yield). ^{1}H NMR (700 MHz, $CDCl_3$) δ 6.67 (s, 1H), 6.28 (s, 1H), 6.06 (m, 1H), 5.85 (m, 2H), 5.18 (s, 1H), 4.99 (s, 1H), 4.43 (m, 2H), 4.32 (m, 1H), 4.27 (m, 1H), 3.60 (m, 2H), 3.26 (m, 6H), 3.10 (m, 2H), 2.87 (s, 4H), 2.34 (t, J = 8.5 Hz, 2H), 2.21 (m, 6H), 1.97 (m, 2H), 1.67 (m, 2H), 1.64 (m, 6H), 1.45 (m, 35H), 1.31 (m, 8H). HRMS-ESI $[M+H]^+$ *m/z* calc for $[C_{50}H_{87}N_8O_{16}^+]$ 1055.6235, found 1055.8965.

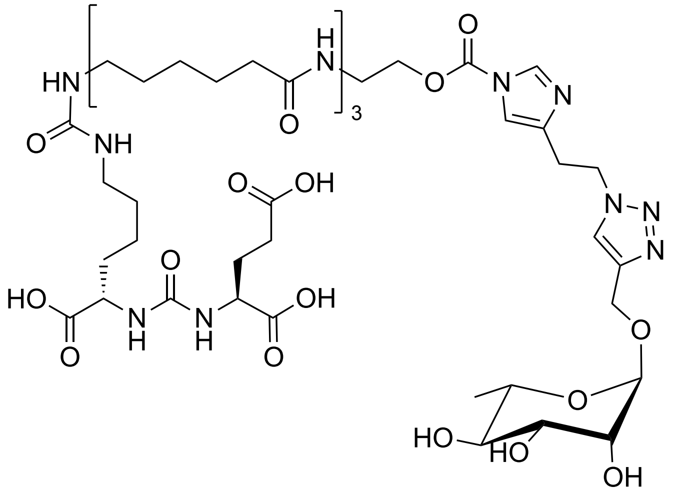

GUL-Alkyl-cARM (10). **32** (10.8 mg, 0.01 mmol) was dried under reduced pressure, then resuspended in Dioxane.HCl (2 mL, 4 M solution) in an argon atmosphere. This was stirred for 2 hours, then solvent removed under reduced pressure. The crude oil was resuspended in anhydrous DMF (300 µL) with pyridine (30 µL, 0.37 mmol) and **11** (2.1 mg, 0.02 mmol). After 4 hours, directly to this solution **6** (8.1 mg, 0.04 mmol) was added with sodium ascorbate (10 mg, 0.05 mmol), $CuSO_4$ (2.5 mg, 0.01 mmol), and water (100 µL). After 20 minutes, this solution was purified via HPLC and pH neutralized (pH ~7) prior to lyophilization to yield product (1.7 mg, 15.6% yield over 3 steps). HRMS-ESI $[M+H]^+$ *m/z* calc for $[C_{48}H_{79}N_{12}O_{18}^+]$ 1111.5630, found 111.8328.

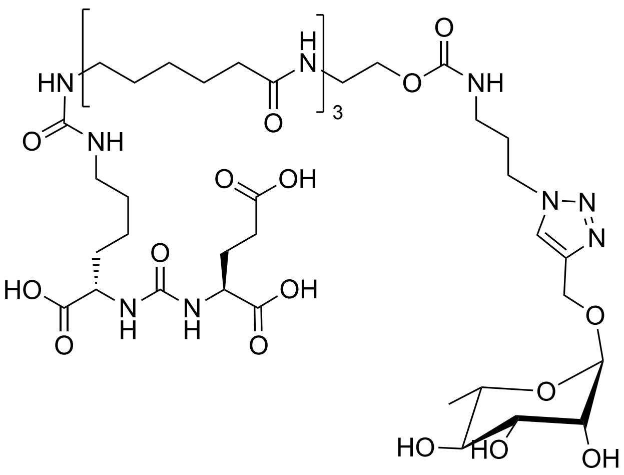

GUL-Alkyl-ARM (11). **32** (12.2 mg, 0.01 mmol) was dried under reduced pressure, then resuspended in Dioxane.HCl (2 mL, 4 M solution) in an argon atmosphere. This was stirred for 2 hours, then solvent removed under reduced pressure. The crude oil was resuspended in anhydrous DMF (400 µL) with TEA (50 µL, 0.36 mmol) and 1-azido-3-propanamine (2.3 mg, 0.02 mmol). After 2 hours, directly to this solution **6** (18.7 mg, 0.09 mmol) was added with sodium ascorbate (22.9 mg, 0.12 mmol), $CuSO_4$ (5.8 mg, 0.02 mmol), and water (200 µL). After 30 minutes, this solution was purified via HPLC and pH neutralized (pH ~7) prior to lyophilization to yield product (3.6 mg, 29.5% yield over 3 steps). ^{1}H NMR (700 MHz, D_2O_salt) δ 8.09 (s, 1H), 4.92 (s, 1H), 4.79 (m, 2H), 4.51 (t, J = 6.9 Hz, 2H), 4.12 (m, 2H), 4.01 (m, 2H), 3.95 (m, 1H), 3.75 (m, 1H), 3.66 (m, 1H), 3.46 (m, 3H), 3.13 (m, 10H), 2.24 (m, 8H), 2.14 (m, 2H), 1.93 (m, 2H), 1.71 (m, 2H), 1.61 (m, 6H), 1.49 (m, 8H), 1.37 (m, 2H), 1.31 (m, 6H), 1.26 (d, J = 6.2 Hz, 3H). ^{13}C NMR (176 MHz, D_2O_salt) δ 182.57, 180.76, 180.23, 176.79, 160.72, 159.23, 143.68, 125.09, 99.69, 71.99, 70.08, 68.78, 63.54, 60.07, 55.57, 47.89, 39.69, 39.05, 35.69, 34.15, 32.14, 29.43, 28.92, 27.97, 25.41, 22.41, 16.59. HRMS-ESI $[M+H]^+$ m/z calc for $[C_{46}H_{80}N_{11}0_{18}^{+}]$ 1074.5677, found 1074.6058.

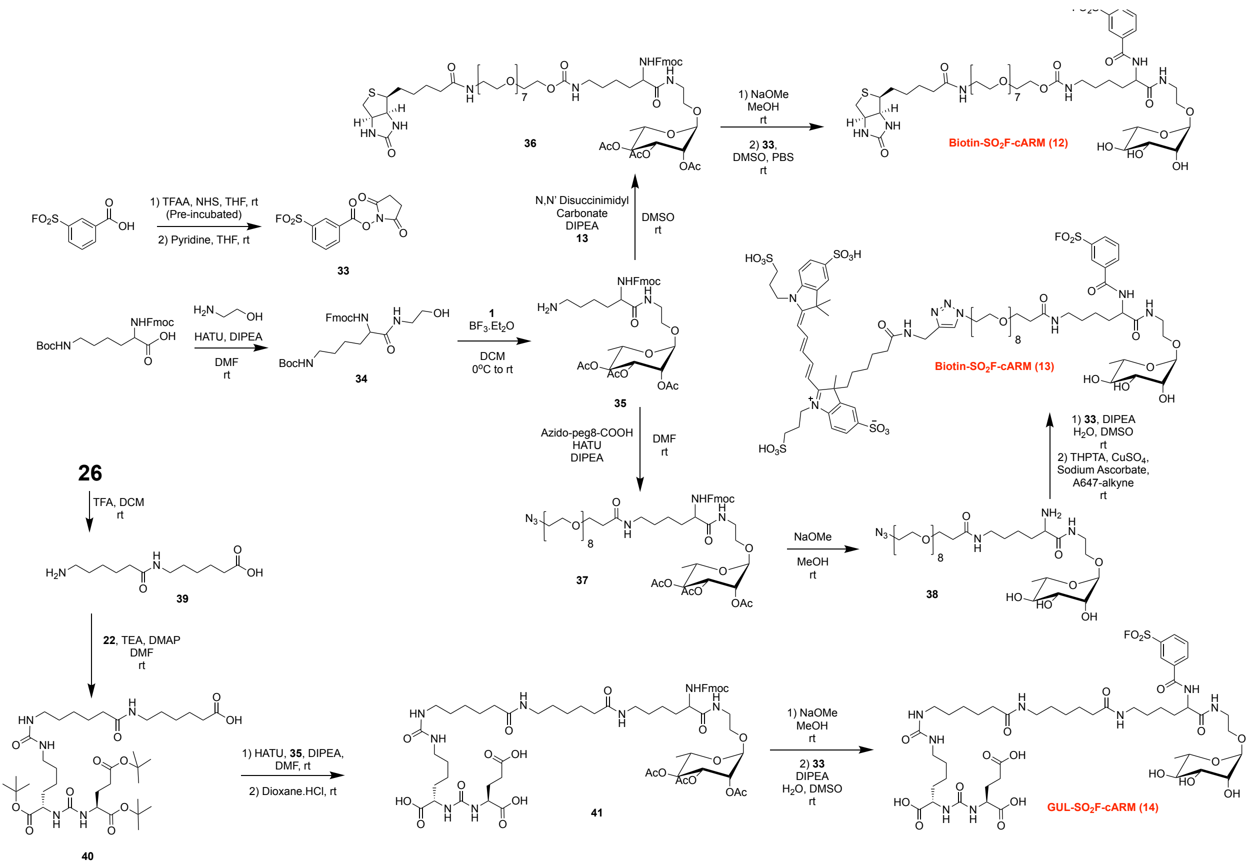

Scheme S5. Synthesis of SO_2F compound series **(12-14)**

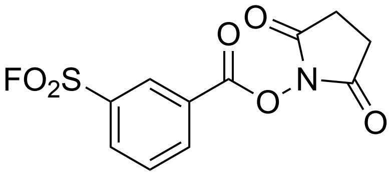

__**Aryl-SO_2F-NHS (33)**.__ To a flame dried flask set with an anhydrous argon atmosphere, NHS (155 mg, 1.35 mmol) was dissolved in THF (1 mL). This solution was placed in a room temperature water bath, followed by dropwise addition of TFAA (375 µL, 2.70 mmol). This solution was stirred for 90 minutes, followed by addition of toluene (1 mL) and solvent removal under reduced pressure. The resulting oil was resuspended in toluene (1 mL) and dried under reduced pressure while retaining inert argon atmosphere. To the resulting white powder, anhydrous THF (1 mL) was added with 3-sulfonylfluoride benzoic acid (50 mg, 0.25 mmol) and pyridine (24 µL, 0.30 mmol). This solution was stirred for 150 minutes followed by purification via RP flash chromatography to yield **33** (55 mg, 60.9% yield). ^{1}H NMR (700 MHz, DMSO) δ 8.62 (m, 3H), 8.08 (m, 1H), 2.92 (s, 4H). ^{13}C NMR (176 MHz, DMSO) δ 169.89, 159.99, 137.36, 134.63, 132.90, 132.75, 132.16, 129.22, 126.30, 25.46.

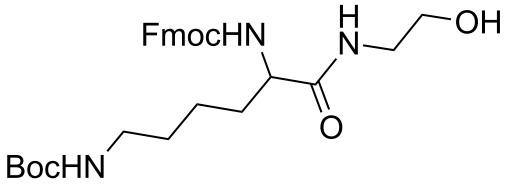

ε-NHBoc-α-Fmoc-Lysine-Ethanol (34). ε-NHBoc-α-Fmoc-Lysine (110 mg, 0.24 mmol) was dissolved in DMF (2 mL) with DIPEA (90 µL, 0.517 mmol) and HATU (93 mg, 0.245 mmol) for 15 minutes, followed by addition of ethanolamine (16 µL, 0.26 mmol). The solution as stirred for 3 hours followed by purification via RP flash chromatography to yield **34** (81.7 mg, 68.0% yield). ^{1}H NMR (700 MHz, $CDCl_3$) δ 7.73 (m, 2H), 7.56 (m, 2H), 7.37 (m, 2H), 7.28 (m, 2H), 7.05 (s, 1H), 5.95 (s, 1H), 4.82 (s, 1H), 4.36 (m, 2H), 4.17 (t, J = 6.8 Hz, 1H), 4.12 (m, 1H), 3.64 (m, 2H), 3.36 (m, 2H), 3.06 (m, 2H), 1.72 (m, 2H), 1.46 (m, 2H), 1.41 (s, 9H), 1.34 (m, 2H). ^{13}C NMR (176 MHz, $CDCl_3$) δ 172.71, 156.48, 143.79, 141.28, 127.74, 127.09, 125.05, 119.98, 79.41, 67.00, 61.41, 54.87, 47.11, 42.11, 39.81, 32.12, 29.50, 28.41, 22.47.

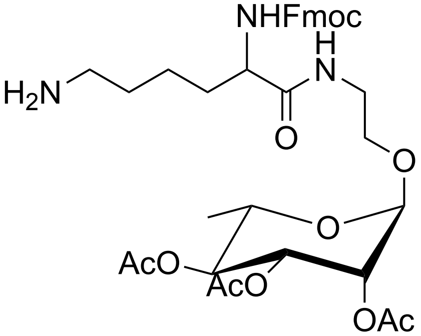

Peracetyl-α-O-Rhamnose-Lysine-α-Fmoc-ε-NH$_2$ (35). **1** (210 mg, 0.63 mmol) and **34** (323 mg, 0.63 mmol) was dissolved in a DCM/toluene co-solvent and dried under reduced pressure (3x). The resultant residue was resuspended in anhydrous DCM (1.5 mL) under an argon atmosphere and placed in an ice bath. After 15 minutes, $BF_3.Et_2O$ (448 µL, 3.63 mmol) was added dropwise. The solution was allowed to stir, slowly warming to room temperature for 16 hours. Directly to this solution, a saturated sodium bicarbonate solution was added to quench the reaction. The solution was stirred for 10 minutes, or until bubbling subsided. The solution was diluted with EtOAc and washed 2x with saturated sodium bicarbonate, and 2x with brine. The organic layer was dried over Na_2SO_4 and concentrated under reduced pressure. The resulting crude oil was purified via flash chromatography to yield **35** (75.9 mg, 17.6% yield). ^{1}H NMR (700 MHz, MeOD) δ 7.80 (m, 2H), 7.67 (m, 2H), 7.40 (m, 2H), 7.32 (m, 2H), 5.25 (m, 1H), 5.19 (dd, J = 10.2, 3.4 Hz, 1H), 4.99 (dd, J = 10.0, 10.0 Hz, 1H), 4.77 (m, 1H), 4.41 (d, J = 7.1 Hz, 2H), 4.23 (m, 1H), 4.06 (m, 1H), 3.90 (m, 1H), 3.66 (m, 2H), 3.43 (m, 2H), 2.91 (m, 2H), 2.09 (s, 3H), 1.99 (s, 3H), 1.93 (s, 3H), 1.76 (m, 2H), 1.66 (m, 2H), 1.46 (m, 2H), 1.16 (d, J = 6.2 Hz, 3H). ^{13}C NMR (176 MHz, MeOD) δ 173.55, 170.33, 157.04, 144.00, 143.76, 141.23, 127.43, 127.42, 126.76, 124.76, 119.58, 97.44, 70.65, 69.46, 66.35, 65.88, 54.91, 48.45, 42.75, 39.17, 38.71, 31.18, 26.84, 23.85, 22.46, 19.25, 16.36. HRMS-ESI $[M+H]^+$ *m/z* calc for $[C_{35}H_{46}N_{3}0_{11}^{+}]$ 684.3127, found 684.6744.

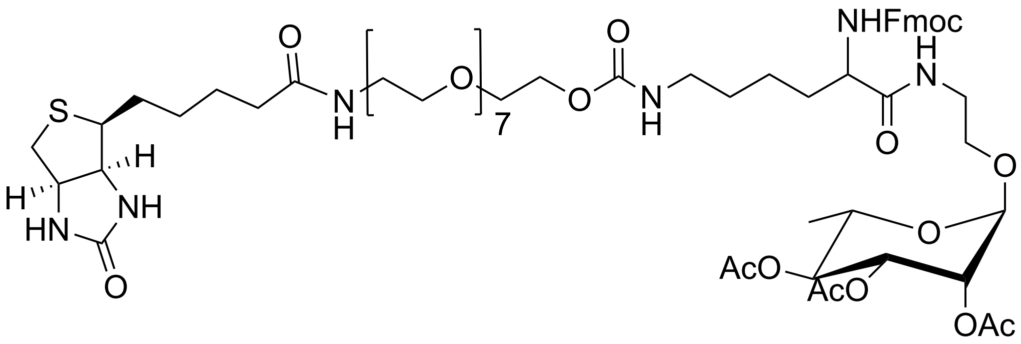

Peracetyl-α-O-Rhamnose-Lysine-α-Fmoc-ε-Peg8-Biotin (36). **13** (36.6 mg, 0.06 mmol) was dissolved in anhydrous DMSO (0.5 mL) with N,N'-disuccinimidyl carbonate (17.3 mg, 0.07 mmol) and DIPEA (9.5 µL, 0.05 mmol). This solution was stirred for 3 hours, then crashed in cold ether. The supernatant was removed, and the reaction mixture was crashed in cold ether again. The supernatant was removed, and the remaining ether was removed under reduced pressure. To the crude solution, DMSO (600 µL) was added with **35** (21 mg, 0.03 mmol) and DIPEA (10 µL, 0.06 mmol). This solution was stirred for 3 hours, then purified via HPLC (10 mM, pH 4 ammonium formate buffer - 95:5 to 5:95 water:MeOH) to yield **36** (9.5 mg, 23.7% yield). ^{1}H NMR (700 MHz, DMSO) δ 8.14 (t, *J* = 5.5 Hz, 1H), 7.90 (m, 2H), 7.85 (t, *J* = 5.6 Hz, 1H), 7.74 (m, 2H), 7.48 (m, 1H), 7.42 (m, 2H), 7.33 (m, 2H), 7.20 (t, *J* = 5.6 Hz, 1H), 6.42 (s, 1H), 6.37 (s, 1H), 5.12 (m, 2H), 4.88 (m, 1H), 4.79 (m, 1H), 4.29 (m, 1H), 4.22 (m, 3H), 4.13 (m, 1H), 4.03 (t, *J* = 4.6 Hz, 2H), 3.94 (m, 1H), 3.84 (m, 1H), 3.61 (m, 1H), 3.54 (t, *J* = 4.6 Hz, 2H), 3.51 (m, 24H), 3.47 (m, 1H), 3.39 (m, 3H), 3.18 (m, 3H), 3.10 (m, 1H), 2.95 (m, 2H), 2.70 (m, 2H), 2.09 (s, 3H), 2.06 (m, 2H), 2.02 (s, 3H), 1.92 (s, 3H), 1.54 (m, 2H), 1.53 (m, 2H), 1.50 (m, 2H), 1.37 (m, 2H), 1.31 (m, 2H), 1.25 (m, 2H), 1.10 (d, *J* = 6.2 Hz, 3H). ^{13}C NMR (176 MHz, DMSO) δ 172.73, 170.16, 170.05, 165.89, 163.37, 163.17, 156.58, 156.43, 144.39, 144.26, 141.17, 129.40, 128.10, 127.76, 127.52, 125.82, 121.86, 120.57, 120.51, 97.24, 70.57, 70.24, 70.18, 70.03, 69.63, 69.40, 69.35, 69.14, 66.55, 66.19, 66.05, 63.46, 61.50, 59.66, 55.89, 55.11, 47.13, 38.91, 35.55, 32.16, 29.61, 28.66, 28.50, 25.73, 23.42, 21.05, 20.92, 20.89, 17.69. HRMS-ESI $[M+H]^+$ *m/z* calc for $[C_{62}H_{93}N_{6}0_{22}S^{+}]$ 1305.6058, found 1305.9709.

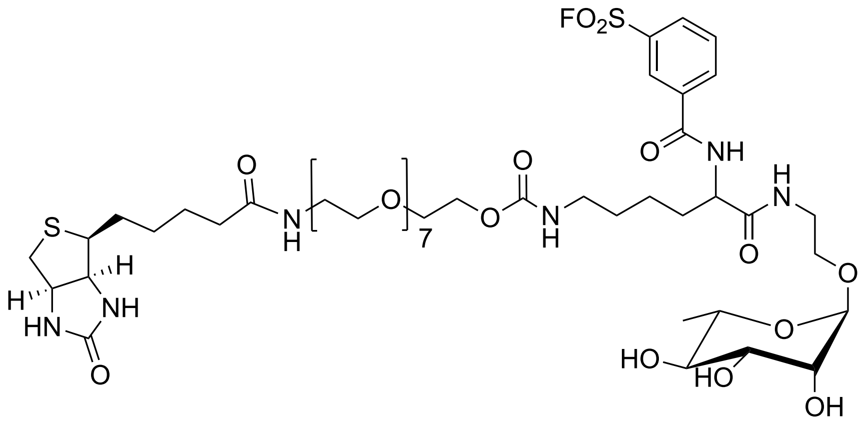

Biotin-SO$_2$F-cARM (12). **36** (7.5 mg, 0.006 mmol) was resuspended in MeOH (0.4 mL) followed by addition of sodium methoxide solution (20 µL, 25 wt% in MeOH). The solution was stirred for 20 minutes then diluted with 10 mL water and quenched to pH 7 with a 0.1 M HCl solution. The resulting solution was run through a celite plug, and the eluent was lyophilized to yield crude deprotected product. This crude deprotected product was directly resuspended in DMSO (400 µL) and PBS (200 µL, pH 7.4) with **33** (6.9 mg, 0.02 mmol) and stirred for 20 minutes. The solution was purified on HPLC to yield product (<1 mg,

1% yield over two steps, concentration determined via standard curve). HRMS-ESI $[M+H]^+$ *m/z* calc for $[C_{48}H_{80}FN_{6}0_{20}S_{2}^{+}]$ 1143.4847, found 1143.7416.

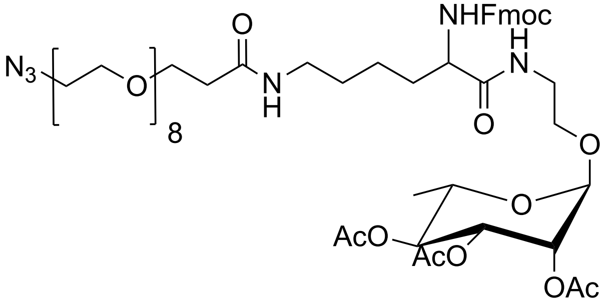

Peracetyl-α-O-Rhamnose-Lysine-α-Fmoc-ε-Peg8-N_3 (37). Azido-Peg8-COOH (32 mg, 0.07 mmol) was dissolved in DMF (500 μL) with HATU (23.7 mg, 0.06 mmol) and DIPEA (19.1 μL, 0.11 mmoL). The resulting solution was stirred for 15 minutes followed by addition of **35** (30 mg, 0.04 mmol). The reaction mixture was stirred overnight, followed by purification via reverse phase flash chromatography to yield **37** (26.8 mg, 53.9% yield). ^{1}H NMR (700 MHz, MeOD) δ 7.87 (s, 1H), 7.81 (m, 2H), 7.68 (m, 2H), 7.40 (m, 2H), 7.32 (m, 2H), 5.25 (m, 1H), 5.21 (m, 1H), 4.99 (m, 1H), 4.77 (s, 1H), 4.40 (m, 2H), 4.23 (t, *J* = 6.8 Hz, 1H), 4.04 (m, 1H), 3.90 (m, 1H), 3.77 (m, 1H), 3.70 (t, *J* = 6.1 Hz, 2H), 3.65 (m, 2H), 3.61 (m, 28H), 3.54 (m, 1H), 3.43 (t, *J* = 5.3 Hz, 2H), 3.36 (t, *J* = 4.9 Hz, 2H), 3.19 (m, 2H), 2.41 (t, *J* = 6.1 Hz, 2H), 2.09 (s, 3H), 1.99 (s, 3H), 1.93 (s, 3H), 1.72 (m, 2H), 1.53 (m, 2H), 1.41 (m, 2H), 1.17 (d, *J* = 6.2 Hz, 3H). ^{13}C NMR (176 MHz, MeOD) δ 173.84, 172.52, 170.20, 157.02, 143.82, 141.22, 127.44, 126.81, 124.83, 119.58, 97.59, 70.70, 70.23, 70.15, 70.03, 69.90, 69.73, 69.50, 69.40, 66.91, 66.44, 66.33, 66.10, 55.18, 50.38, 38.82, 36.35, 31.45, 28.57, 22.90, 19.29, 16.38. HRMS-ESI $[M+H]^+$ *m/z* calc for $[C_{54}H_{81}N_{6}0_{20}^{+}]$ 1133.5500, found 1133.2056.

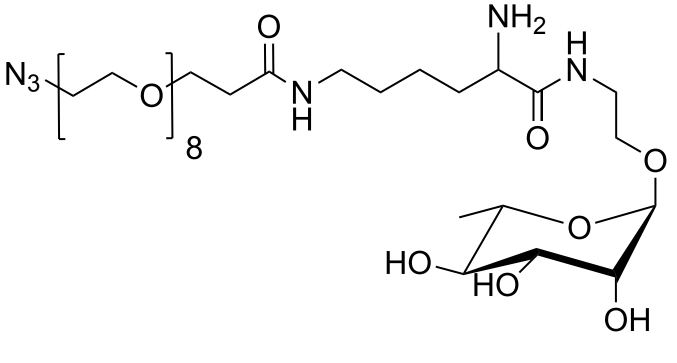

Peracetyl-α-O-Rhamnose-Lysine-α-NH_2-ε-Peg8-N_3 (38). **37** (12.8 mg, 0.01 mmol) was resuspended in MeOH (0.3 mL) followed by addition of sodium methoxide solution (30 μL, 25 wt% in MeOH). The solution was stirred for 20 minutes then diluted with 10 mL water and quenched to pH 7 with a 0.1 M formic acid solution. The resulting solution was run through a celite plug, and the eluent was lyophilized to yield **38** with a benign methyl acetate impurity in quantitative yields (100%). ^{1}H NMR (700 MHz, D_2O) δ 4.82 (s, 1H), 3.96 (m, 1H), 3.80 (m, 4H), 3.73 (m, 31H), 3.62 (m, 1H), 3.54 (t, *J* = 4.9 Hz, 2H), 3.47 (m, 3H), 3.39 (t, 1H), 3.22 (t, 2H), 2.54 (t, *J* = 6.1 Hz, 2H), 1.65 (m, 2H), 1.56 (m, 2H), 1.38 (m, 2H), 1.31 (d, *J* = 6.3 Hz, 3H). ^{13}C NMR (176 MHz, D_2O) δ 173.84, 99.69, 72.01, 69.60, 69.49, 69.24, 68.68, 66.85, 65.87, 54.55, 50.18, 39.12, 38.84, 36.13, 33.98, 28.15, 23.30, 22.27, 16.67. HRMS-ESI $[M+H]^+$ *m/z* calc for $[C_{33}H_{65}N_{6}0_{15}^{+}]$ 785.4502, found 785.3089.

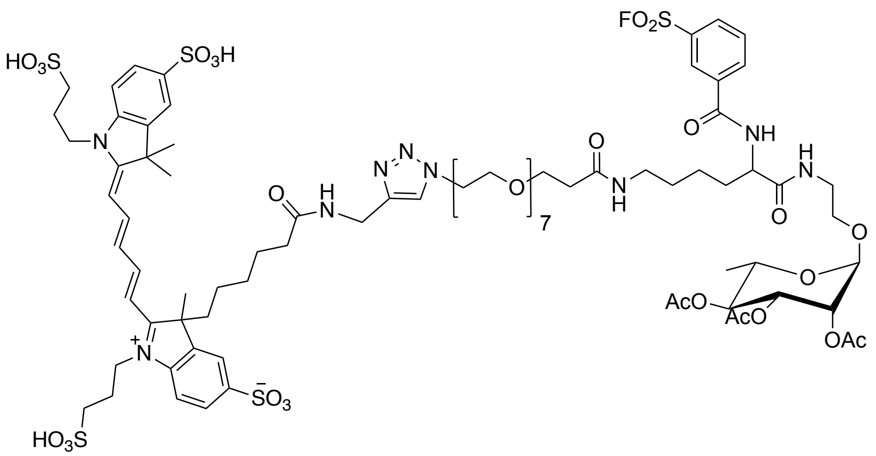

A647-SO_2F-cARM (13). **38** (3.4 mg, 0.004 mmol) was dissolve in water (400 µL). To this solution, DIPEA (1.5 µL, 0.009 mmol) was added followed by **33** (5.2 mg, 0.017 mmol, 55 µL in DMSO stock) dropwise. This solution was stirred for 20 minutes. Excess unreactive **33** was removed from solution via centrifugation. To the clear supernatant a pre-mixed copper click solution was added composed of THPTA (5.7 mg, 0.011 mmol), $CuSO_4$ (0.54 mg, 0.002 mmol), sodium ascorbate (4.26 mg, 0.022 mmol), and A647-alkyne (2.5 mg, 0.003 mmol) in water (130 µL). This solution was stirred for 1 hour followed by purification via HPLC (10 mM, pH 4 ammonium formate buffer - 95:5 to 5:95 water:MeOH) to yield product (<1 mg, 0.8% yield over two steps, concentration determined via standard curve). HRMS-ESI $[M+H+NH_4]^{2+}$ *m/z* calc for $[C_{79}H_{116}FN_90_{31}S_5^{2+}]$ 942.3391, found 942.3573.

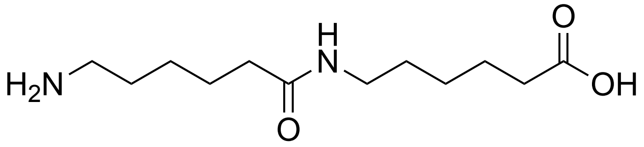

NH_2-(Hexanoic)$_2$-Carboxylic Acid (39). **26** (339 mg, 0.98 mmol) was dissolved in TFA:DCM (3 mL, 50:50) and stirred for 3 hours. The solvent was removed under air stream. The resulting oil was resuspended in DCM and dried under reduced pressure 3x to yield **39** in quantitative yields (100% yield). ^{1}H NMR (700 MHz, MeOD) δ 3.17 (t, *J* = 7.0 Hz, 2H), 2.92 (t, *J* = 7.6 Hz, 2H), 2.29 (t, *J* = 7.4 Hz, 2H), 2.21 (t, *J* = 7.4 Hz, 2H), 1.64 (m, 6H), 1.52 (m, 2H), 1.39 (m, 4H). ^{13}C NMR (176 MHz, MeOD) δ 177.50, 175.75, 174.90, 40.53, 36.61, 34.79, 30.11, 28.29, 27.50, 26.94, 26.29, 25.71.

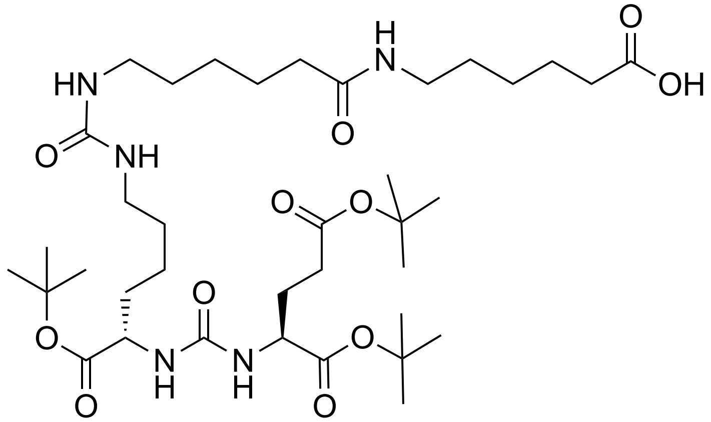

Tri-t-butyl-GUL-Alkyl-COOH (40). **22** (100 mg, 0.16 mmol) was dissolved in DMF (1.8 mL) with TEA (370 µL, 2.65 mmol). To this solution, **39** (176.6 mg, 0.73 mmol) was added with DMAP (20 mg, 0.016 mmol). This solution was stirred overnight, followed by dilution with EtOAc. The organic layer was washed 3X with citric acid (10% solution) and 3X with saturated brine. The organic layer was dried over Na_2SO_4 and the solvent was removed under reduced pressure. The resulting crude oil was purified via reverse phase flash chromatography to yield **40** (47.4 mg, 39.1 % yield). ^{1}H NMR (700 MHz, MeOD) δ 7.94 (s, 1H), 4.19 (m, 1H), 4.12 (m, 1H), 3.16 (t, J = 7.0 Hz, 2H), 3.10 (m, 4H), 2.31 (m, 4H), 2.18 (t, J = 7.5 Hz, 2H), 1.93 (m, 2H), 1.71 (m, 2H), 1.62 (m, 4H), 1.46 (m, 33H), 1.38 (m, 6H). ^{13}C NMR (176 MHz, MeOD) δ 177.68, 176.11, 174.03, 173.82, 173.56, 161.34, 160.03, 82.87, 82.61, 81.81, 54.94, 54.23, 40.78, 40.23, 37.09, 34.99, 33.30, 32.55, 31.10, 30.17, 29.09, 28.41, 28.37, 28.35, 27.58, 26.85, 25.84, 23.93. HRMS-ESI $[M+H]^+$ *m/z* calc for $[C_{37}H_{68}N_5O_{11}^{+}]$ 758.4910, found 758.4572.

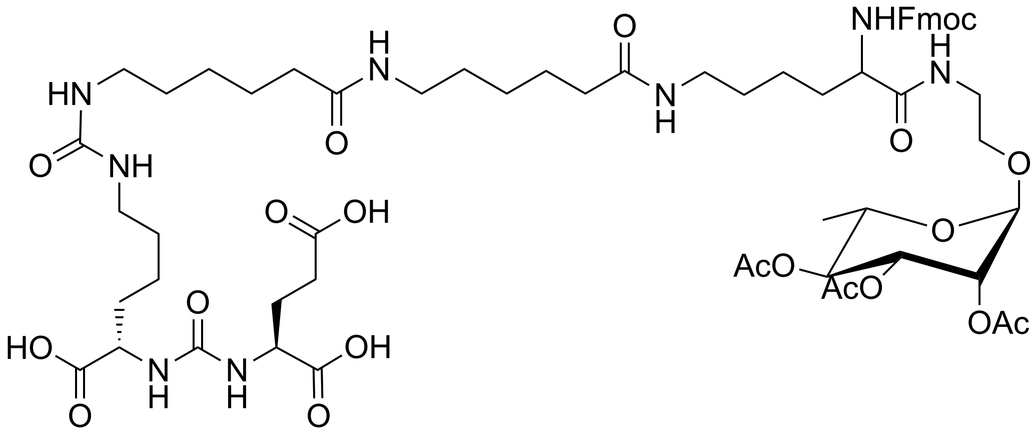

GUL-Alkyl-NHFmoc-α-O-Rhamnose (41). **40** (12.7 mg, 0.02 mmol) was dissolved in DMF (500 µL) with HATU (6.5 mg, 0.02 mmol) and DIPEA (8.7 µL, 0.05 mmol) and stirred for 15 minutes. Directly to this solution, **35** (13.9 mg, 0.02 mmol) was added. The resulting solution was stirred 16 hours, then diluted in EtOAc and washed 3X with each of saturated bicarb and saturated brine. The organic layer was dried over Na_2SO_4 and solvent removed under reduced pressure for crude product. The crude oil was resuspended in Dioxane.HCl (2 mL, 4 M solution) under argon atmosphere and stirred for 3 hours. The solution was then crashed in cold ether and pelleted via centrifugation. The crude pellet was purified via HPLC to yield **41** (3.2 mg, 15% yield over 2 steps). ^{1}H NMR (700 MHz, MeOD) δ 7.80 (m, 2H), 7.68 (m, 2H), 7.40

(m, 2H), 7.32 (m, 2H), 5.25 (m, 1H), 5.21 (m, 1H), 4.99 (m, 1H), 4.77 (s, 1H), 4.38 (m, 2H), 4.23 (m, 3H), 4.05 (m, 1H), 3.90 (m, 1H), 3.65 (m, 2H), 3.43 (m, 2H), 3.15 (m, 4H), 3.10 (m, 4H), 2.39 (m, 2H), 2.16 (t, *J* = 7.5 Hz, 4H), 2.09 (s, 3H), 2.01 (m, 2H), 1.99 (s, 3H), 1.93 (s, 3H), 1.74 (m, 2H), 1.71 (m, 2H), 1.60 (m, 4H), 1.42 (m, *J* = 7.7 Hz, 16H), 1.16 (d, *J* = 6.2 Hz, 3H). ^{13}C NMR (176 MHz, MeOD) δ 176.05, 171.61, 161.33, 160.10, 145.42, 145.19, 142.63, 128.82, 128.18, 126.24, 120.95, 98.99, 72.11, 70.91, 70.81, 67.72, 56.58, 54.78, 54.67, 48.99, 40.83, 40.18, 40.07, 37.03, 33.69, 32.88, 31.75, 31.02, 30.80, 30.12, 30.02, 29.71, 27.57, 27.47, 26.80, 26.71, 24.38, 23.87, 20.67, 17.76. HRMS-ESI $[M+H]^+$ *m/z* calc for $[C_{60}H_{87}N_8O_{21}^+]$ 1255.5980, found 1256.5691.

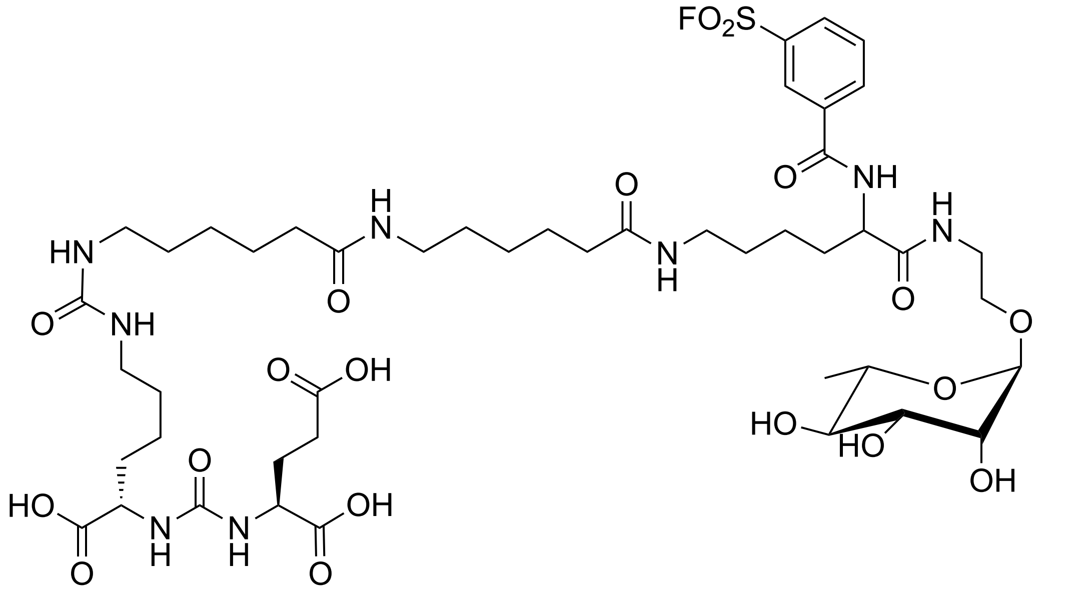

GUL-SO$_2$F-cARM (14). **41** (3.2 mg, 0.003 mmol) was dissolved in MeOH (300 μL) followed by addition of sodium methoxide solution (30 μL, 25 wt% in MeOH). The solution was stirred for 20 minutes then diluted with 10 mL water and quenched to pH 7 with a 0.1 M HCl solution. The solution was flash frozen and lyophilized. The resulting Fmoc/Acetyl deprotected product was directly resuspended with water (300 μL) containing DIPEA (1.2 μL, 0.007 mmol). To this solution, **33** (4.3 mg, 0.014 mmol, 90 μL in DMSO stock) was added. The solution was stirred for 30 minutes, then purified via HPLC to yield product (0.4mg, 13.4% yield over two steps, concentration determined via standard curve). HRMS-ESI $[M+H]^+$ *m/z* calc for $[C_{46}H_{74}FN_8O_{19}S^+]$ 1093.4769, found 1093.2444.

General Notes and Materials

ELISA - General notes and Materials

In all cases the ELISA plate is incubated at 37°C, using a plate sealer (Biolegend - 423601). streptavidin coated ELISA plates were purchased from Thermofisher Scientific (15500). Human Serum was purchased from Innovative Research (ISERAB100ML). Secondary anti-IgG antibody was purchased from Thermofisher Scientific (A18805), while anti-IgM antibody was purchased from Fisher Scientific (NC9048815). L-rhamnose monohydrate used as competitor was purchased from Sigma Aldrich (R3875-10G). BSA (Sigma Aldrich – A9576-50ML) and Tween-20 (Sigma Aldrich – P9416 – 50ML) were added to PBS to create Wash Buffer (WB, 0.5% Tween-20, 0.1% BSA, in PBS). A TMB substrate kit was purchased from Thermofisher Scientific (34021). Sulfuric acid was purchased from VWR (470302-860). Plate absorbance was measured at 450 nm using a Tecan Spark Plate Reader.

Biolayer Interferometry (BLI) General notes and Materials

IgG from human serum was purchased from Innovative Research (IHUIGGAP1000MG). Anti-23F antibody was purchased from Creative Biolabs (PABX-173). Biolayer Interferometry (BLI) was conducted using a ForteBio Octet Red96, and streptavidin biosensors (Sartorius - 18-5019) or ProG biosensors (Sartorius – 18-5082). For quench, a 5% skim milk (Nestle Carnation Instant Skim Milk) solution in 1X kinetics buffer was used. The instrument was set at 25°C, with 1000 RPM, and acquisition rate of 5 Hz. Each well of a black well plate (Greiner Bio-One - 655086) was filled with 200 µL of appropriate solution. Dissociation buffer was comprised of L-rhamnose (100 mM, Sigma Aldrich – R3875-10G) in 1X kinetics buffer to reduce avidity contribution to dissociation rate (k_{off}).

SDS-PAGE General notes and Materials

Human Serum was purchased from Innovative Research (ISERAB100ML). L-rhamnose monohydrate used as competitor was purchased from Sigma Aldrich (R3875-10G). 5(6)-Carboxyfluorescein was purchased from Sigma Aldrich (21877-5G-F). IgG from human serum was purchased from Innovative Research (IHUIGGAP1000MG). Anti-23F antibody was purchased from Creative Biolabs (PABX-173). Laemellie Buffer (2x) was purchased from Sigma Aldrich (S3401-10VL). Pre-cast Tris-Glycine Gels (4-20%) were purchased from Fisher Scientific (XP04202BOX). Standard protein ladder was purchased from Bio Rad (1610363). Gel fluorescence was imaged using a GE Typhoon imager (Cy2 channel for fluorescein, Cy5 channel for A647). A647-alkyne was purchased from Broadpharm (BP-28217). Gels were stained using Coomassie Stain from Bio Rad (1610786), and subsequently imaged using an Odyssey CLX imager. Gels were run at 90V for 15 minutes, followed by 120V for 50 minutes.

ADCC Reporter Assay General notes and Materials

Anti-23F antibody was purchased from Creative Biolabs (Custom Order with mouse IgG2a Fc). Human IgG from serum was purchased from Innovative Research (IHUIGGAP1000MG). Jurkat activation utilizing human IgG antibody source was quantified using the ADCC Reporter Bioassay kit (Promega G7010). Jurkat activation utilizing anti-23F antibody source was quantified using the ADCC Reporter Bioassay kit (Promega M1211). For desalting, centrifugal filters (10 kDa cut-off, 0.5 mL) were purchased from Fisher Scientific (UFC501024). White opaque 96 well flat-bottom plates were used to measure luminescence (Corning Costar, 3917). Luminescence was measured on a SpectraMax i3 plate reader (Molecular Devices). For PSMA competition controls, 2-PMPA was purchased from Sigma Aldrich (SML1612). L-rhamnose monohydrate used as competitor was purchased from Sigma Aldrich (R3875-10G). Hek-293T (PSMA+/-) cell lines were generously provided by Dr. Cyril Barinka (Institute of

Biotechnology CAS, Czech Republic). Ultra-low IgG FBS was purchased from Fischer Scientific (A3381901). RPMI-1640 was purchased as a powder from Fischer Scientific (31800089) and resuspended. DMEM was purchased as a powder from Fischer Scientific (12800082) and resuspended. Pen/Strep was purchased from Fischer Scientific (15140-122). FBS was purchased from Fischer Scientific (12484-028). Zeocin was purchased from Fischer Scientific (R25001). HEK-PSMA cells were cultured in DMEM media with 2mM L-glut, 1% Pen/Strep, 10% FBS, 50 µg/mL Zeocin. HEK cells were cultured in DMEM media with 2mM L-glut, 1% Pen/Strep, 10% FBS.

Microscopy General notes and Materials

Anti-23F antibody was purchased from Creative Biolabs (Custom Order with mouse IgG2a Fc). Paraformaldehyde was purchased from Electron Microscopy Sciences (15714), diluted to 4% in PBS, and aliquoted/frozen for stock solutions. F(ab')2-Goat anti-Mouse IgG (H+L) secondary antibody-PE conjugate, was purchased from Thermo Fisher Scientific (12-4010-82). Secondary antibody solution was prepared through 50x dilution in RPMI. Cells were plated and imaged using flat bottom 96-well plates (Falcon - 353072). Images were acquired using an Incucyte S3 Live-Cell Imager. L-rhamnose monohydrate used as competitor was purchased from Sigma Aldrich (R3875-10G). Hek-293T (PSMA+/-) cell lines were generously provided by Dr. Cyril Barinka (Institute of Biotechnology CAS, Czech Republic). RPMI-1640 was purchased as a powder from Fischer Scientific (31800089) and resuspended. DMEM was purchased as a powder from Fischer Scientific (12800082) and resuspended. Pen/Strep was purchased from Fischer Scientific (15140-122). FBS was purchased from Fischer Scientific (12484-028). Zeocin was purchased from Fischer Scientific (R25001). HEK-PSMA cells were cultured in DMEM media with 2mM L-glut, 1% Pen/Strep, 10% FBS, 50 µg/mL Zeocin. HEK cells were cultured in DMEM media with 2mM L-glut, 1% Pen/Strep, 10% FBS.

ADCP Reporter General notes and Materials

ADCP experiments were run on a BD LSRII or Fortessa Flow Cytometer. PSMA expression was confirmed with an anti-PSMA antibody alexa 647 conjugate (Novus Biologicals, Catalog #FAB4234R). Anti-23F antibody was purchased from Creative Biolabs (Custom Order with mouse IgG2a Fc). Human IgG from serum was purchased from Innovative Research (IHUIGGAP1000MG). Hek-293T (PSMA+/-) cell lines were generously given by Dr. Cyril Barinka (Institute of Biotechnology CAS, Czech Republic). U937 cells were generously given by Dr. John Valliant (McMaster University, Canada). LNCaP cells were generously given by Dr. Karen Mossman (McMaster University, Canada). IFN-γ was purchased from Fischer Scientific (PHC4031). Ultra-low IgG FBS was purchased from Fischer Scientific (A3381901). RPMI-1640 was purchased as a powder from Fischer Scientific (31800089) and resuspended. DMEM was purchased as a powder from Fischer Scientific (12800082) and resuspended. DiD cell dye was purchased from Fischer Scientific (V22887). DiO cell dye was purchased from Fischer Scientific (V22886). TrypLE Express was purchased from Fischer Scientific (12604013). 96-Well U-bottom plates were purchased from FischerScientific (08-772-17). Pen/Strep was purchased from Fischer Scientific (15140-122). FBS was purchased from Fischer Scientific (12484-028). Zeocin was purchased from Fischer Scientific (R25001). HEK-PSMA cells were cultured in DMEM media with 2mM L-glut, 1% Pen/Strep, 10% FBS, 50ug/mL Zeocin. HEK cells were cultured in DMEM media with 2mM L-glut, 1% Pen/Strep, 10% FBS. LNCaP/C4-2 cells were cultured in RPMI media with 1% Sodium Pyruvate, 1% Pen/Strep, 10% FBS. U937 monocytes were cultured in RPMI media with 2mM L-Glut, 1% Pen/Strep, 10% FBS.

Experimental Procedures

ELISA: Non-Covalent Recruitment of Anti-Rhamnose Antibody From Human Serum

Each well of pre-blocked ELISA plate was hydrated by addition of 100 µL of wash buffer (WB) and incubation for 1 hour at 37°C. During plate hydration, 2% human serum was made by diluting 100% human serum with WB or WB with dissolved rhamnose (for rhamnose Comp conditions, 100 mM final Rhamnose). The ELISA plate was emptied through rapid inversion, and each well was washed 3X with wash buffer (100 µL each wash, removed via rapid plate inversion). A solution of each ARM (ARM **1-3**, 4 µM, 50 µL) was added to appropriate wells followed by 30-minute incubation at room temperature. The ELISA plate was emptied once again by rapid plate inversion, followed by 3x washes with WB. Each well was then incubated with human serum (50 µL, either 2%, or 2% with rhamnose competitor), and incubated for 1 hour at 37°C. The plate was then emptied, and washed 3x with WB. To each appropriate well, secondary antibody solution was added and incubated for 1 hour at 37°C (50 µL, either anti-IgG or anti-IgM, secondary antibody diluted 3000x in WB for working solution). The plate was then emptied, and washed 3x with WB. TMB substrate and Hydrogen peroxide (from kit) were mixed 1:1, and 50 µL was added to each ELISA well. This solution was incubated in the ELISA plate at room temperature for approximately 2 minutes (colour gradient develops), followed by addition of sulfuric acid quench (2M, 50 µL). Plate absorbance at 450 nm was read immediately following quench.

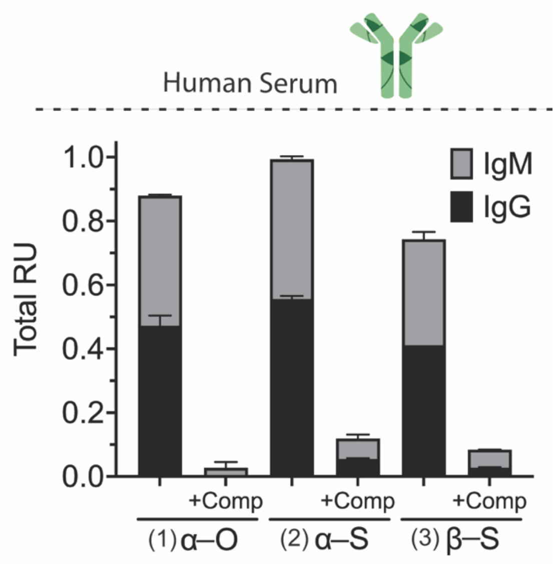

Figure S1. Recruitment of anti-rhamnose IgG/IgM directly from human serum using probes (1), (2), and (3) measured via ELISA assay. High-capacity streptavidin plates were loaded with appropriate probe followed by addition of 2% human serum. Recruitment was evaluated through addition of anti-IgG or anti-IgM HRP-conjugated secondary antibody.

BLI: Approximation of anti-Rhamnose K_d (Figure 1B/C)

Streptavidin biosensors were first equilibrated in 1X kinetics buffer for 10 minutes prior to execution of experimental sequence. Next biosensors were equilibrated in 1X kinetics buffer for 5 minutes followed by submersion in ARM solution (**1-3**, 500 nM) or 1X kinetics buffer (for "Blank") for 5 minutes to allow compound loading. After compound loading, sensors were placed in 5% skim milk solution for 120 seconds followed by 5 minutes in 1X kinetics buffer to establish baseline. Next, biosensors were submerged in either anti-23F antibody or human IgG to allow antibody association for 10 or 15 minutes respectively. Finally, biosensors were submerged in dissociation buffer for 10 minutes.

To approximate K_d, each antibody source was assayed at three association concentrations (anti-23F at 500 nM, 250 nM, and 125 nM. IgG at 105 µM, 52.5 µM, and 26.2 µM). Raw data baselined to the association step was plotted in GraphPad Prism. Due to a small amount of association seen with the "Blank" condition with IgG, all IgG curves were baselined to "Blank". The anti-rhamnose concentration in IgG was assumed to be 3% total IgG for approximation of k_{on}.[3,4] The k_{on} of each association curve was determined via $\frac{k_{obs}}{Conc\ Anti-Rha}$, where k_{obs} = "K"$_{Fast}$ derived with the "Two Phase Association" function. The k_{off} of each dissociation curve was determined via the "Two Phase Decay" function, where $k_{off} = K_{Fast}$. K_d was determined as $\frac{k_{off}}{k_{on}}$.

Table. S1 Kinetic measurements for IgG polyclonal antibody

	105 µM IgG			52.5 µM IgG			26.2 µM IgG			
	k_{on} ($M^{-1}s^{-1}$)	k_{off} (s^{-1})	K_d (µM)	k_{on} ($M^{-1}s^{-1}$)	k_{off} (s^{-1})	K_d (µM)	k_{on} ($M^{-1}s^{-1}$)	k_{off} (s^{-1})	K_d (µM)	**Avg K_d (µM)**
**α-O (1)**	8235	1.058	128	13810	1.112	81	20749	1.199	58	**89±36**
**α-S (2)**	8194	0.8262	101	14298	0.97	68	21981	1.103	50	**73±26**
**β-S (3)**	6254	0.3901	62	11537	0.481	42	19771	0.576	29	**44±17**

Table. S2 Kinetic measurements for anti-23F monoclonal antibody

	500 nM Anti-23F			250 nM Anti-23F			125 nM Anti-23F			
	k_{on} ($M^{-1}s^{-1}$)	k_{off} (s^{-1})	K_d (µM)	k_{on} ($M^{-1}s^{-1}$)	k_{off} (s^{-1})	K_d (µM)	k_{on} ($M^{-1}s^{-1}$)	k_{off} (s^{-1})	K_d (µM)	**Avg K_d (µM)**
**α-O (1)**	6.3E+05	4.673	7.4	9.2E+05	5.773	6.2	1.1E+06	5.591	5.2	**6.3±1.1**
**α-S (2)**	5.6E+05	3.509	6.2	7.3E+05	4.378	6.0	7.7E+05	4.018	5.2	**5.8±0.5**

Small Molecule Docking

Autodock docking studies used the anti-23F crystal structure (PDB - 4HIJ). Briefly, the native ligand of the crystal structure was removed, and compound binding was limited to a 60x60x60 grid point box surrounding the native ligand binding site. Figures S1-S2 demonstrate potential binding conformations which place the electrophilic chemistry proximal to an appropriate nucleophilic residue.

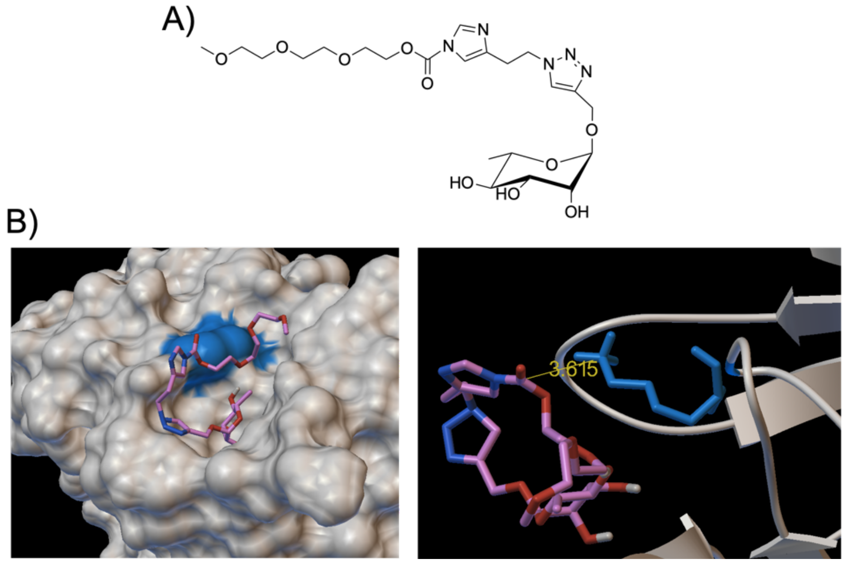

Figure S2. A) Representative Rha-AI-cARM fragment docked onto monoclonal anti-23F Fab Crystal Structure. B) Autodock representations of Rha-AI-cARM fragment binding to Fab, with AI proximal to heavy chain lysine 104.

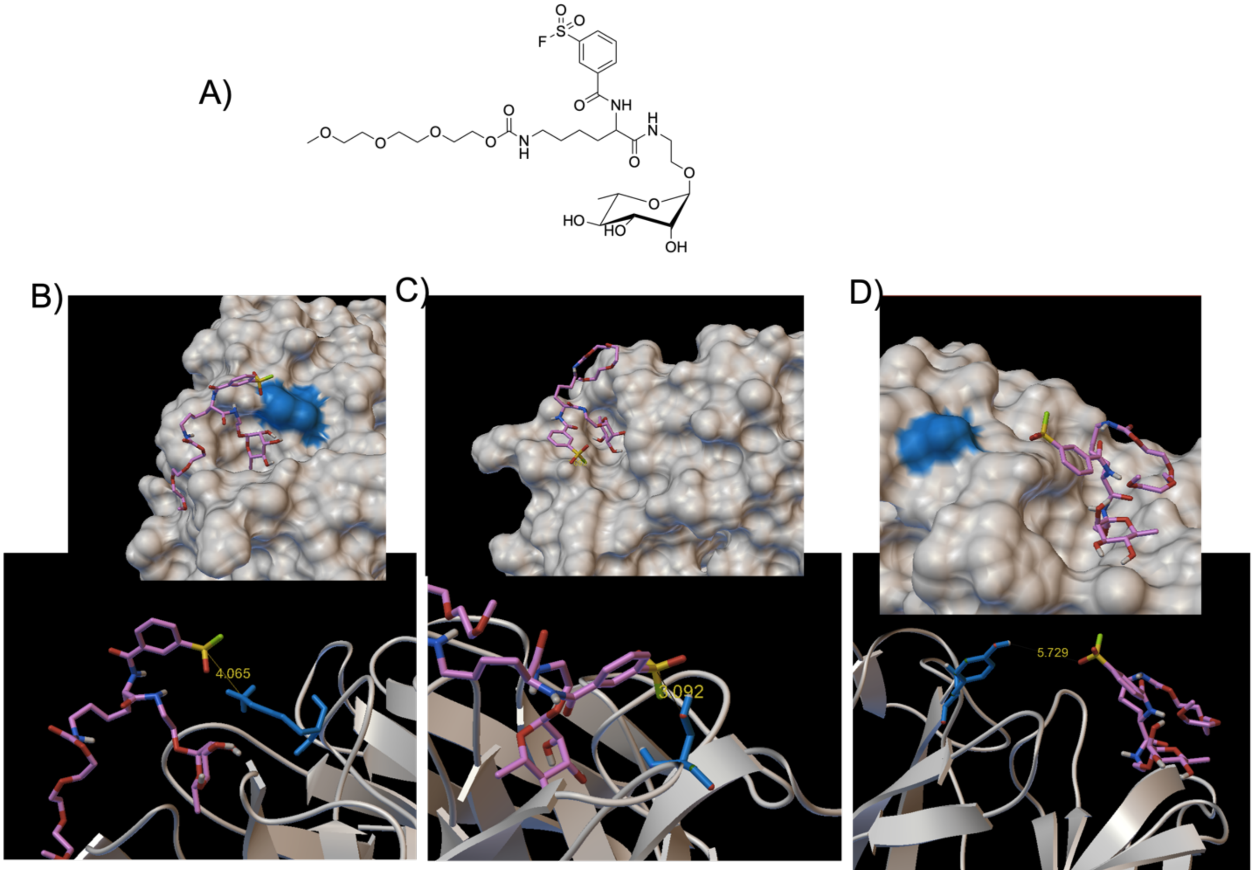

Figure S3. A) Representative Rha-SO_2F-cARM fragment docked onto monoclonal anti-23F Fab crystal structure. B) Autodock representations of Rha-SO_2F-cARM fragment binding to Fab, with SO_2F proximal to heavy chain lysine 104. C) Autodock representations of Rha-SO_2F-cARM fragment binding to Fab, with SO_2F proximal to heavy chain Serine 53. D) Autodock representations of Rha-SO_2F-cARM fragment binding to Fab, with SO_2F proximal to light chain tyrosine 32.

ELISA: Covalent Engagement of Serum Endogenous Anti-Rhamnose Antibody (Figure 2B-main text)

Each ARM (**1**-**2**) and cARM (**4**-**5**) was incubated directly in appropriate 2% human serum solution at a concentration of 2 µM for 48 hours. For "Comp" conditions, each compound was incubated in 2% human serum with 500 mM rhamnose for the entire 48-hours. For "Quench" conditions, 1 M rhamnose stock in WB was added to the 2% human serum/compound solution at the end of 48-hour incubation for a 100 mM final rhamnose concentration. These solutions were added to prepared ELISA plate (see below), as "serum solution".

Each well of the pre-blocked ELISA plate was hydrated by addition of 100 µL of wash buffer (WB) and incubation for 1 hour at 37°C. After hydration, the ELISA plate was emptied through rapid inversion, and each well was washed 3X with wash buffer (100 µL each wash, removed via rapid plate inversion). Each "serum solution" (50 µL) was added to appropriate wells followed by 30-minute incubation at room temperature. The ELISA plate was emptied once again by rapid plate inversion, followed by 3x washes with WB. To each appropriate well, secondary antibody solution was added and incubated for 1 hour at 37°C (50 µL, either anti-IgG or anti-IgM, secondary antibody diluted 3000x in WB for working solution). The plate was then emptied, and washed 3x with WB. TMB substrate and Hydrogen peroxide (from kit) were mixed 1:1, and 50 µL was added to each ELISA well. This solution was incubated in the ELISA plate at room temperature for approximately 2 minutes (colour gradient develops), followed by addition of sulfuric acid quench (2 M, 50 µL). Plate absorbance at 450 nm was read immediately following quench.

SDS-PAGE: Validation of Covalent Labelling from Human Serum (Figure 2C)

Each cARM (**6**-**8**, 4 µM) was incubated with anti-23F antibody (2 µM) in 10% human serum for 24 hours. For "Rha.C" condition, L-Rhamnose competitor (100 mM) was included during incubation. For "Fluor.C" condition, fluorescein competitor (2.5 mM) was included during incubation. Immediately following incubation all samples were diluted 2x with laemelli buffer, heated at 95°C for 3 minutes, and loaded directly on a reducing gel (10 µL loaded in each well). Gels were first imaged on a Typhoon imager to visualize protein covalent labelling with fluorescent tag. Following fluorescent imaging, gels were stained via Coomassie stain and imaged to visualize protein content.

Hydrolysis Half-life Determination

Each cARM was diluted to 80 µM in PBS and incubated at room temperature. At different timepoints, an aliquot was removed for LCMS analysis. The relative intensities of “broken” and “in-tact” cARM were compared via TIC over time to yield Figure S4.

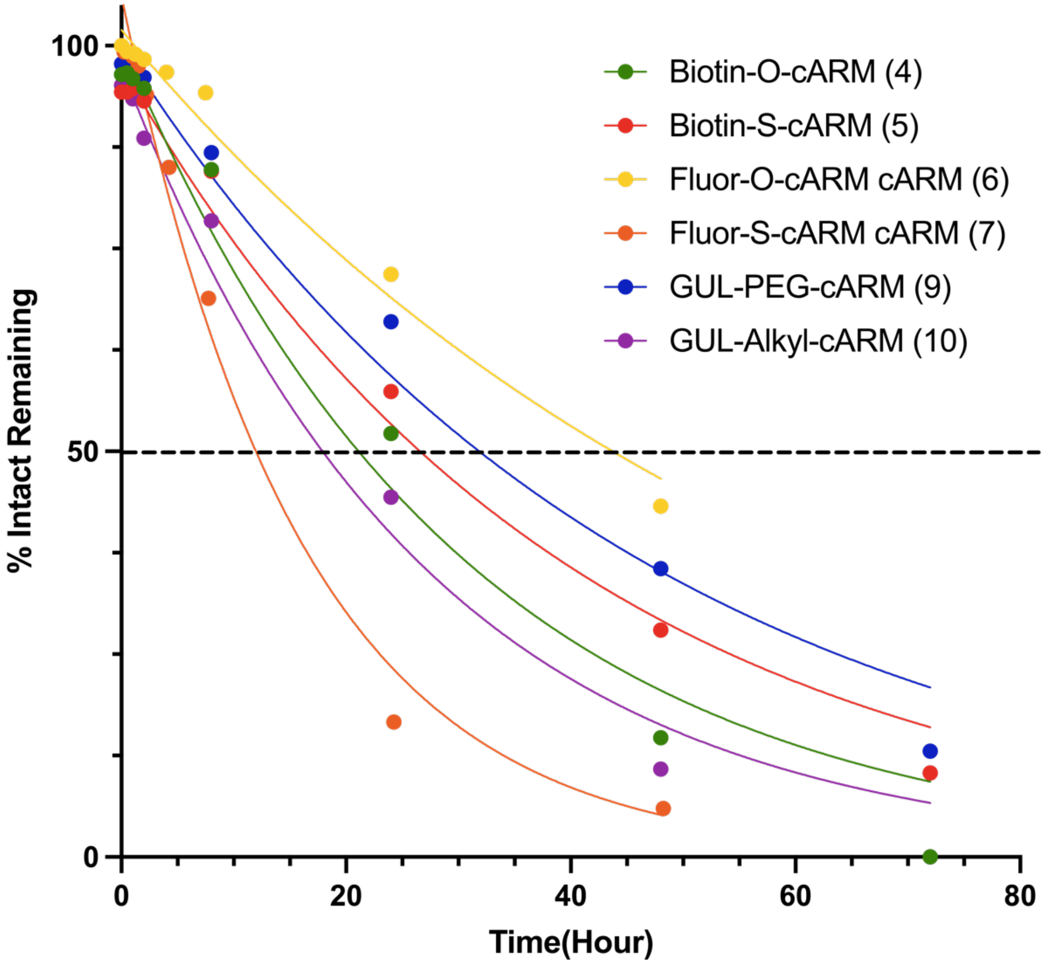

Figure S4. Hydrolysis over time of select cARMs.

SDS-PAGE: Approximation of Rha-AI-cARM Covalent Labelling kinetics using model Monoclonal anti-23F (Figure 2D)

A647 - cARM **8** (2 µM – 100 µM) was incubated with anti-23F or IgG antibody (2 µM) for up to 4 hours in PBS. After incubation, all conditions were simultaneously diluted 2x with laemelli buffer (containing L-rhamnose quench, final L-rhamnose concentration 100 mM), heated at 95°C for 3 minutes, and loaded directly on a reducing gel (10 µL loaded in each well). The gels were imaged on a Typhoon imager to visualize protein covalent labelling with fluorescent tag, (example gel seen in Figure 2D). ImageJ was used to perform densitometry analysis on each gel, allowing comparison of relative labelling rates, (Figure S5A). To convert RFU to % labelled protein for kinetic approximations, a standard curve of A647-

alkyne was prepared, run on a gel, imaged, and analyzed similarly to protein gel to provide a standard curve of RFU vs A647 concentration, (Figure S5B). Plotting this standard curve in GraphPad Prism and applying linear regression provided an equation to convert RFU to concentration of A647. Known concentrations of A647 combined with known antibody concentrations were applied to Figure S5A to provide Figure S5C. The slope of each curve (provided by linear regression in GraphPad Prism), vs associated construct concentration was used to derive Figure 2D, and Figure S5D. Figure S5D demonstrates only the initial rate of Figure 2D. Slope of Figure S5C (k_{obs}(min^{-1})) including 0h – 4h, 0h - 2h, and 0h – 1h was used for error calculation. The slope in Figure S5D approximates $\frac{k_{inact}}{k_I}$, and the K_I was derived from Figure 2D as cARM concentration at half maximal rate.[5] Subsequent calculation of k_{inact} can be completed with Equation S1.

Equation S1. Approximation of k_{inact}.

$$k_{inact} = Initial\ slope * K_I$$

Note: Initial slope is calculated from Figure S5D.

Note: The value of KI used is 15 µM, approximated as cARM concentration at half maximal rate (Figure 2D – main text).

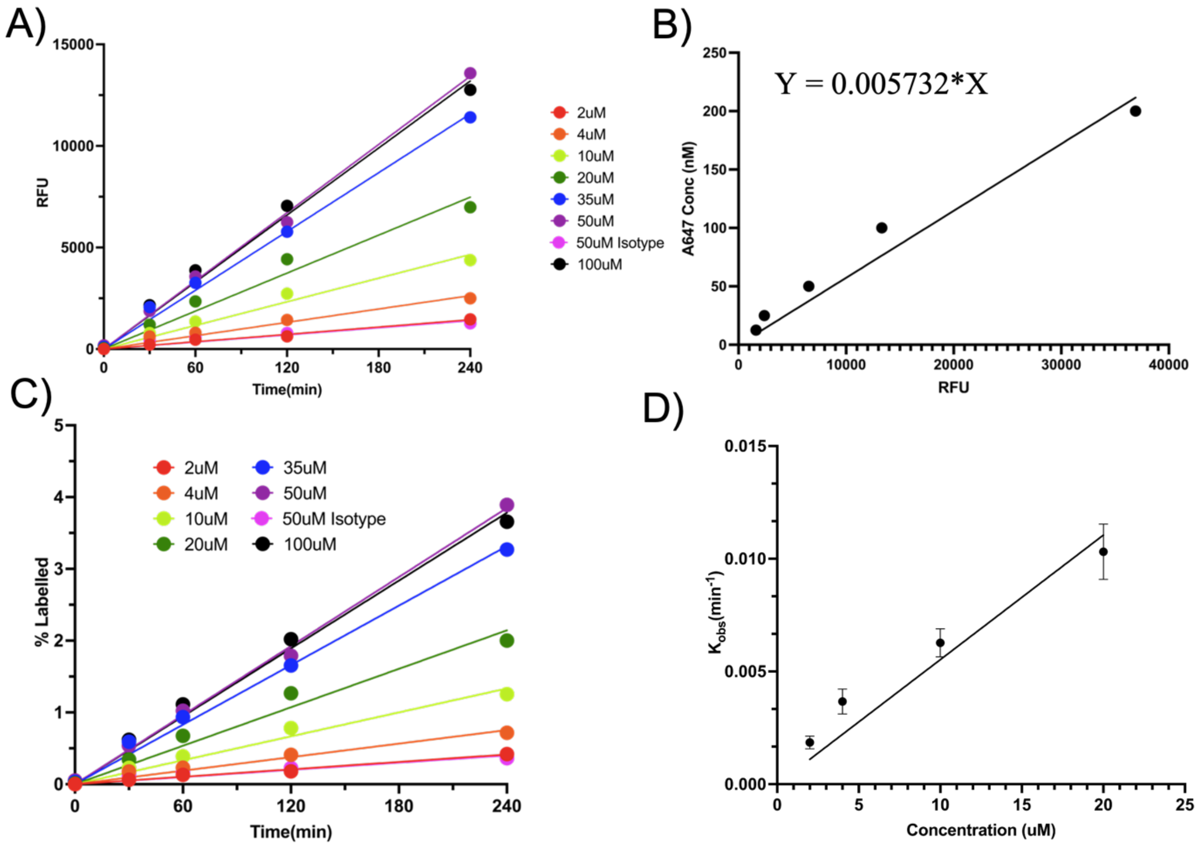

Figure S5. Supplemental figures to derive Figure 2D (main text). A) Plotted raw densitometry analysis of initial rate gels. B) Standard curve of A647 RFU vs loaded concentration.. C) Plotted densitometry analysis of initial rate gels converted to % antibody labelled using Figure S5B and S5A. D) Initial rate vs cARM concentration derived from Figure S5C, representing initial rate of Figure 2D (main text).

Table S3. Initial slopes ($\frac{k_{inact}}{k_I}$) from Figure S5D (Rha-AI-cARM labelling rate).

**Initial Slope ($\frac{k_{inact}}{k_I}$)**	
	**Heavy Chain**
**$uM^{-1}min^{-1}$**	0.000553
**$M^{-1}s^{-1}$**	9.2
**Std. Error ($M^{-1}s^{-1}$)**	0.5

ADCC Reporter Assay: Immune Induction with Rha-AI-cARMs (Figure 3)

Prior to antibody addition to target cells/jurkats, IgG antibody (230 μM) was incubated with cARM/ARM (50 μM) for 72 hours. When L-rhamnose competitor is included, 100 mM is doped in during incubation. After incubation, each condition was desalted with centrifugal filters (10 kDa cut off) then resuspended with RPMI. When 2-PMPA is included as PSMA competitor, 2.5 mM is doped in post desalting. Each antibody-cARM/ARM mixture was then diluted in a 12-point 2x dilution series in RPMI. These "antibody solutions" were added to pre-plated target cells for analysis.

Hek-PSMA cells were seeded in opaque white 96-well plates at 50,000 cells/well, and incubated (37°C, 5% CO_2) for 24 hours. After incubation, cell supernatant was removed slowly. Directly to each well, 25 μL of assay media (4% ultra-low IgG FBS in RPMI) followed by 25 μL of appropriate "antibody solution" were added. Cells were then incubated (37°C, 5% CO_2) for 30 minutes. Directly to each well, 25 μL of engineered Jurkats (from Promega ADCC Reporter Bioassay Kit G7010) in assay media were added. Cells were incubated (37°C, 5% CO_2) for 24 hours then allowed to sit at room temperature for 15 minutes. BioGlo reagent (75 μL) was then added to each well. Luminescence was measured 5 minutes after addition of BioGlo reagent.

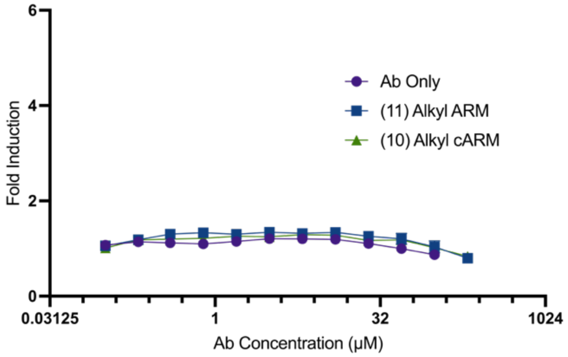

Figure S6. Supporting ADCC reporter assay data utilizing PSMA(-) HEK cells, (man manuscript Figure 3).

BLI: Rha-AI-PEG-cARM (9) vs Rha-AI-Alkyl-cARM (10) Ternary Complex Experiment

Prior to BLI assay, each compound (cARM **9** or cARM **10**, 100 µM) was incubated with anti-23F antibody (2 µM) or anti-DNP (mmAb from Ref 1, 2 µM) for 24 hours. These “antibody solutions” were loaded directly into BLI experiment.

ProG biosensors were first equilibrated in 1X kinetics buffer for 10 minutes prior to execution of experimental sequence. Next, biosensors were equilibrated in 1X kinetics buffer for 5 minutes followed by submersion into “antibody solutions” for 15 minutes to allow association. Probes were then submerged into dissociation buffer (100 mM L-rhamnose in 1X kinetics buffer) for 10 minutes followed by equilibration in 1X kinetics buffer for 5 minutes to remove non-covalently associated cARM from antibody. Probes were then submerged into a PSMA solution (500 nM) to allow association onto covalently-modified antibodies for 15 minutes, followed by dissociation in 1X kinetics buffer for 15 minutes. Raw data was plotted in GraphPad prism baselined to PSMA association for Figure S7.

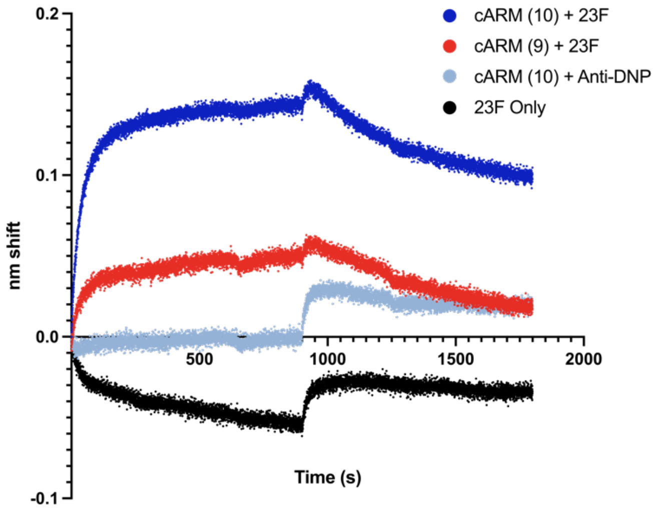

Figure S7. BLI analysis comparing anti-23F labelling capacity of Alkyl-cARM (10) to Peg- cARM (9).

BLI: Monitoring Covalent Reaction of novel SO_2F cARM (12) (Figure 4B)

Each compound (10 µM) was incubated with human IgG (230 µM) or BSA (230 µM) for up to 25 hours. Prior to BLI experiment, each timepoint was quenched simultaneously via 8x dilution with L-rhamnose buffer (final L-rhamnose concentration 100 mM). These quenched “antibody solutions” were used immediately in BLI experimental.

Streptavidin biosensors were first equilibrated in 1X kinetics buffer for 10 minutes prior to execution of experimental sequence. Upon experiment start, biosensors were equilibrated in 1X kinetics buffer for 3 minutes followed by submersion in “antibody solutions” for 3 minutes to allow association. Finally, biosensors were submerged in dissociation buffer for 3 minutes. Raw data was baselined to association observed using non-covalent ARM (1) to differentiate and measure signal resulting from covalent reaction. The final amplitude during the association step was plotted vs incubation time to yield Figure 4, (Figure S8).

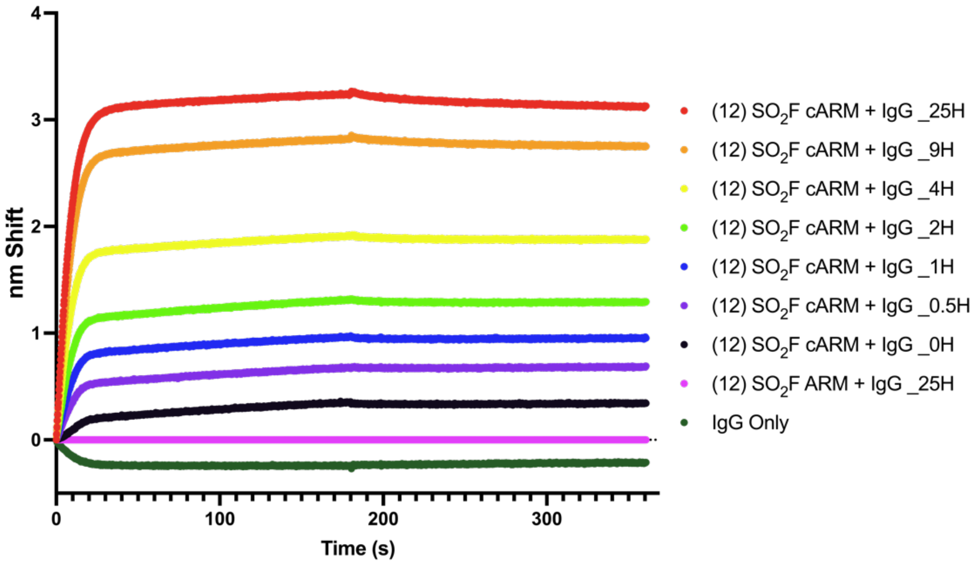

Figure S8. Example raw association/dissociation sensorgram data using SO_2F cARM (12) incubated with IgG, baselined to ARM association.

SDS-PAGE: Approximation of Rha-SO_2F-cARM (13) Covalent Labelling Rate of Model Monoclonal anti-23F (Figure 4C)

A647 - cARM **13** (0.5 µM – 16 µM) was incubated with anti-23F or IgG antibody (2 µM) for up to 2 hours in PBS. The rest of the procedure is orthogonal to assay described above for cARM **8** (Figure S5, Figure 2D).

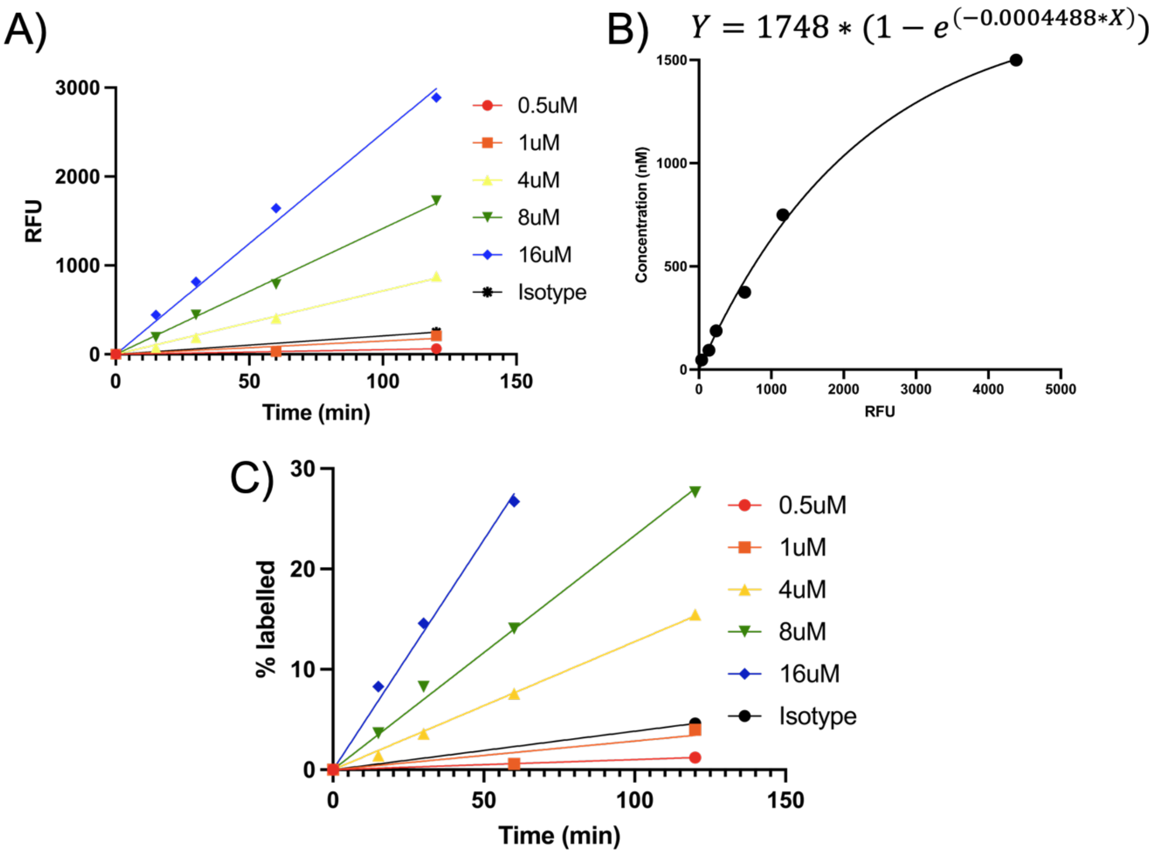

Figure S9. Supplemental figures to derive Figure 4C (main text). A) Plotted raw densitometry analysis (sum of heavy and light chain labelling) of initial rate gels. B) Standard curve of A647 RFU vs loaded concentration. "One-Phase Association" in GraphPad Prism was used to fit the curve. C) Plotted densitometry analysis of initial rate gels (sum of heavy and light chain labelling) converted to % antibody labelled using Figure S9B and S9A. The last timepoint for 16 µM cARM was removed to maintain linearity.

Note: Plot comparable to Figure S5D (initial rate of labelling vs cARM concentration, derived from Figure S9C) is represented as Figure 4C (main text).

Table S4. Slopes ($\frac{k_{inact}}{k_I}$) associated with main text Figure 4C (SO_2F cARM labelling rate).

**Initial Slope ($\frac{k_{inact}}{k_I}$)**			
	**Sum**	**Heavy Chain**	**Light Chain**
**$uM^{-1}min^{-1}$**	0.03021	0.01834	0.01187
**$M^{-1}s^{-1}$**	503.5	305.7	197.8
**Std. Error ($M^{-1}s^{-1}$)**	9.1	6.3	4.2

Mass Spectrometry Study – Anti-23F Labelling

Anti-23F (2.7 μM) was incubated with 10 μM of Biotin-AI-cARM **4** or Biotin-SO_2F-cARM **12**, or PBS (antibody only) for 45 hours before MS analysis. Analysis was completed by Bioinformatics Solutions Inc. Briefly, for intact protein analysis each sample was treated with PNGase F to remove glycans followed by LC-MS analysis. To determine site of labelling, each sample was digested with tryspin, chymostrypsin, and pepsin then analyzed by LC-MS/MS on a timsTOF Pro. Peptide spectra were mapped to a known antibody sequence (anti-23F antibody, Creative Biolabs - PABX-173). No labelling was identified with acyl imidazole containing cARM **4**. Labelling of only lysine 104 of antibody heavy chain was seen for SO_2F cARM **12**, (Figure S10). The labelling was partially quantified via intact MS, demonstrating approximately 30% labelling post MS protocol (Figure S11)

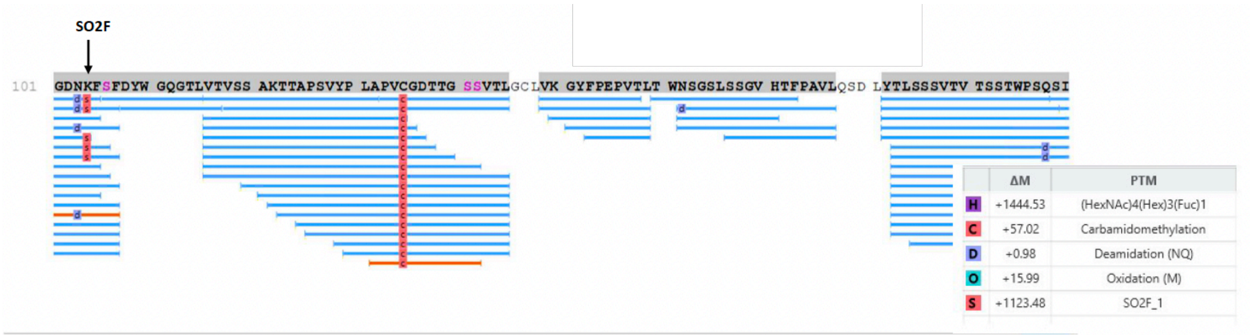

Figure S10. Sample analysis of peptide mapping of anti-23F heavy chain. Modification with cARM **12** is shown on lysine 104.

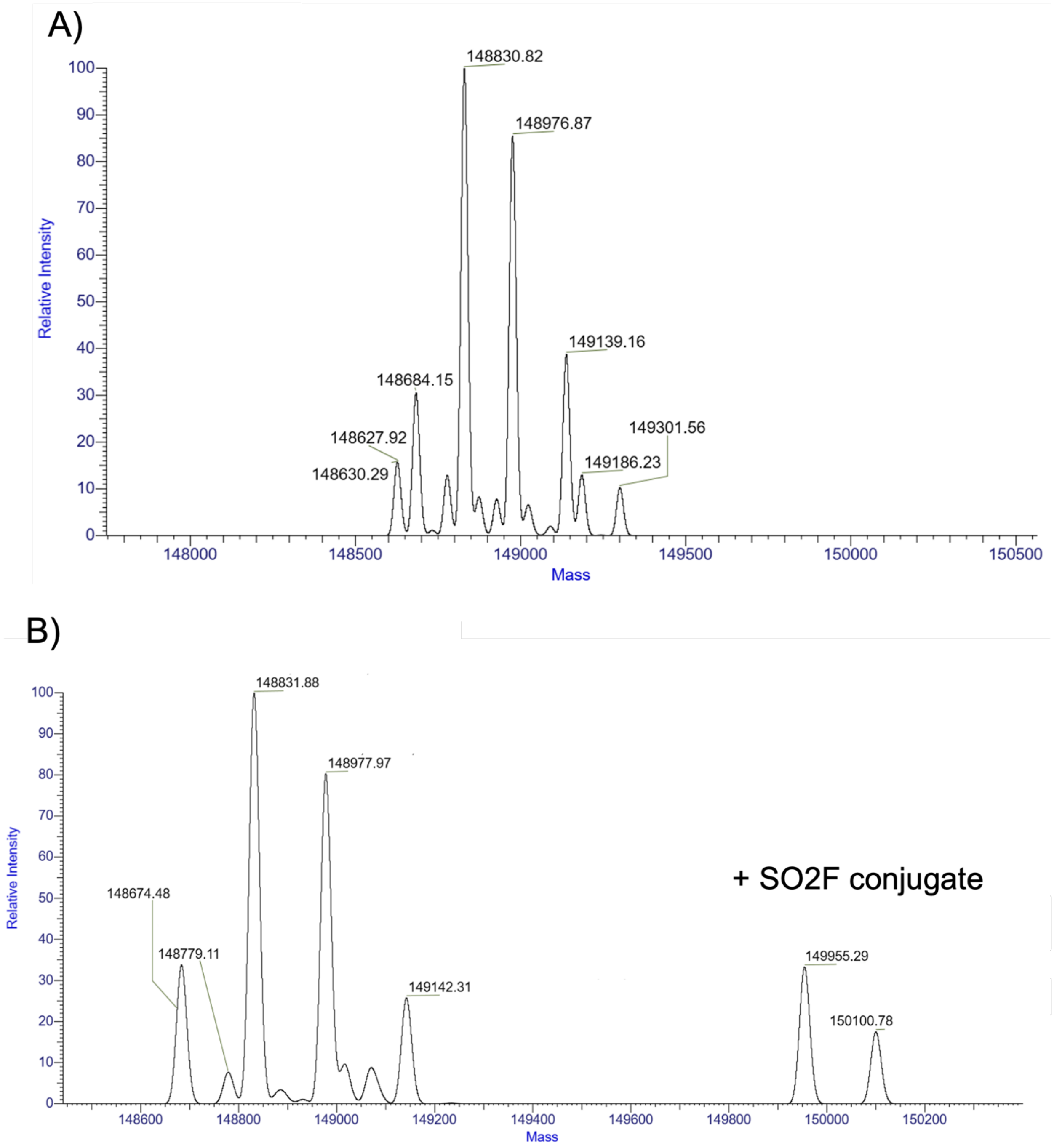

Figure S11. Intact MS of antibody only (A) or antibody labelling with cARM **12** (B).

Microscopy: Visualization of Induced Antibody Recruitment (Figure 5B Main Text)

Prior to antibody addition to target cells, anti-23F antibody (4 μM) was incubated with cARM/ARM (8 μM) for 24 hours. When L-rhamnose competitor was included, 100 mM was doped in during incubation. After incubation, each antibody-cARM/ARM mixture ("antibody solution") was diluted to 50 nM antibody for addition to pre-plated target cells.

Hek-PSMA cells were seeded in a 96-well plate at 10,000 cells/well, and incubated (37°C, 5% CO_2) for 24 hours. After incubation, cell supernatant was removed slowly. Directly to each well, 30 μL of an appropriate "antibody solution" was added and incubated for 20 minutes (room temperature). After incubation, 30 μL of secondary antibody solution was added to each well and incubated for 20 minutes (room temperature). Directly to each well, 40 μL of PFA (4% in PBS) was added, and incubated for 20 minutes (room temperature). The supernatant of each well was slowly removed. Wells were resuspended with 200 μL imaging solution (4% FBS in RPMI) and incubated at 37°C for 30 minutes. Plates were then imaged on an Incucyte imager.

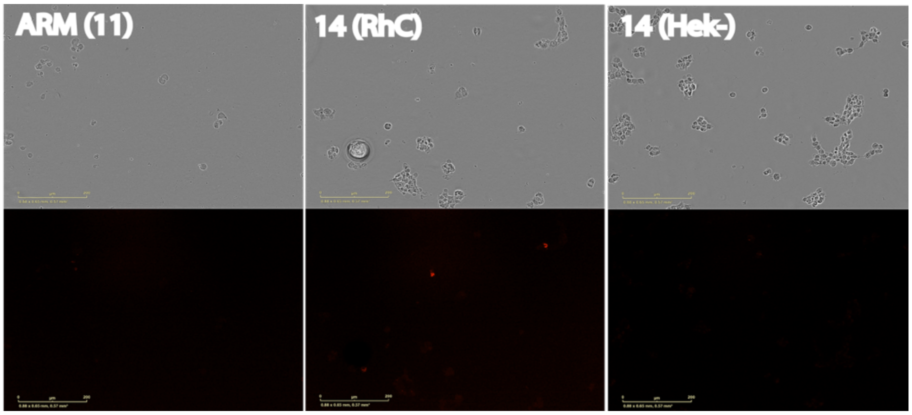

Figure S12. Supporting microscopy data for main text Figure 5B. Confocal (top), and fluorescent image (bottom) of Hek-PSMA cells treated with anti-23F antibody pre-incubated with ARM **11** or cARM **14** with L-rhamnose competitor (left two images). The rightmost image represents PSMA (-) Hek cells treated with anti-23F antibody pre-incubated with cARM (14).

<u>*ADCC Reporter Assay (Figure 5C Main Text, Figure S13)*</u>

IgG antibody (230 μM) was incubated with cARM/ARM (9 μM, Figure 5C) for 24 hours or, Anti-23F antibody (4 μM) was incubated with cARM/ARM (8 μM, Figure S13) for 24 hours. When L-rhamnose competitor is included, 100 mM is doped in during incubation. After incubation, each antibody-cARM/ARM mixture was then diluted in a 12-point (Figure 5C) or 8-point (Figure S13) 2x dilution series in RPMI. Where 0-time incubation was used, cARM was doped into antibody solution immediately prior to dilution series. These "antibody solutions" were added to pre-plated target cells immediately.

Hek-PSMA cells were seeded in opaque white 96-well plates at 50,000 cells/well, and incubated (37°C, 5% CO_2) for 24 hours. After incubation, cell supernatant was removed slowly. Directly to each well, 25 μL of assay media (4% ultra-low IgG FBS in RPMI) followed by 25 μL of appropriate "antibody solution" were added. Cells were then incubated (37°C, 5% CO_2) for 30 minutes. Directly to each well, 25 μL of engineered Jurkats (Figure 5C: from Promega ADCC Reporter Bioassay Kit G7010, Figure S13: from Promega ADCC Reporter Bioassay Kit M1211) in assay media were added. Cells were incubated (37°C, 5% CO_2) for 24 hours then allowed to sit at room temperature for 15 minutes. BioGlo reagent (75 μL) was then added to each well. Luminescence was measured 5 minutes after addition of BioGlo reagent.

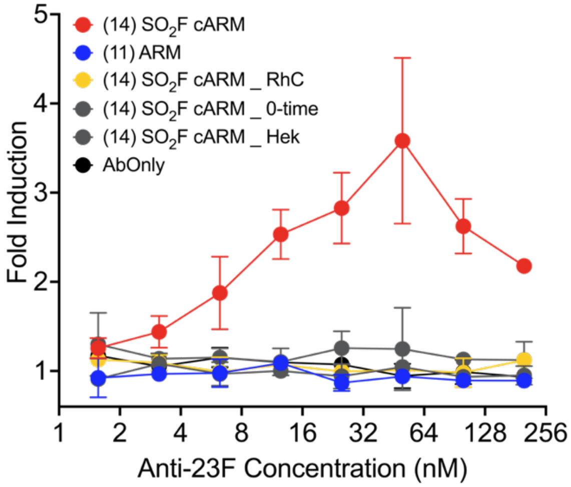

Figure S13. Jurkat activation assay of anti-23F antibody-cARM/ARM solutions.

ADCP: Capacity of cARMs to Induce Specific ADCP of Cancer Targets (Figure 5D Main Text)

Prior to antibody addition to target cells, human IgG or anti-23F antibody (4 µM) was incubated with cARM/ARM (8 µM) for 24 hours. After incubation, each antibody-cARM/ARM mixture ("antibody solution") was diluted 4x to 1 µM, then diluted in an 8-point 2x dilution series for addition to target/effector cells.

To prepare effector monocytes, U937 monocytes were seeded in complete media with IFN-γ (0.1mg/mL) at 500,000 cells/mL, and incubated for 24 hours. After incubation, activated monocytes were washed 2X with RPMI. Monocytes were then resuspended to a concentration of 1 million cells/mL in staining solution (1.9 µM Vybrant DiD Cell-Labelling Solution in RPMI) and incubated for 30 minutes (37°C, 5% CO_2). Monocytes were then washed 3x with warm assay media (AM, 14% Ultra-Low IgG FBS in RPMI) and resuspended to a concentration of 3.0×10^6 million cells/mL to be used in assay (50 µL holds 150,000 cells).

Target cells (90% confluent in a T-150 flask) were suspended with TrypLE and quenched with complete growth media. These cells were washed 2X with RPMI. Cells were then suspended to a concentration of 1 million cells/mL in staining solution (5.7 µM Vybrant DiO Cell-Labelling Solution in RPMI) and incubated for 30 minutes (37°C, 5% CO_2). Cells were then washed 3x with warm AM and resuspended to a concentration of 6.0×10^6 million cells/mL to be used in assay (25 µL holds 150,000 cells).

To a U-bottom 96-well plate, 25 µL of target cells followed by 25 µL of each "antibody solution" were added (for PSMA expressions check, 1.5 µL anti-PSMA A647 antibody was added with 23.5 µL assay media in lieu of "antibody solution"). Next, 50 µL of activated monocytes were added, and the plate was centrifuged at 800 rpm for 2 minutes. The plate was incubated for 1 hour (37°C, 5% CO_2) then placed on ice for immediate analysis via flow cytometry. DiO stained cells were detected in the A488 channel, DiD stained cells were detected in the APC Cy7 channel, PSMA expression was confirmed with the Alexa 647 channel. The following voltages were used: FSC: 390, SSC: 290, A488: 310, APC Cy7: 350, A647: 400. ADCP was determined by plotting monocyte stain against target stain, and was quantified as

$\% \, Target \; Phagocytosed = \left(\frac{Double \; Positive \; Events}{Target \; Only \; Events + Double \; Positive \; Events} \right) * 100$. This was normalized to antibody only control.

Spectra and Characterization Data

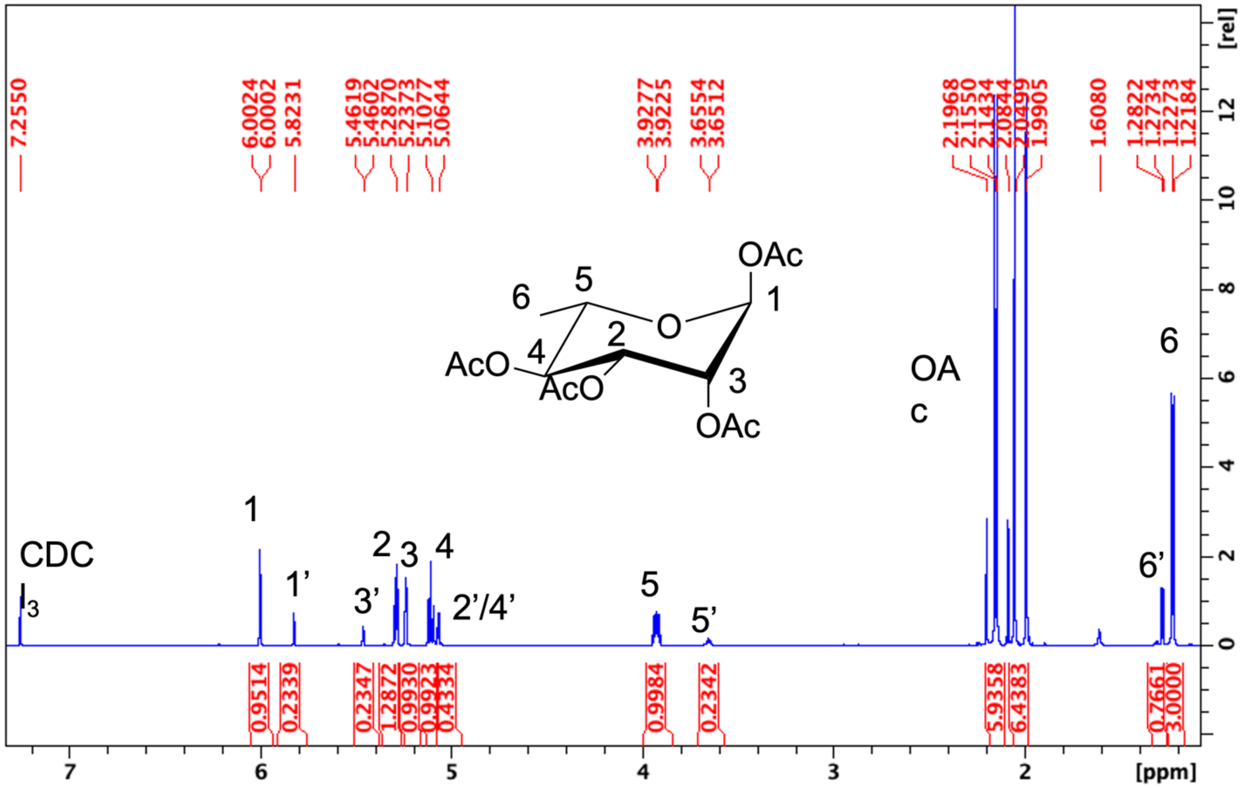

Figure S14. ^{1}H-NMR of **Peracetylated L-Rhamnose (1)**, $CDCl_3$.

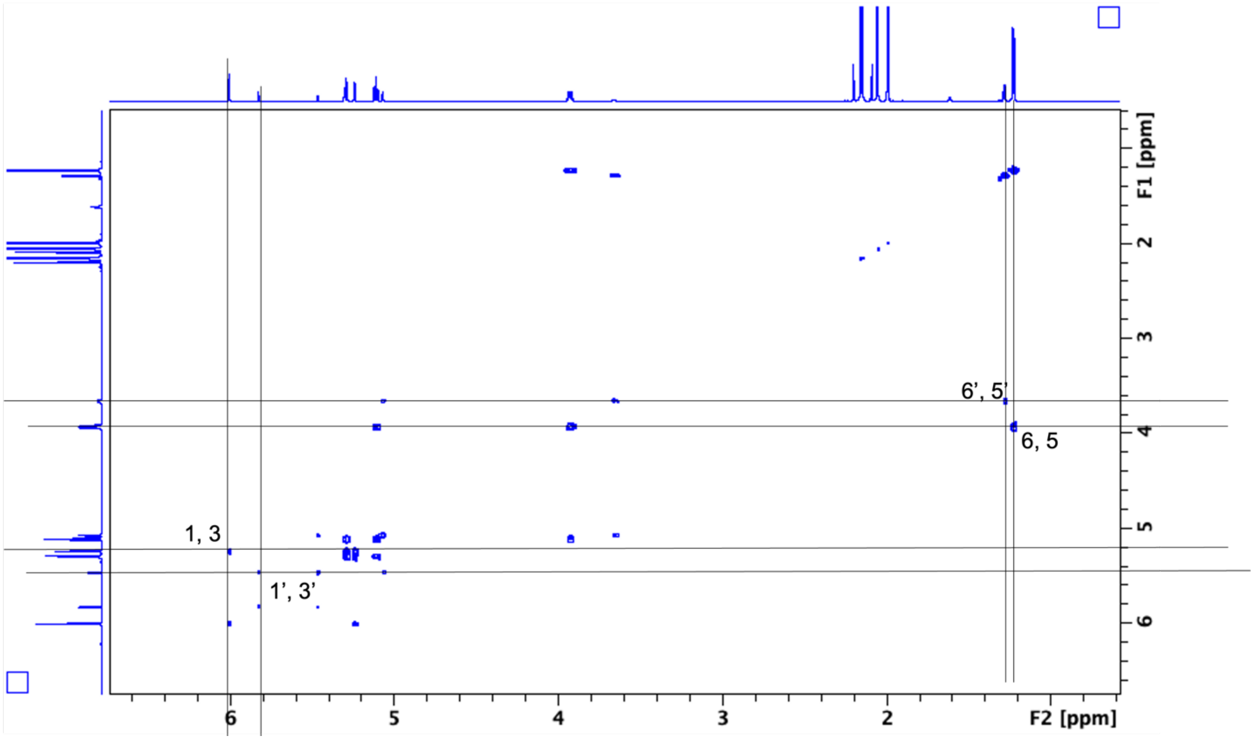

Figure S15. $^{1}H^{1}H$-COSY NMR of **Peracetylated L-Rhamnose (1)**, $CDCl_3$.

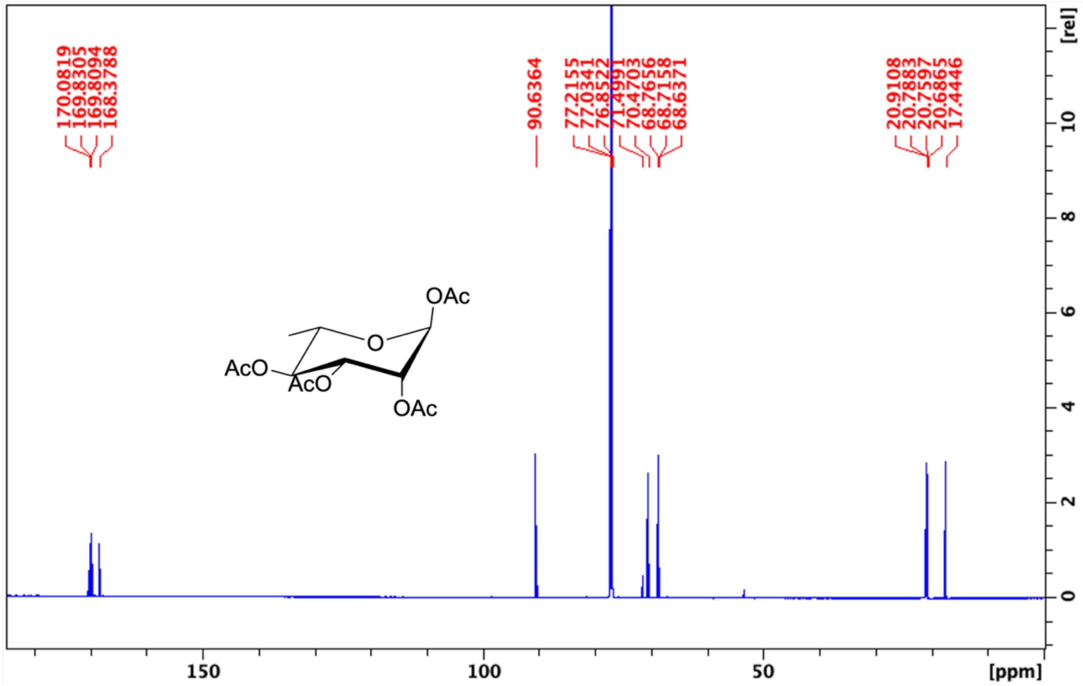

Figure S16. ^{13}C-NMR of **Peracetylated L-Rhamnose (1)**, $CDCl_3$.

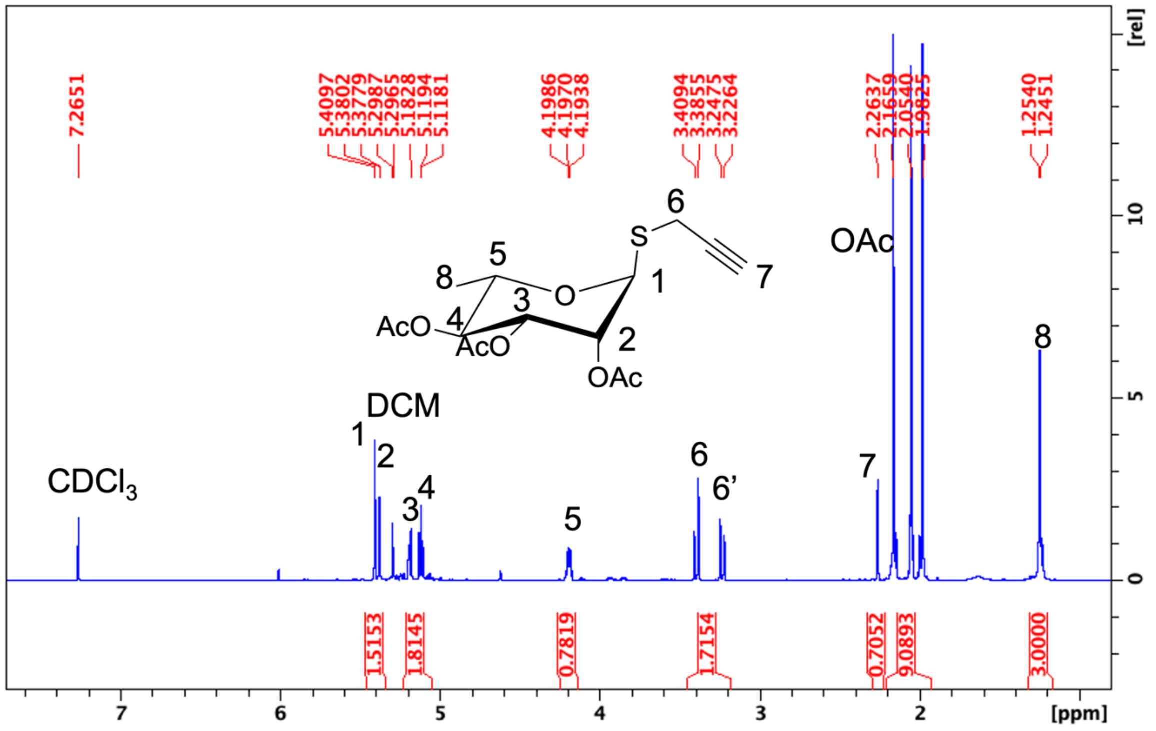

Figure S17. ^{1}H-NMR of **Peracetyl-α-S-Propargyl Rhamnose (3)**, $CDCl_3$.

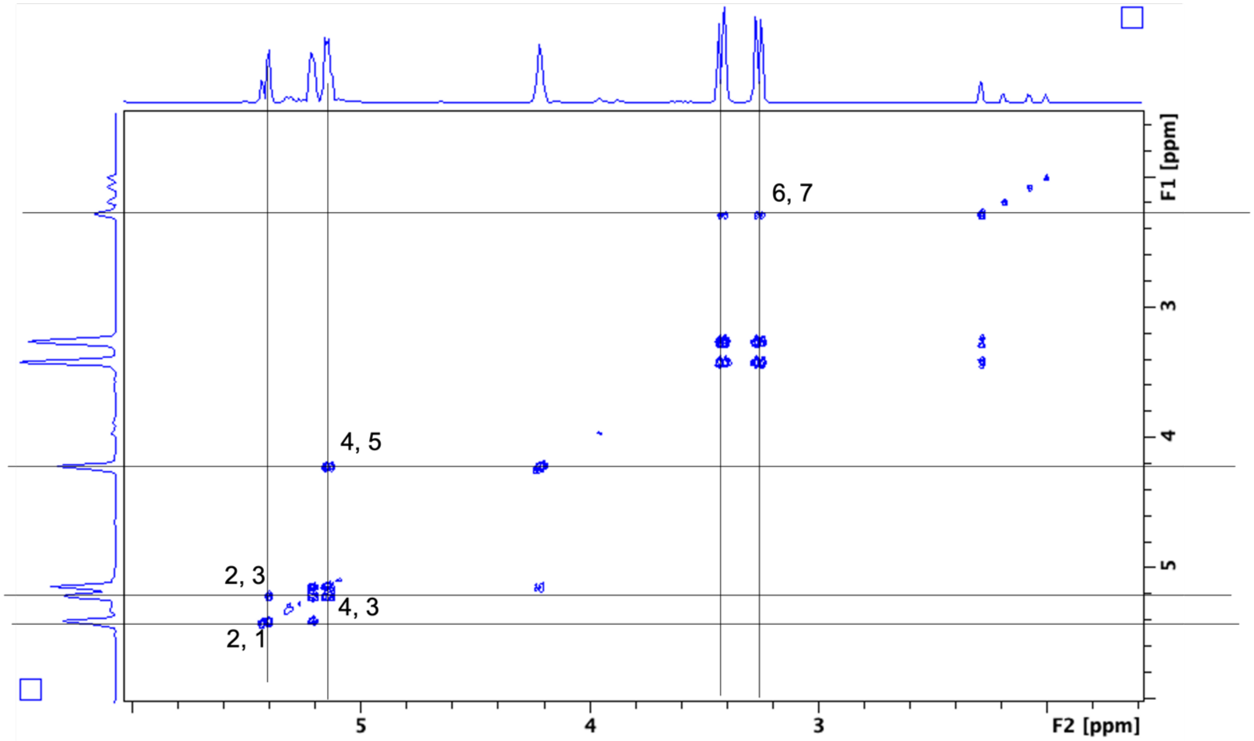

Figure S18. ^{1}H^{1}H-COSY NMR of **Peracetyl-α-S-Propargyl Rhamnose (3)**, $CDCl_3$.

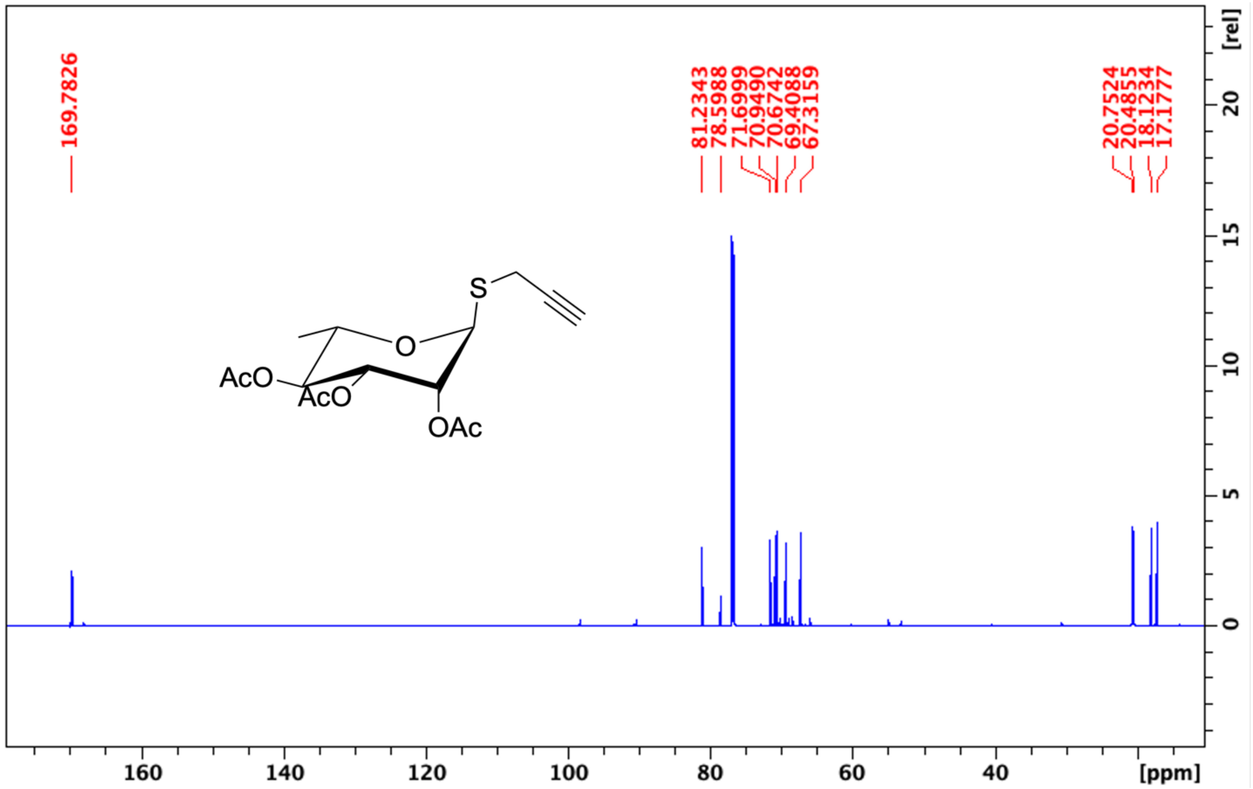

Figure S19. ^{13}C-NMR of **Peracetyl-α-S-Propargyl Rhamnose (3)**, $CDCl_3$.

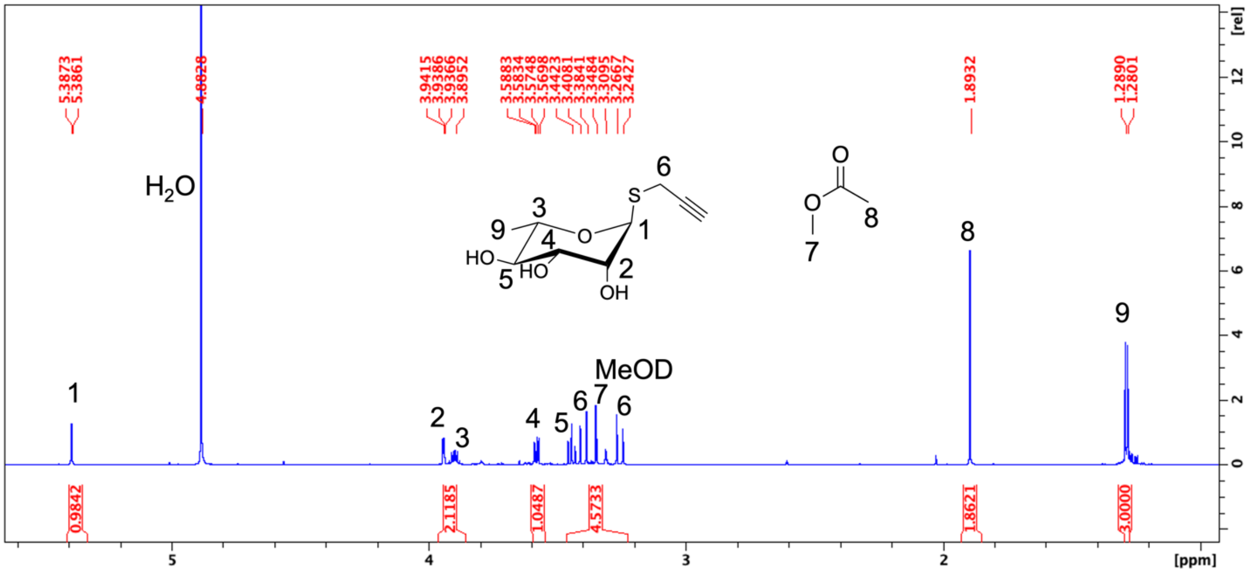

Figure S20. ^{1}H-NMR of **α-S-Propargyl Rhamnose (4)**, MeOD.

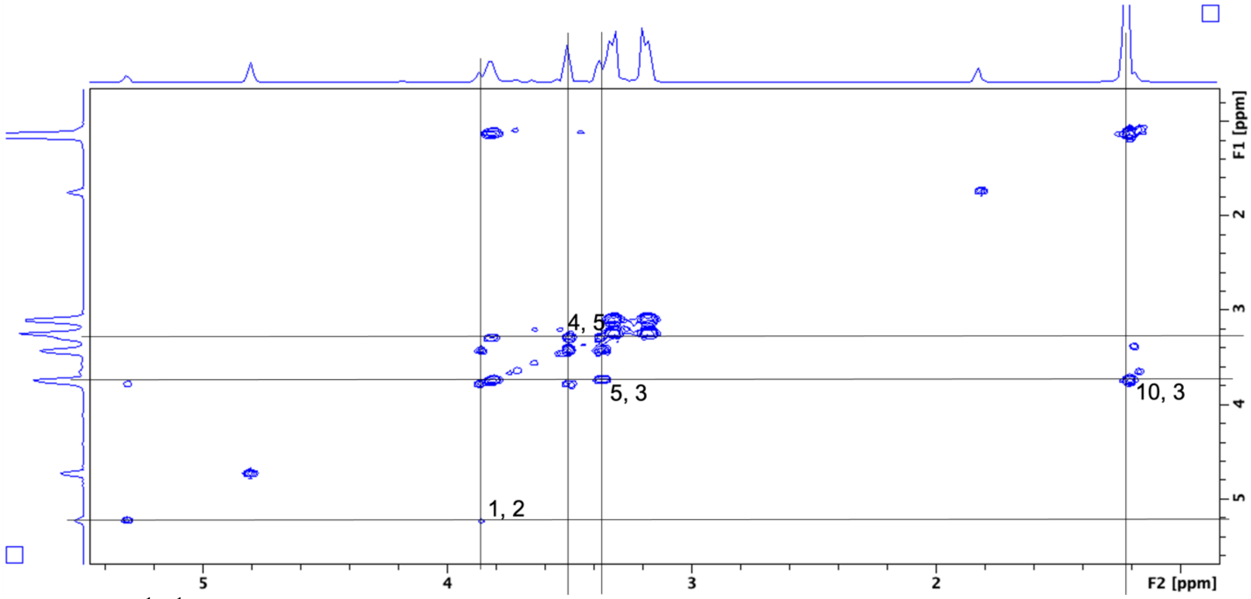

Figure S21. $^1H^1H$-COSY NMR of **α-S-Propargyl Rhamnose (4)**, MeOD.

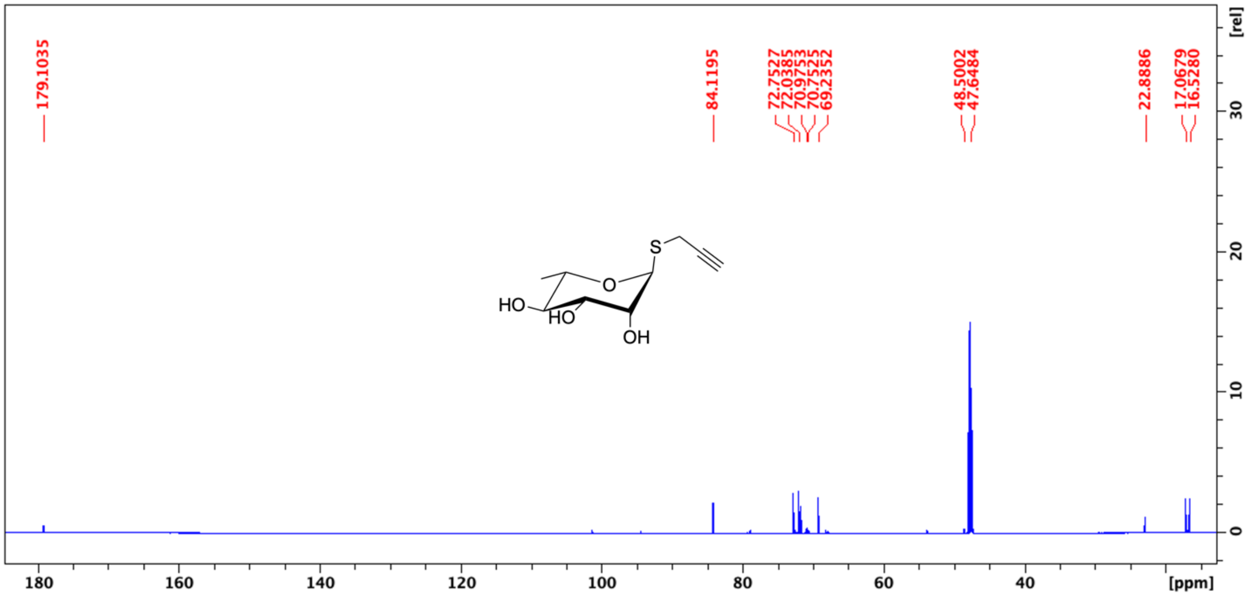

Figure S22. ^{13}C-NMR of **α_S Propargyl Rhamnose (4)**, MeOD.

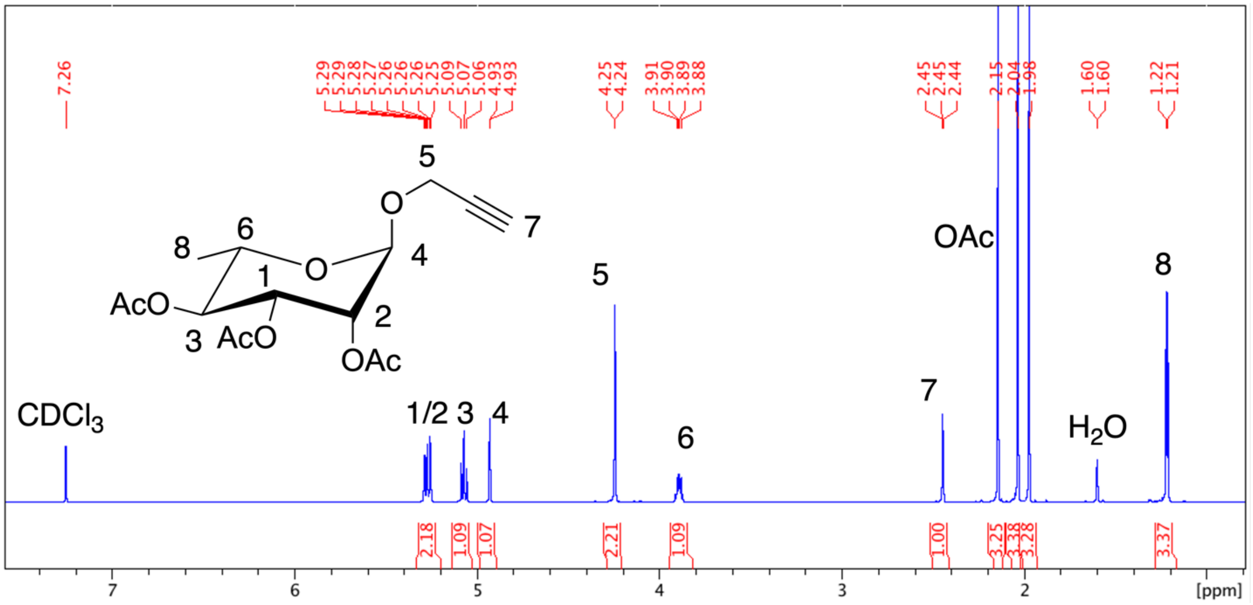

Figure S23. ^{1}H-NMR of **Peracetyl-α-O-Propargyl Rhamnose (5)**, $CDCl_3$.

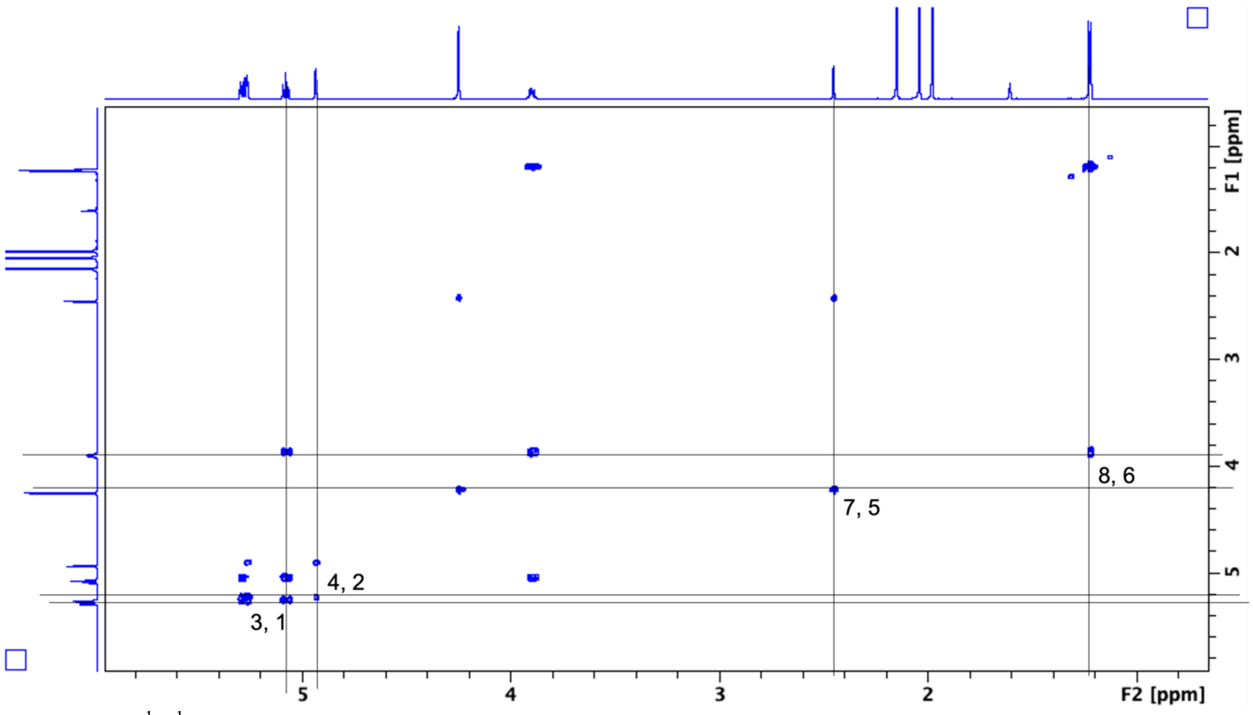

Figure S24. ^{1}H^{1}H-COSY NMR of **Peracetyl-α-O-Propargyl Rhamnose (5)**, $CDCl_3$.

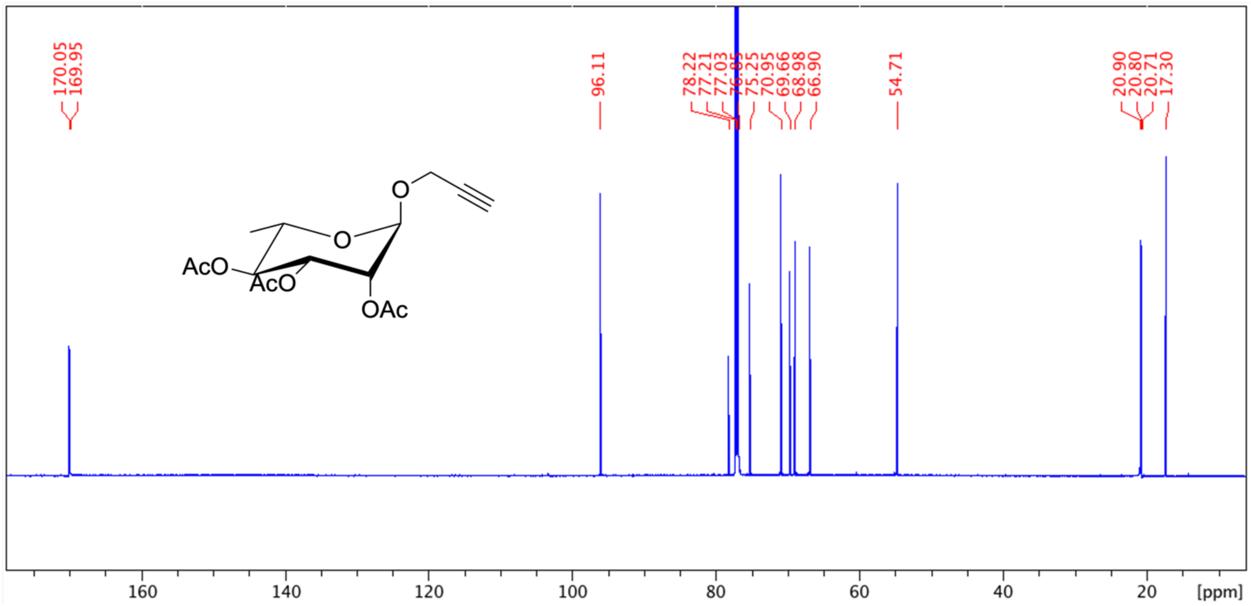

Figure S25. ^{13}C-NMR of **Peracetyl-α-O-Propargyl Rhamnose (5)**, $CDCl_3$.

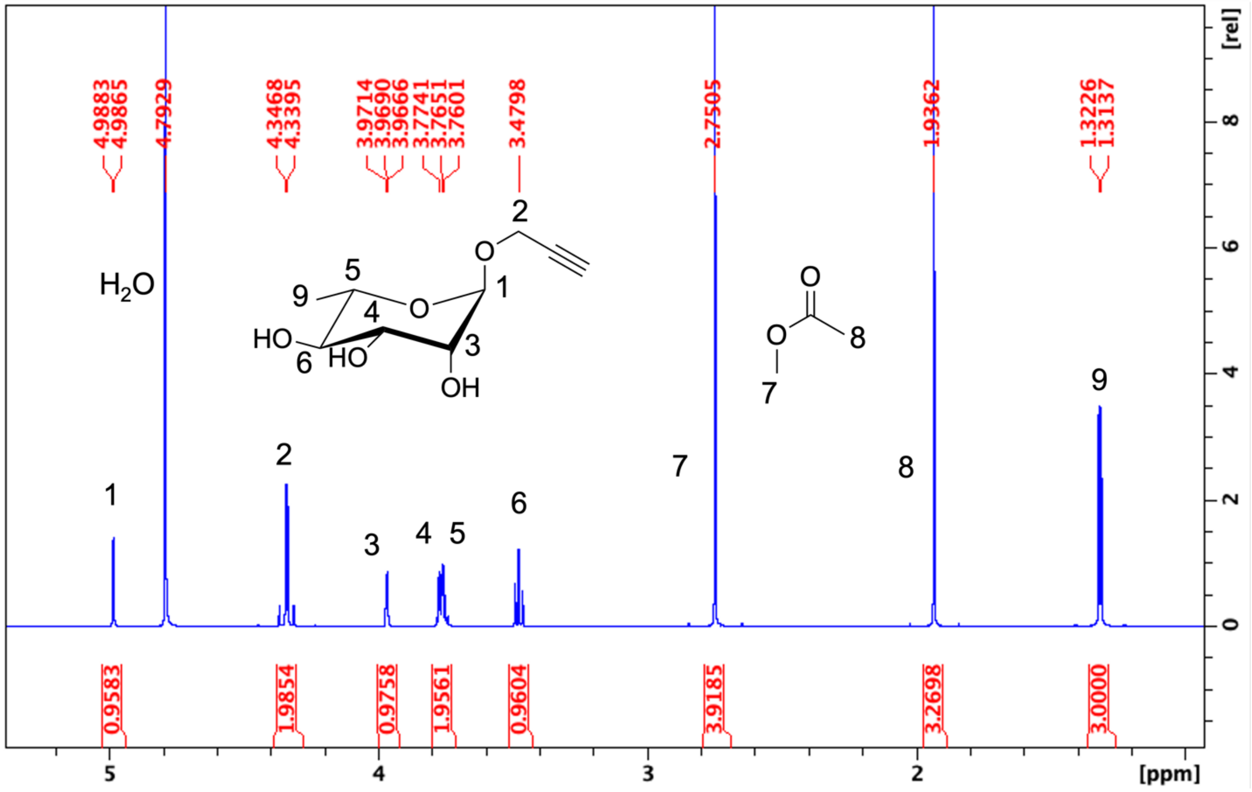

Figure S26. ^{1}H-NMR of **α-O-Propargyl Rhamnose (6)**, D_2O.

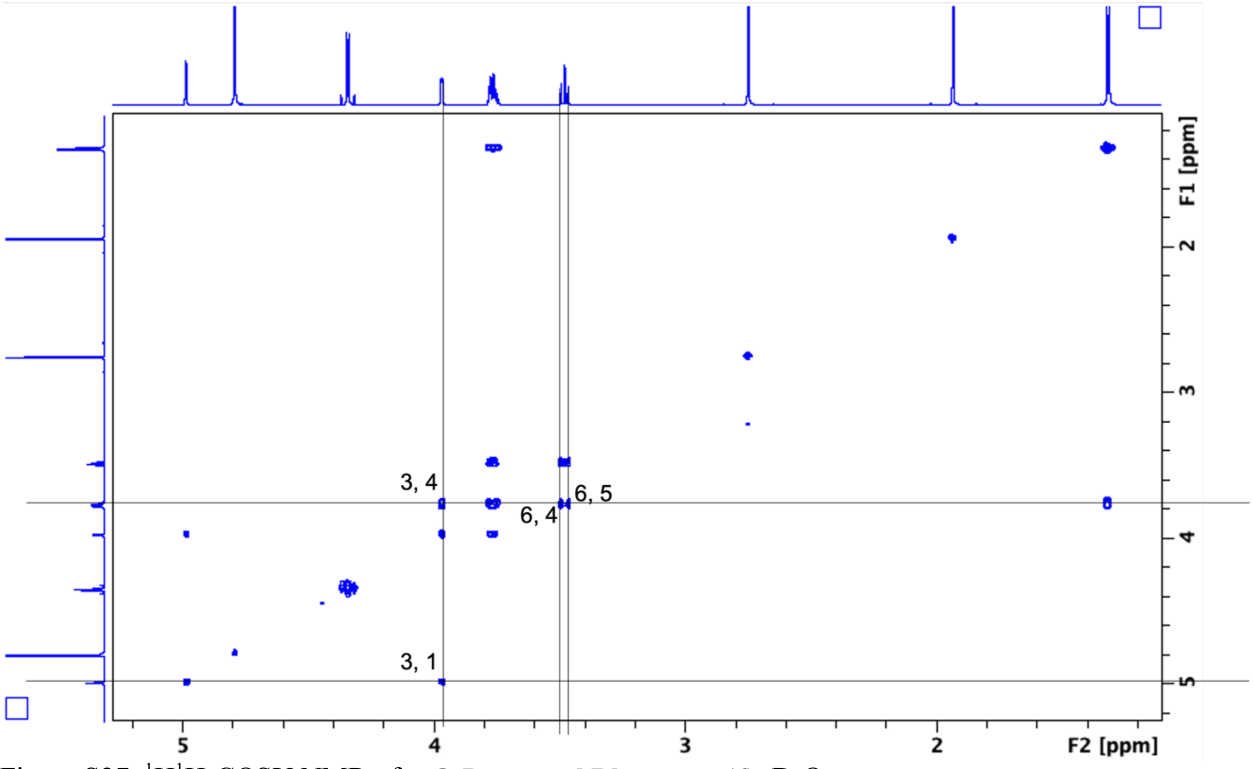

Figure S27. $^{1}H^{1}H$-COSY NMR of **α-O-Propargyl Rhamnose (6)**, D_2O.

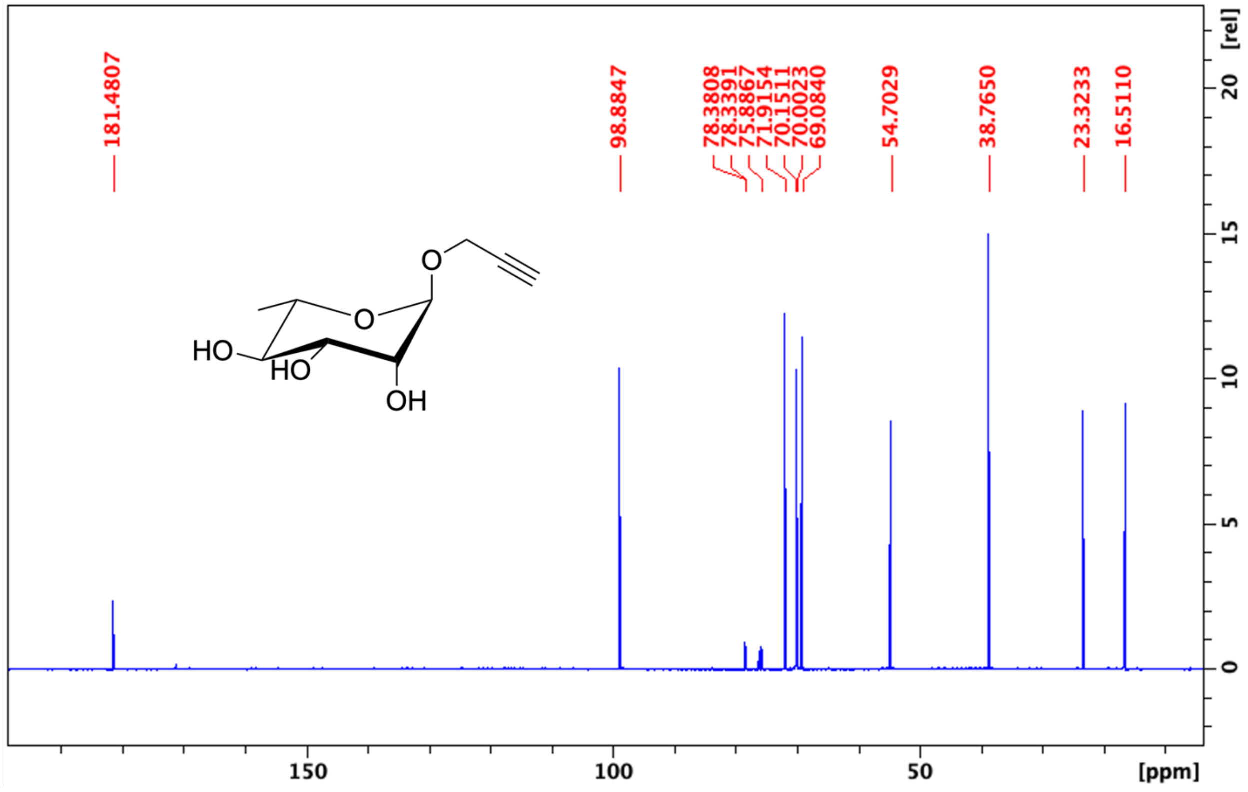

Figure S28. ^{13}C-NMR of **α-O-Propargyl Rhamnose (6)**, D_2O.

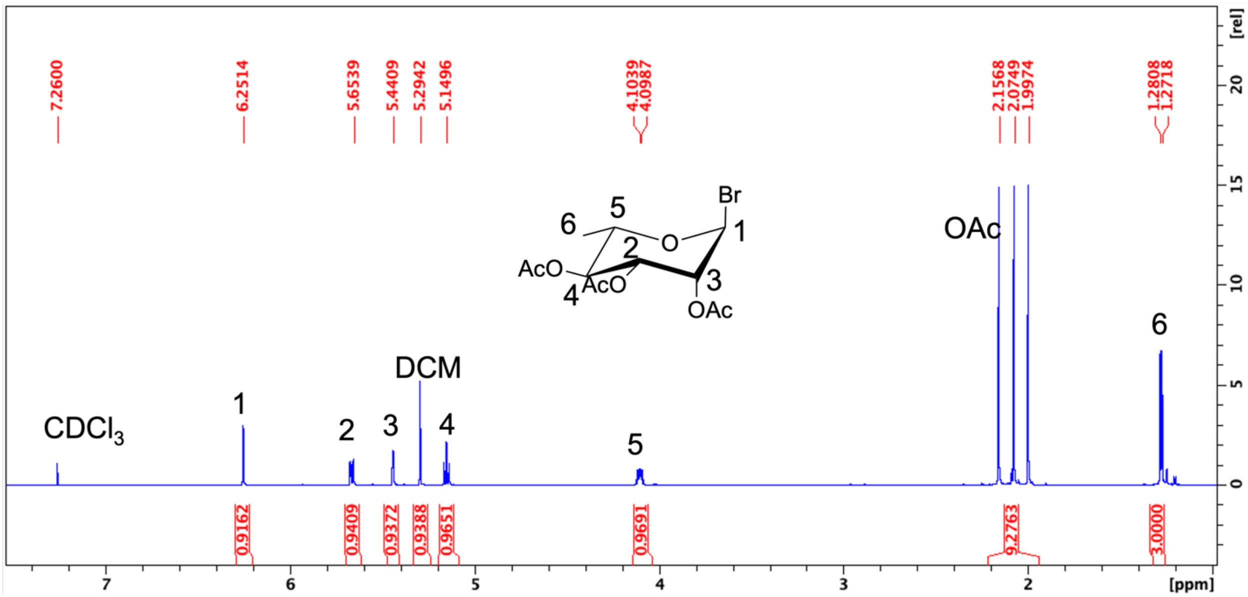

Figure S29. ^{1}H-NMR of **α-O-Bromo Rhamnose (7**), $CDCl_3$.

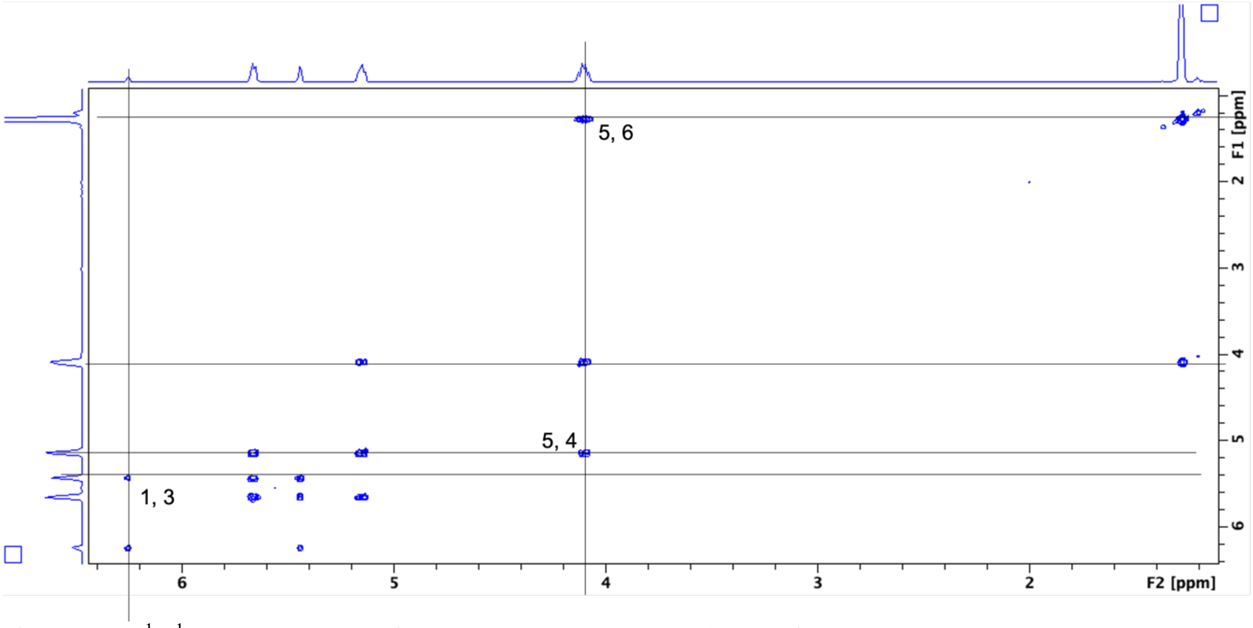

Figure S30. ^{1}H^{1}H-COSY NMR of **α-O-Bromo Rhamnose (7**), $CDCl_3$.

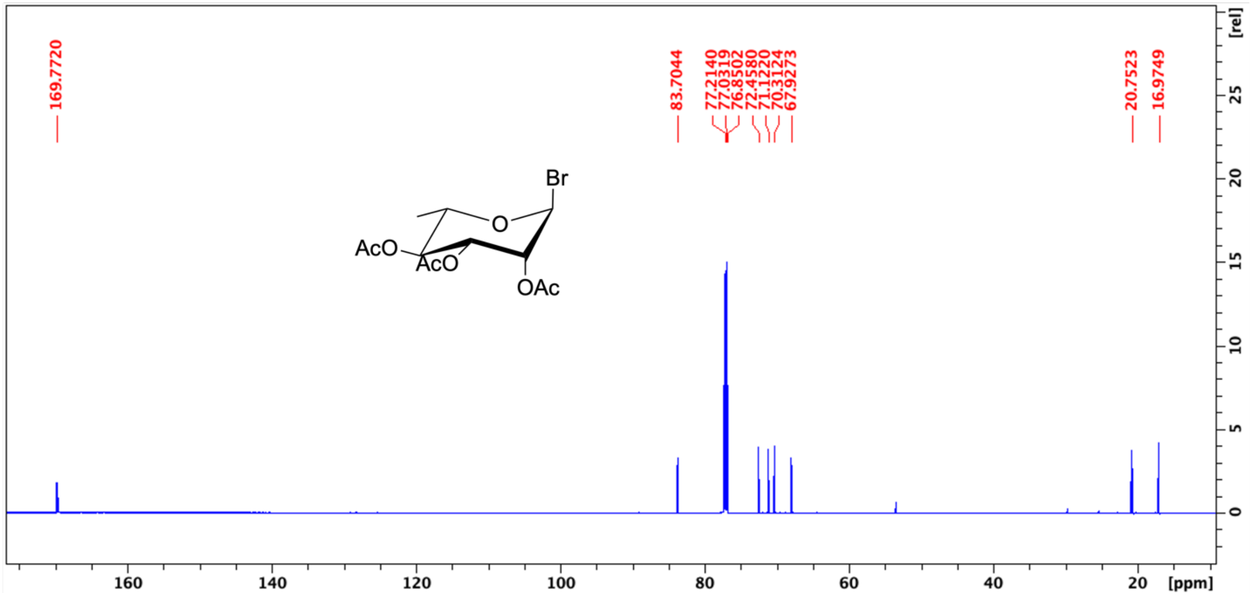

Figure S31. ^{13}C-NMR of **α-O-Bromo Rhamnose (7)**, $CDCl_3$.

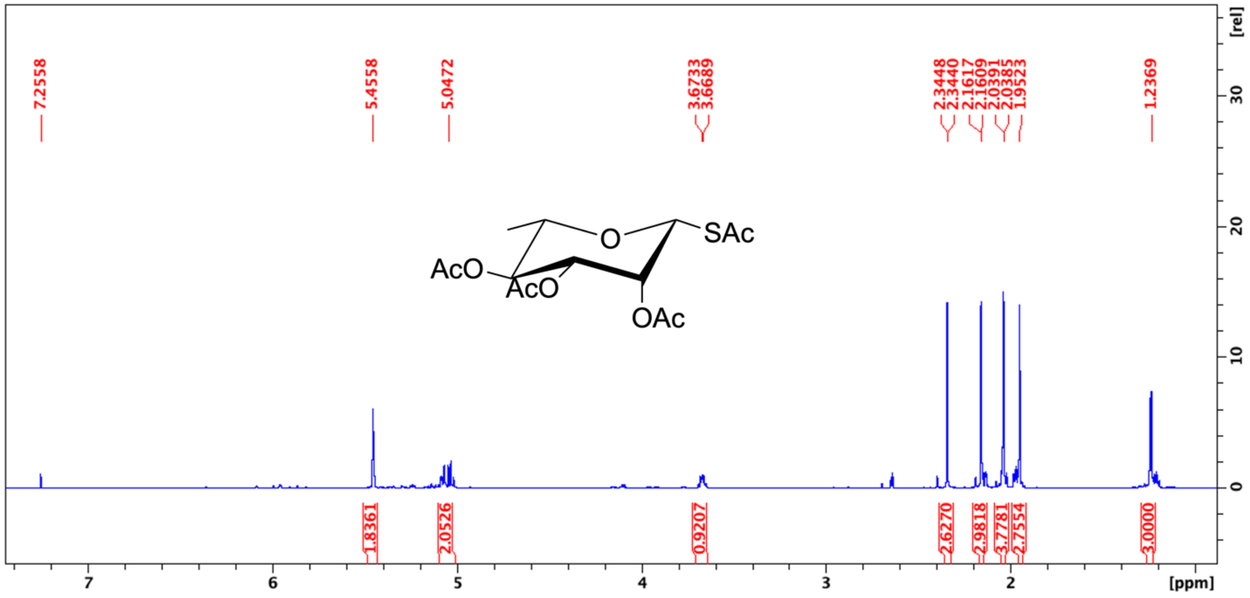

Figure S32. ^{1}H-NMR of intermediate **8**, $CDCl_3$.

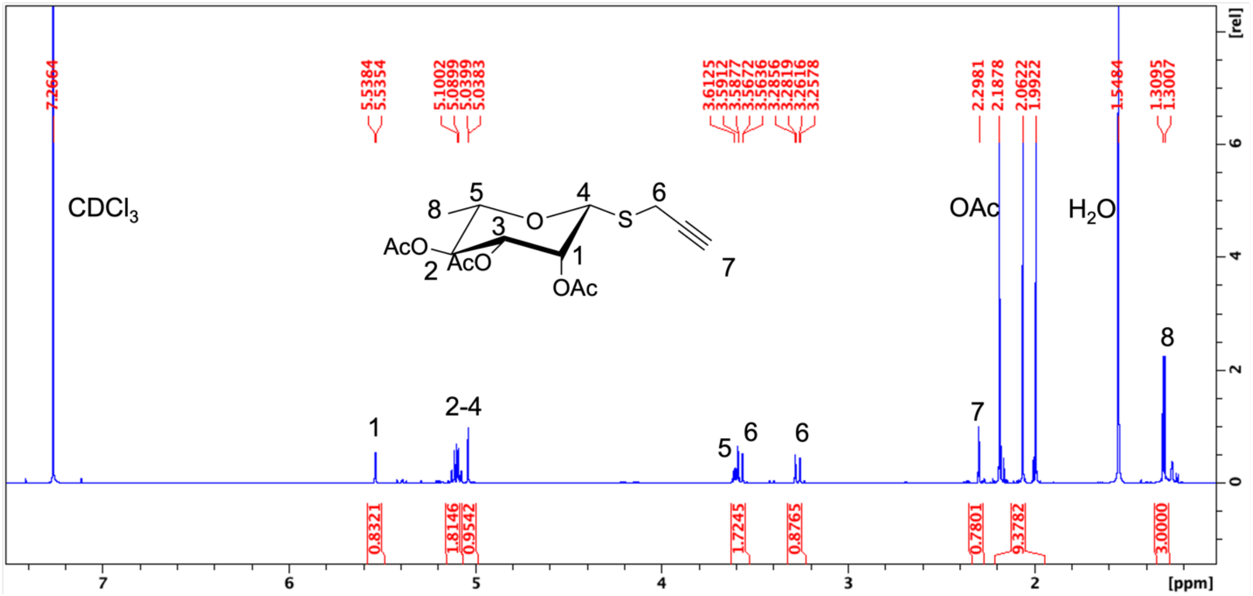

Figure S33. ^{1}H-NMR of **Peracetyl-β-S-Propargyl Rhamnose (9)**, $CDCl_3$.

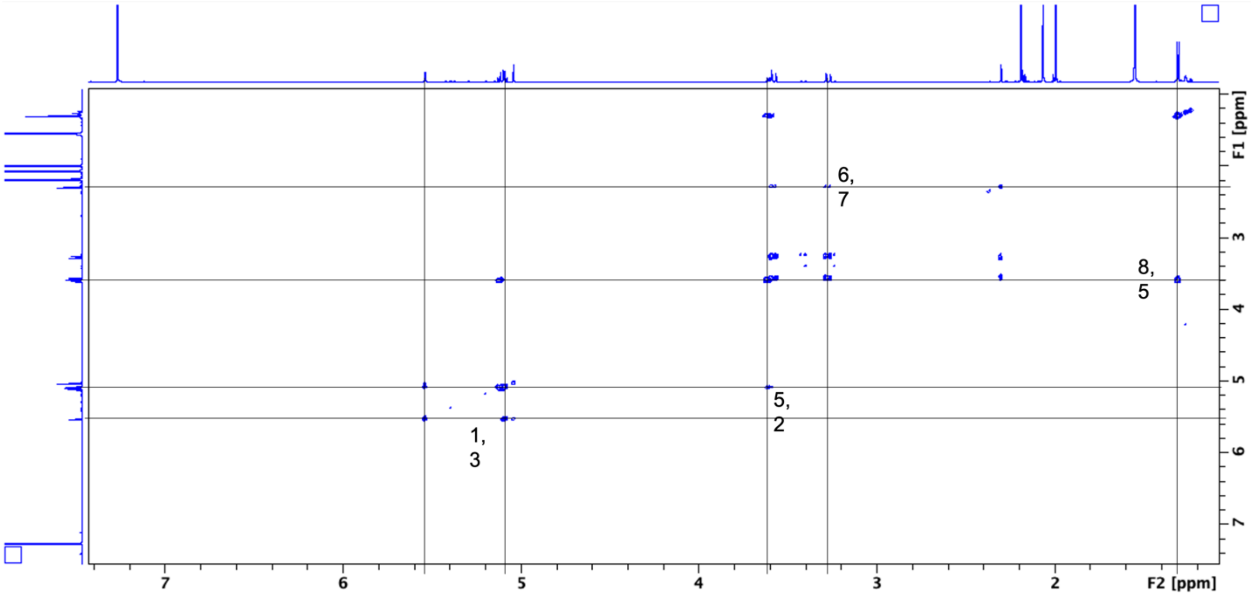

Figure S34. ^{1}H^{1}H-COSY NMR of **Peracetyl-β-S-Propargyl Rhamnose (9)**, $CDCl_3$.

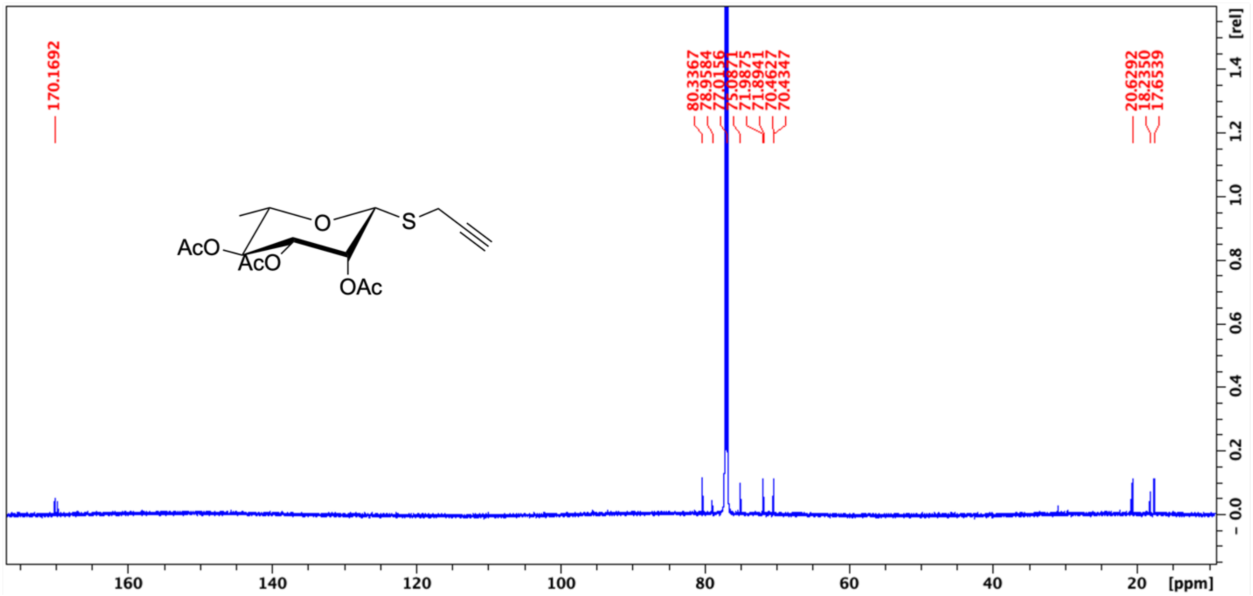

Figure S35. ^{13}C-NMR of **Peracetyl-β-S-Propargyl Rhamnose (9)**, $CDCl_3$.

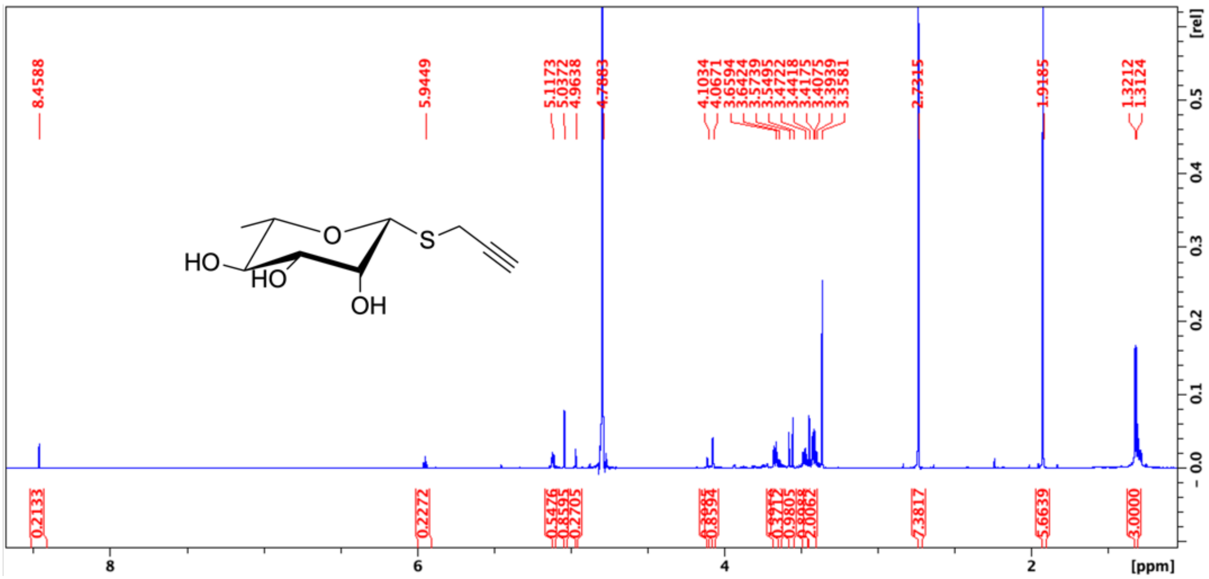

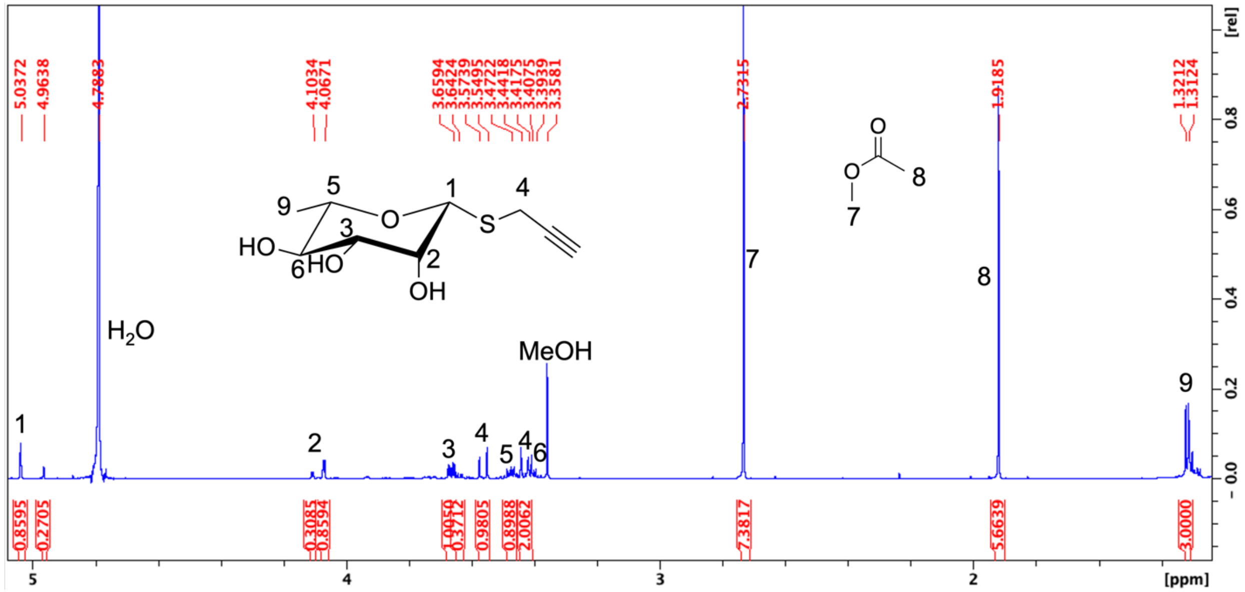

Figure S36. ^{1}H-NMR of **β-S-Propargyl Rhamnose (10)**, D_2O. Top is full spectrum. Bottom highlights product.

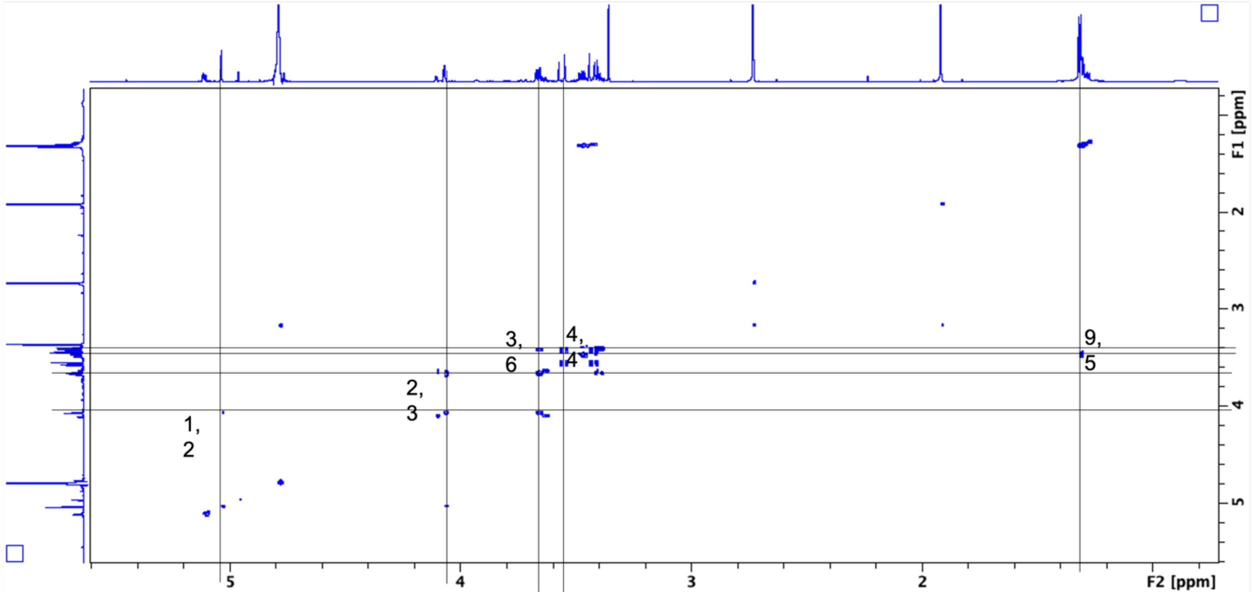

Figure S37. ^{1}H^{1}H-COSY NMR of **β-S-Propargyl Rhamnose (10)**, D_2O.

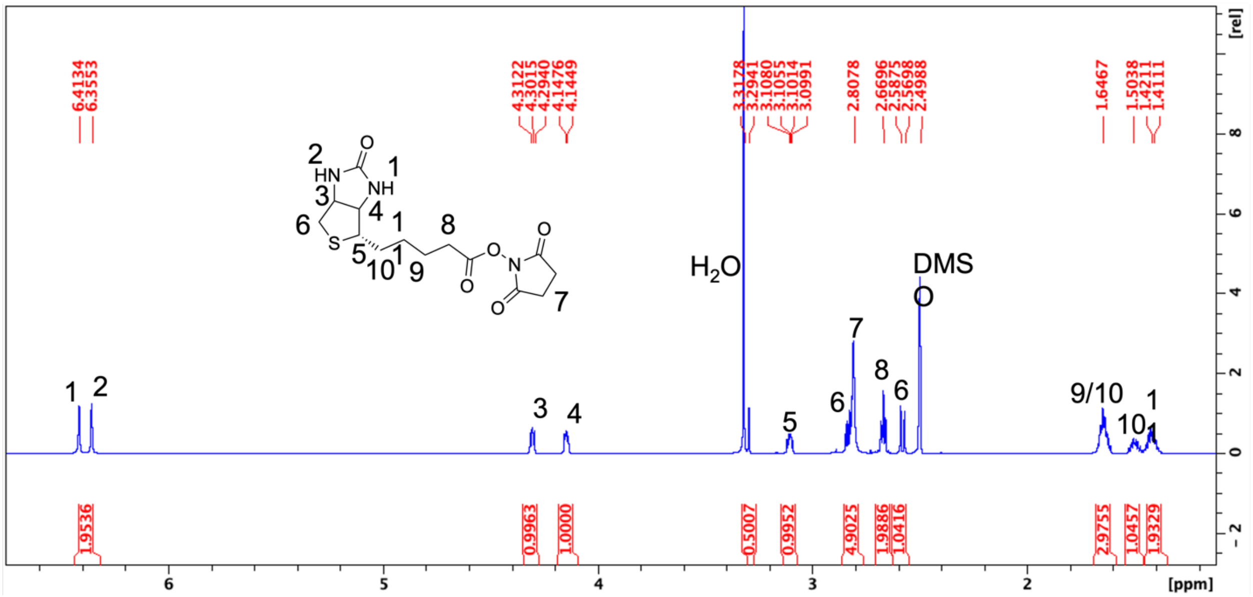

Figure S38. ^{1}H-NMR of **Biotin-NHS (12)**, DMSO.

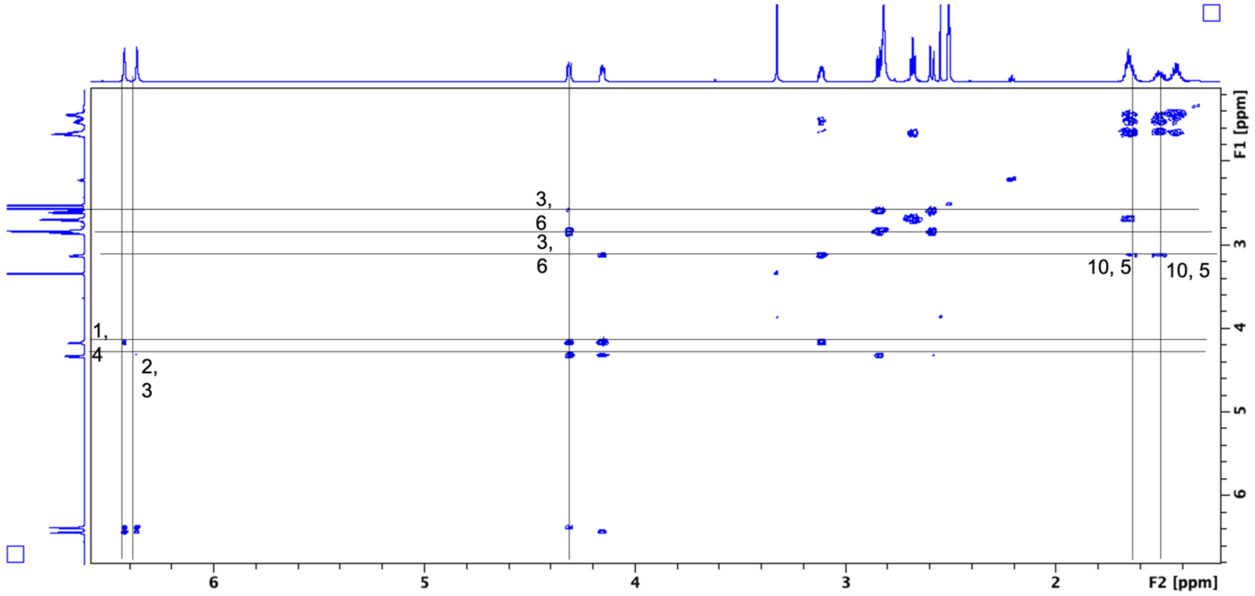

Figure S39. ^{1}H^{1}H-COSY NMR of **Biotin-NHS (12)**, DMSO.

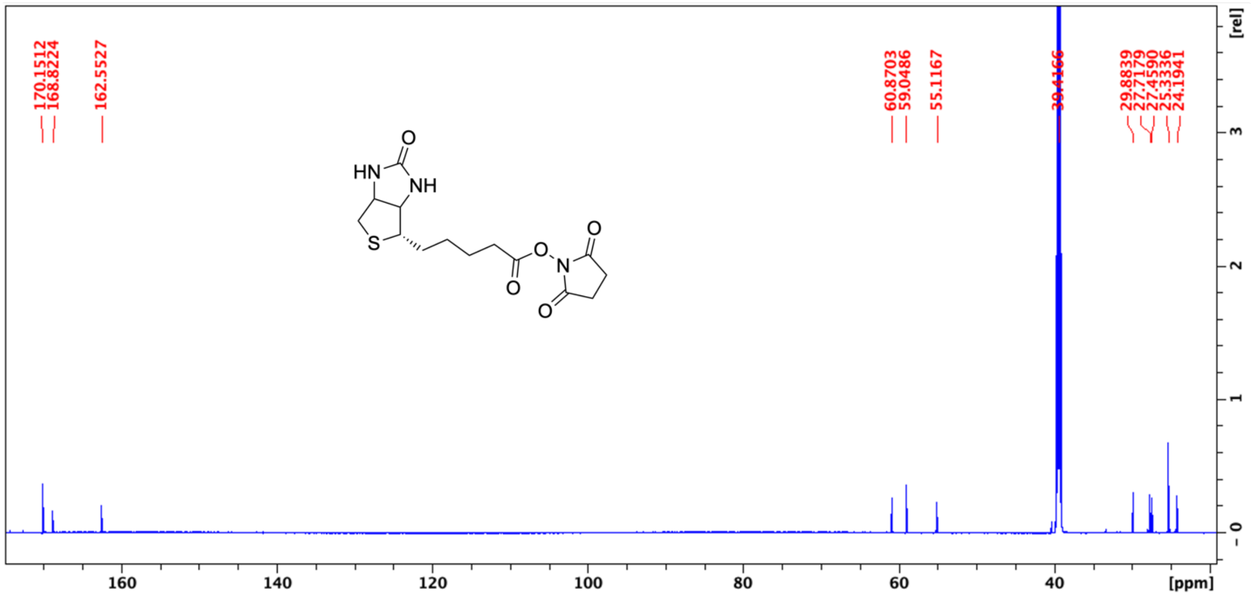

Figure S40. ^{13}C-NMR of **Biotin-NHS (12)**, DMSO.

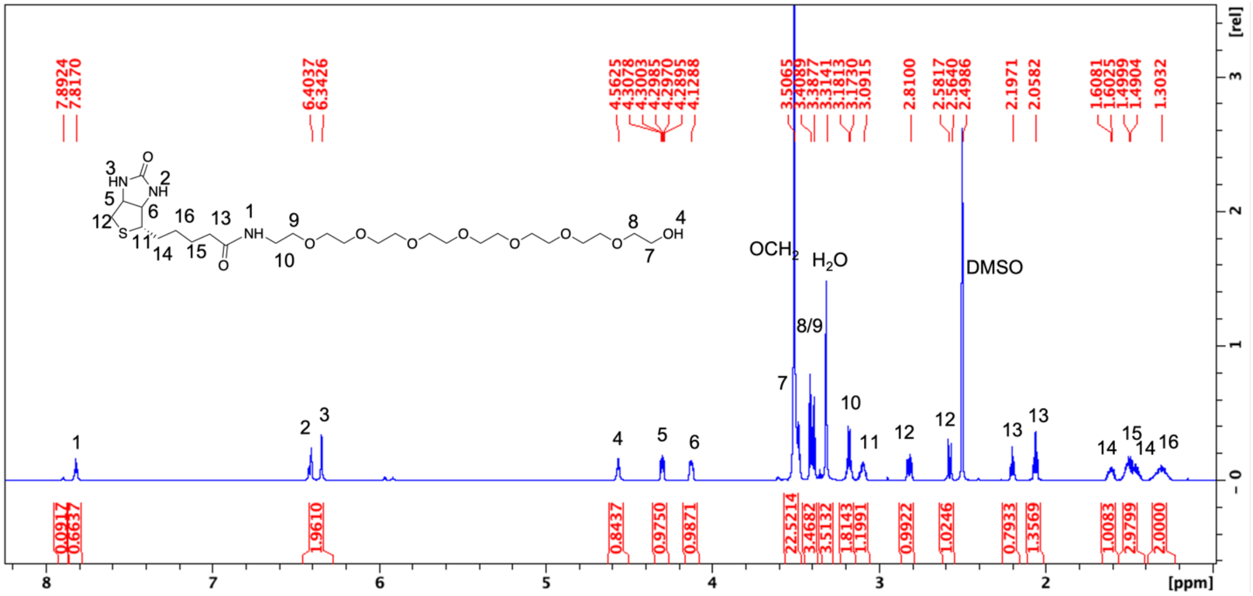

Figure S41. ^{1}H-NMR of **Biotin-Peg8-Hydroxy (13)**, DMSO.

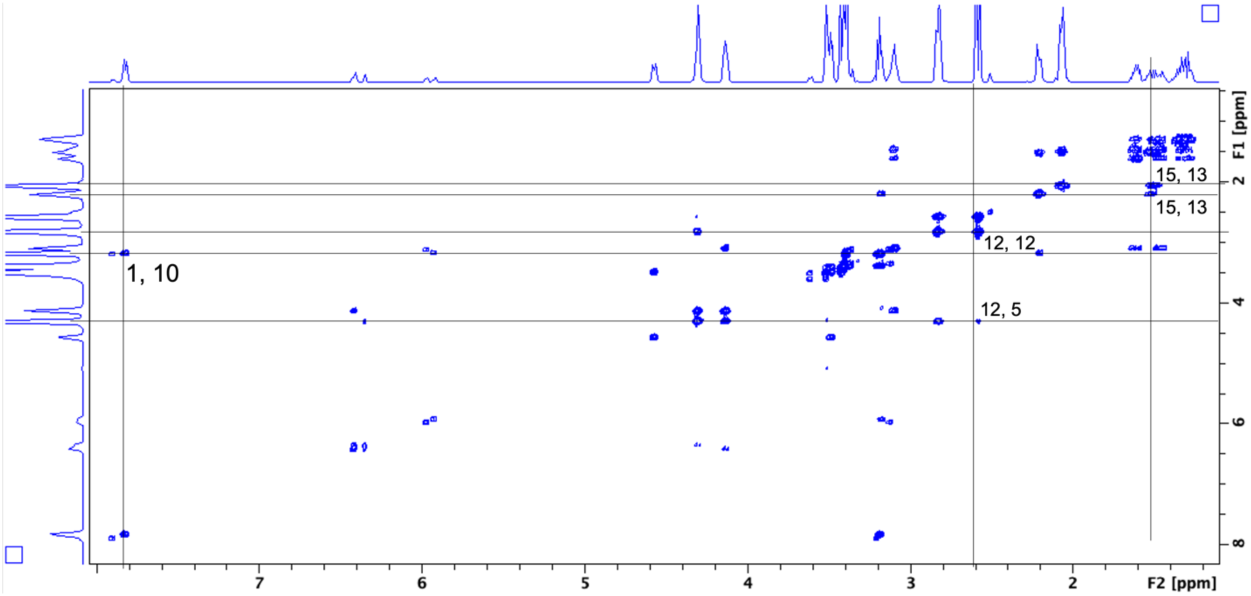

Figure S42. ¹H¹H-COSY NMR of **Biotin-Peg8-Hydroxy (13)**, DMSO.

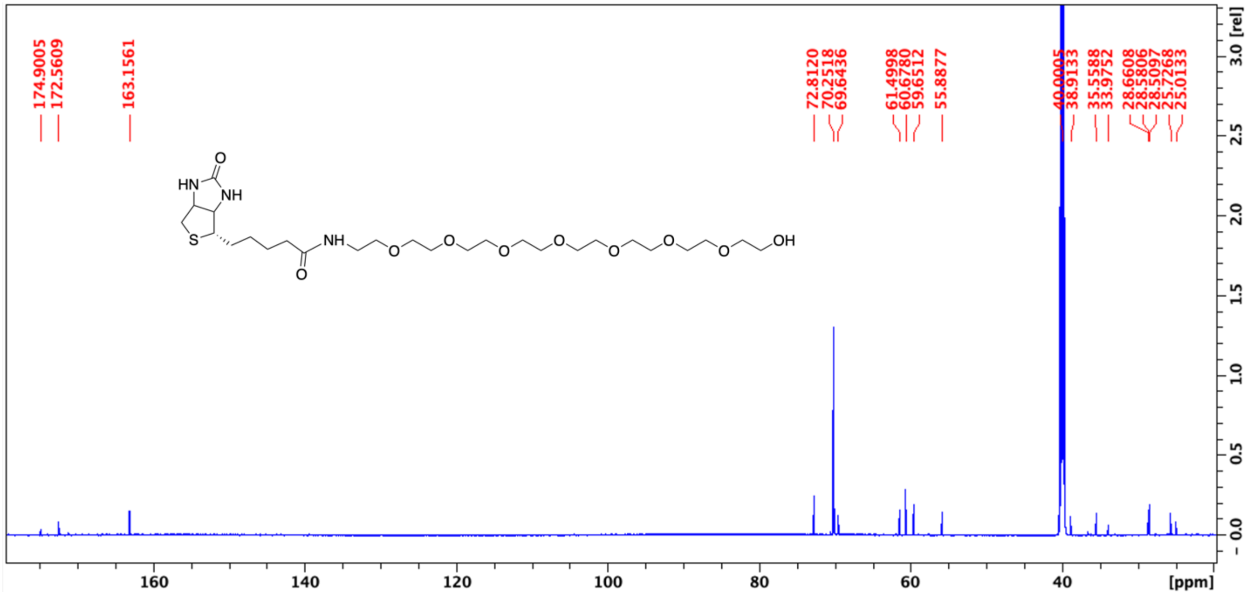

Figure S43. ¹³C-NMR of **Biotin-Peg8-Hydroxy (13)**, DMSO.

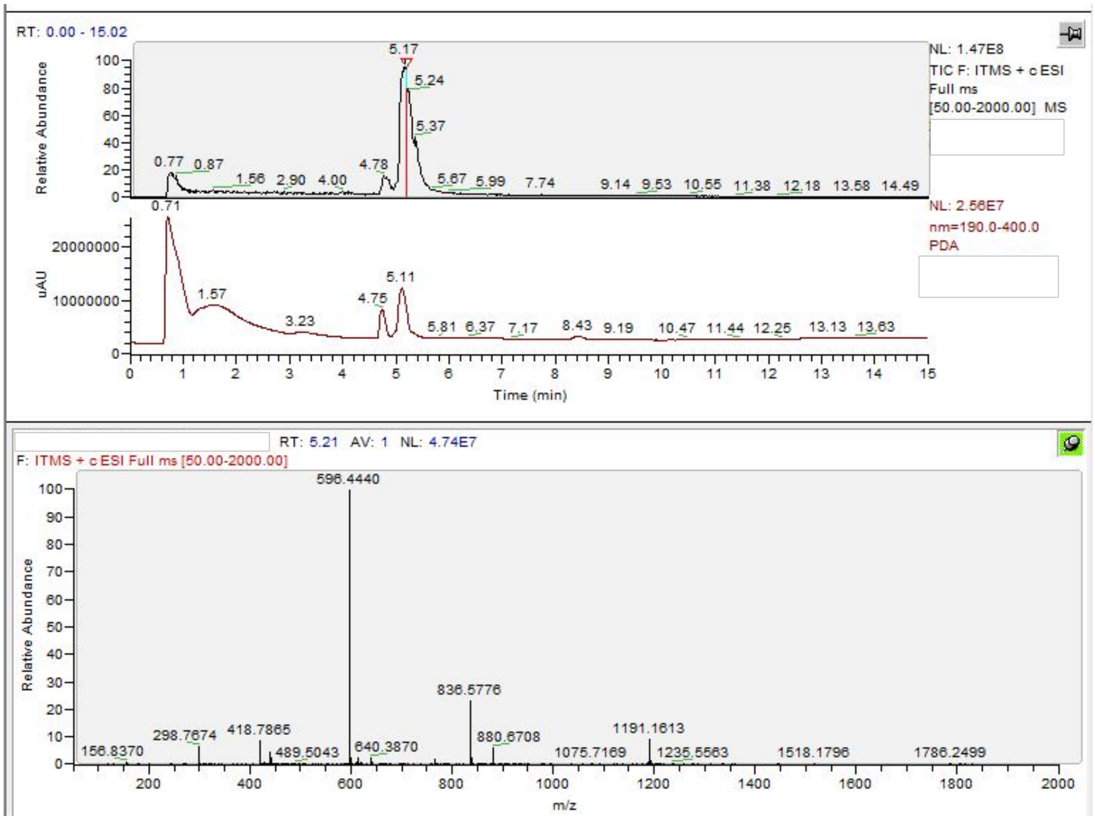

Figure S44. LC-MS trace of post column purified compound **Biotin-Peg8-Hydroxy (13)**. Top - TIC trace. Bottom - selected mass spectrum from TIC highlighted by red bar (retention time 5.21). Middle - UV chromatogram. A slight excess of biotin is present at retention time 4.78 (TIC).

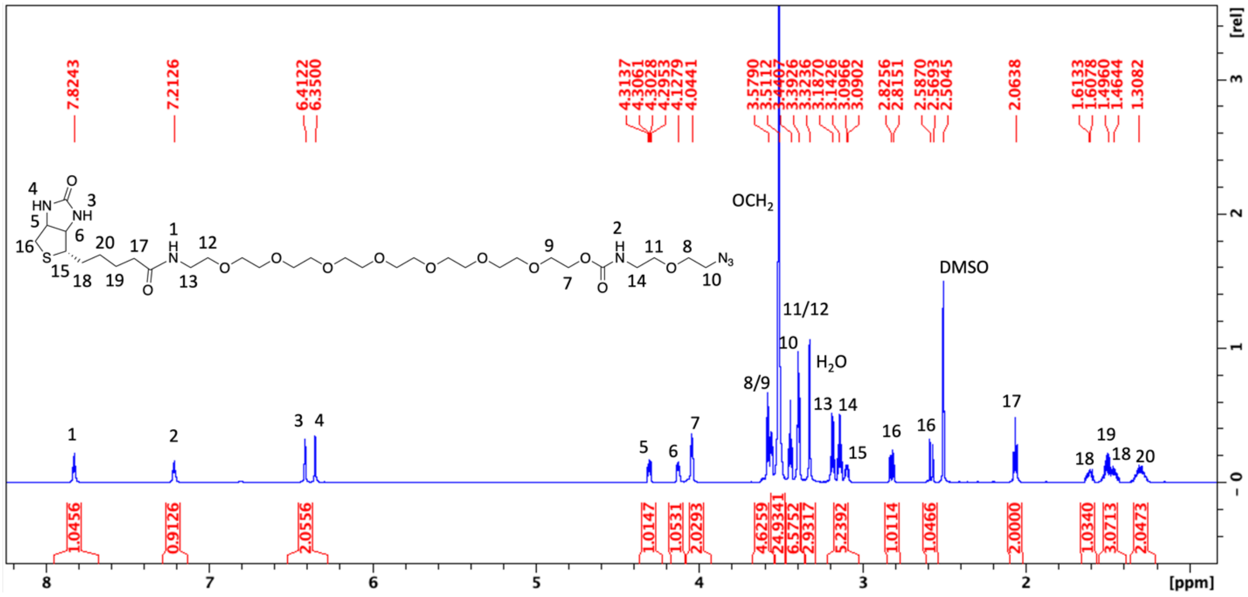

Figure S45. ^{1}H-NMR of **Biotin-Peg8-Peg1-Azide (14)**, DMSO.

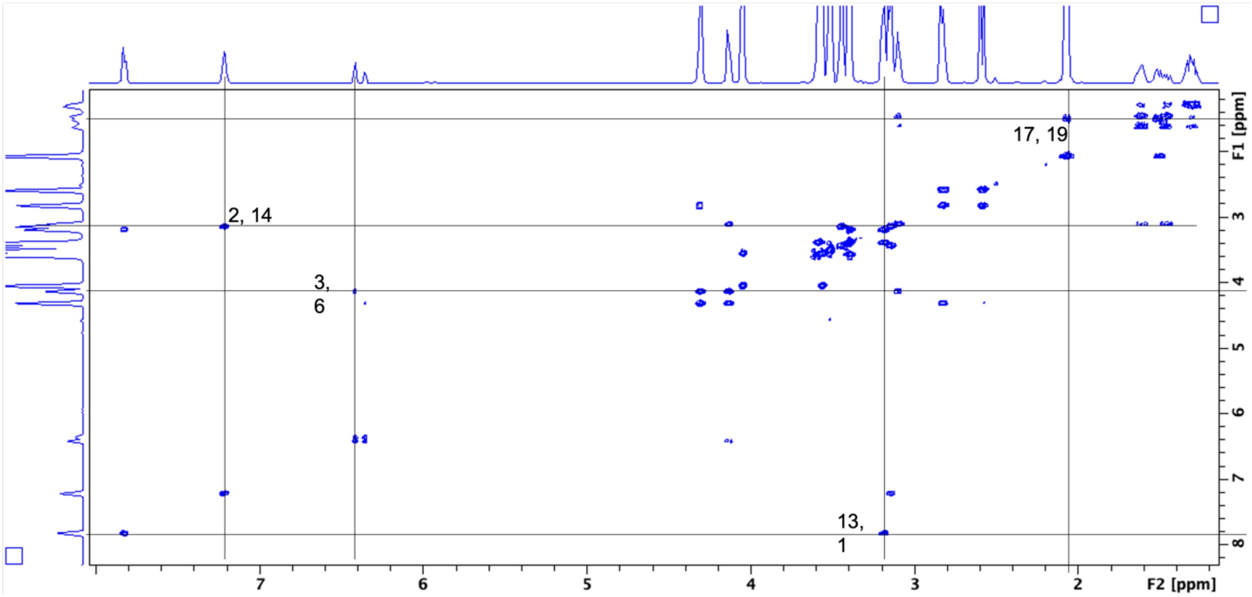

Figure S46. ^{1}H^{1}H-COSY NMR of **Biotin-Peg8-Peg1-Azide (14)**, DMSO.

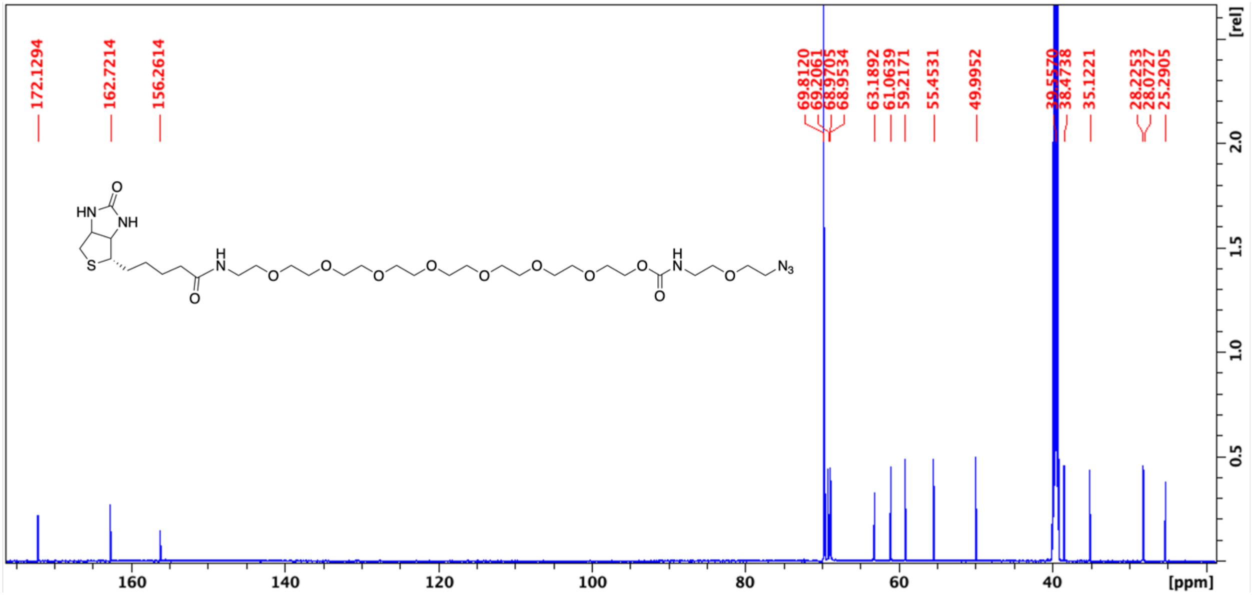

Figure S47. ^{13}C-NMR of **Biotin-Peg8-Peg1-Azide (14)**, DMSO.

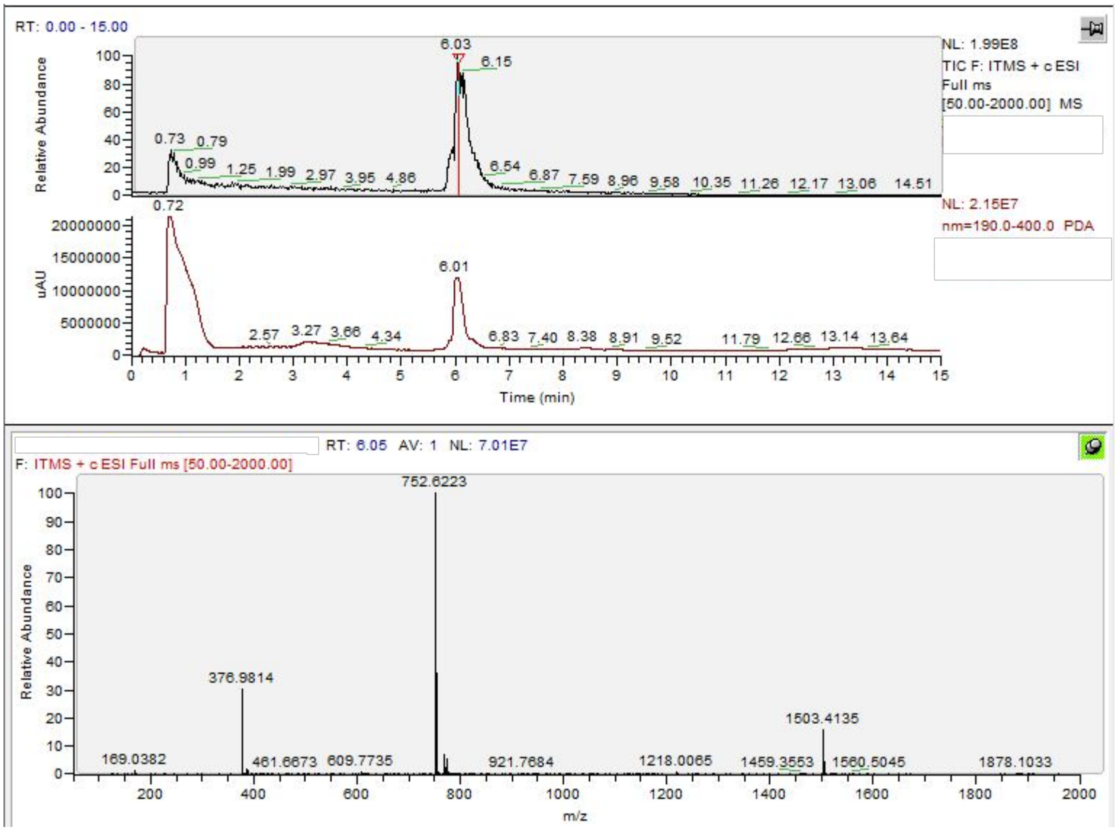

Figure S48. LC-MS trace of post column purified compound **Biotin-Peg8-Peg1-Azide (14)**. Top - TIC trace. Bottom - selected mass spectrum from TIC highlighted by red bar (retention time 6.05). Middle - UV chromatogram.

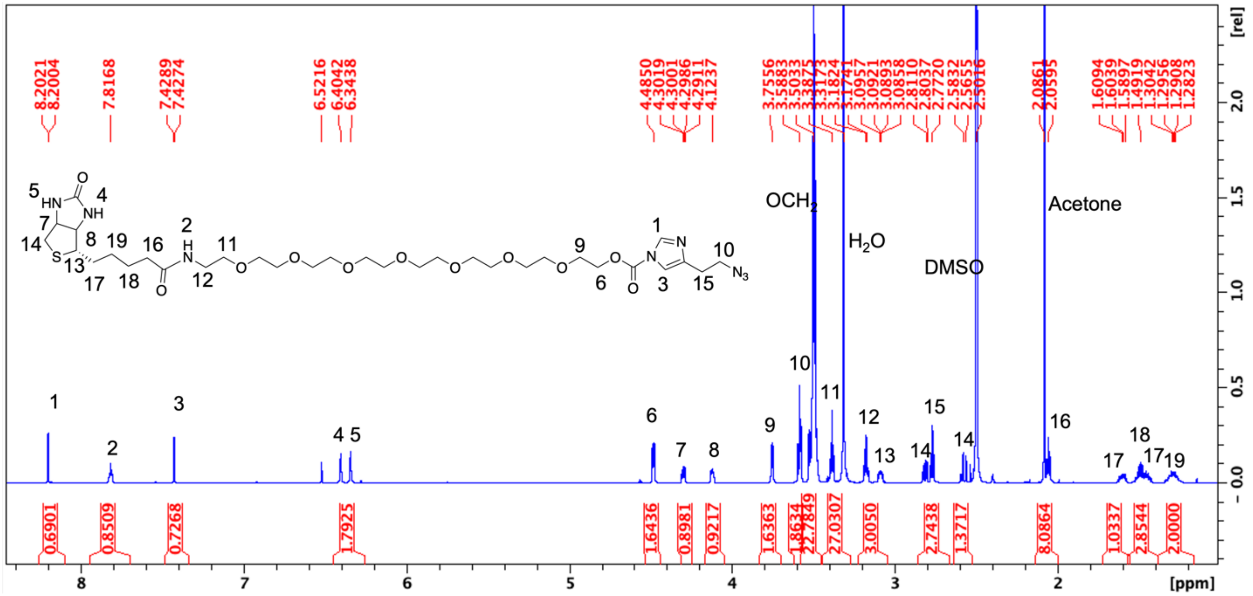

Figure S49. ^{1}H-NMR of **Biotin-Peg8-AI-Azide (15)**, DMSO.

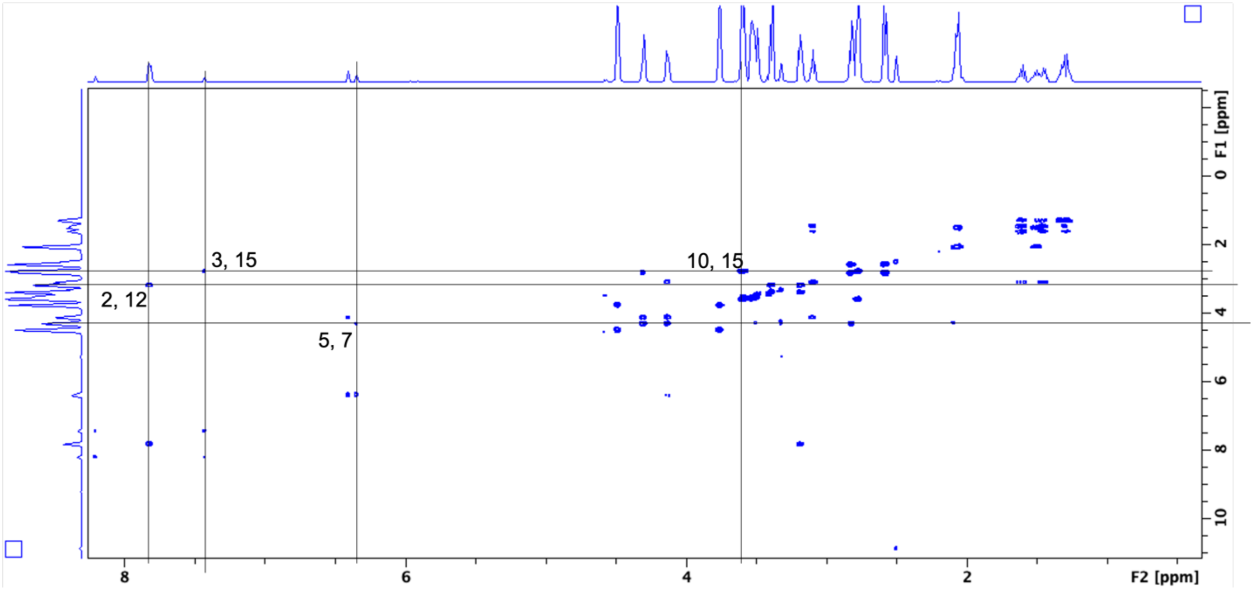

Figure S50. $^{1}H^{1}H$-COSY NMR of **Biotin-Peg8-AI-Azide (15)**, DMSO.

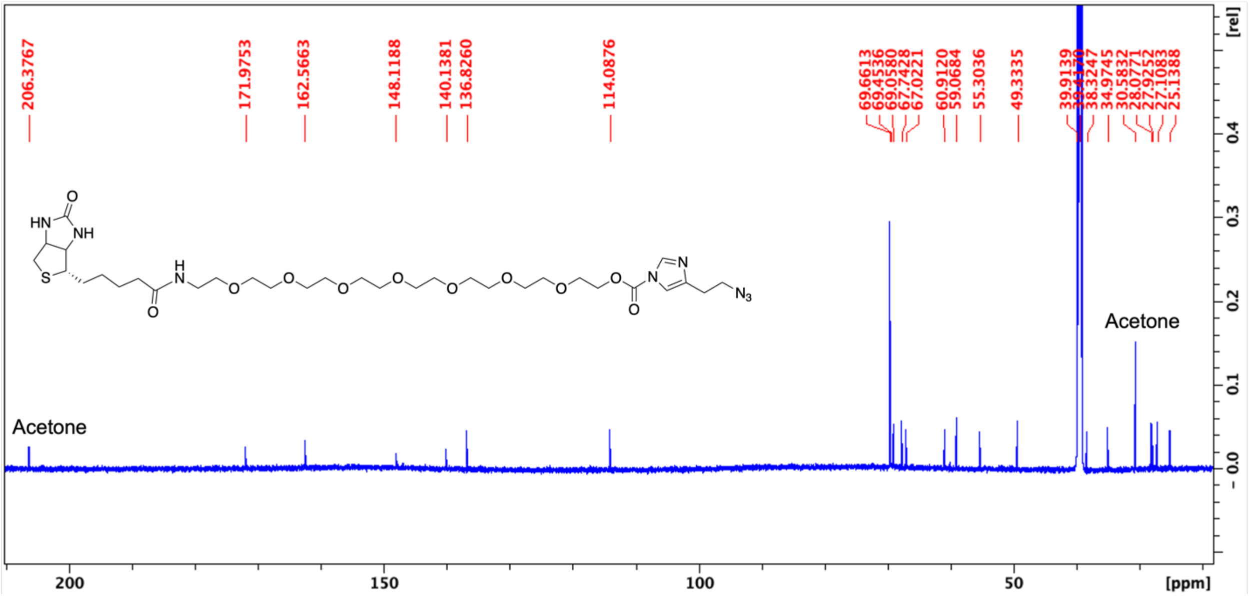

Figure S51. ^{13}C-NMR of **Biotin-Peg8-AI-Azide (15)**, DMSO.

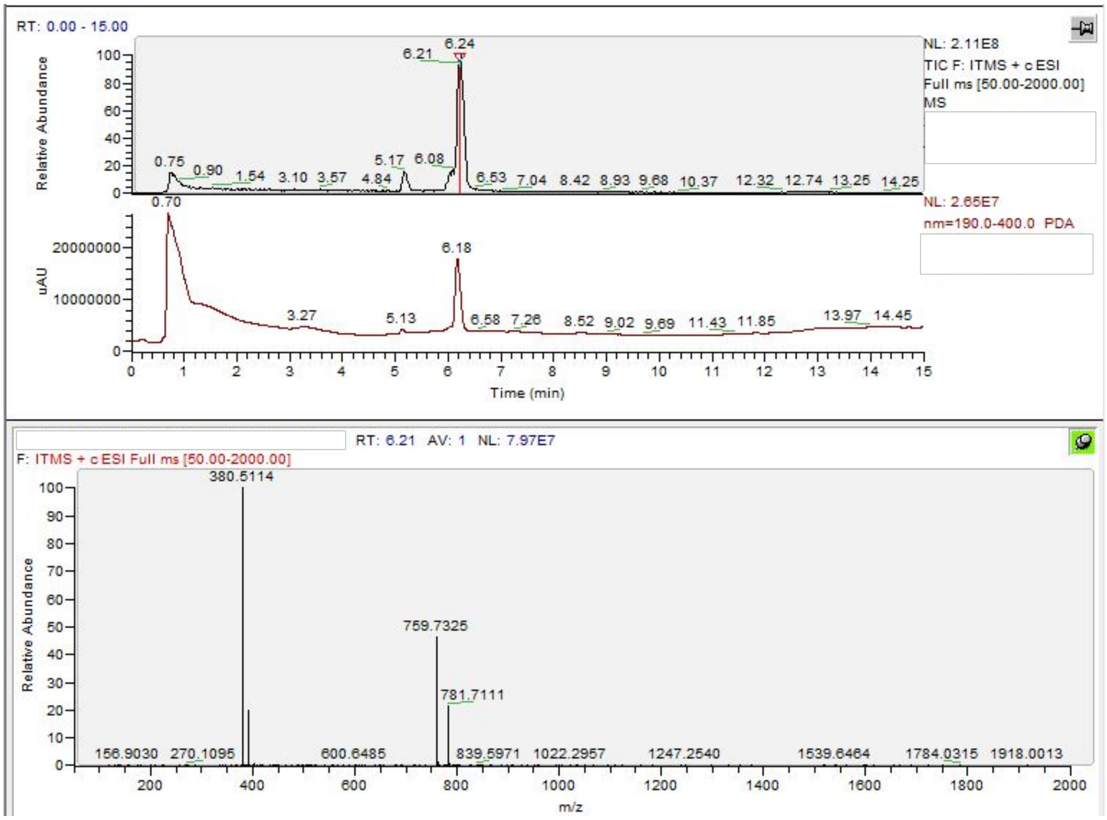

Figure S52. LC-MS trace of post column purified compound **Biotin-Peg8-AI-Azide (15)**. Top - TIC trace. Bottom - selected mass spectrum from TIC highlighted by red bar (retention time 6.21). Middle - UV chromatogram.

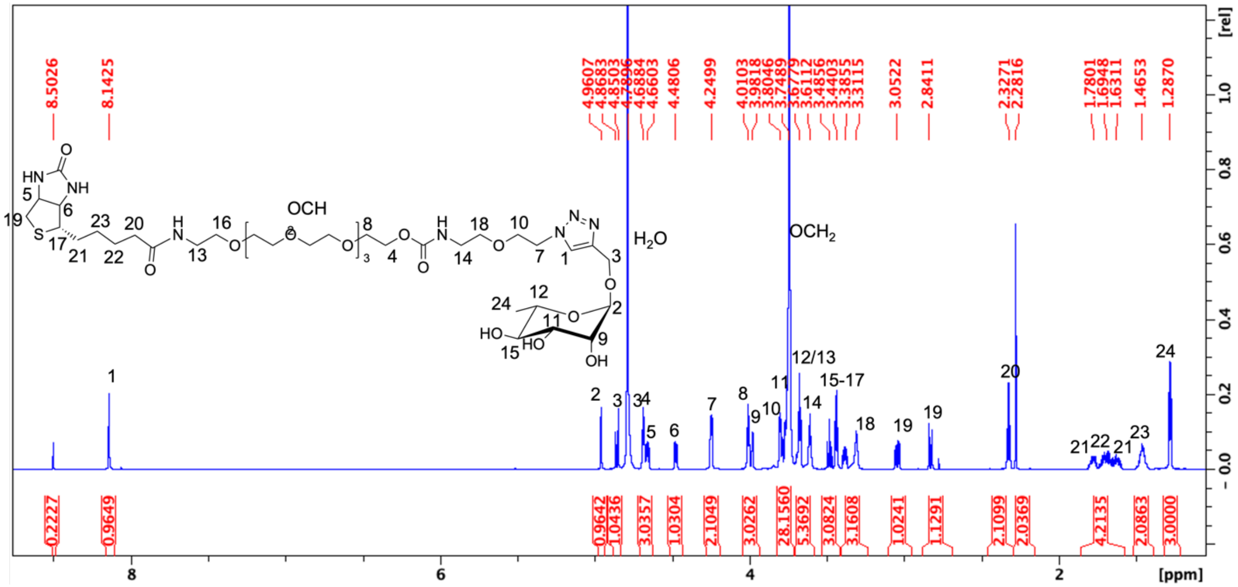

Figure S53. ^{1}H-NMR of **Biotin-α-O-ARM (1),** D_2O_salt.

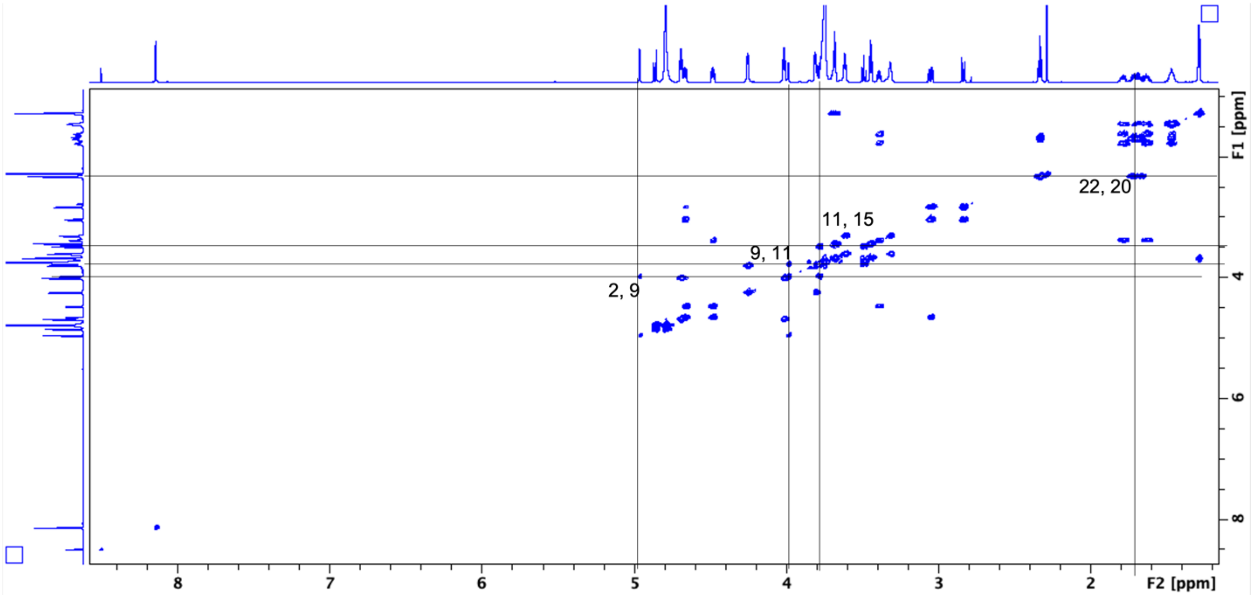

Figure S54. ^{1}H^{1}H-COSY NMR of **Biotin-α-O-ARM (1),** D_2O_salt.

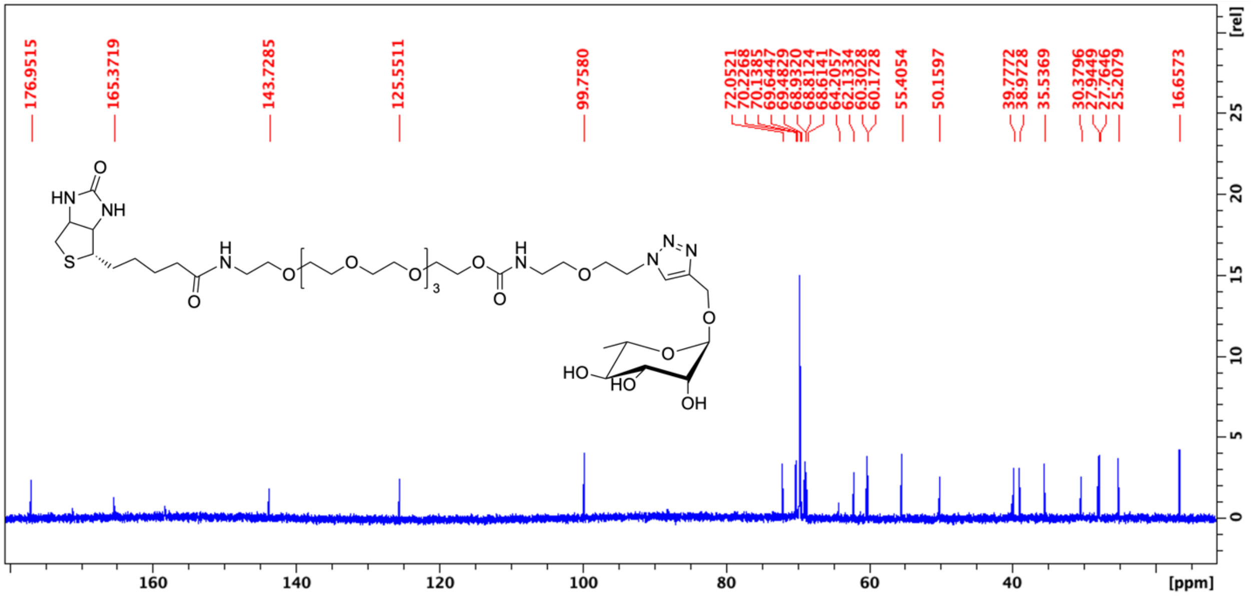

Figure S55. ^{13}C-NMR of **Biotin-α-O-ARM (1),** D_2O_salt.

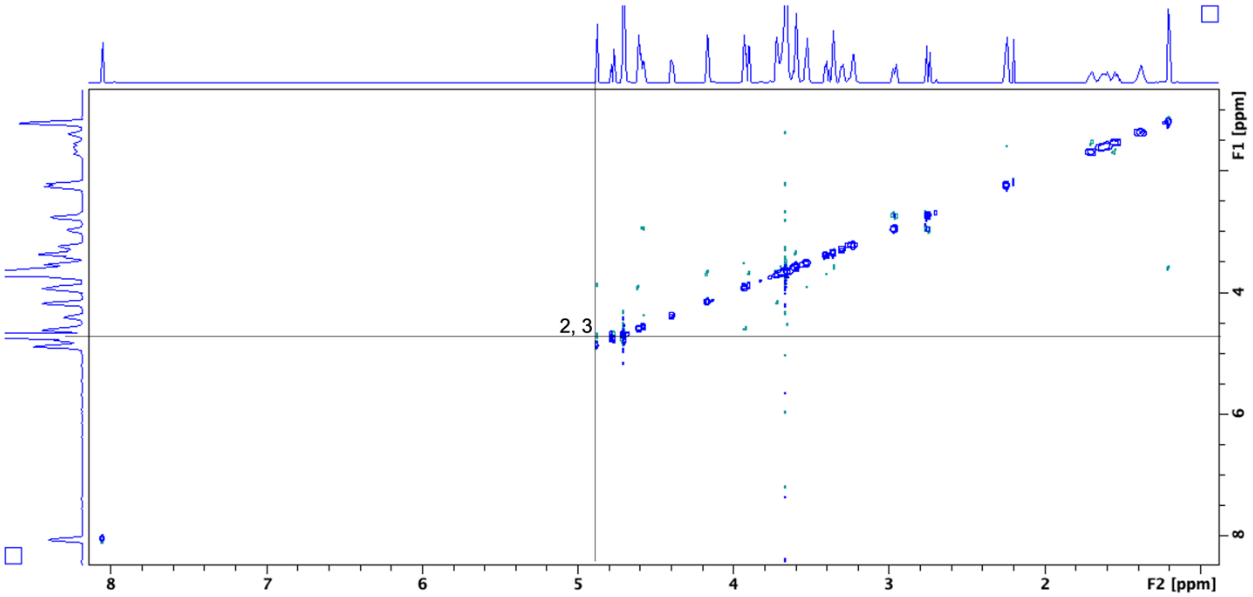

Figure S56. $^1H^1H$-NOESY NMR of **Biotin-α-O-ARM (1),** D_2O_salt.

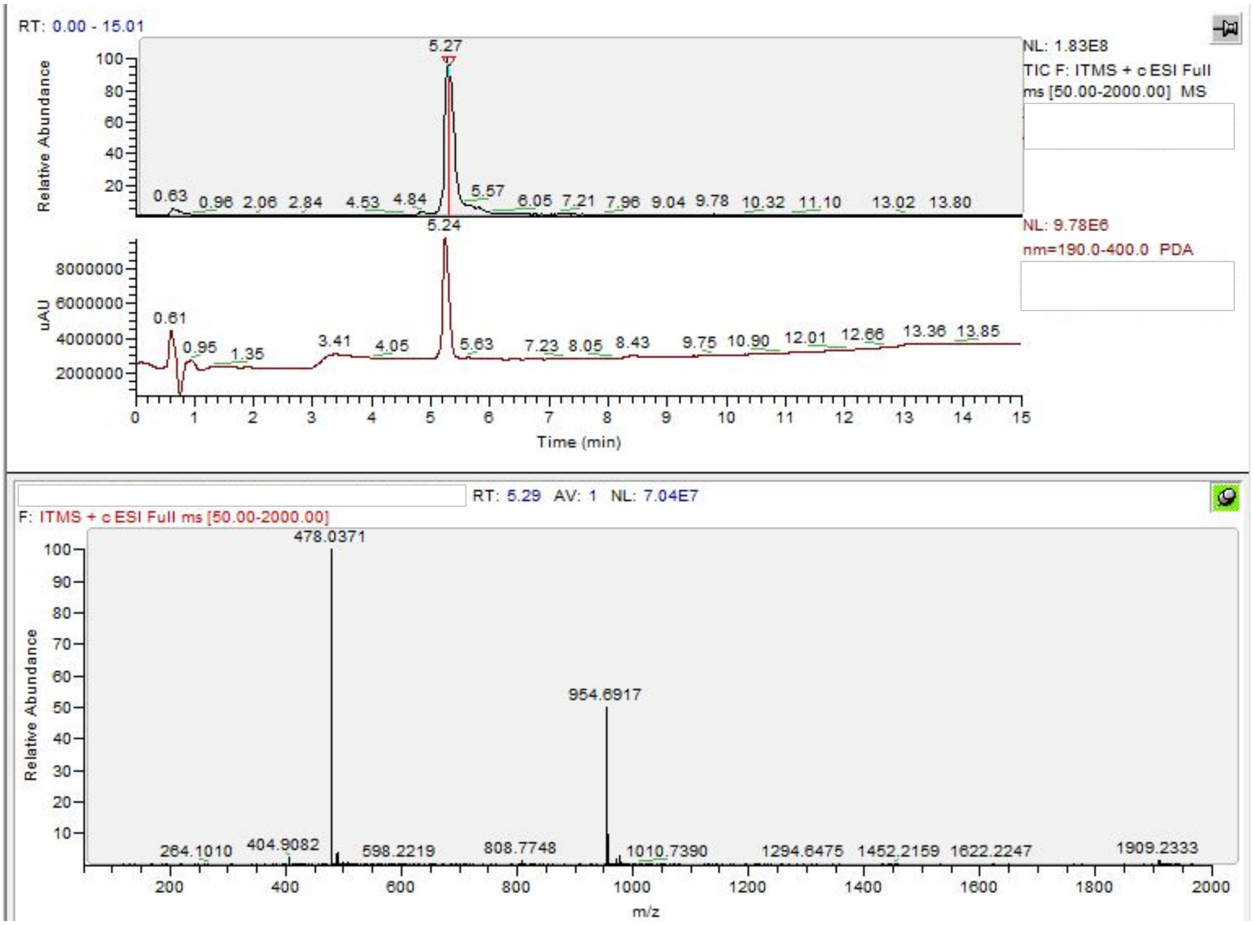

Figure S57. LC-MS trace of post column purified compound **Biotin-α-O-ARM (1)**. Top - TIC trace. Bottom - selected mass spectrum from TIC highlighted by red bar (retention time 5.29). Middle - UV chromatogram.

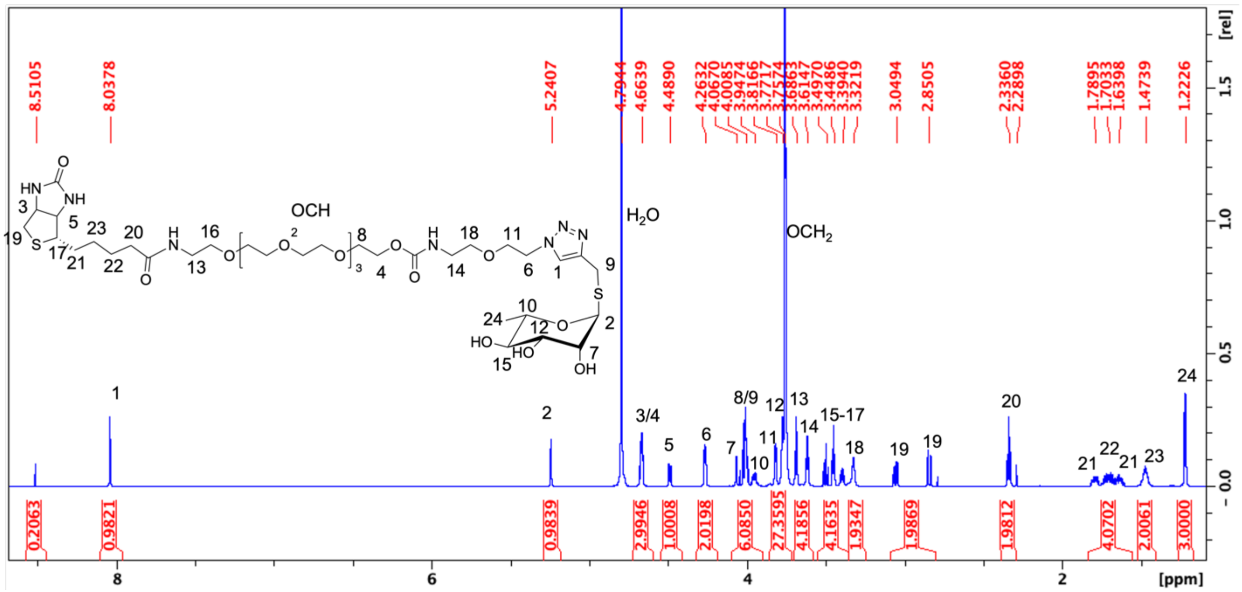

Figure S58. ^{1}H-NMR of **Biotin-α-S-ARM (2),** D_2O_salt.

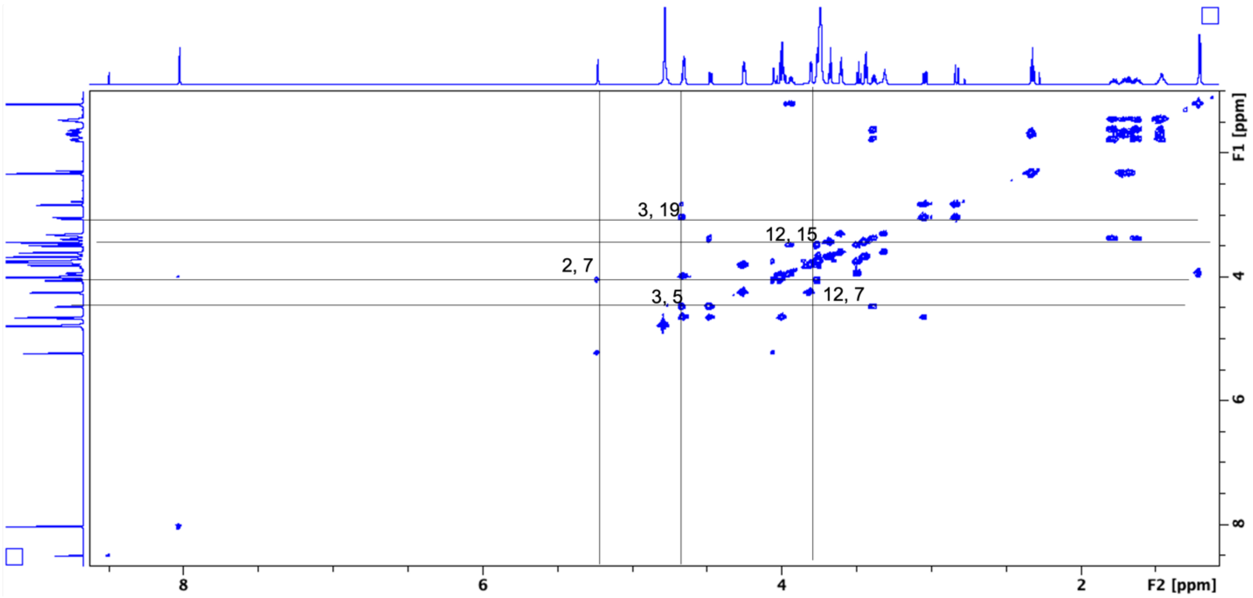

Figure S59. ^{1}H^{1}H-COSY NMR of **Biotin-α-S-ARM (2),** D_2O_salt.

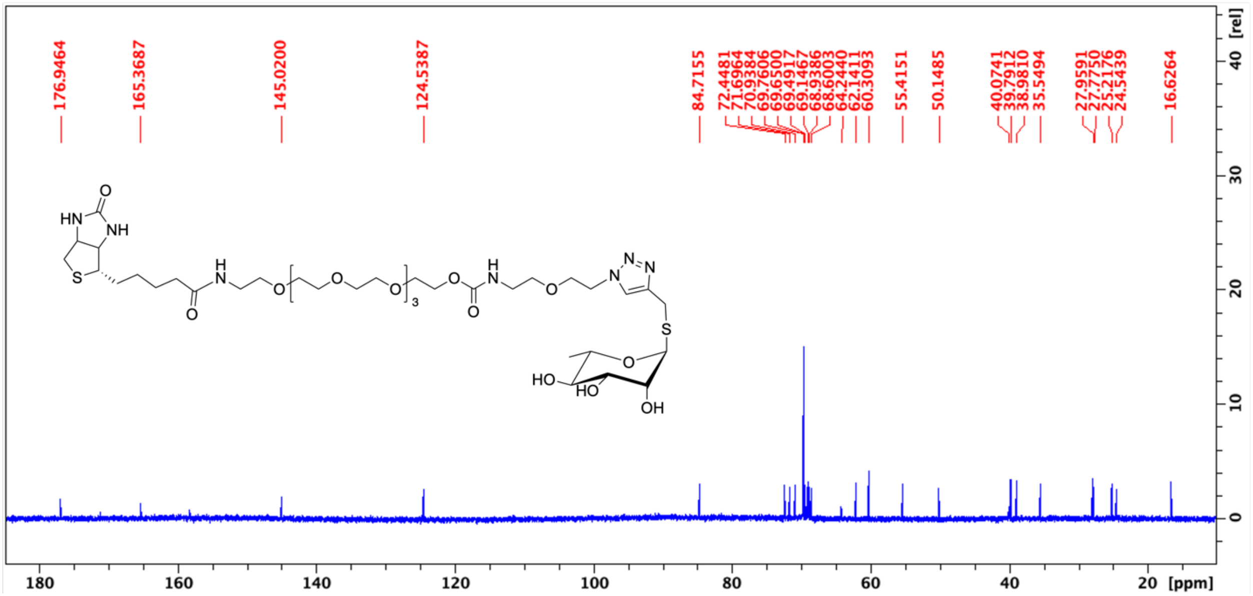

Figure S60. ^{13}C-NMR of **Biotin-α-S-ARM (2),** D_2O_salt.

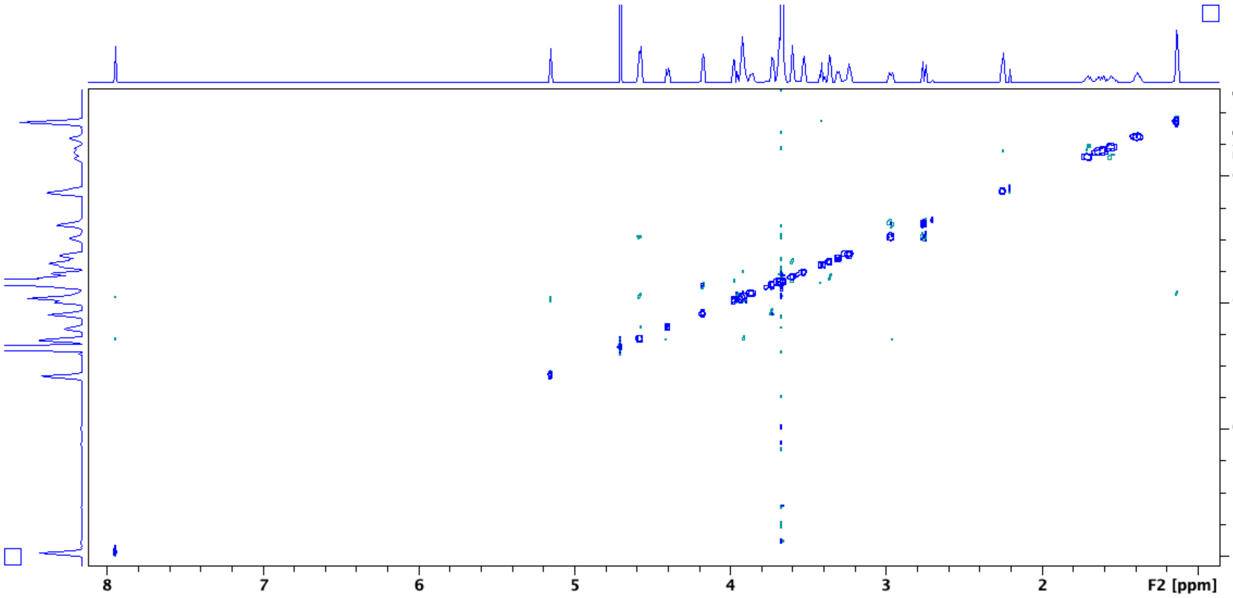

Figure S61. ^{1}H^{1}H-NOESY NMR of **Biotin-α-S-ARM (2),** D_2O_salt.

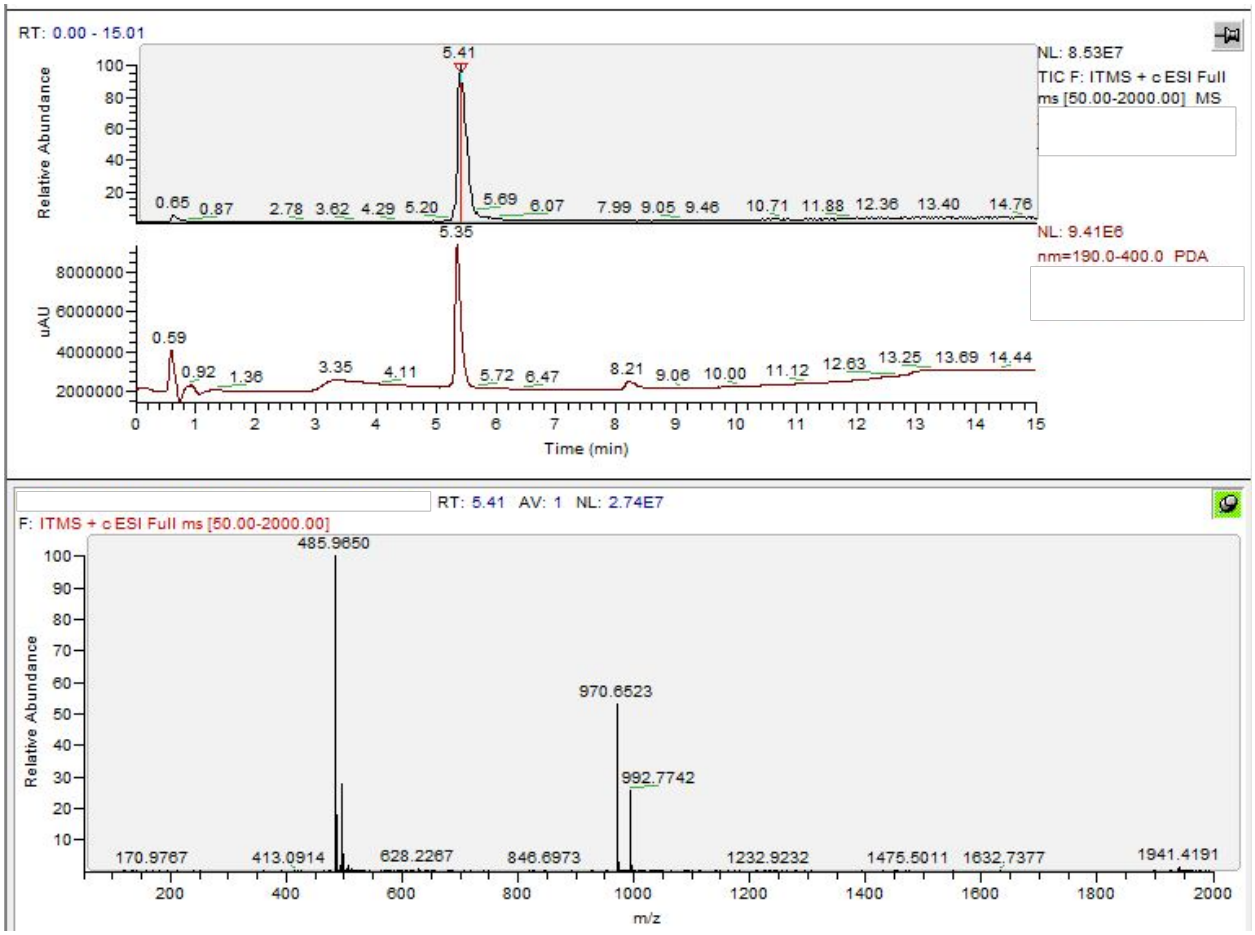

Figure S62. LC-MS trace of post column purified compound **Biotin-α-S-ARM (2)**. Top - TIC trace. Bottom - selected mass spectrum from TIC highlighted by red bar (retention time 5.41). Middle - UV chromatogram.

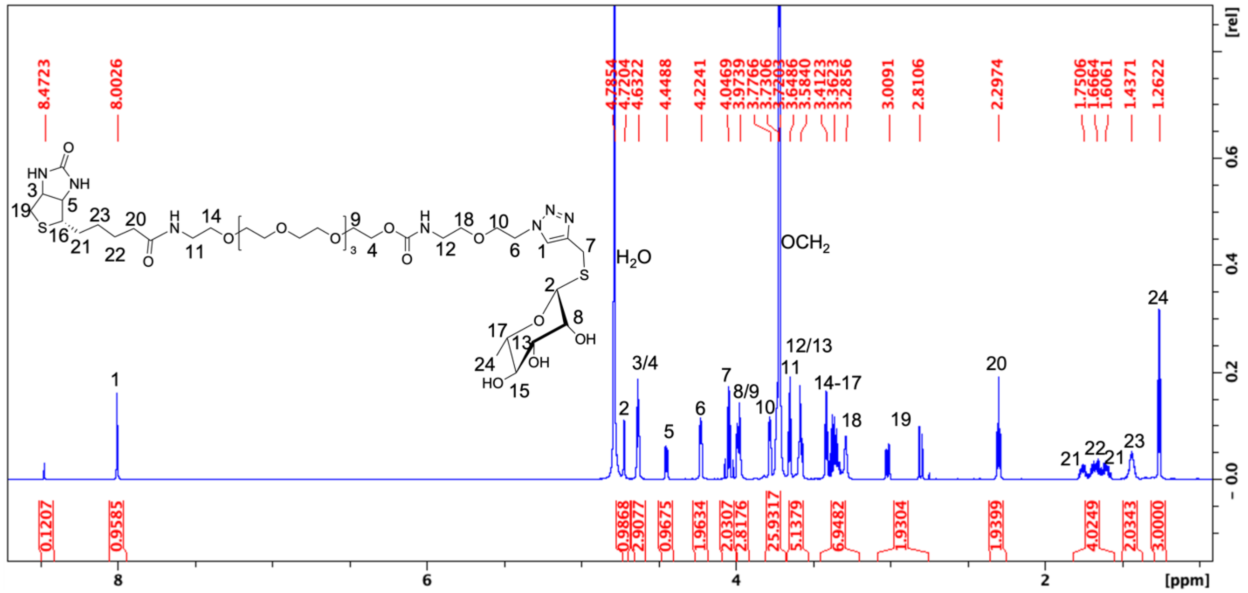

Figure S63. ^{1}H-NMR of **Biotin-β-S-ARM (3),** D_2O_salt.

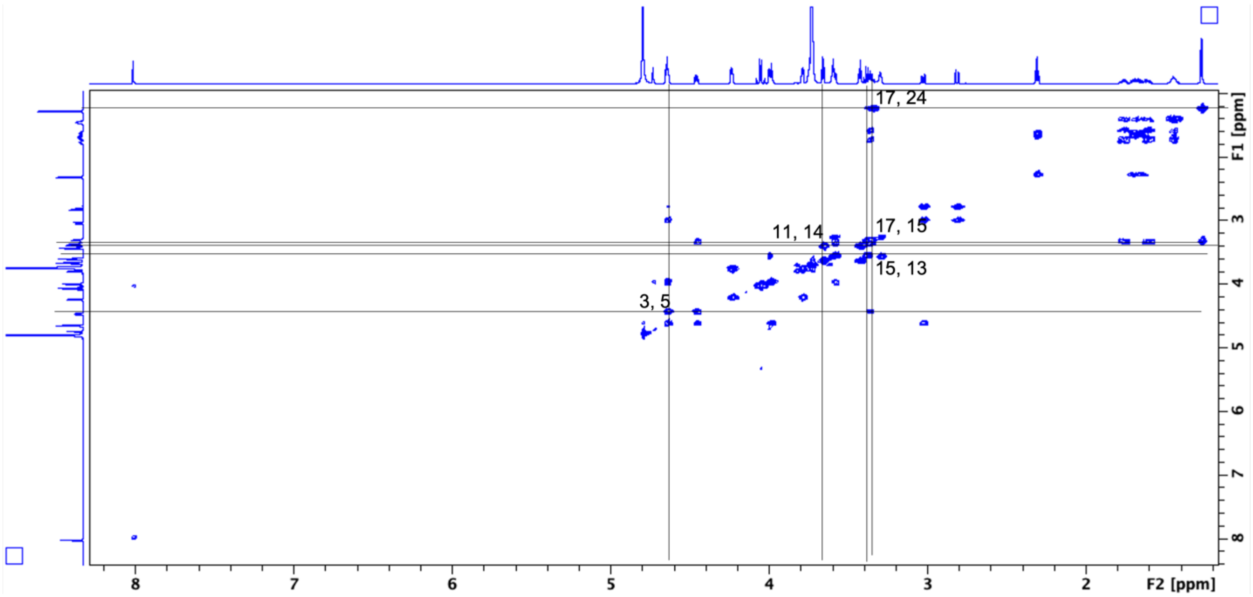

Figure S64. ^{1}H^{1}H-COSY NMR of **Biotin-β-S-ARM (3),** D_2O_salt.

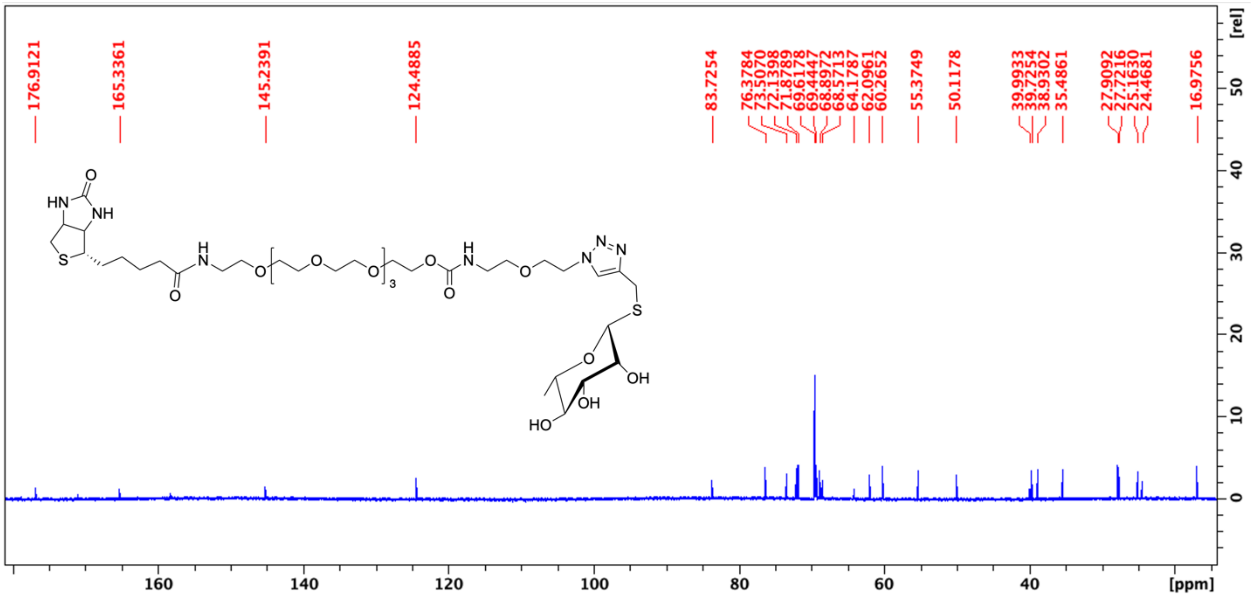

Figure S65. ^{13}C-NMR of **Biotin-β-S-ARM (3),** D_2O_salt.

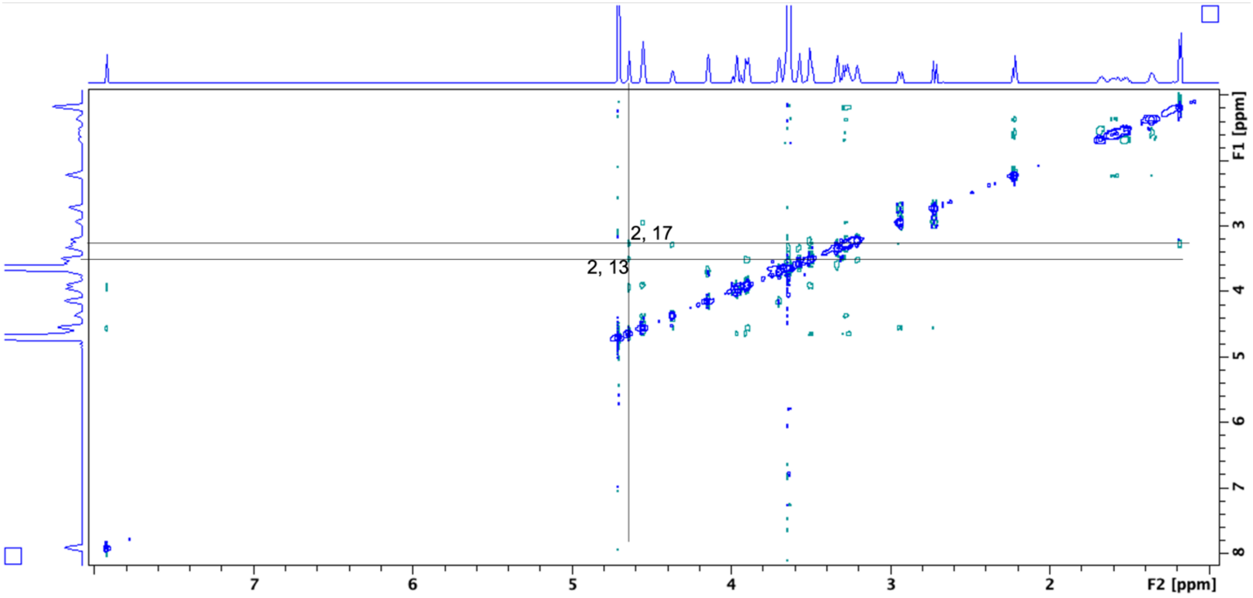

Figure S66. ^{1}H^{1}H-NOESY NMR of **Biotin-β-S-ARM (3),** D_2O_salt.

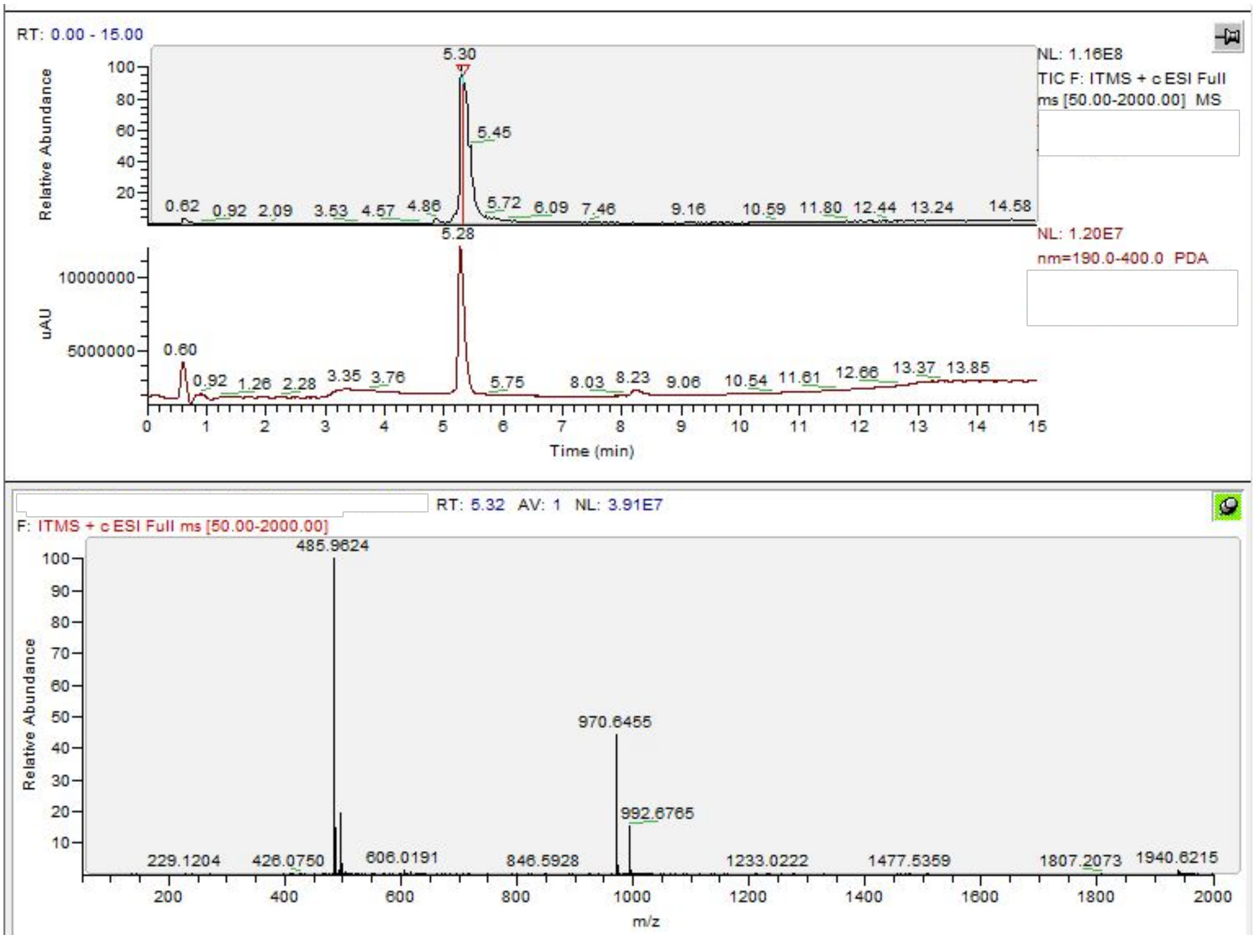

Figure S67. LC-MS trace of post column purified compound **Biotin-β-S-ARM (3)**. Top - TIC trace. Bottom - selected mass spectrum from TIC highlighted by red bar (retention time 5.32). Middle - UV chromatogram.

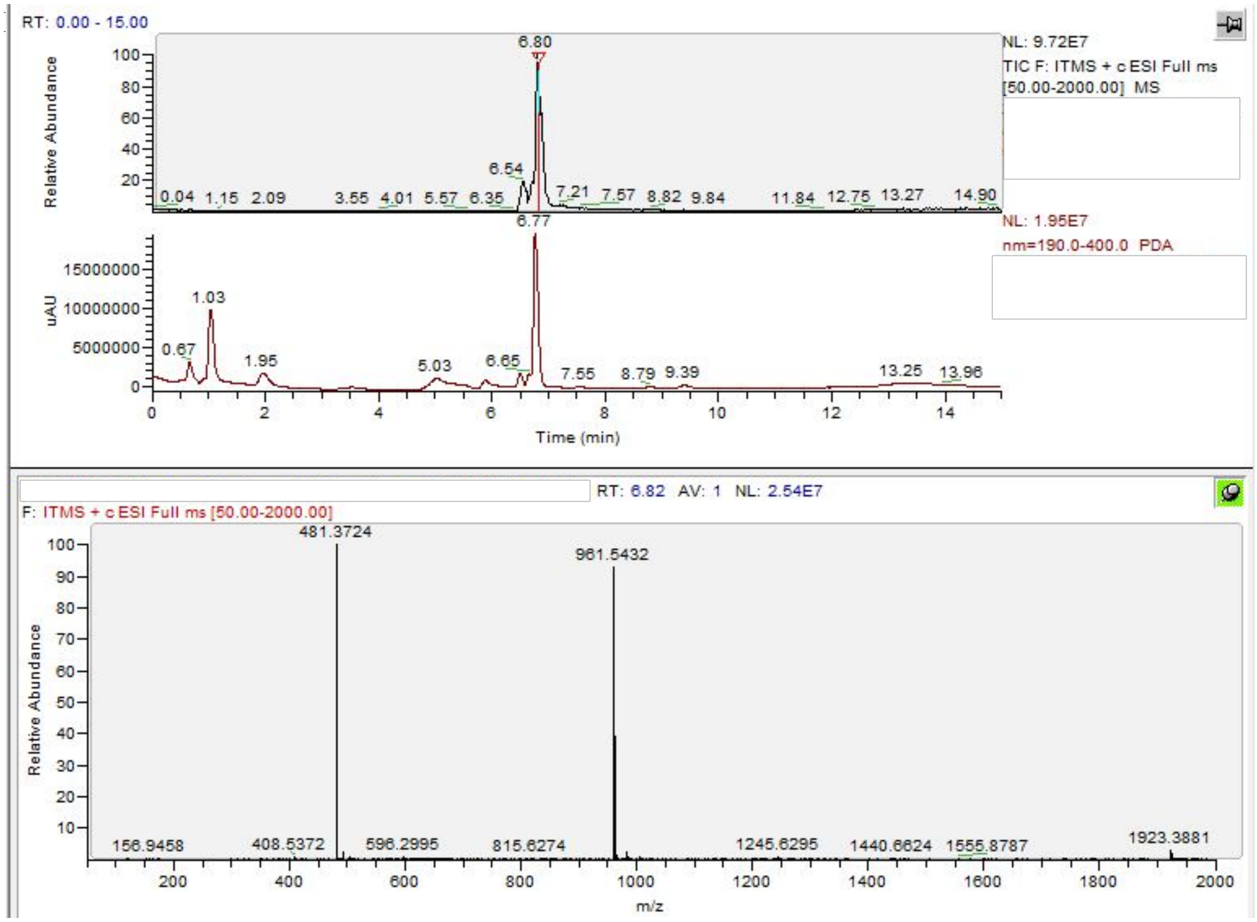

Figure S68. LC-MS trace of post column purified compound **Biotin-α-O-cARM (4)**. Top - TIC trace. Bottom - selected mass spectrum from TIC highlighted by red bar (retention time 6.82). Middle - UV chromatogram.

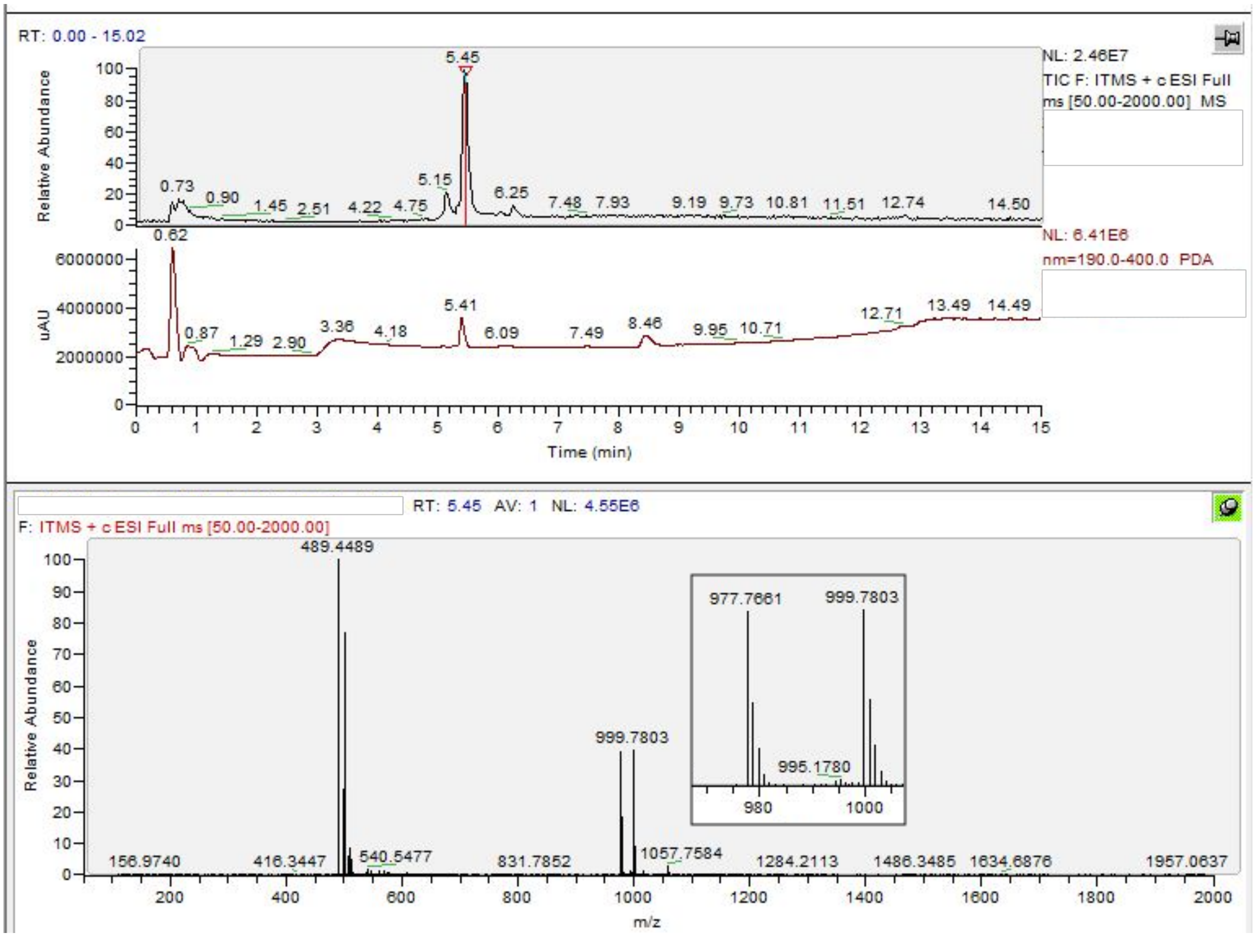

Figure S69. LC-MS trace of post column purified compound **Biotin-α-S-cARM (5)**. Top - TIC trace. Bottom - selected mass spectrum from TIC highlighted by red bar (retention time 5.45). The $[M+Na]^+$ mass is seen (999.7803). To highlight the $[M+H]^+$ peak, a box highlighting m/z around 990 is superimposed. Middle - UV chromatogram.

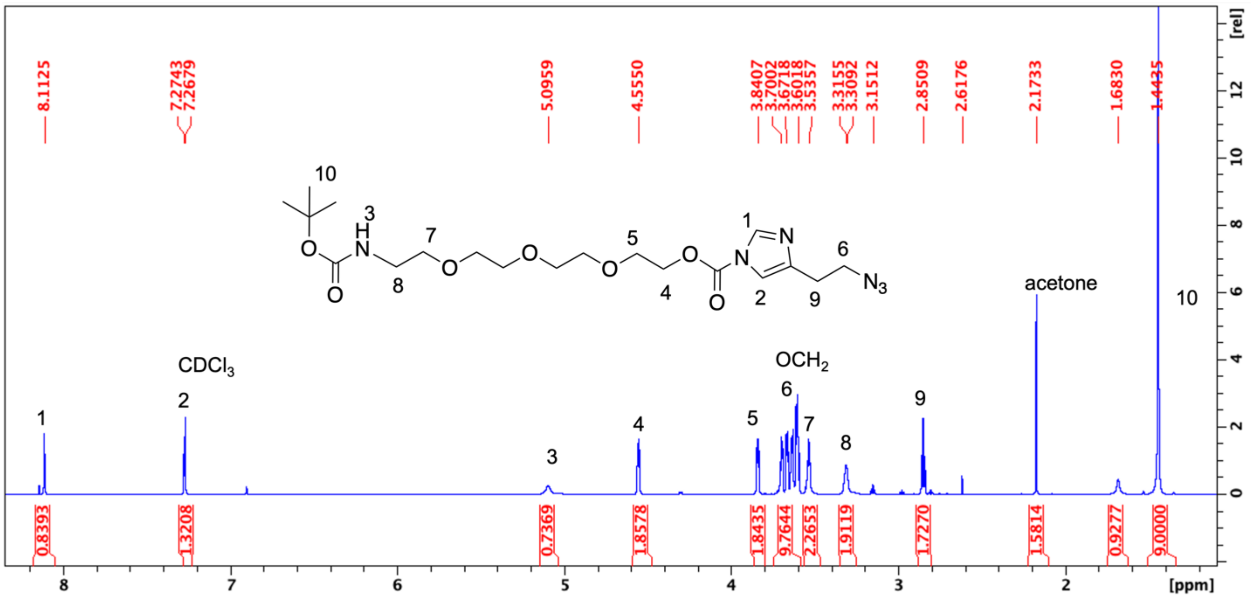

Figure S70. ^{1}H-NMR of **Boc-Peg4-AI-Azide (18)**, $CDCl_3$.

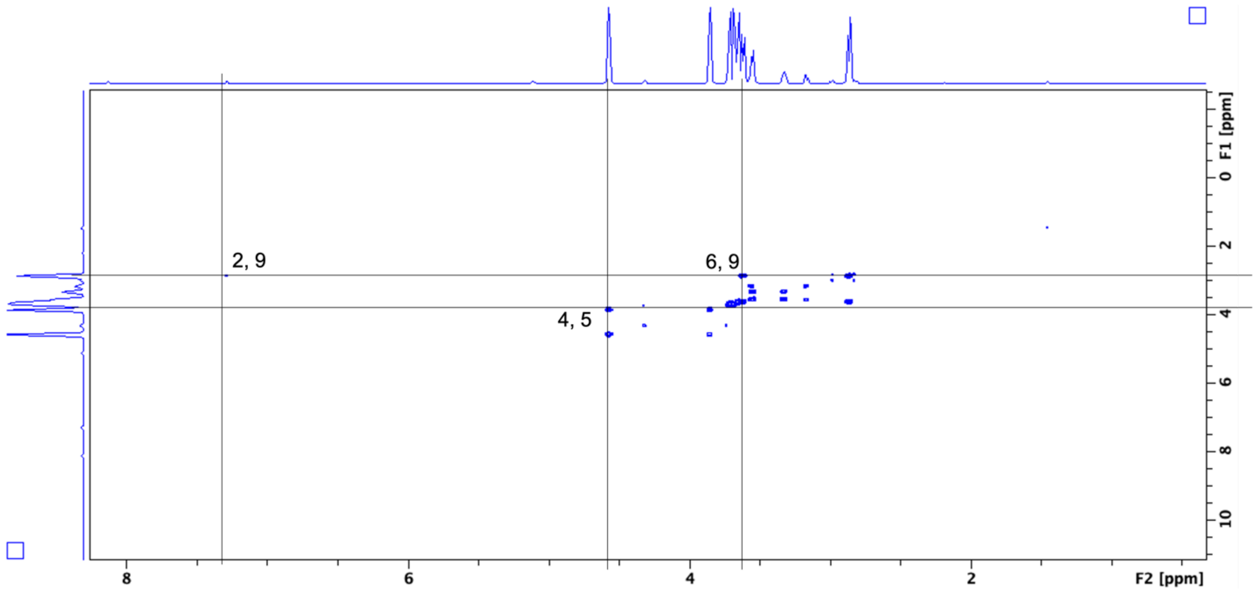

Figure S71. ^{1}H^{1}H-COSY NMR of **Boc-Peg4-AI-Azide (18)**, $CDCl_3$.

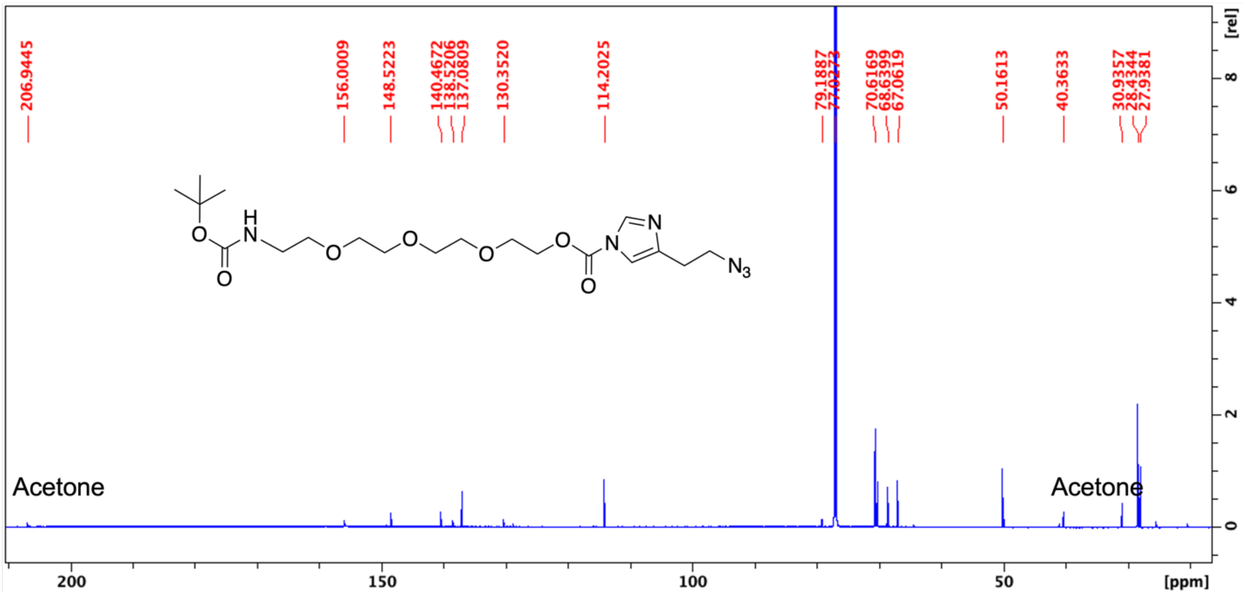

Figure S72. ^{13}C-NMR of **Boc-Peg4-AI-Azide (18)**, $CDCl_3$.

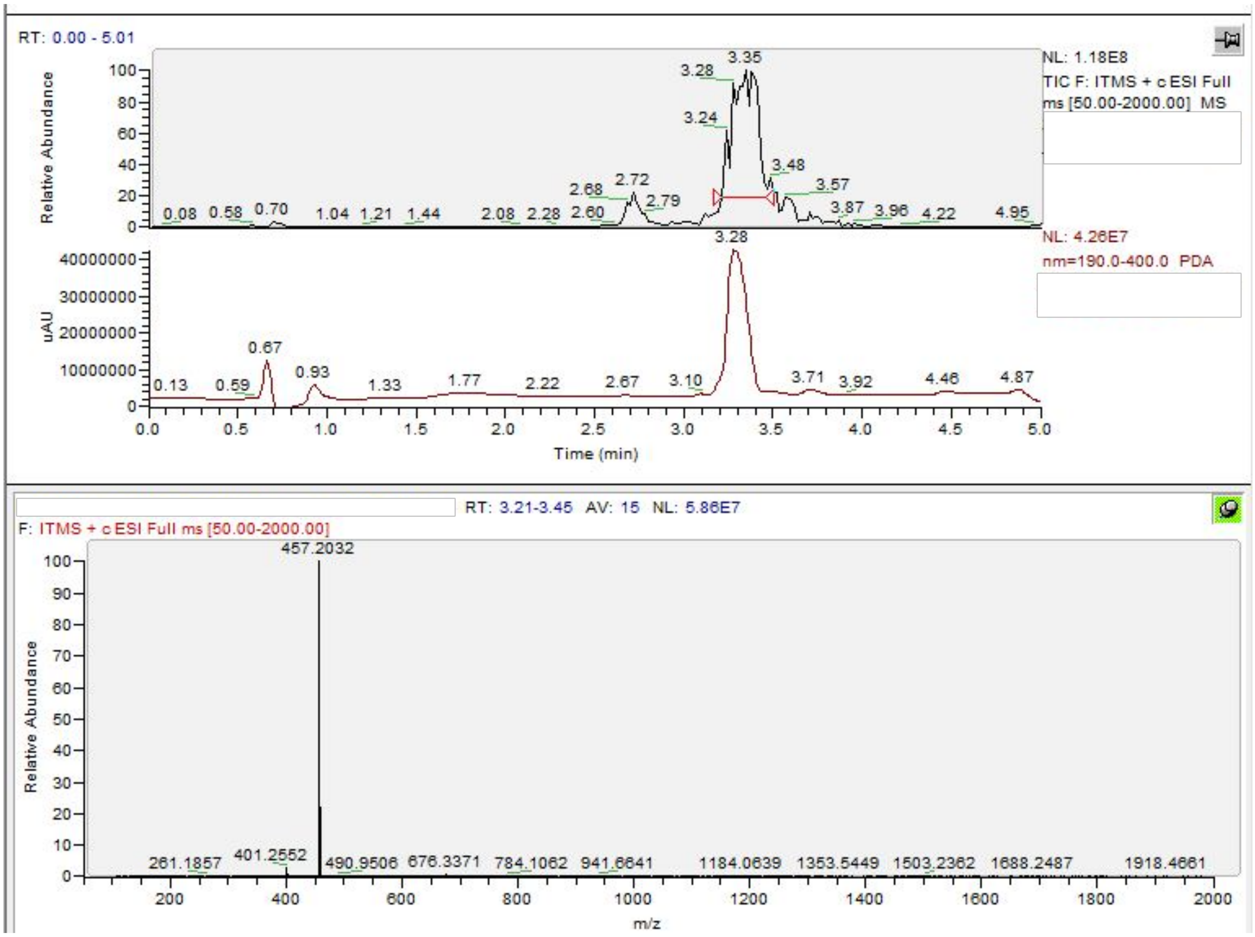

Figure S73. LC-MS trace of post column purified compound **Boc-Peg4-AI-Azide (18)**. Top - TIC trace. Bottom - selected mass spectrum from TIC highlighted by red bar (retention time 3.21 – 3.45). Middle - UV chromatogram.

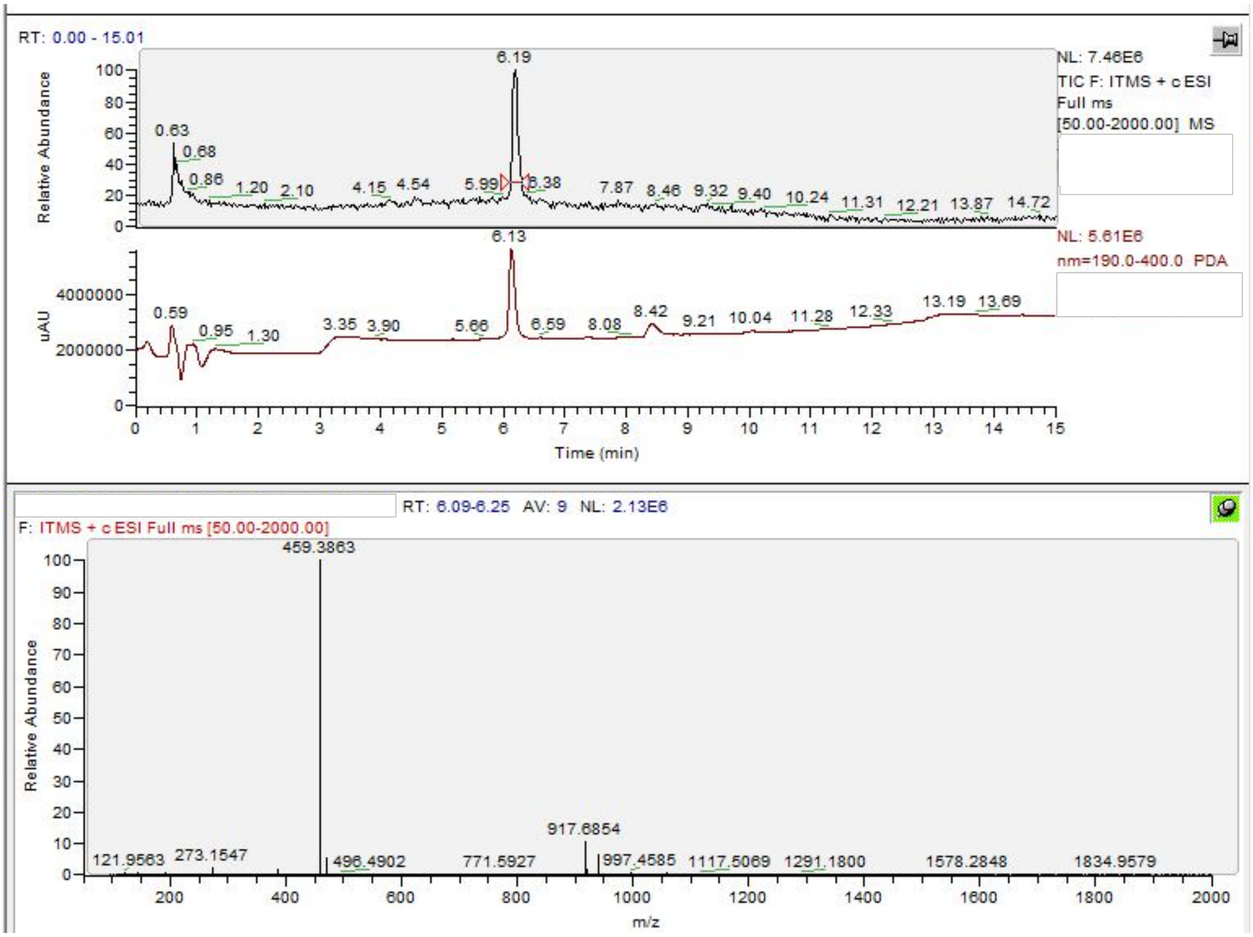

Figure S74. LC-MS trace of post column purified compound **Fluor-O-cARM (6)**. Top - TIC trace. Bottom - selected mass spectrum from TIC highlighted by red bar (retention time 6.09 – 6.25). Middle - UV chromatogram.

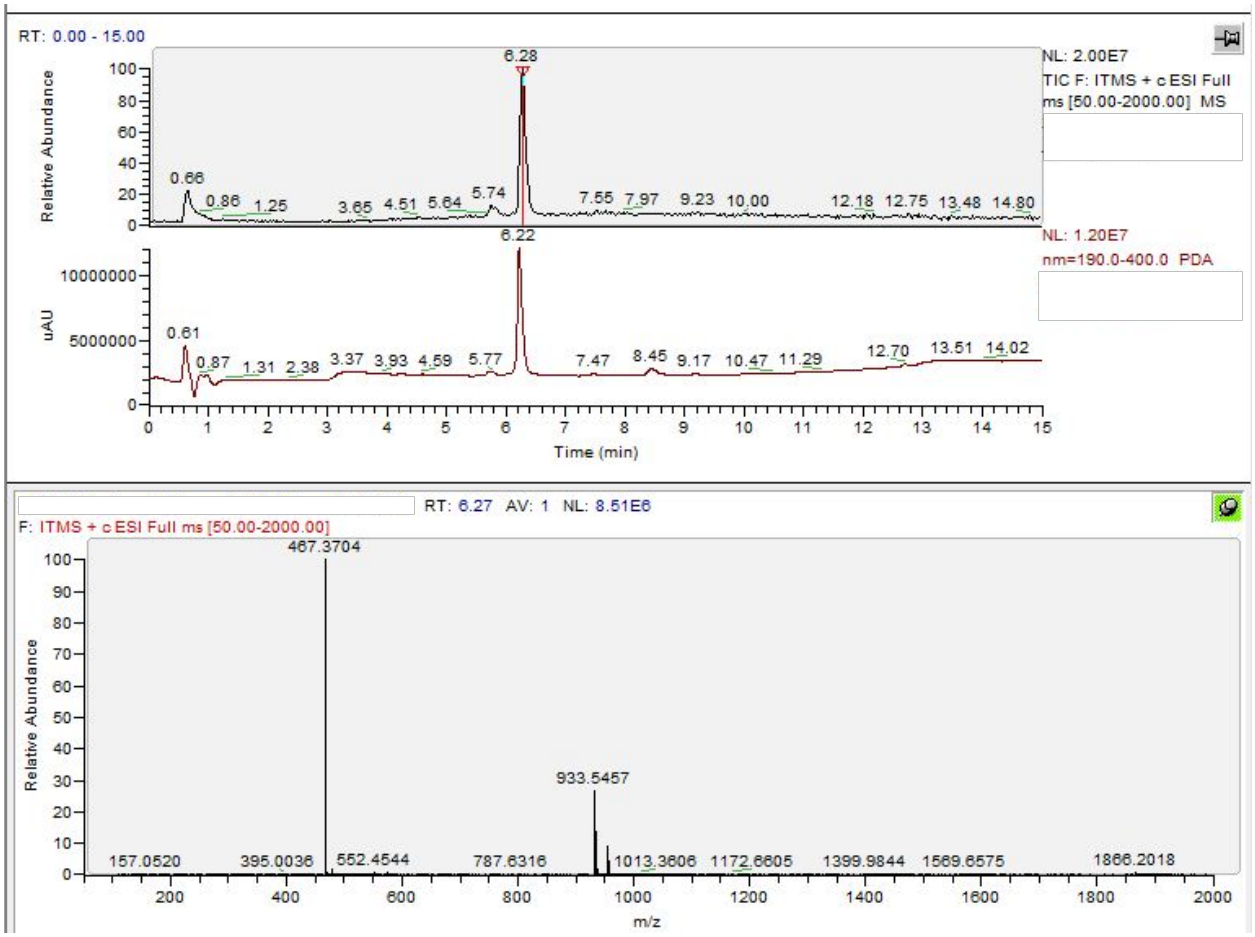

Figure S75. LC-MS trace of post column purified compound **Fluor-S-cARM (7)**. Top - TIC trace. Bottom - selected mass spectrum from TIC highlighted by red bar (retention time 6.27). Middle - UV chromatogram.

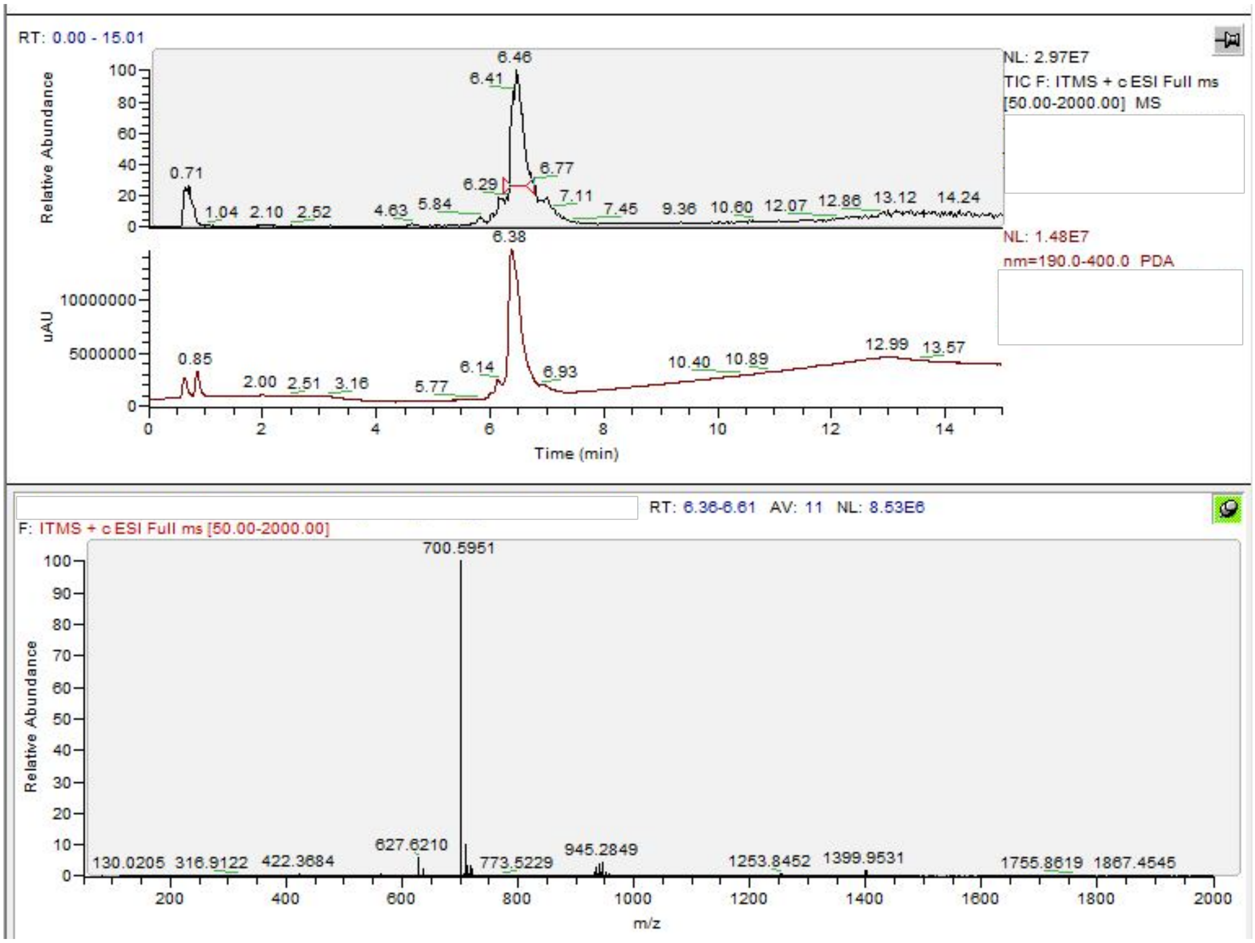

Figure S76. LC-MS trace of post column purified compound **A647-cARM (8)**. Top - TIC trace. Bottom - selected mass spectrum from TIC highlighted by red bar (retention time 6.36-6.61). Middle - UV chromatogram.

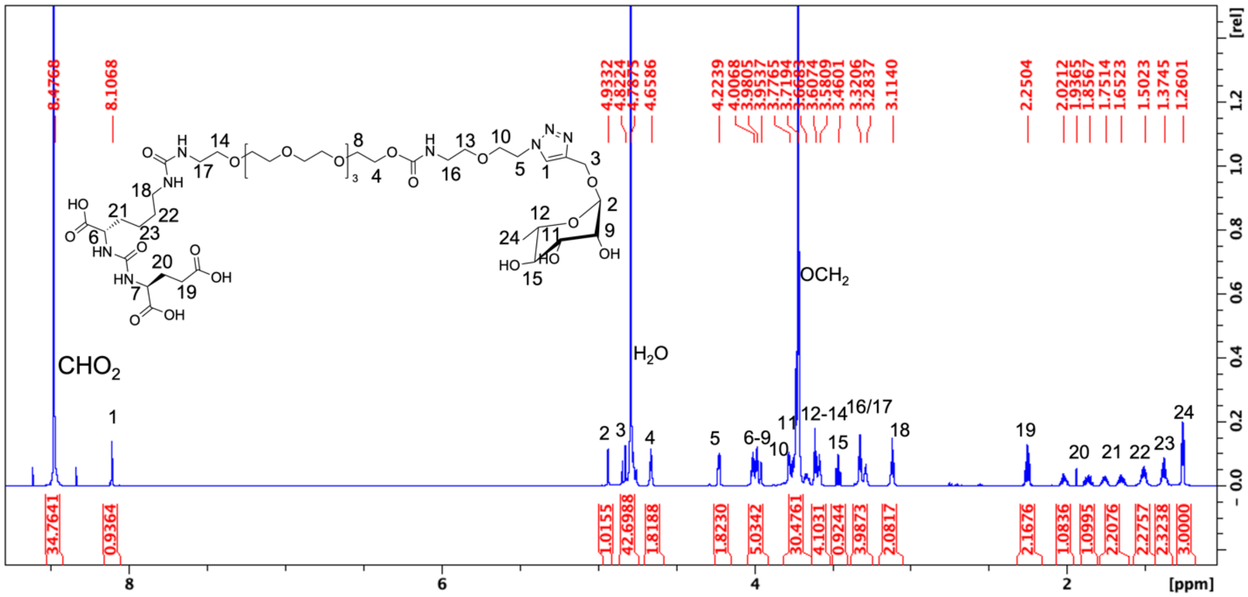

Figure S77. ^{1}H-NMR of **GUL-PEG-ARM**, D_2O_salt.

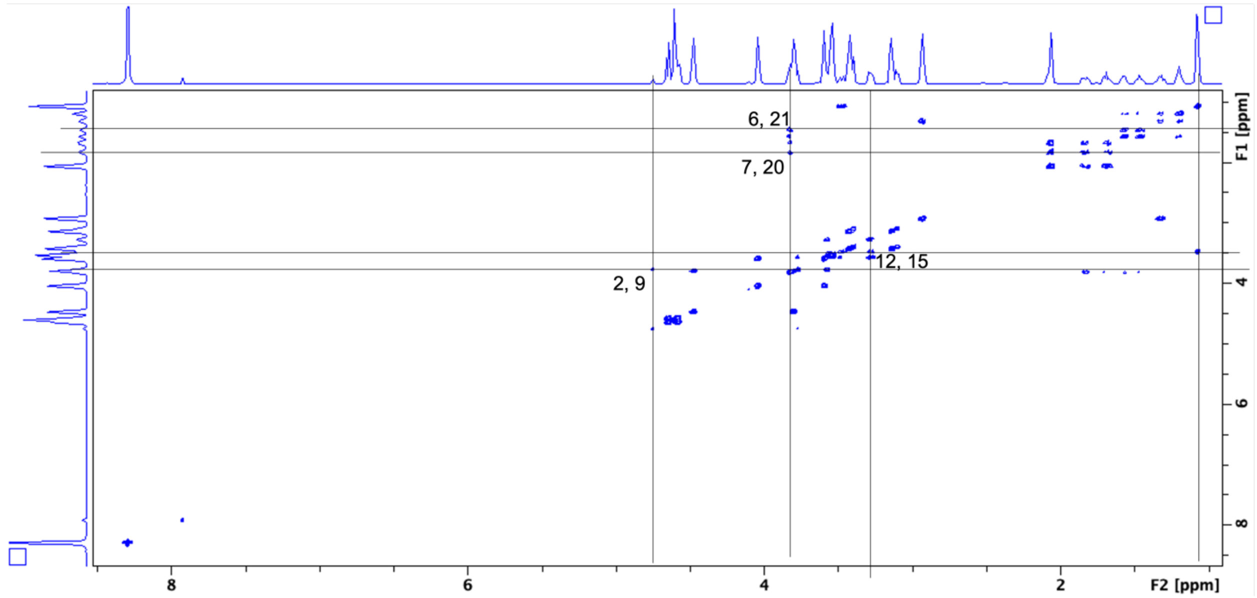

Figure S78. $^{1}H^{1}H$-COSY NMR of **GUL-PEG-ARM**, D_2O_salt.

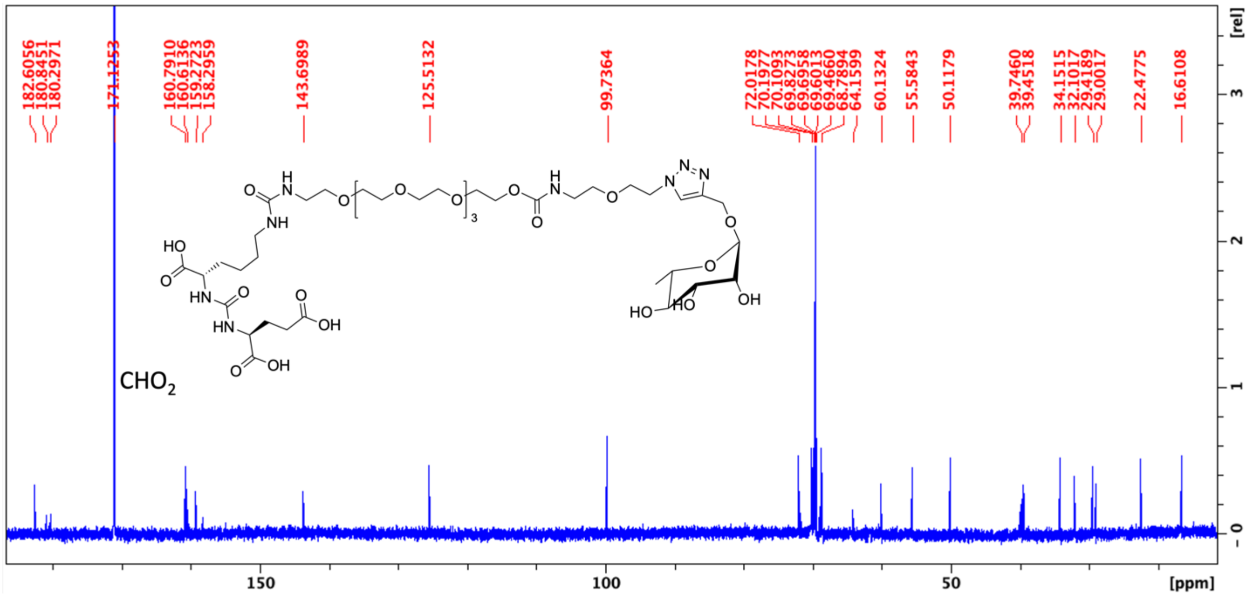

Figure S79. ^{13}C-NMR of **GUL-PEG-ARM**, D_2O_salt.

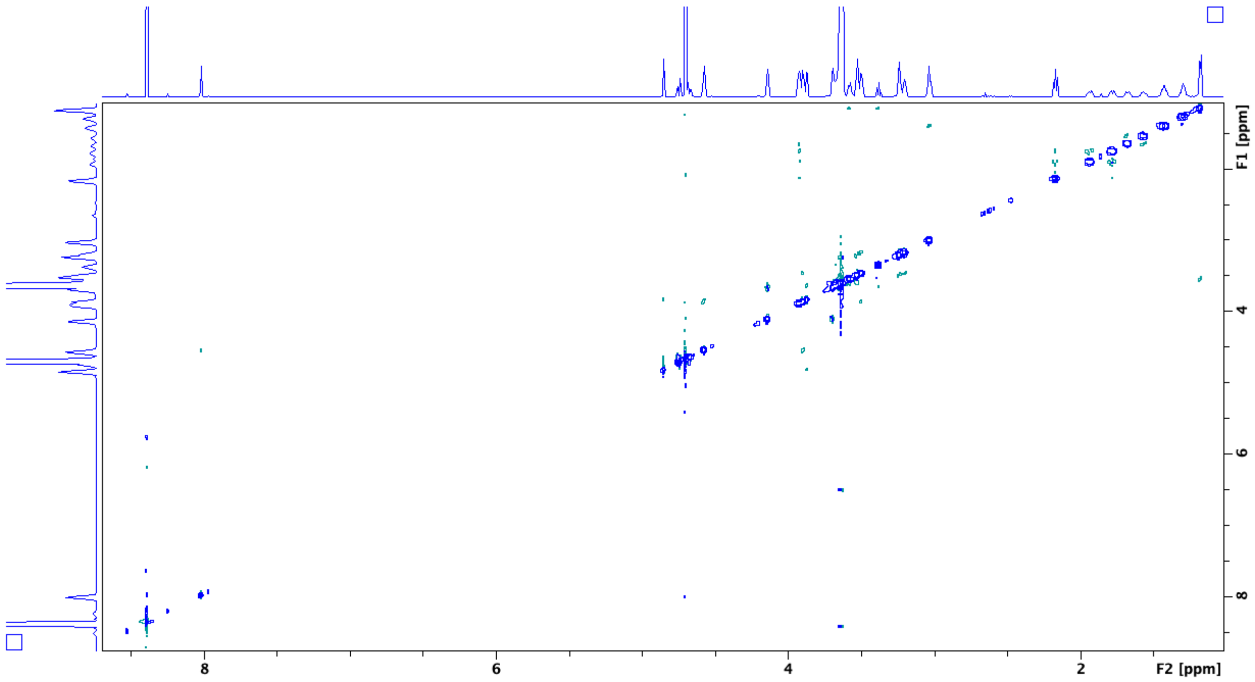

Figure S80. ^{1}H^{1}H-NOESY NMR of **GUL-PEG-ARM**, D_2O_salt.

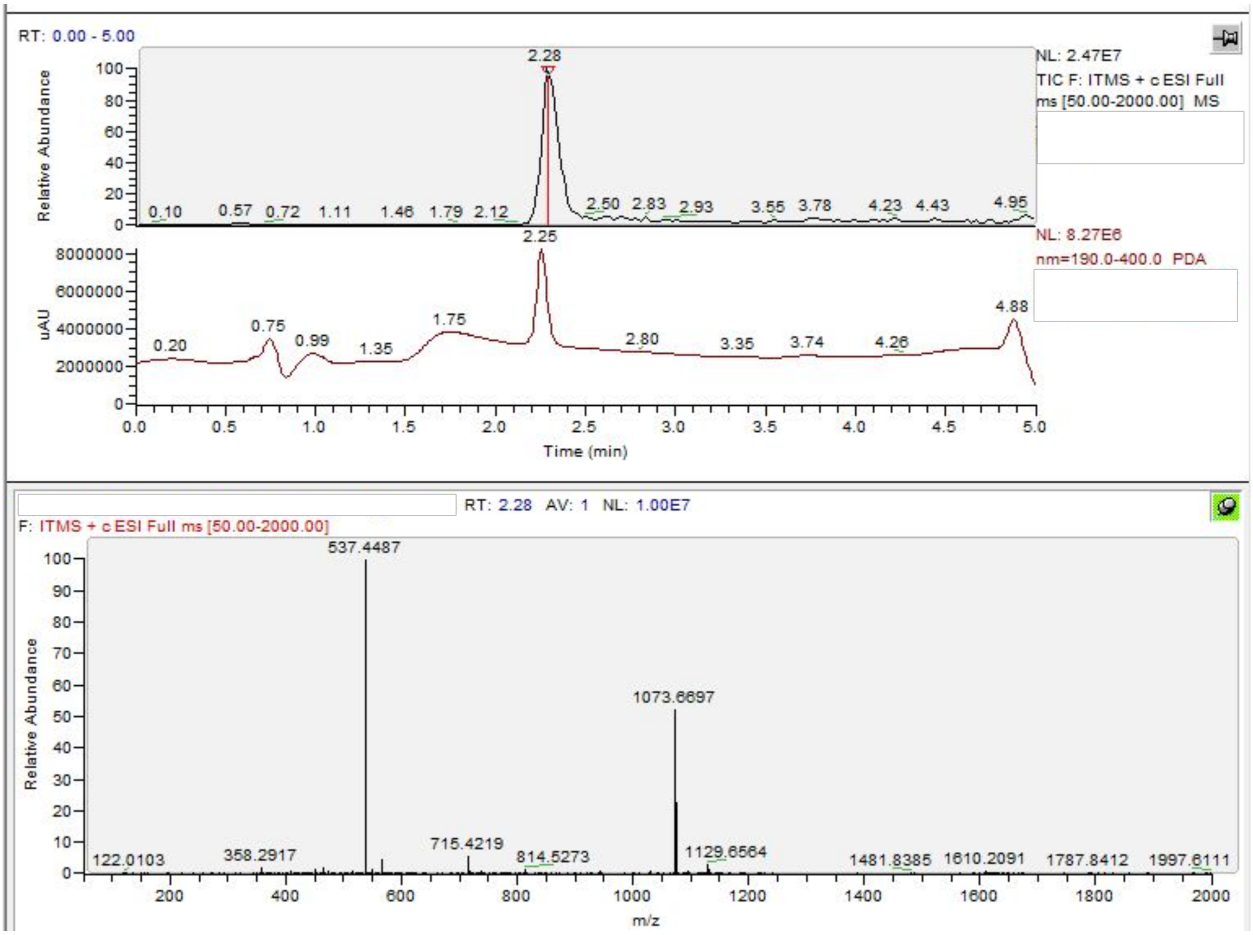

Figure S81. LC-MS trace of post column purified compound **GUL-PEG-ARM**. Top - TIC trace. Bottom - selected mass spectrum from TIC highlighted by red bar (retention time 2.28). Middle - UV chromatogram.

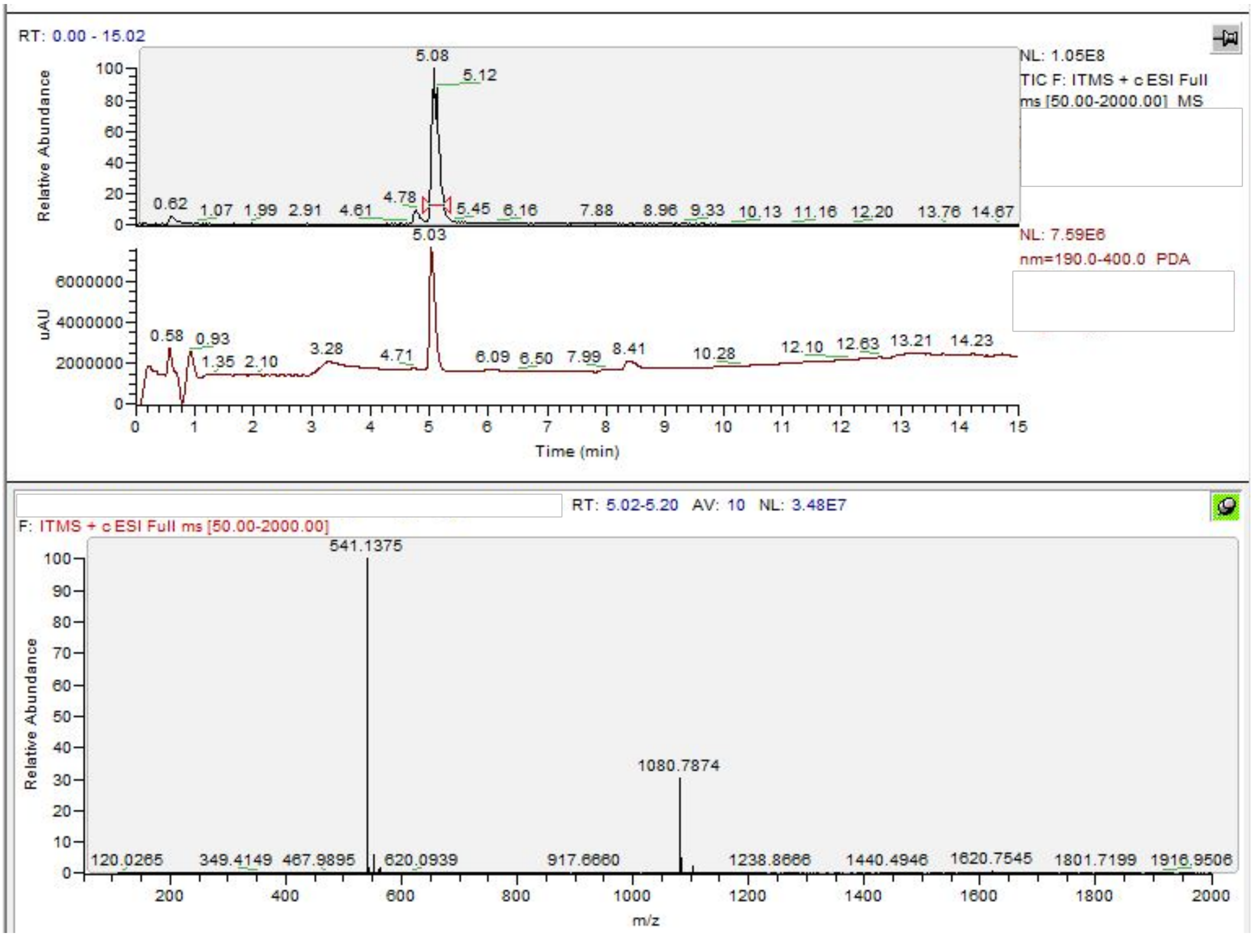

Figure S82. LC-MS trace of post column purified compound **GUL-PEG-cARM (9)**. Top - TIC trace. Bottom - selected mass spectrum from TIC highlighted by red bar (retention time 5.02 – 5.20). Middle - UV chromatogram.

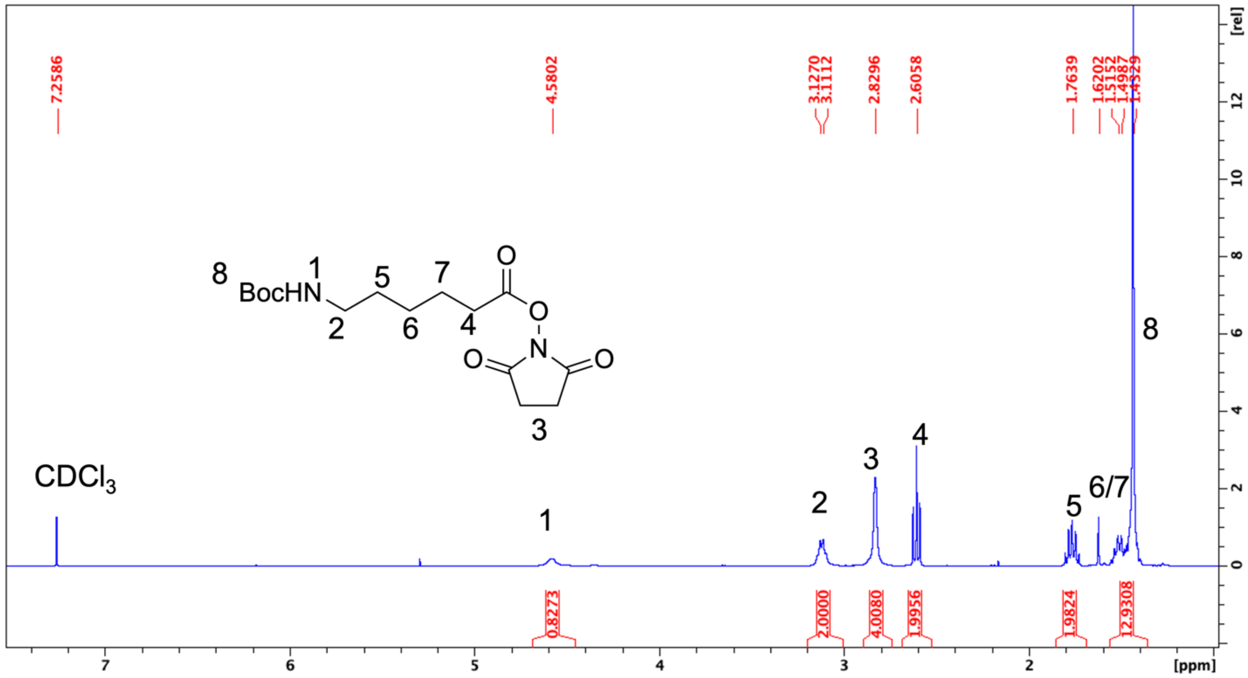

Figure S83. ^{1}H-NMR of **BocNH-Hexanoic-NHS ester (25)**, $CDCl_3$.

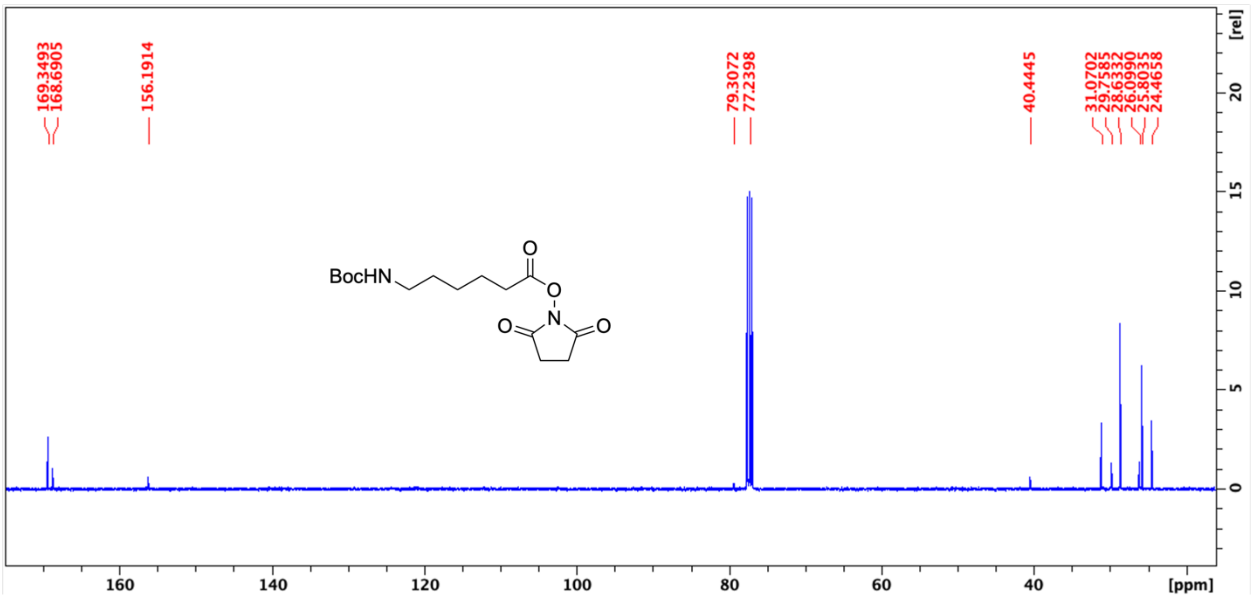

Figure S84. ^{13}C-NMR of **BocNH-Hexanoic-NHS ester (25)**, $CDCl_3$.

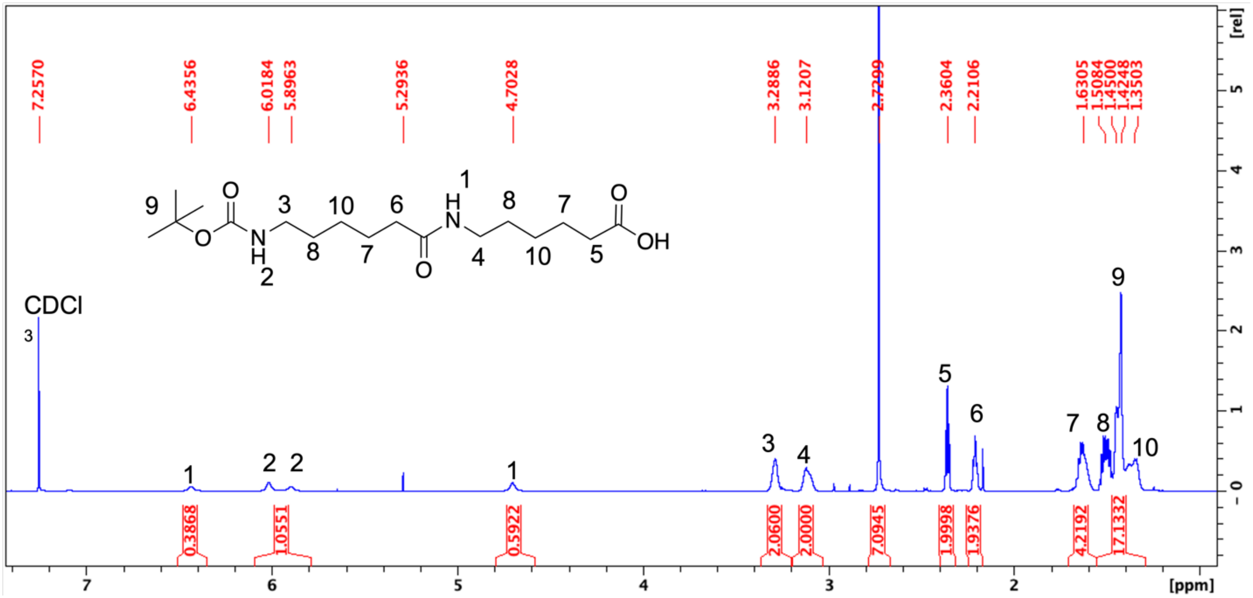

Figure S85. ^{1}H-NMR of **BocNH-(Hexanoic)$_2$-Carboxylic Acid (26),** $CDCl_3$.

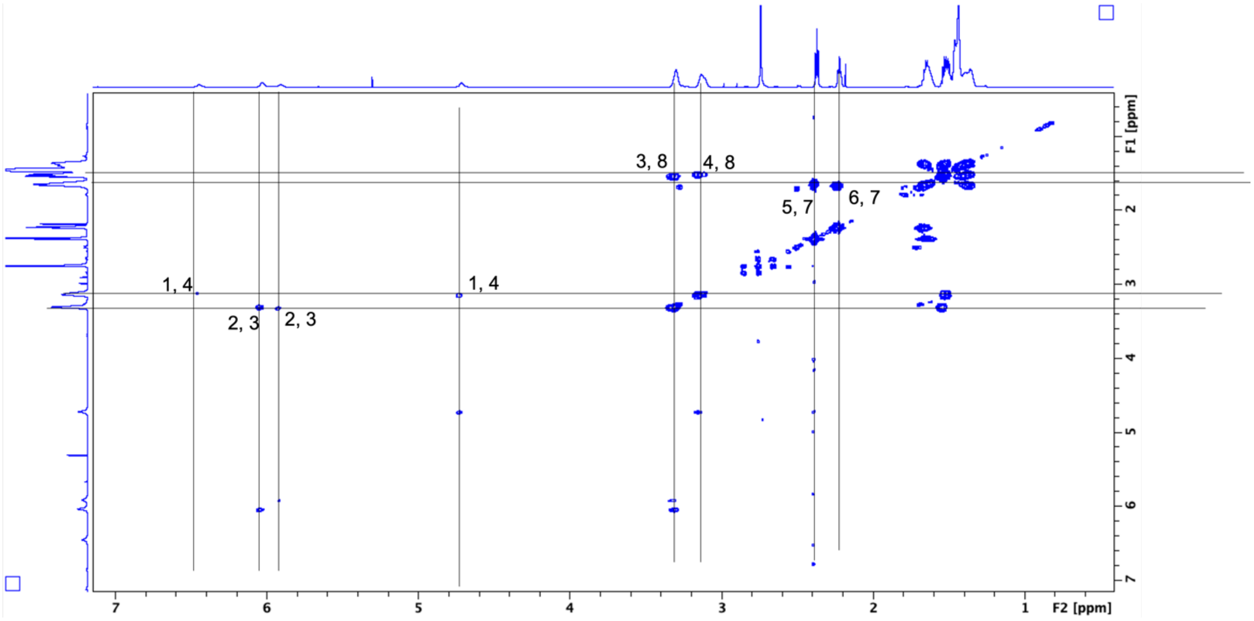

Figure S86. ^{1}H^{1}H-COSY NMR of **BocNH-(Hexanoic)$_2$-Carboxylic Acid (26),** $CDCl_3$.

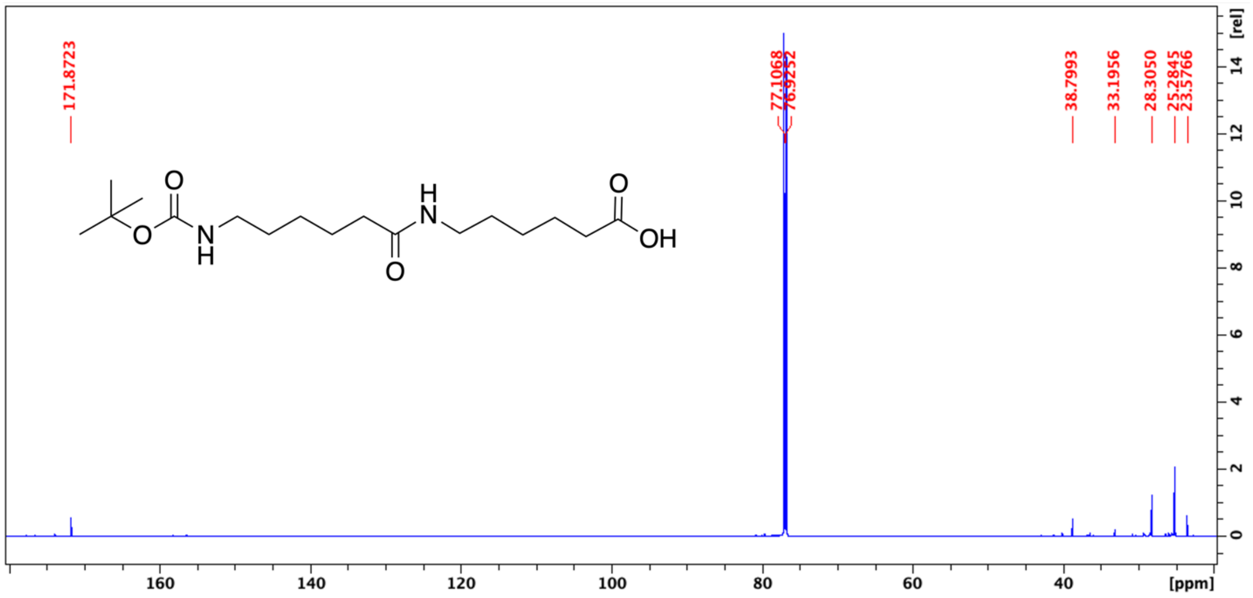

Figure S87. ^{13}C-NMR of **BocNH-(Hexanoic)$_2$-Carboxylic Acid (26),** $CDCl_3$.

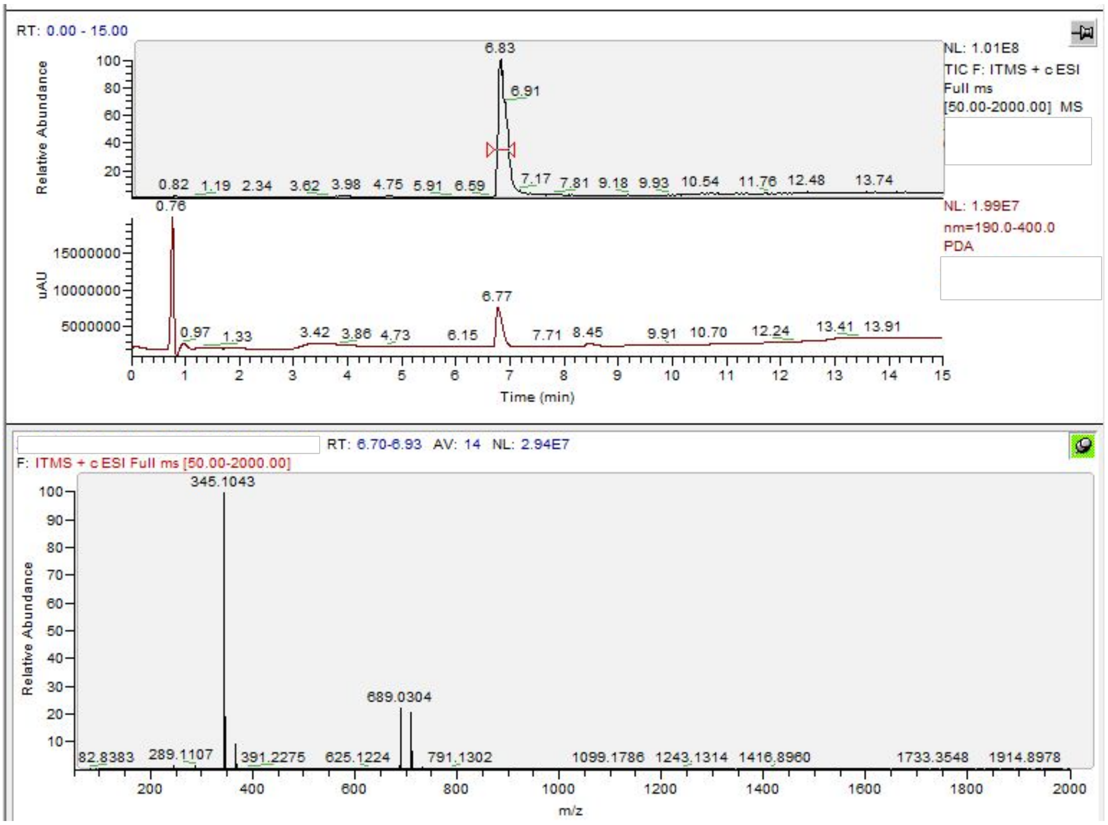

Figure S88. LC-MS trace of post column purified compound **BocNH-(Hexanoic)$_2$-Carboxylic Acid (26)**. Top - TIC trace. Bottom - selected mass spectrum from TIC highlighted by red bar (retention time 6.70 – 6.93). Middle - UV chromatogram.

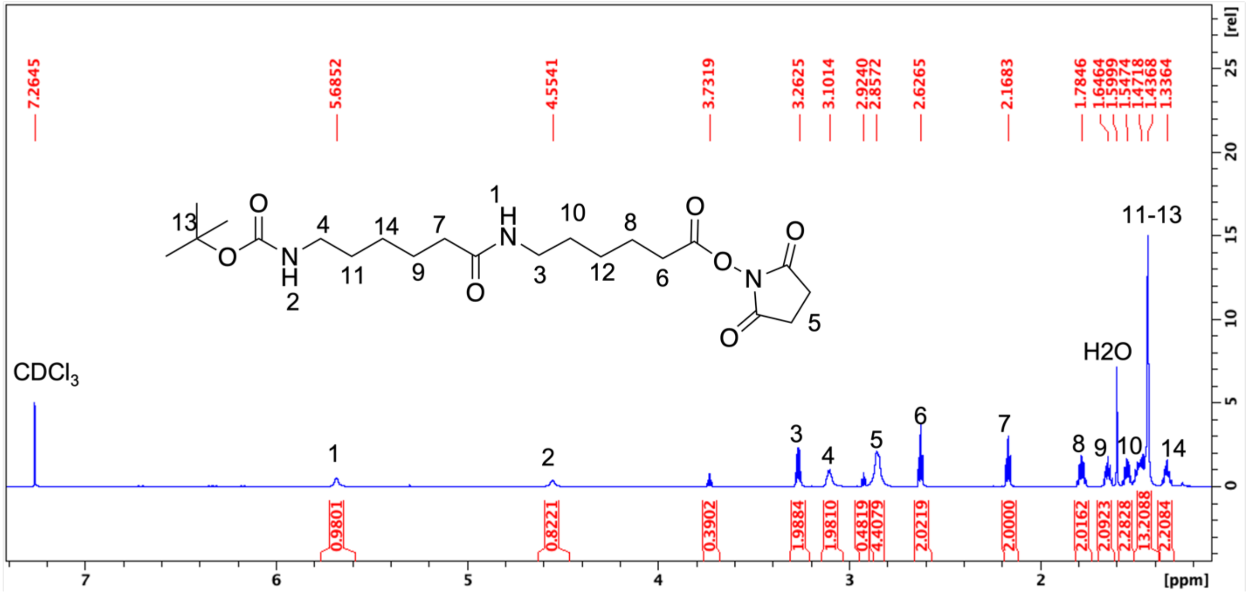

Figure S89. ^{1}H-NMR of **BocNH-(Hexanoic)$_2$-NHS Ester (27)**, $CDCl_3$.

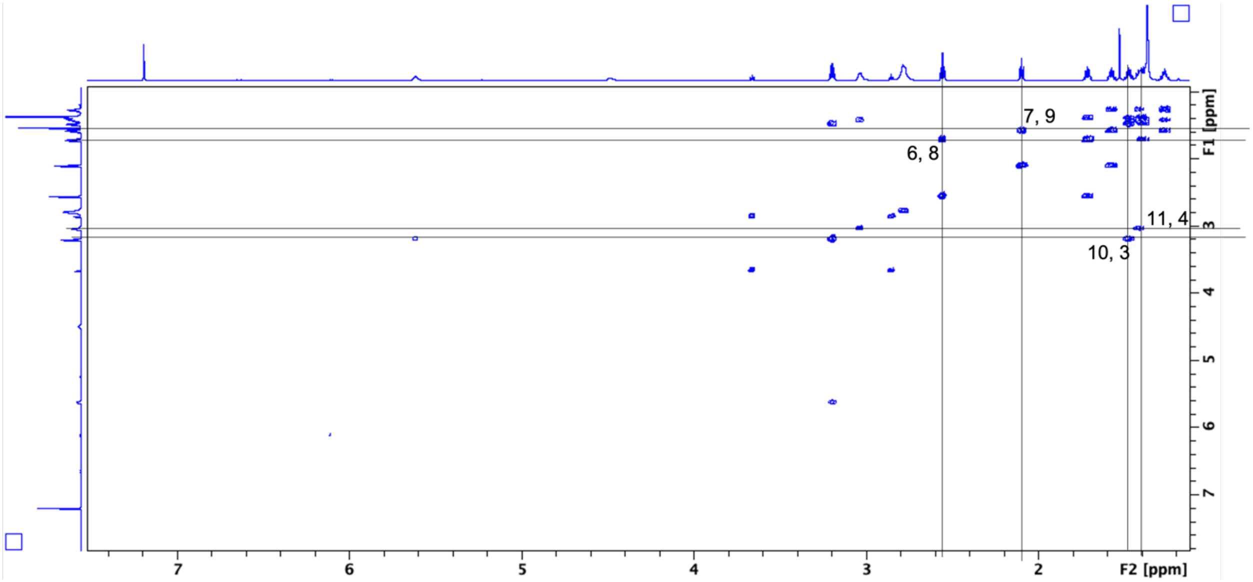

Figure S90. ^{1}H^{1}H-COSY NMR of **BocNH-(Hexanoic)$_2$-NHS Ester (27)**, $CDCl_3$.

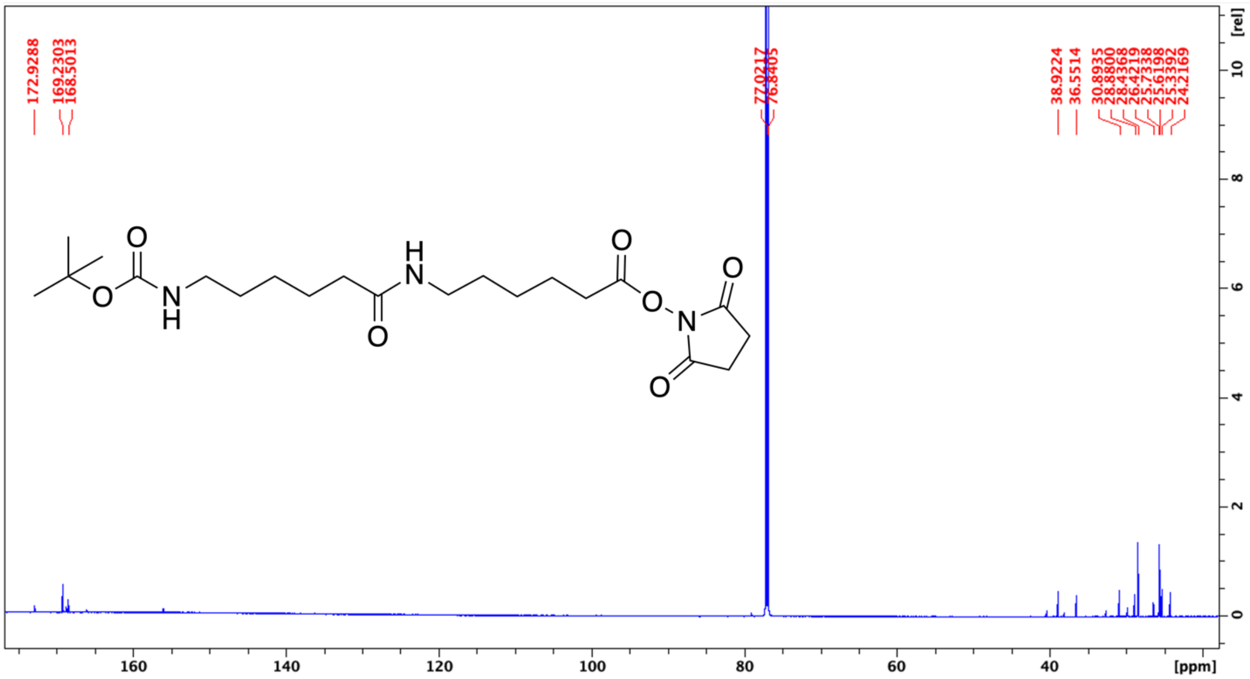

Figure S91. ^{13}C-NMR of **BocNH-(Hexanoic)$_2$-NHS Ester (27)**, $CDCl_3$.

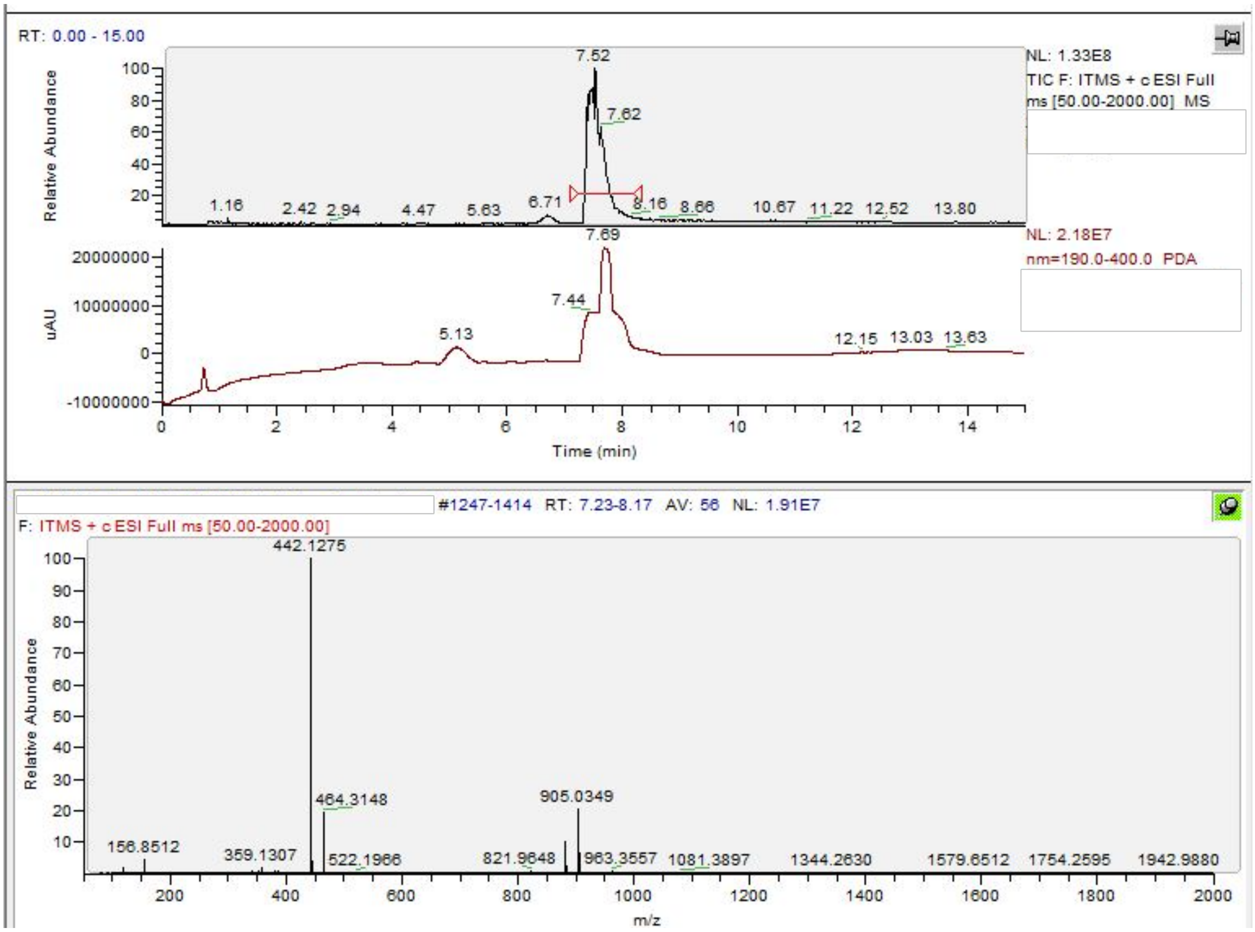

Figure S92. LC-MS trace of post column purified compound **BocNH-(Hexanoic)$_2$-NHS Ester (27)**. Top - TIC trace. Bottom - selected mass spectrum from TIC highlighted by red bar (retention time 7.23 – 8.17). Middle - UV chromatogram.

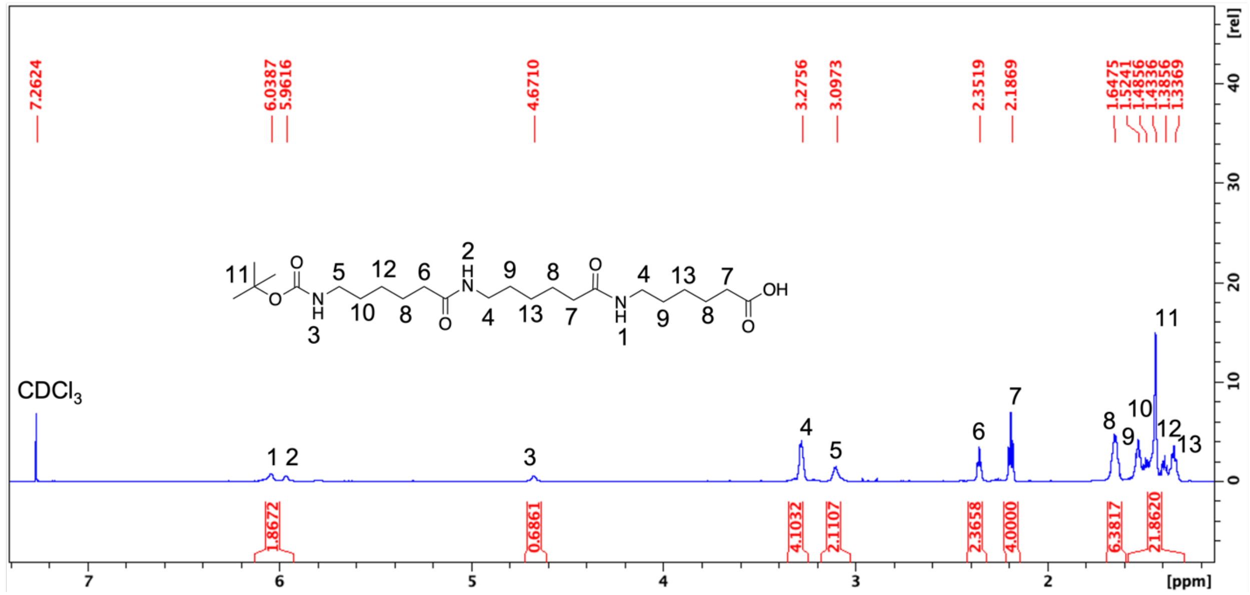

Figure S93. ^{1}H-NMR of **BocNH-(Hexanoic)$_3$-Carboxylic Acid (28),** $CDCl_3$.

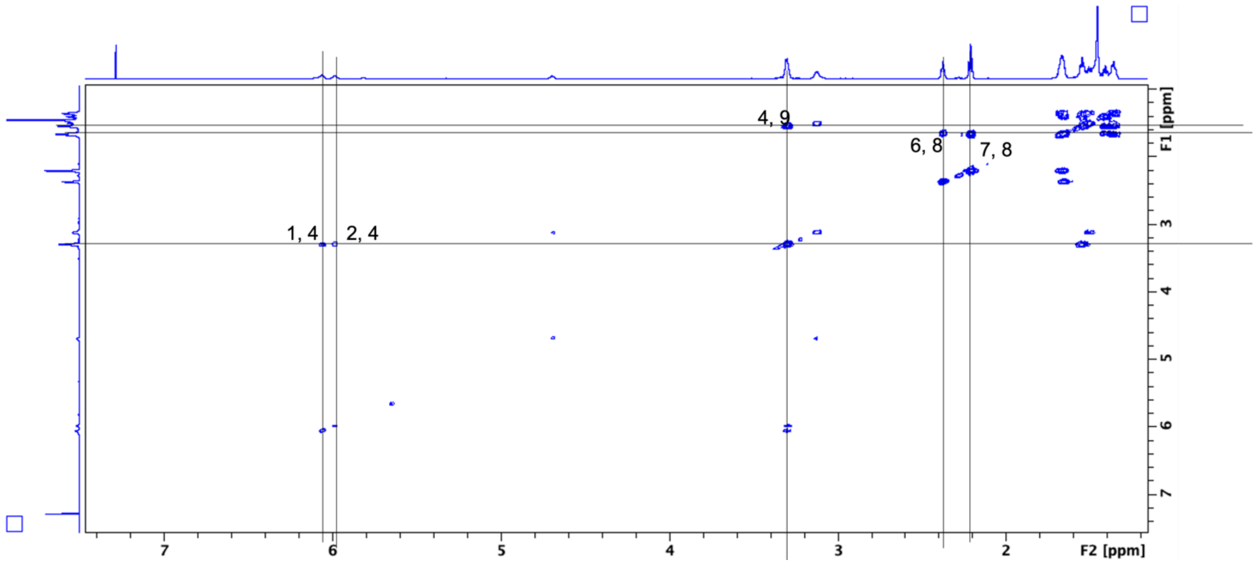

Figure S94. ^{1}H^{1}H-COSY NMR of **BocNH-(Hexanoic)$_3$-Carboxylic Acid (28),** $CDCl_3$.

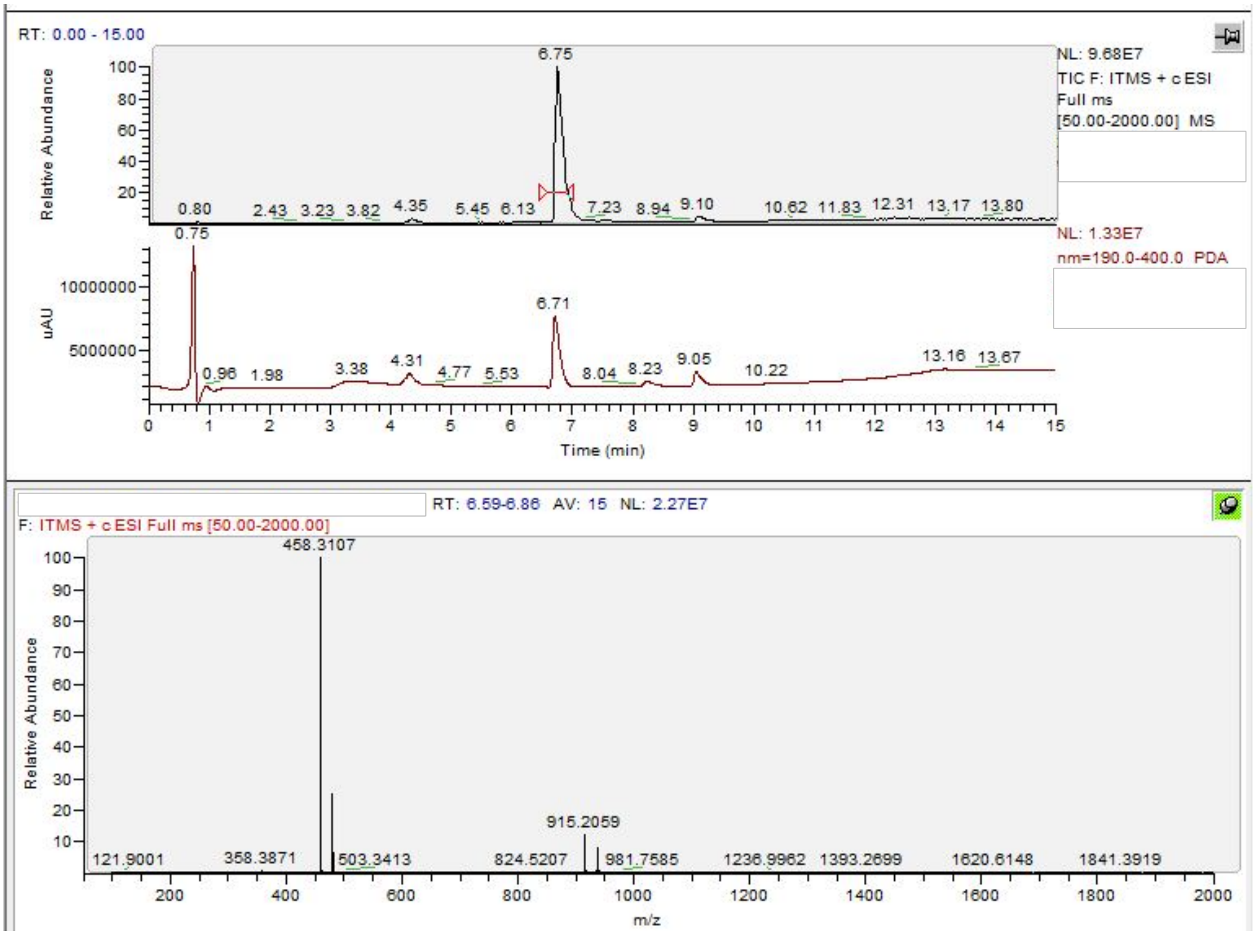

Figure S95. LC-MS trace of post column purified compound **BocNH-(Hexanoic)$_3$-Carboxylic Acid (28)**. Top - TIC trace. Bottom - selected mass spectrum from TIC highlighted by red bar (retention time 6.59 – 6.86). Middle - UV chromatogram.

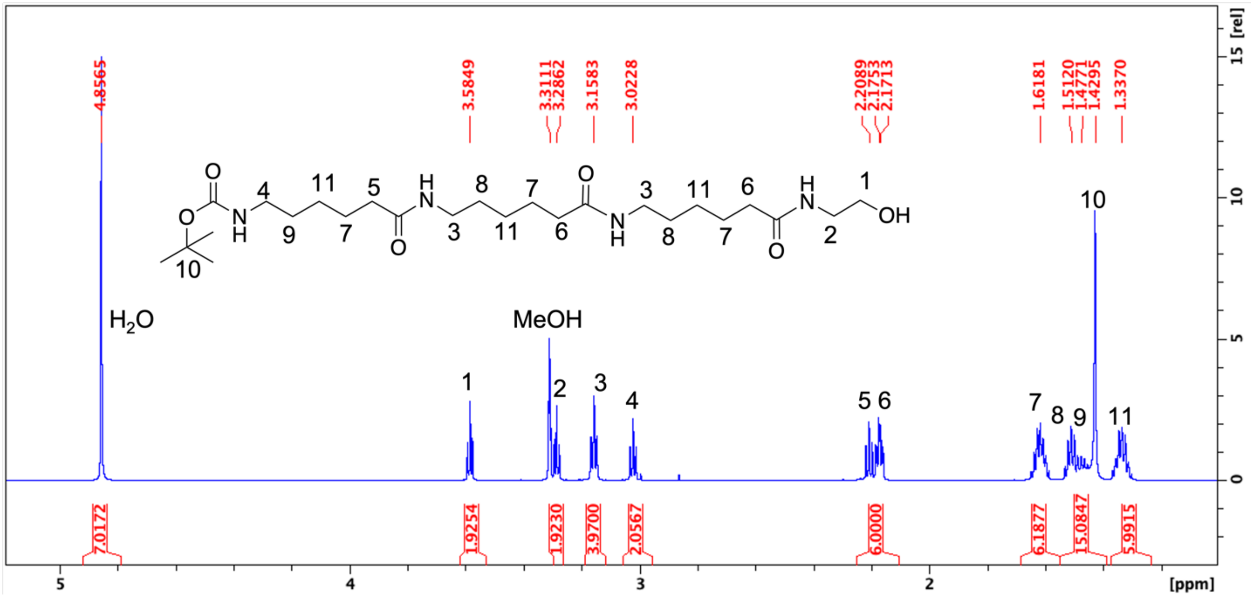

Figure S96. ^{1}H-NMR of **BocNH-(Hexanoic)$_3$-OH (29)**, MeOD.

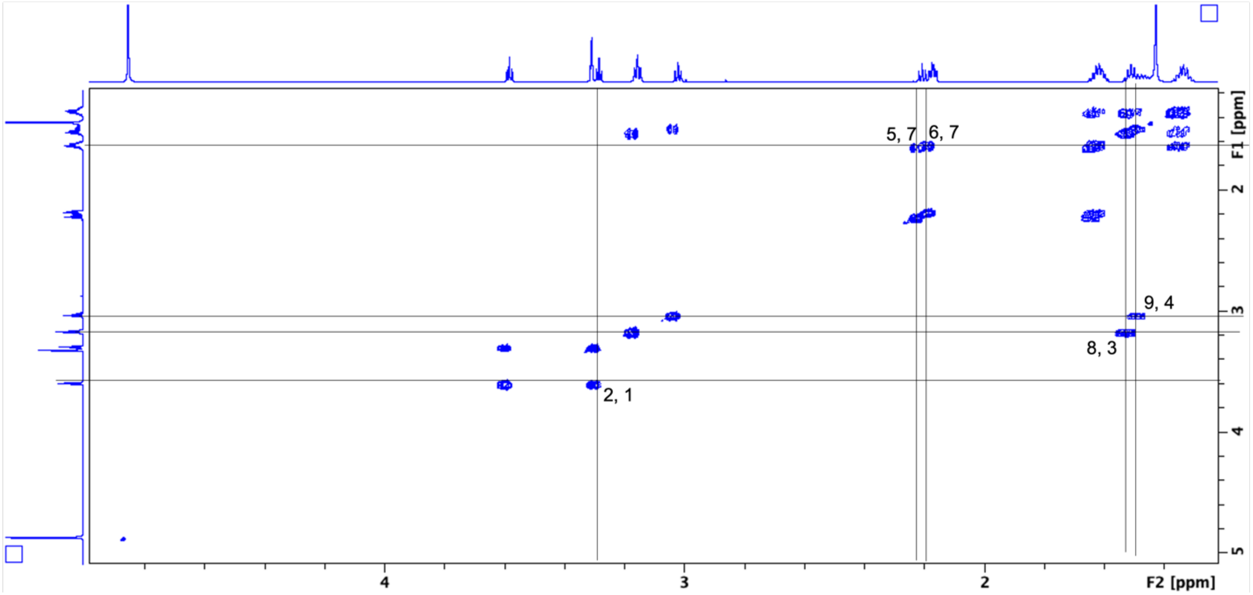

Figure S97. ^{1}H^{1}H-COSY NMR of **BocNH-(Hexanoic)$_3$-OH (29)**, MeOD.

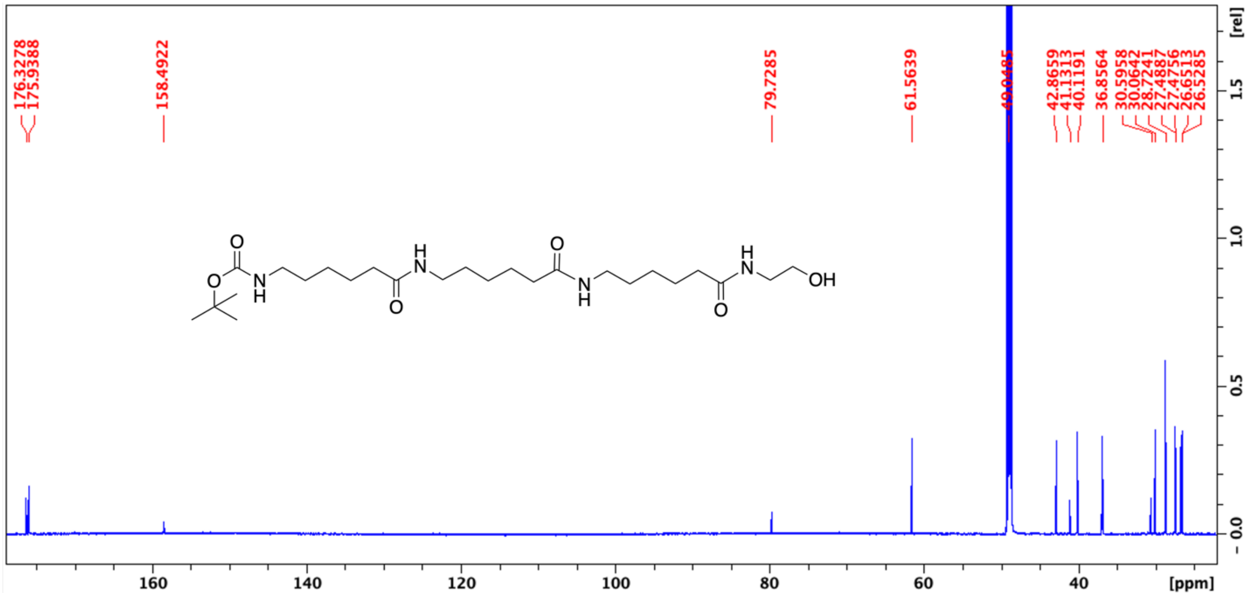

Figure S98. ^{13}C-NMR of **BocNH-(Hexanoic)$_3$-OH (29)**, MeOD.

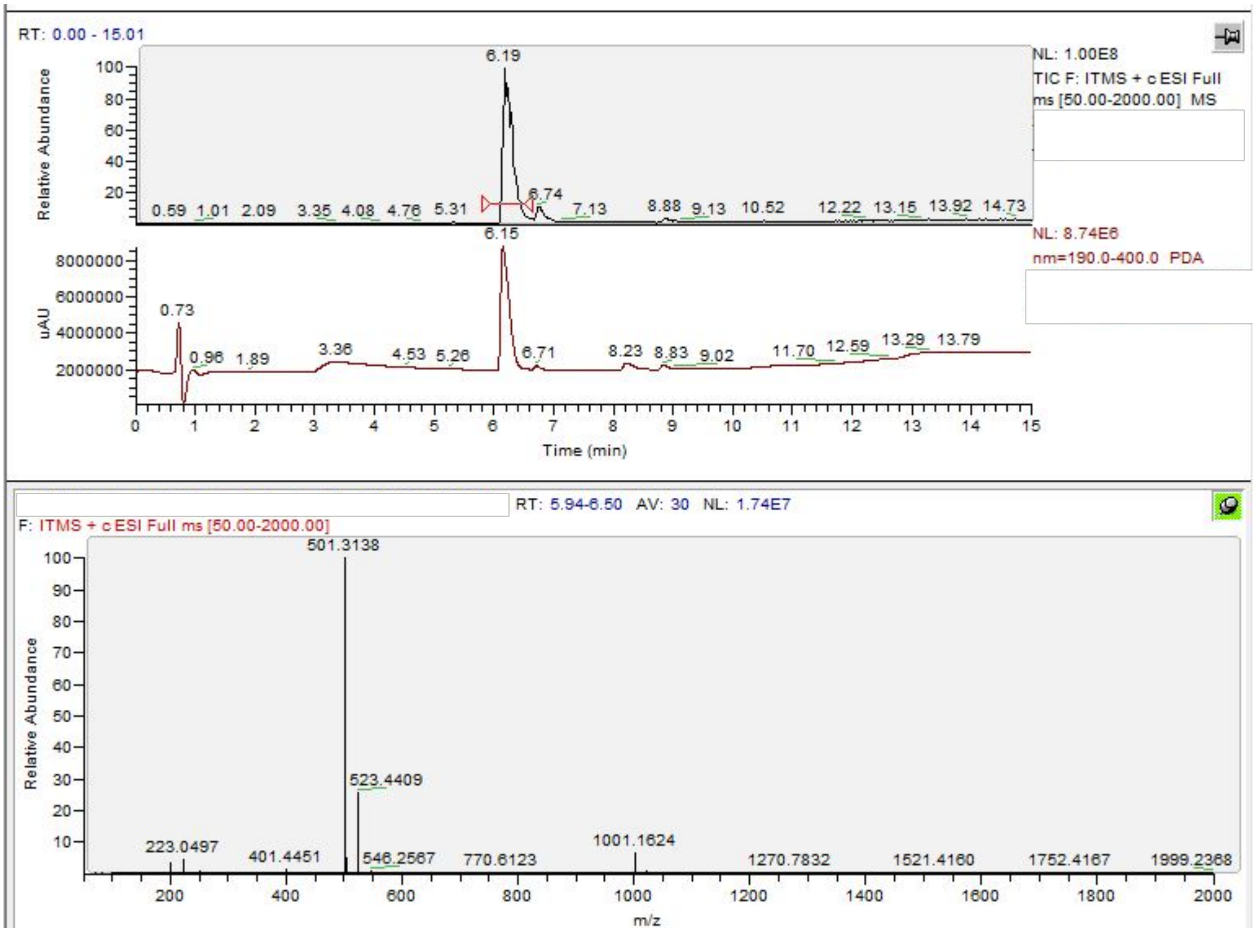

Figure S99. LC-MS trace of post column purified compound **BocNH-(Hexanoic)$_3$-OH (29)**. Top - TIC trace. Bottom - selected mass spectrum from TIC highlighted by red bar (retention time 5.94 – 6.50). Middle - UV chromatogram.

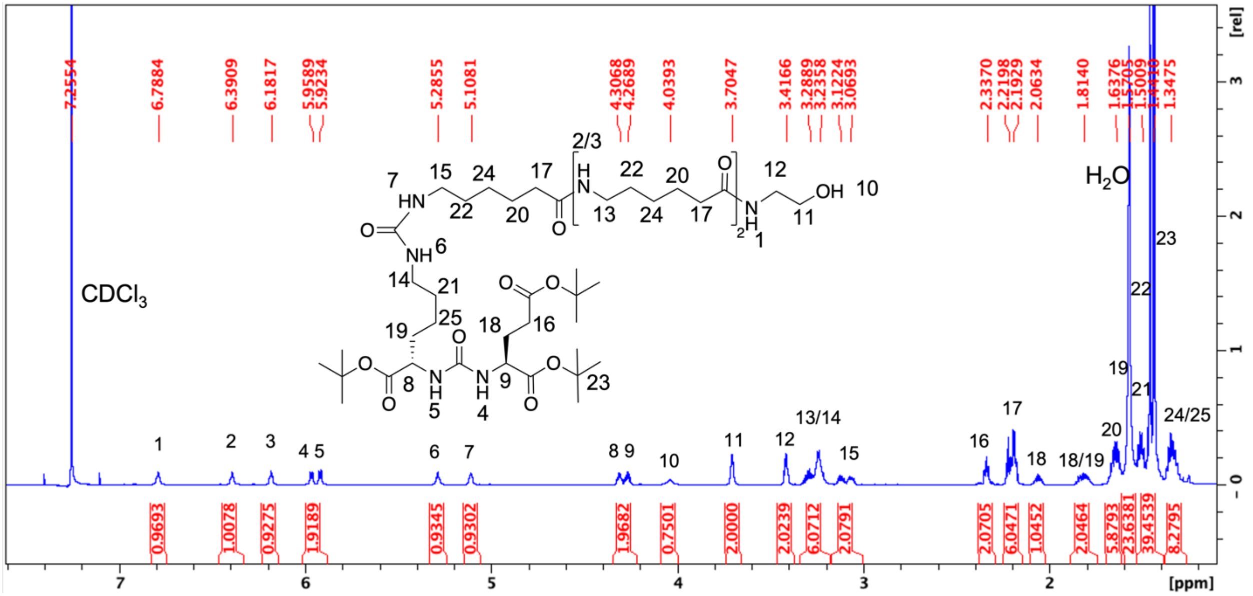

Figure S100. ^{1}H-NMR of **Tri-t-butyl-GUL-Alkyl-OH (31),** $CDCl_3$.

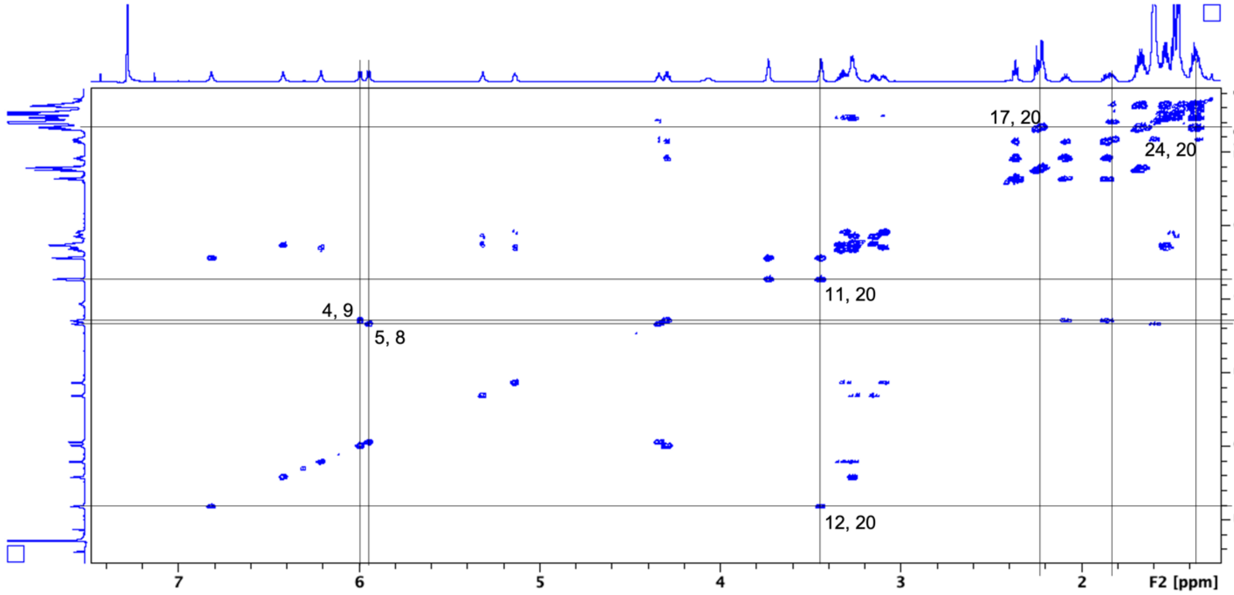

Figure S101. ^{1}H^{1}H-COSY NMR of **Tri-t-butyl-GUL-Alkyl-OH (31),** $CDCl_3$.

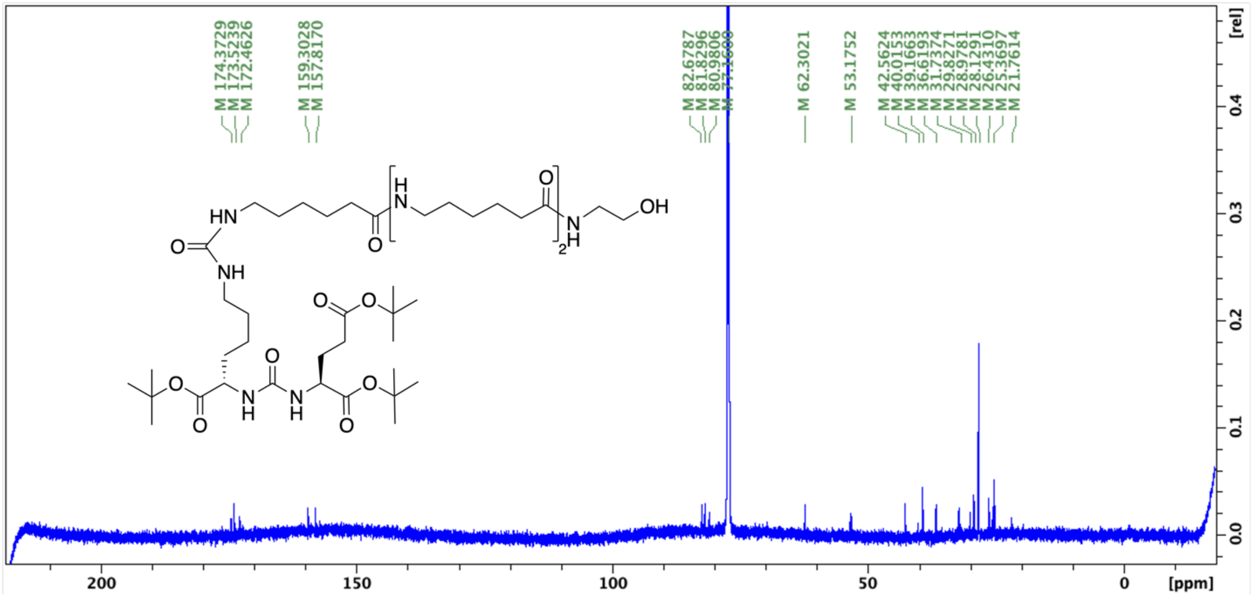

Figure S102. ^{13}C-NMR of **Tri-t-butyl-GUL-Alkyl-OH (31),** $CDCl_3$.

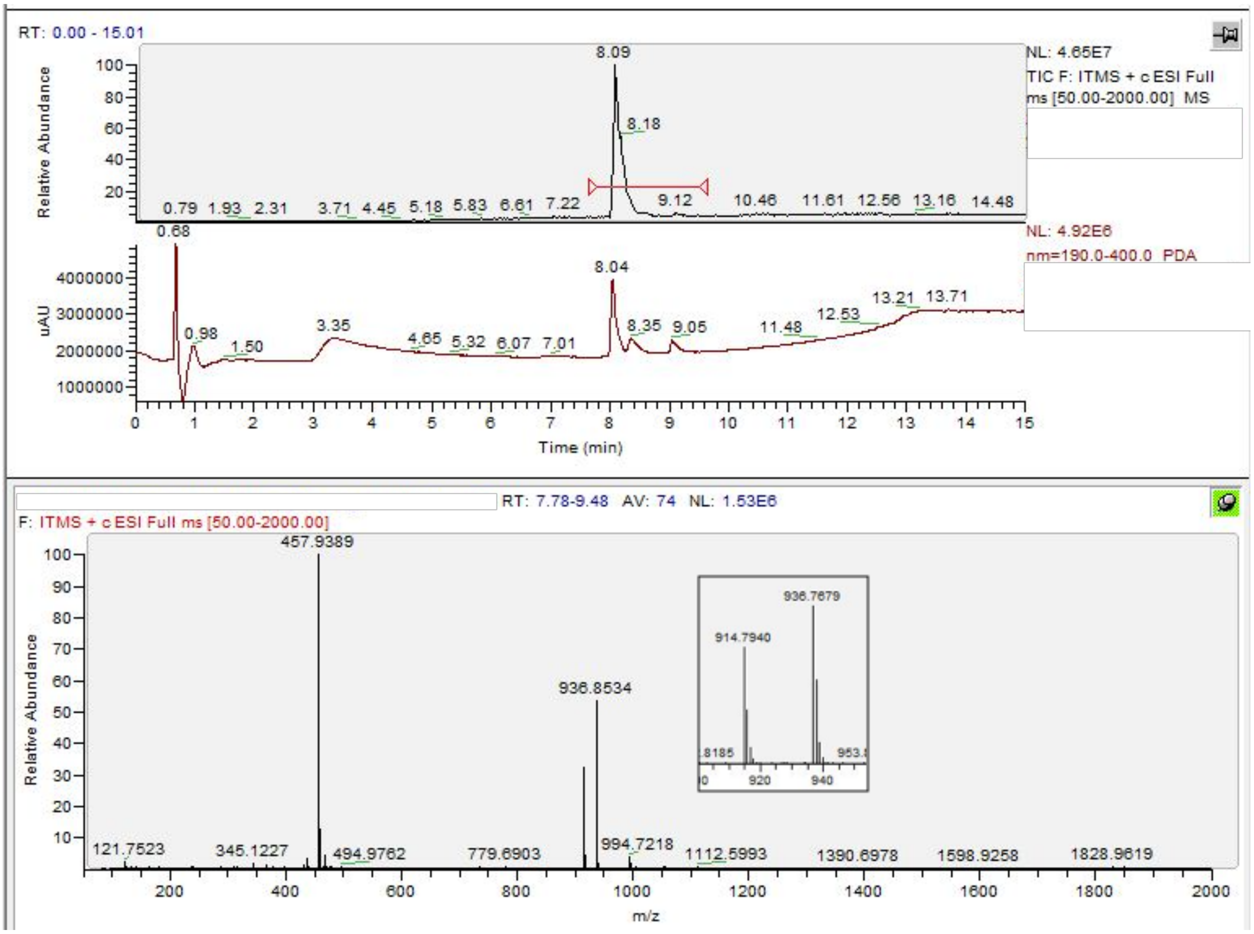

Figure S103. LC-MS trace of post column purified compound **Tri-t-butyl-GUL-Alkyl-OH (31).** Top - TIC trace. Bottom - selected mass spectrum from TIC highlighted by red bar (retention time 5.02 – 5.20). The $[M+Na]^+$ mass is seen (936.7679). To highlight the $[M+H]^+$ peak, a box highlighting m/z around 920 is superimposed. Middle - UV chromatogram.

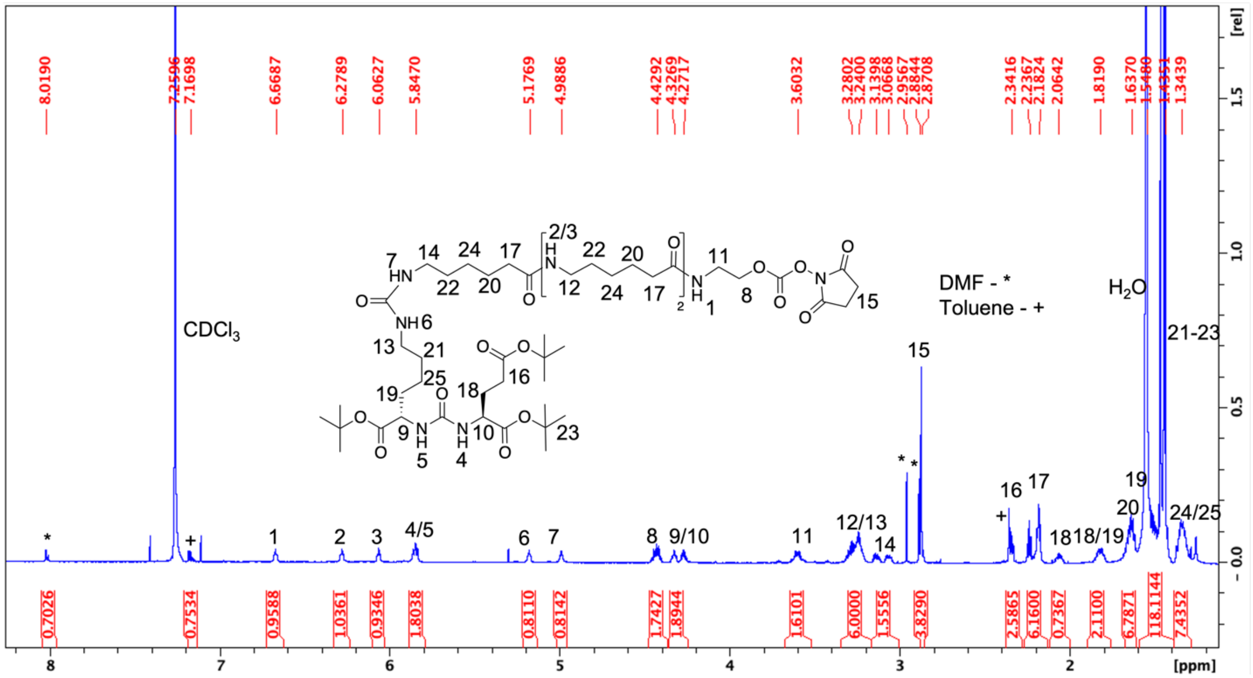

Figure S104. ^{1}H-NMR of **Tri-t-butyl-GUL-Alkyl-NHS (32)**, $CDCl_3$.

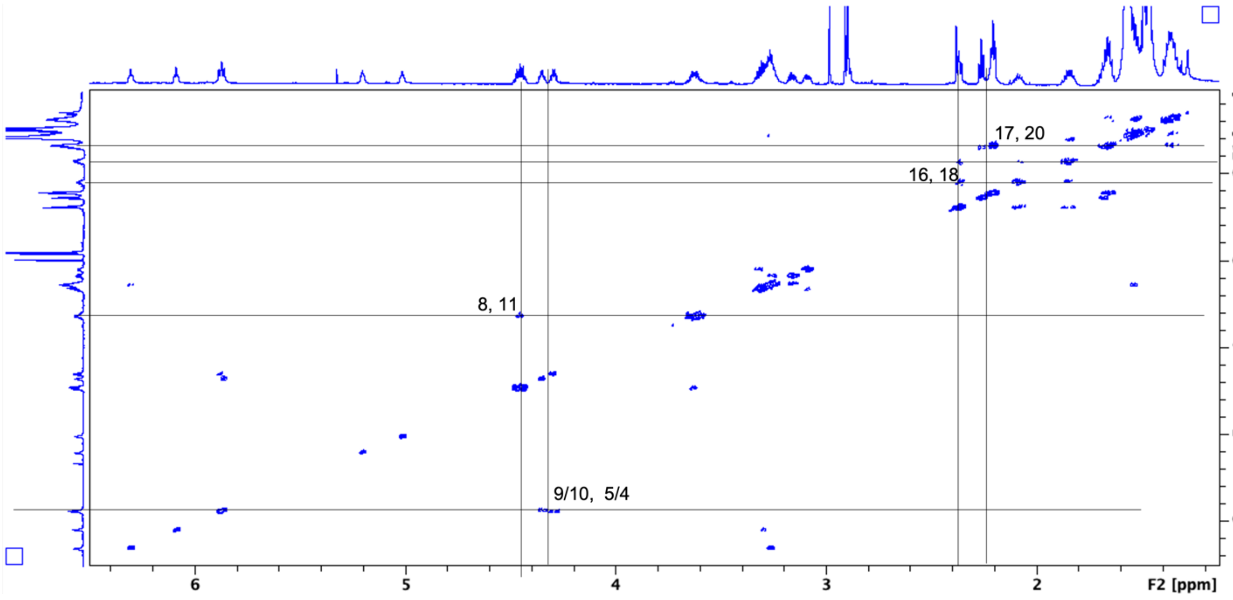

Figure S105. ^{1}H^{1}H-COSY NMR of **Tri-t-butyl-GUL-Alkyl-NHS (32)**, $CDCl_3$.

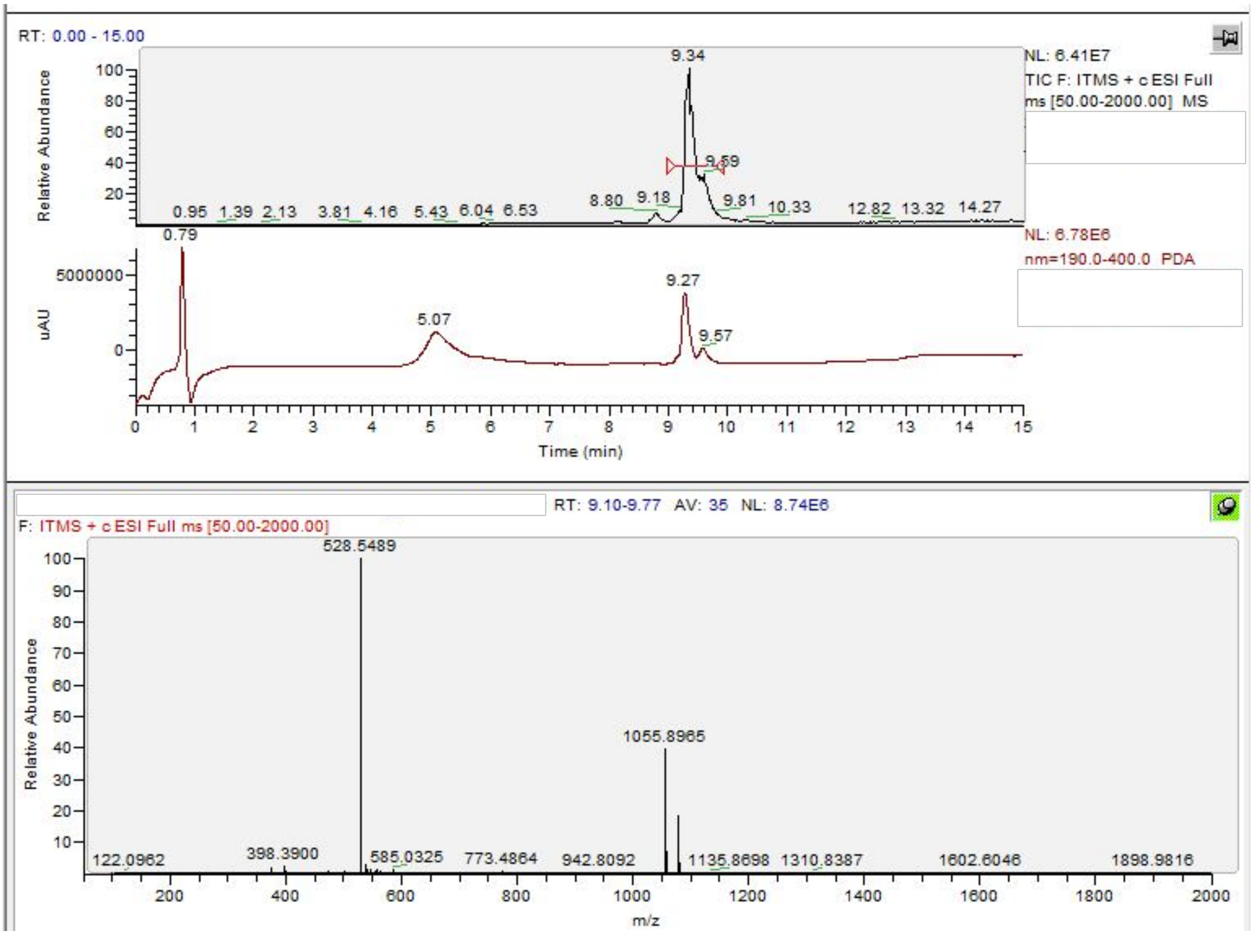

Figure S106. LC-MS trace of post column purified compound **Tri-t-butyl-GUL-Alkyl-NHS (32)**. Top - TIC trace. Bottom - selected mass spectrum from TIC highlighted by red bar (retention time 9.10 – 9.77). Middle - UV chromatogram.

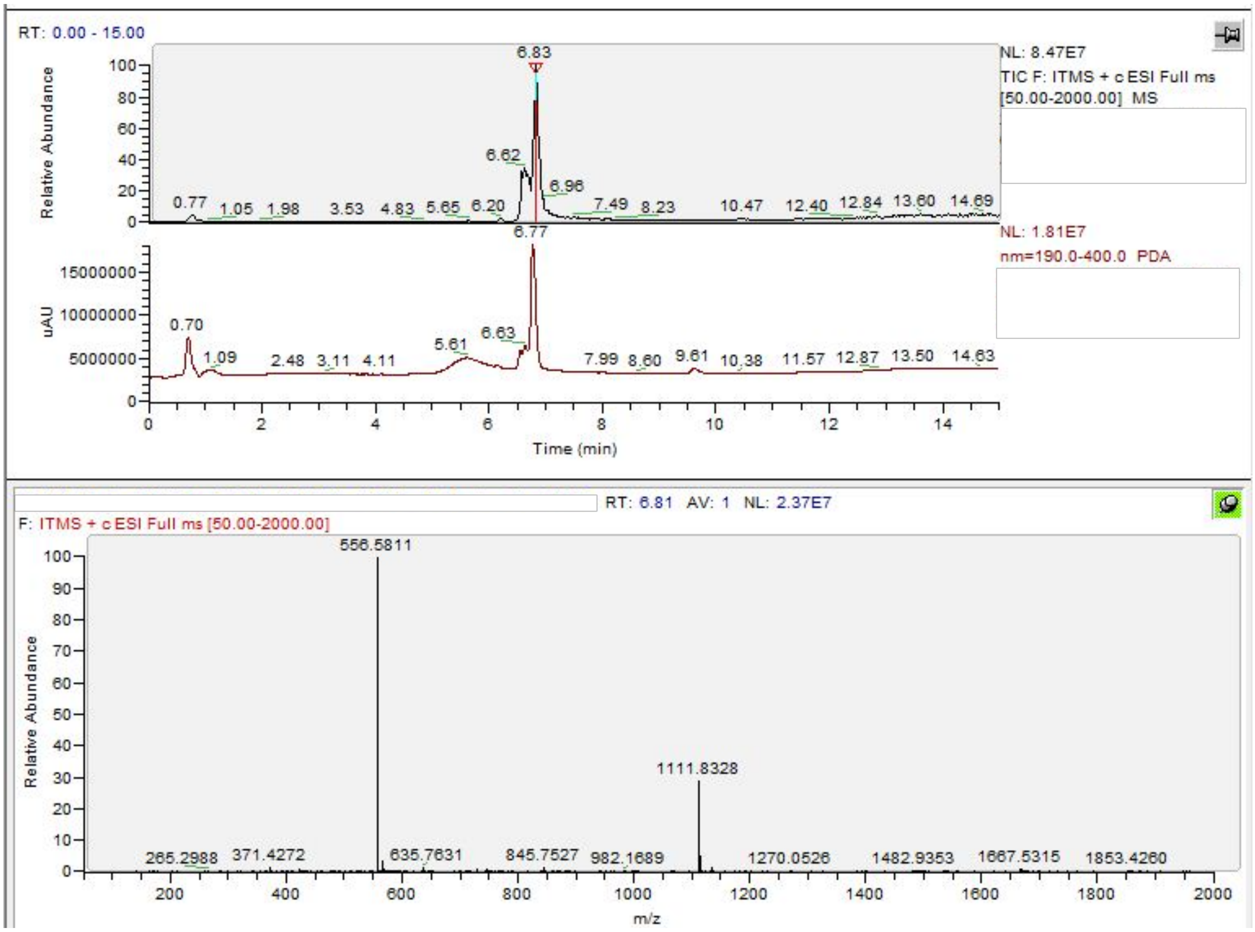

Figure S107. LC-MS trace of post column purified compound **GUL-Alkyl-cARM (10)**. Top - TIC trace. Bottom - selected mass spectrum from TIC highlighted by red bar (retention time 6.81). Middle - UV chromatogram. A slight amount of hydrolyzed product is present at retention time 6.63 (UV).

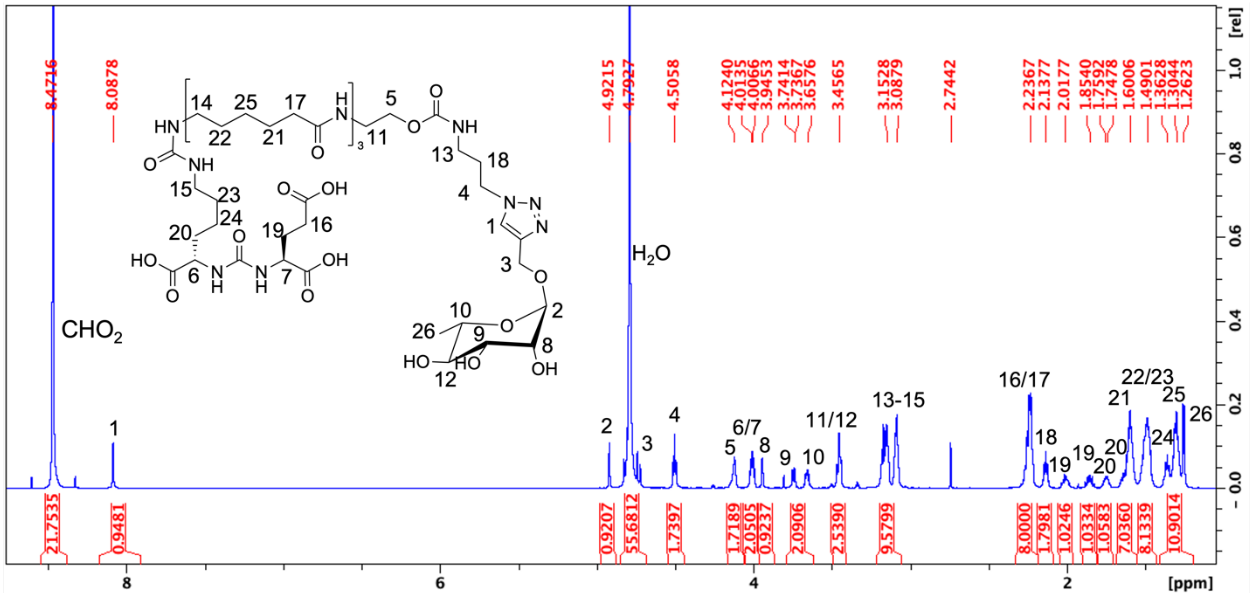

Figure S108. ^{1}H-NMR of **GUL-Alkyl-ARM (11)**, D_2O_salt.

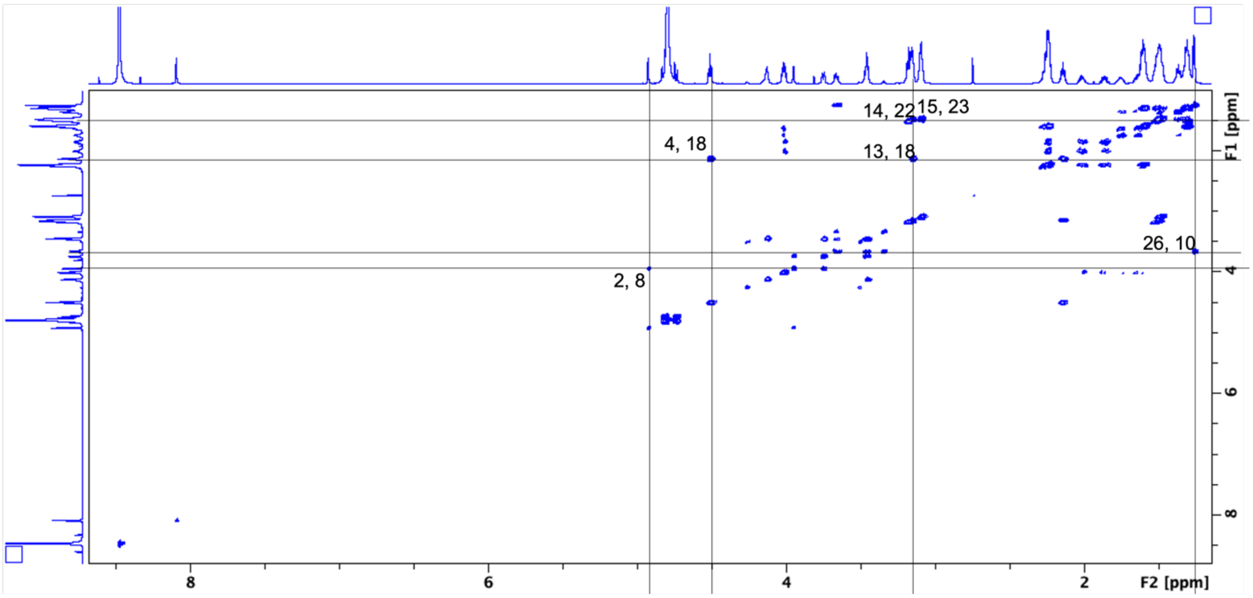

Figure S109. ^{1}H^{1}H-COSY NMR of **GUL-Alkyl-ARM (11)**, D_2O_salt.

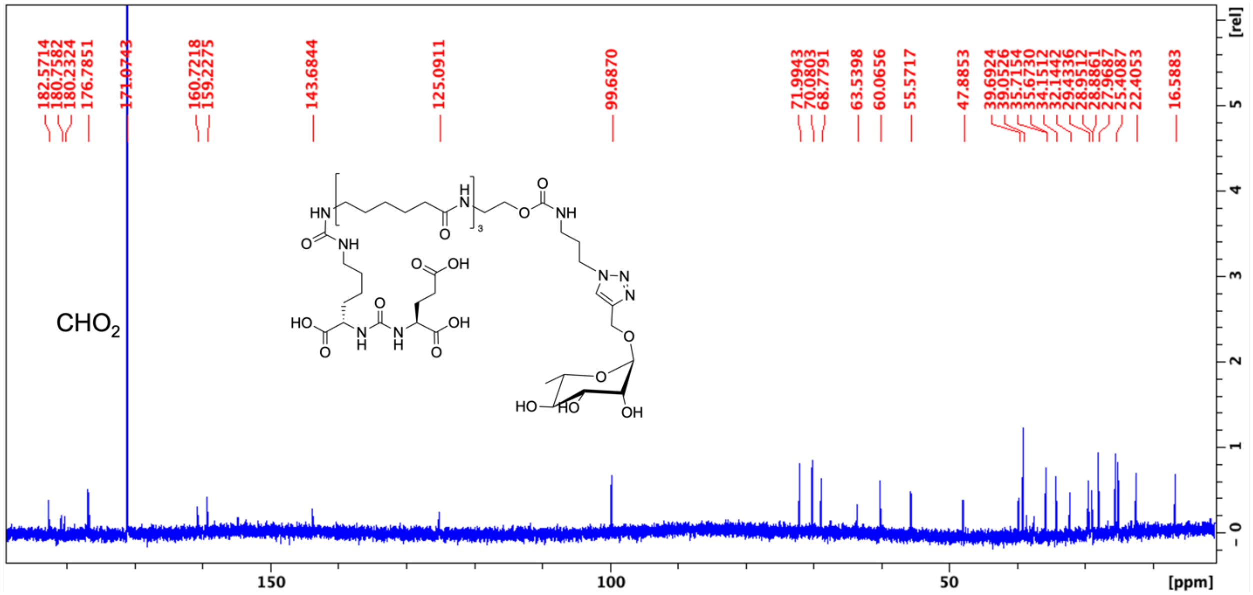

Figure S110. ^{13}C-NMR of **GUL-Alkyl-ARM (11)**, D_2O_salt.

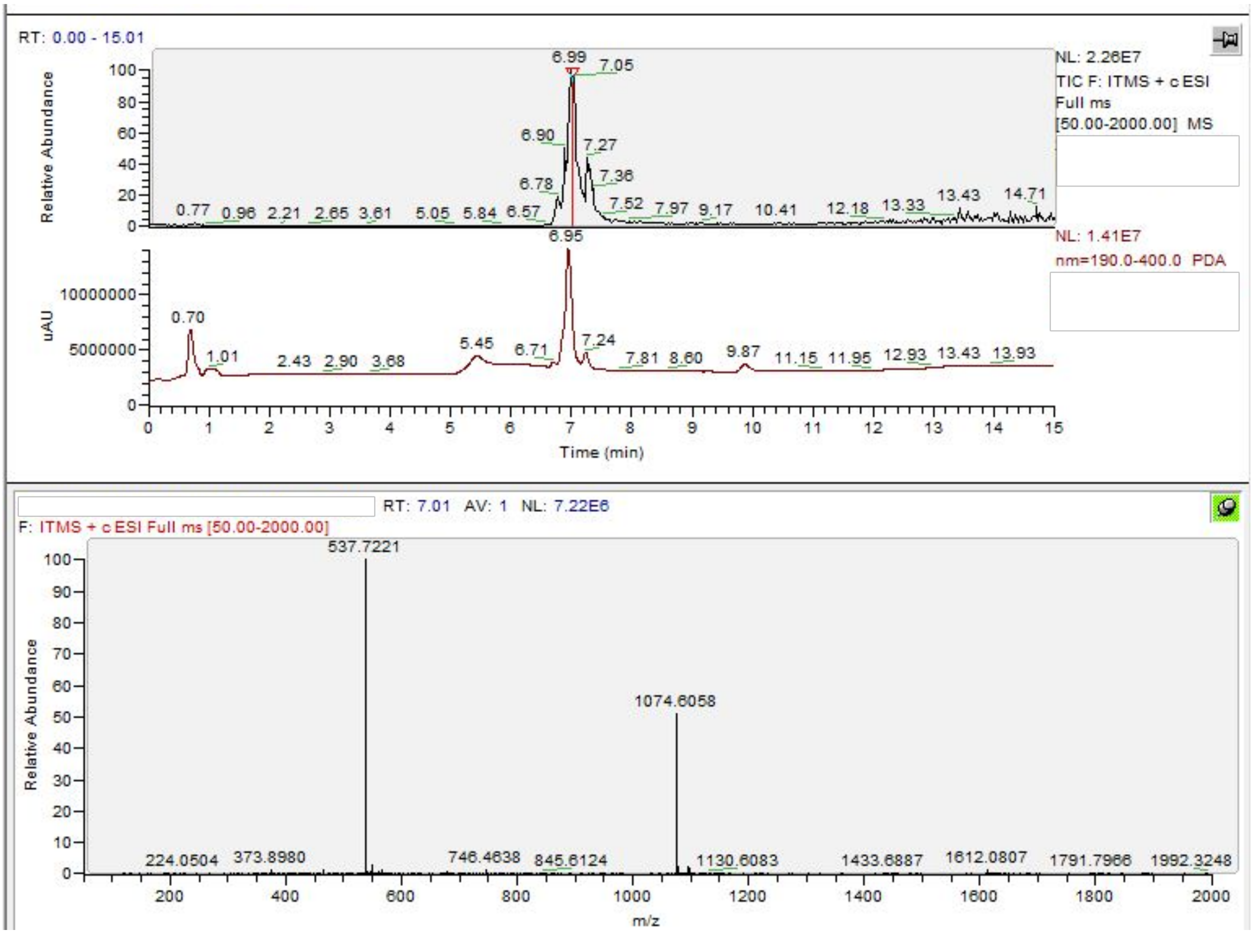

Figure S111. LC-MS trace of post column purified compound **GUL-Alkyl-ARM (11)**. Top - TIC trace. Bottom - selected mass spectrum from TIC highlighted by red bar (retention time 7.01). Middle - UV chromatogram.

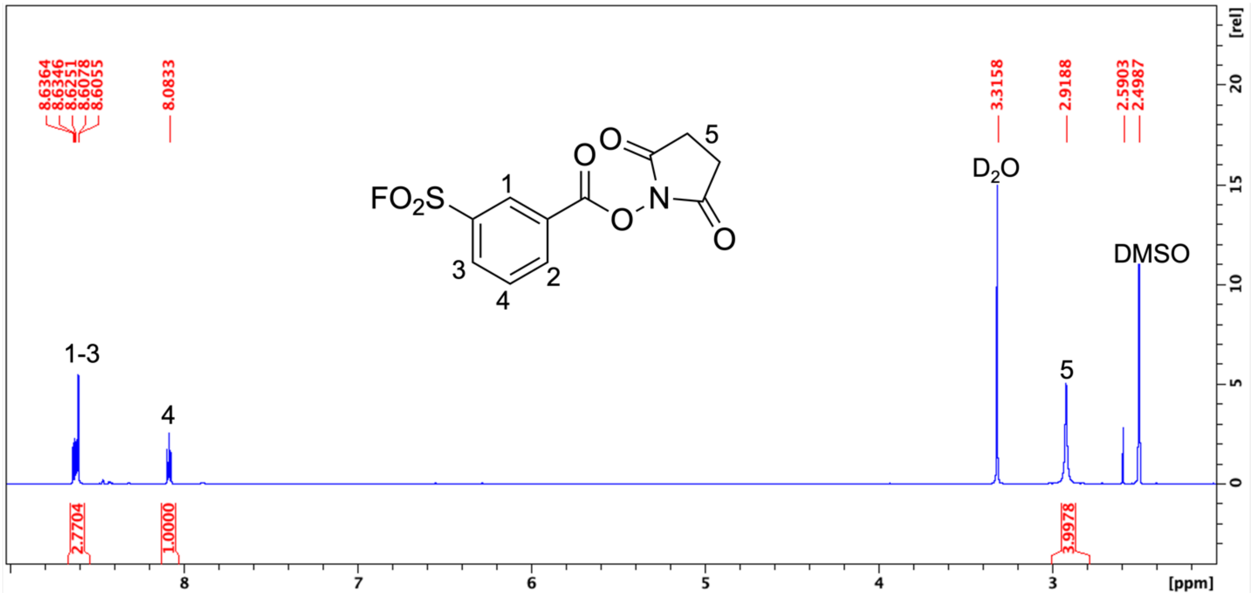

Figure S112. ^{1}H-NMR of **Aryl-SO_2F-NHS (33)**, DMSO.

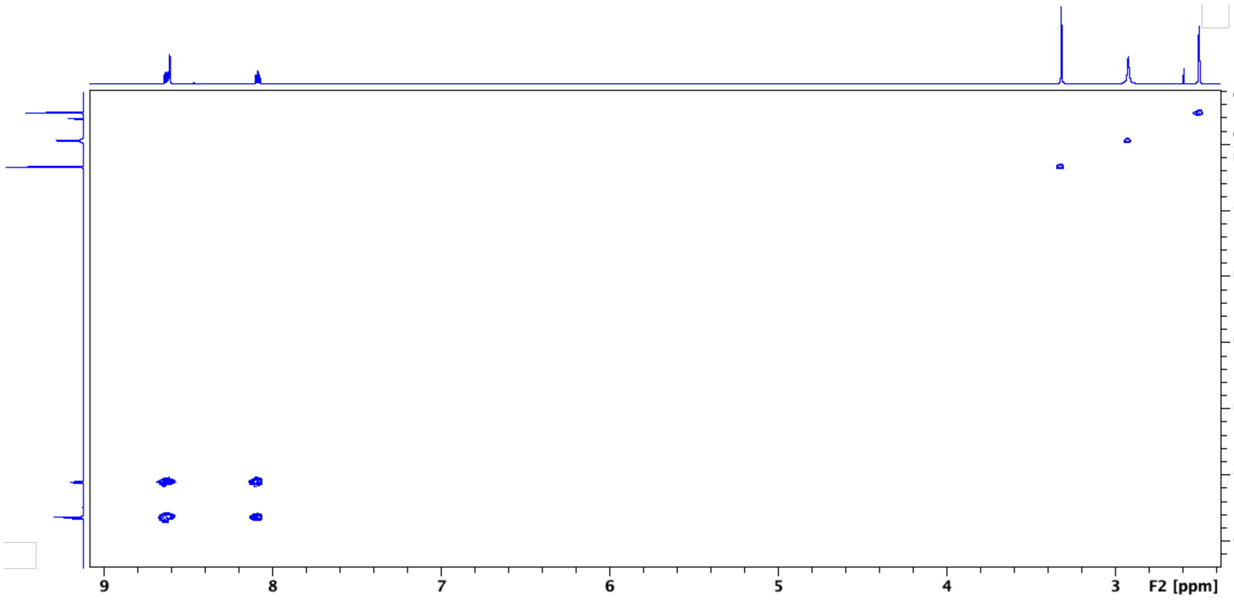

Figure S113. ^{1}H^{1}H-COSY NMR of **Aryl-SO_2F-NHS (33)**, DMSO.

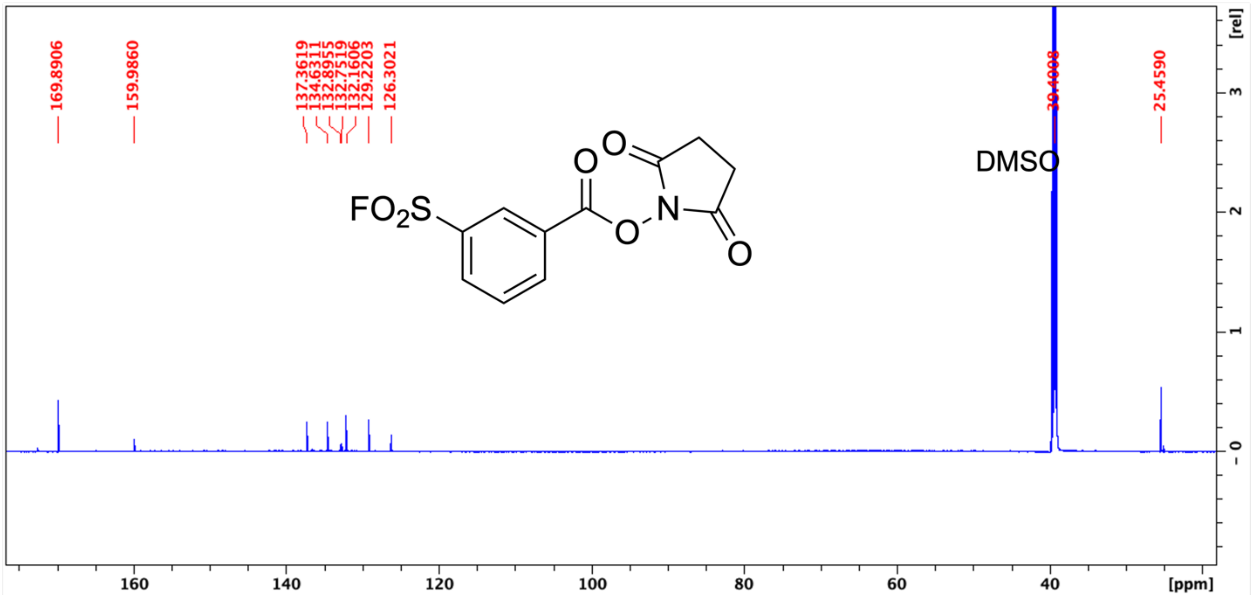

Figure S114. ^{13}C-NMR of **Aryl-SO$_2$F-NHS (33)**, DMSO.

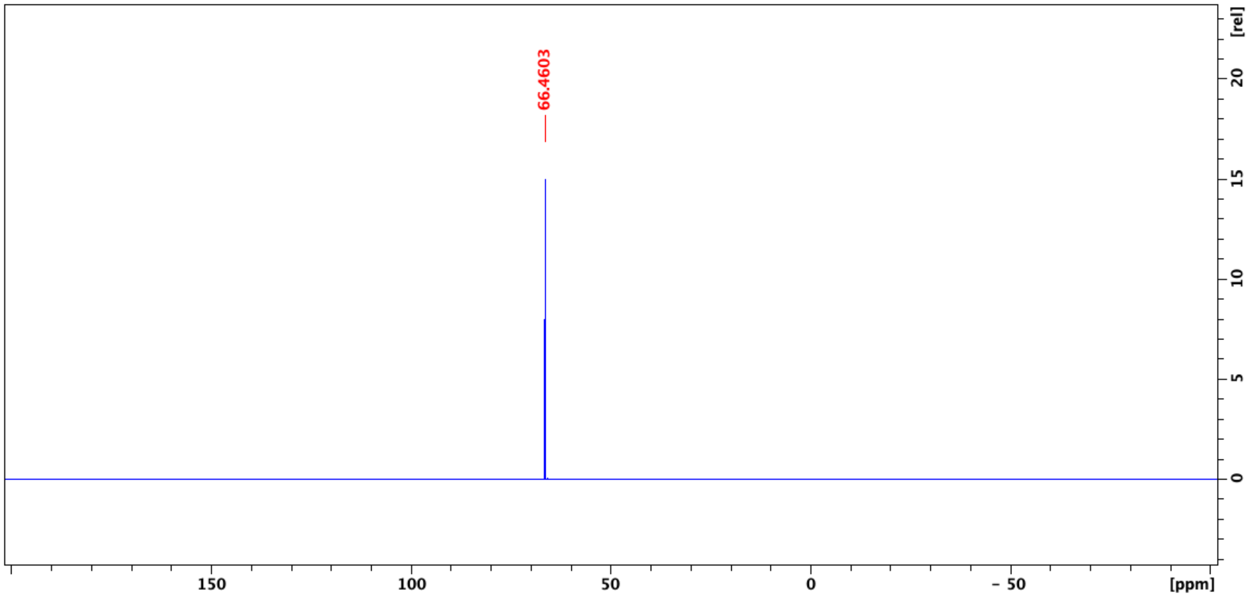

Figure S115. ^{1}F-NMR of **Aryl-SO$_2$F-NHS (33)**, DMSO.

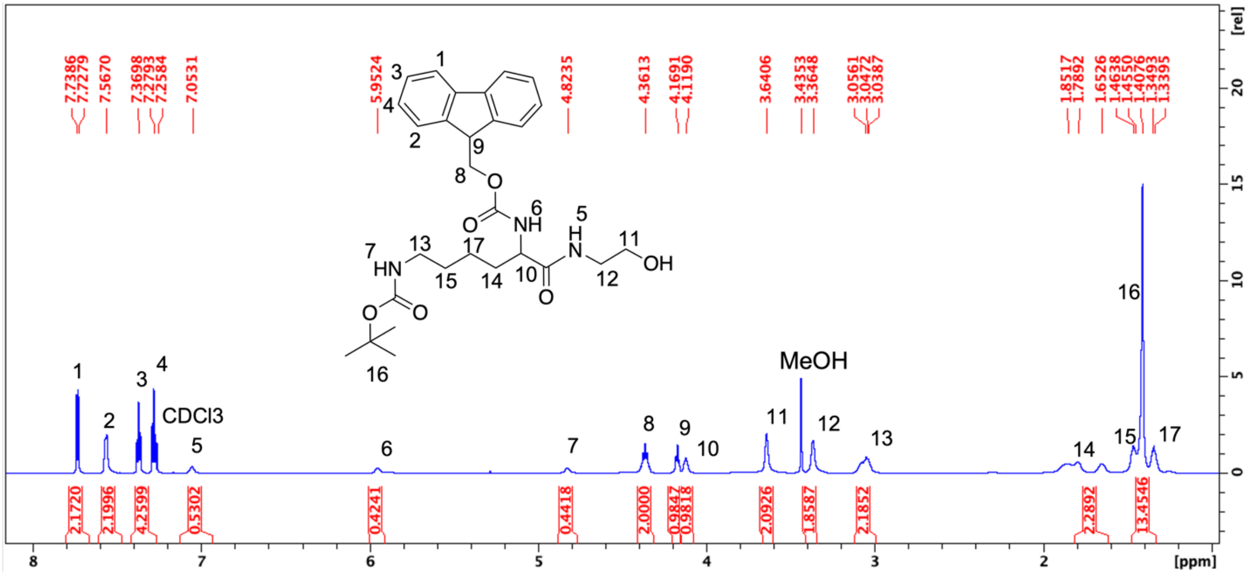

Figure S116. ^{1}H-NMR of **ε-NHBoc-α-Fmoc-Lysine-Ethanol (34)**, $CDCl_3$.

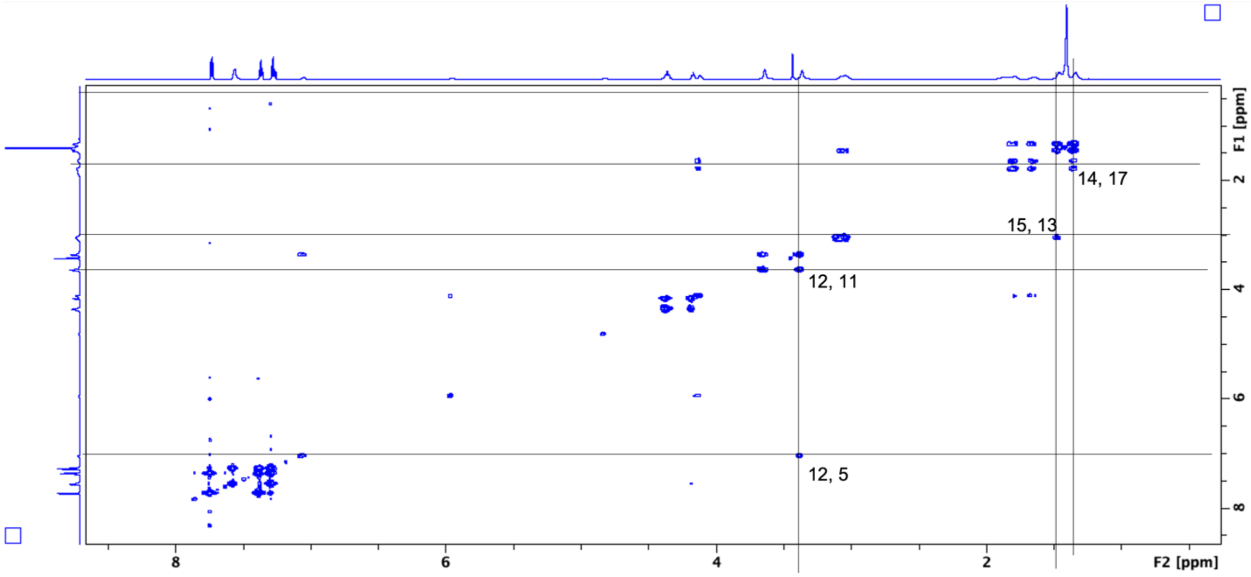

Figure S117. ^{1}H^{1}H-COSY NMR of **ε-NHBoc-α-Fmoc-Lysine-Ethanol (34)**, $CDCl_3$.

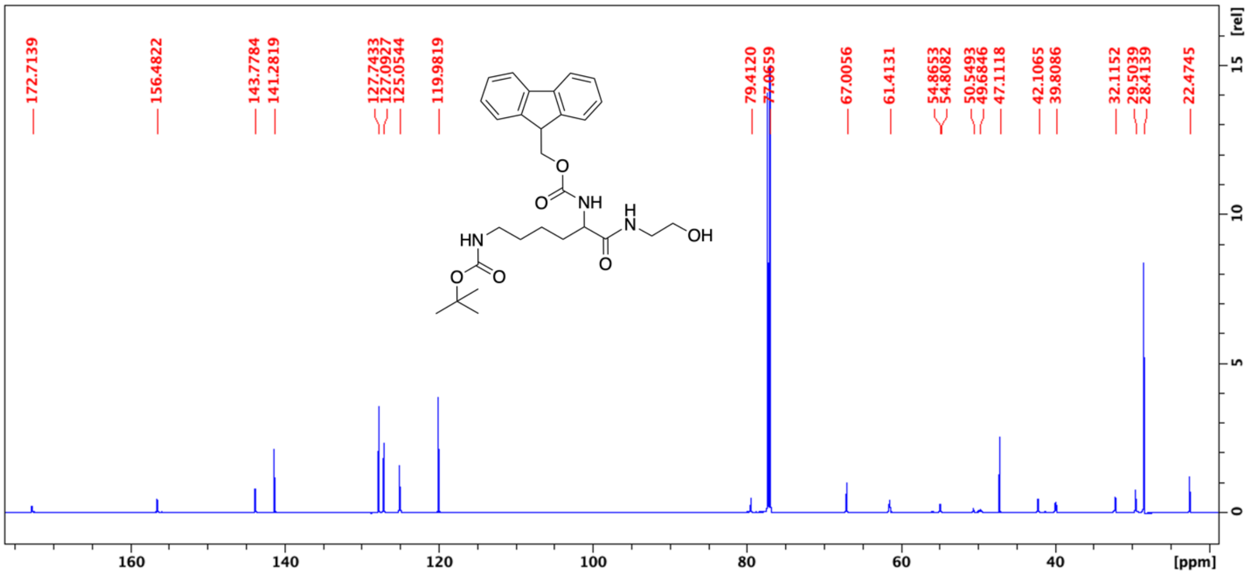

Figure S118. ^{13}C-NMR of **ε-NHBoc-α-Fmoc-Lysine-Ethanol (34)**, $CDCl_3$.

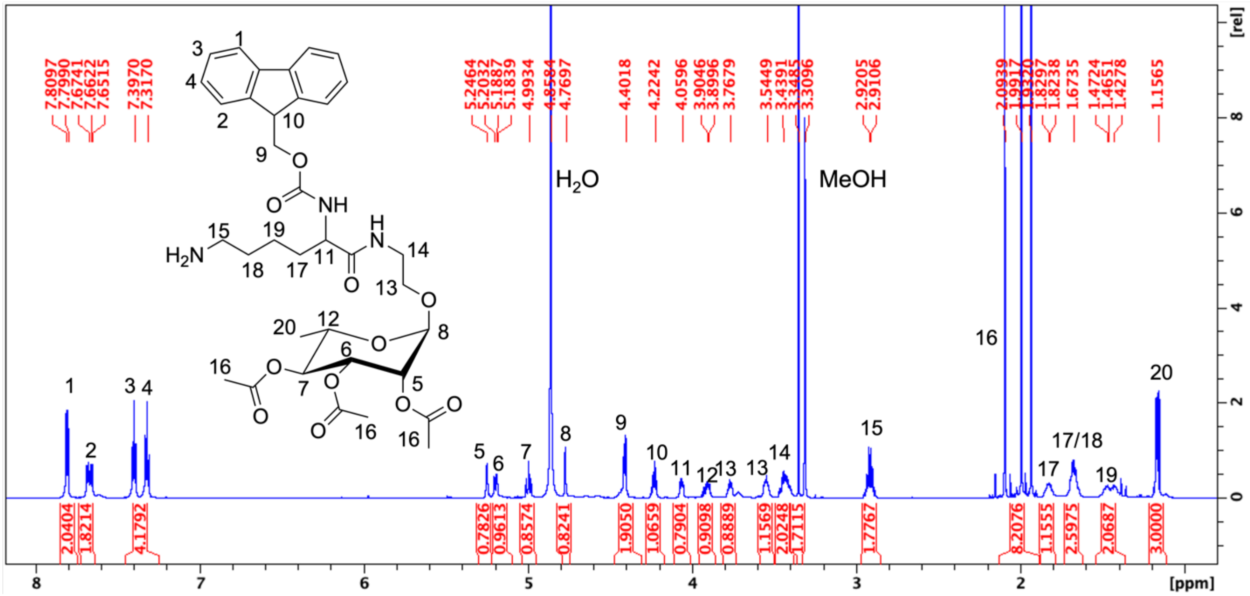

Figure S119. ^{1}H-NMR of **Peracetyl-α-O-Rhamnose-Lysine-α-Fmoc-ε-NH_2 (35)**, MeOD.

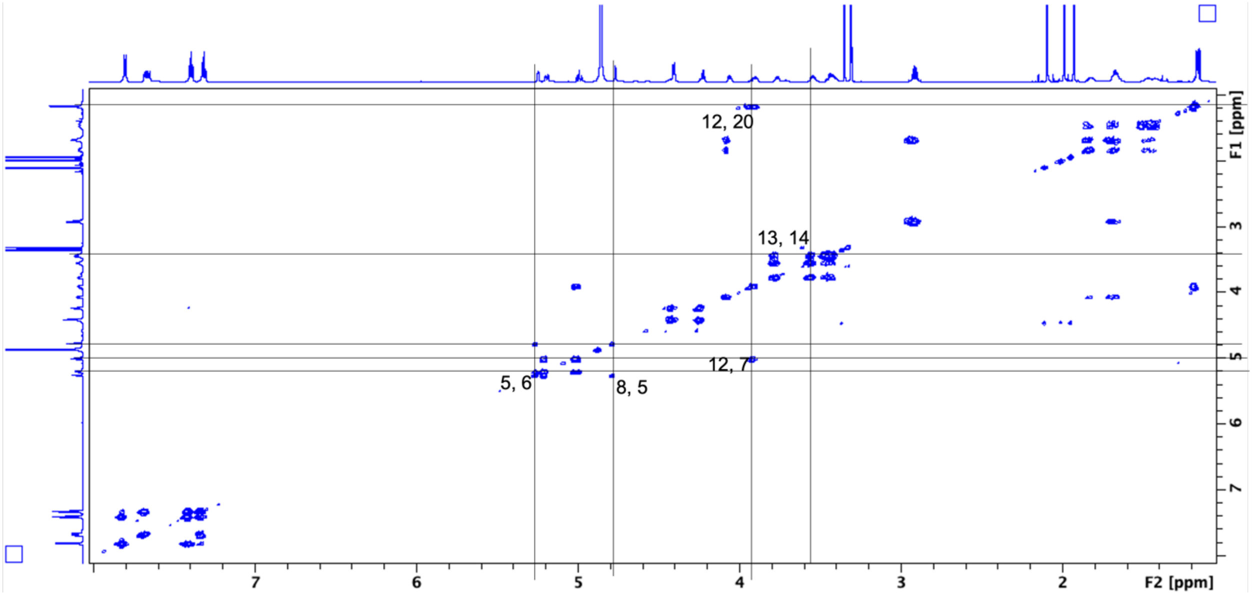

Figure S120. ^{1}H^{1}H-COSY NMR of **<u>Peracetyl-α-O-Rhamnose-Lysine-α-Fmoc-ε-NH$_2$ (35)</u>**, MeOD.

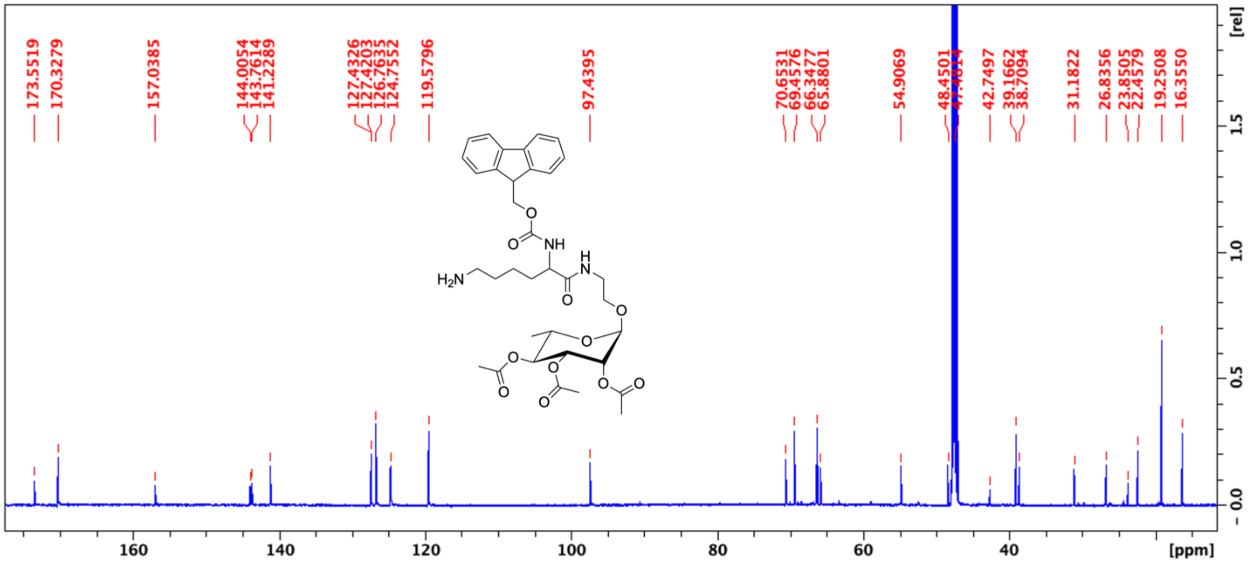

Figure S121. ^{13}C-NMR of **<u>Peracetyl-α-O-Rhamnose-Lysine-α-Fmoc-ε-NH$_2$ (35)</u>**, MeOD.

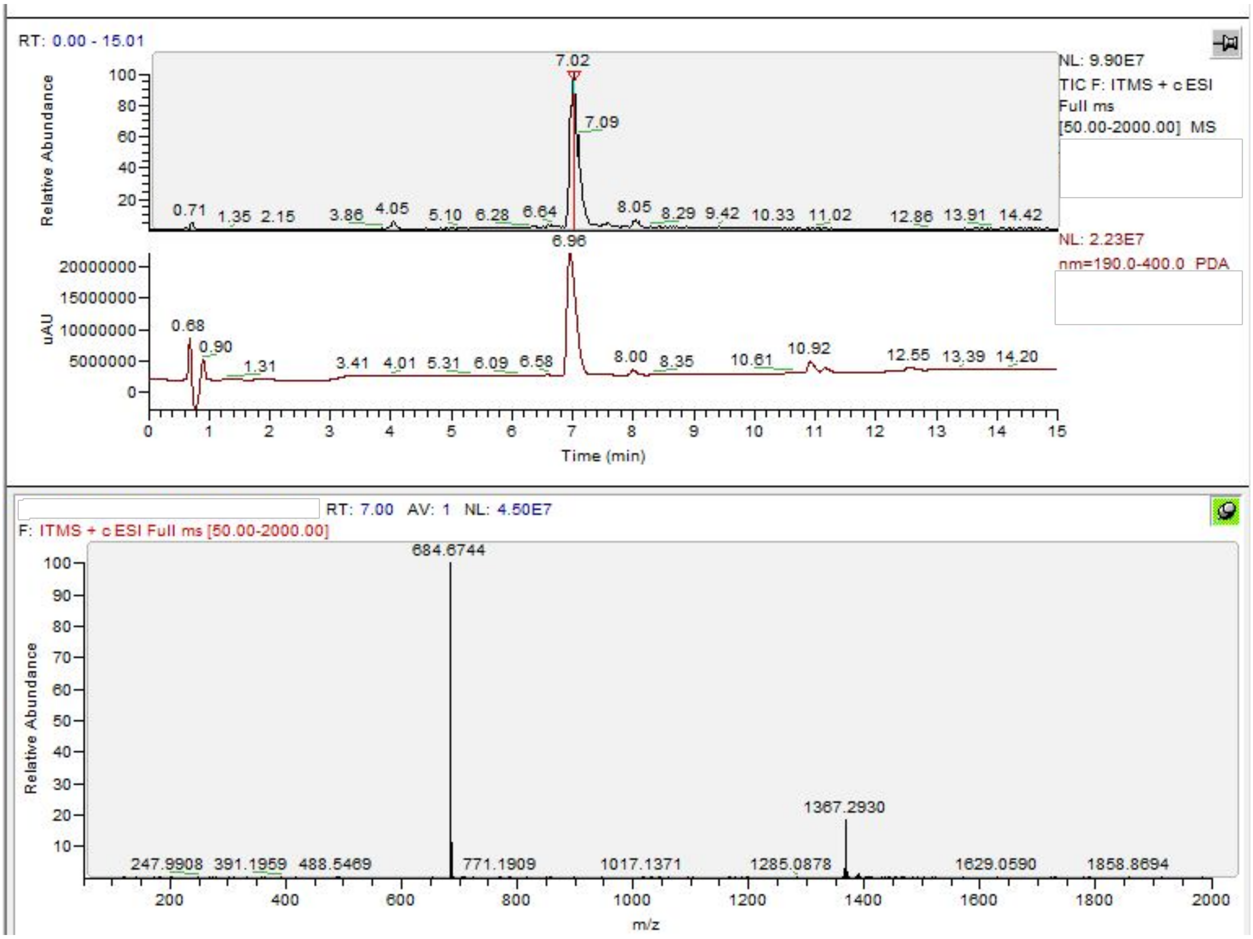

Figure S122. LC-MS trace of post column purified compound **Peracetyl-α-O-Rhamnose-Lysine-α-Fmoc-ε-NH_2 (35)**. Top - TIC trace. Bottom - selected mass spectrum from TIC highlighted by red bar (retention time 7.00). Middle - UV chromatogram.

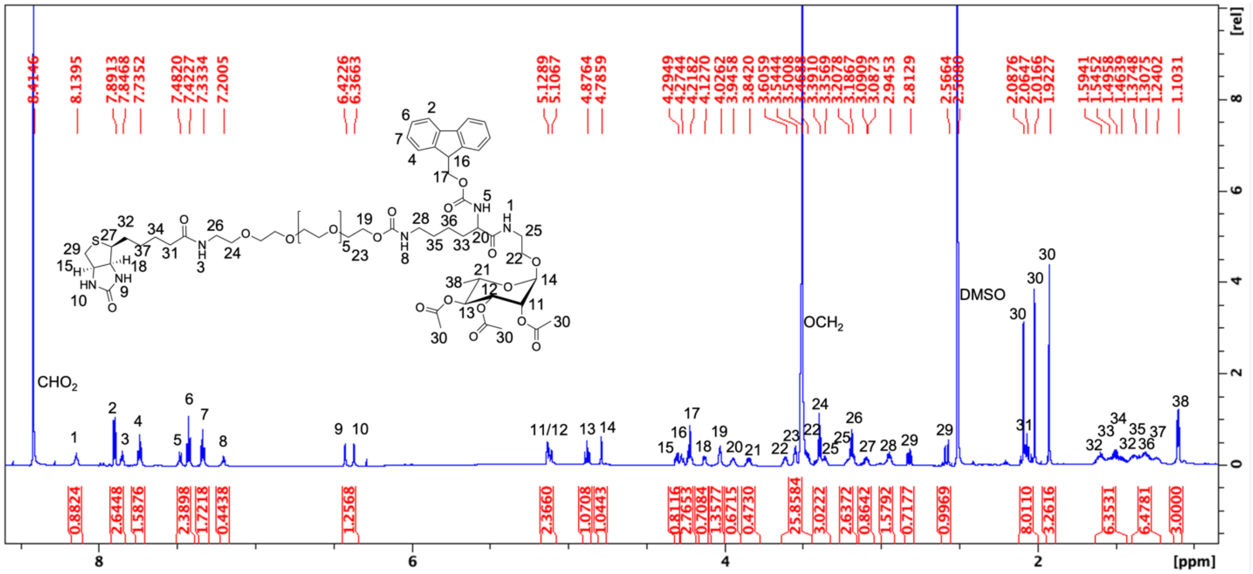

Figure S123. ^{1}H-NMR of **Peracetyl-α-O-Rhamnose-Lysine-α-Fmoc-ε-Peg8-Biotin (36)**, DMSO.

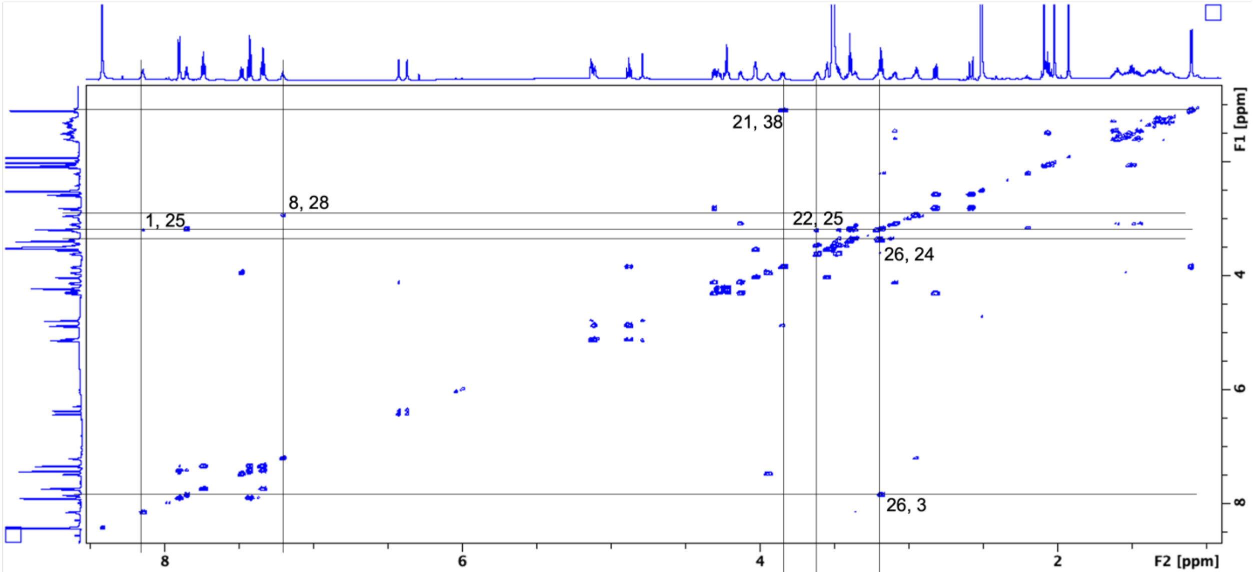

Figure S124. ^{1}H^{1}H-COSY NMR of **Peracetyl-α-O-Rhamnose-Lysine-α-Fmoc-ε-Peg8-Biotin (36)**, DMSO.

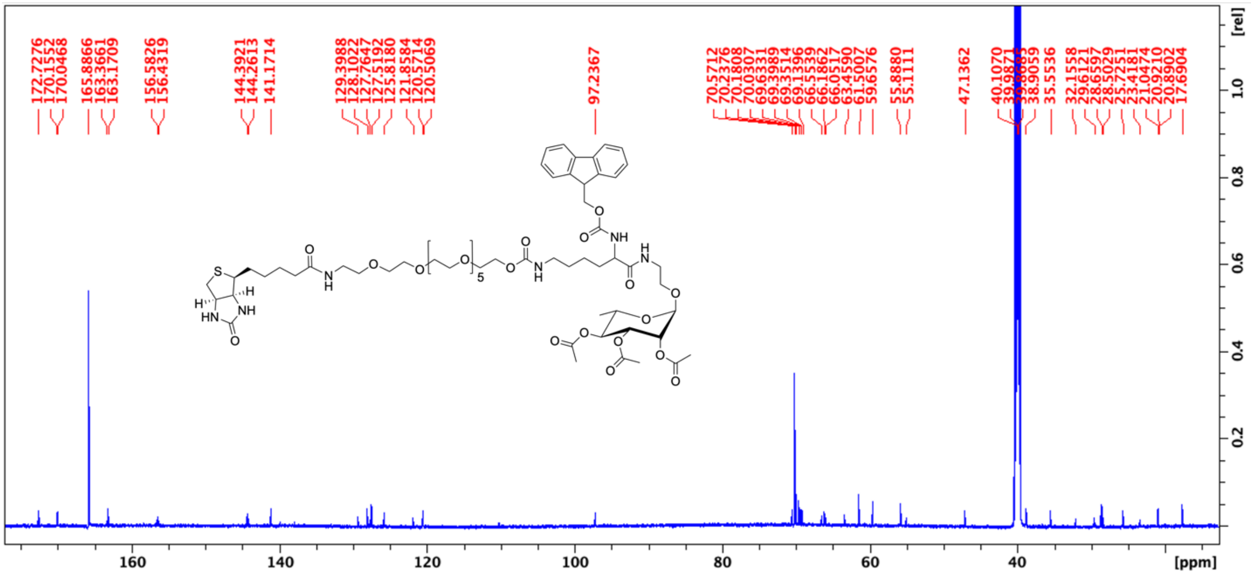

Figure S125. ^{13}C-NMR of **Peracetyl-α-O-Rhamnose-Lysine-α-Fmoc-ε-Peg8-Biotin (36),** DMSO.

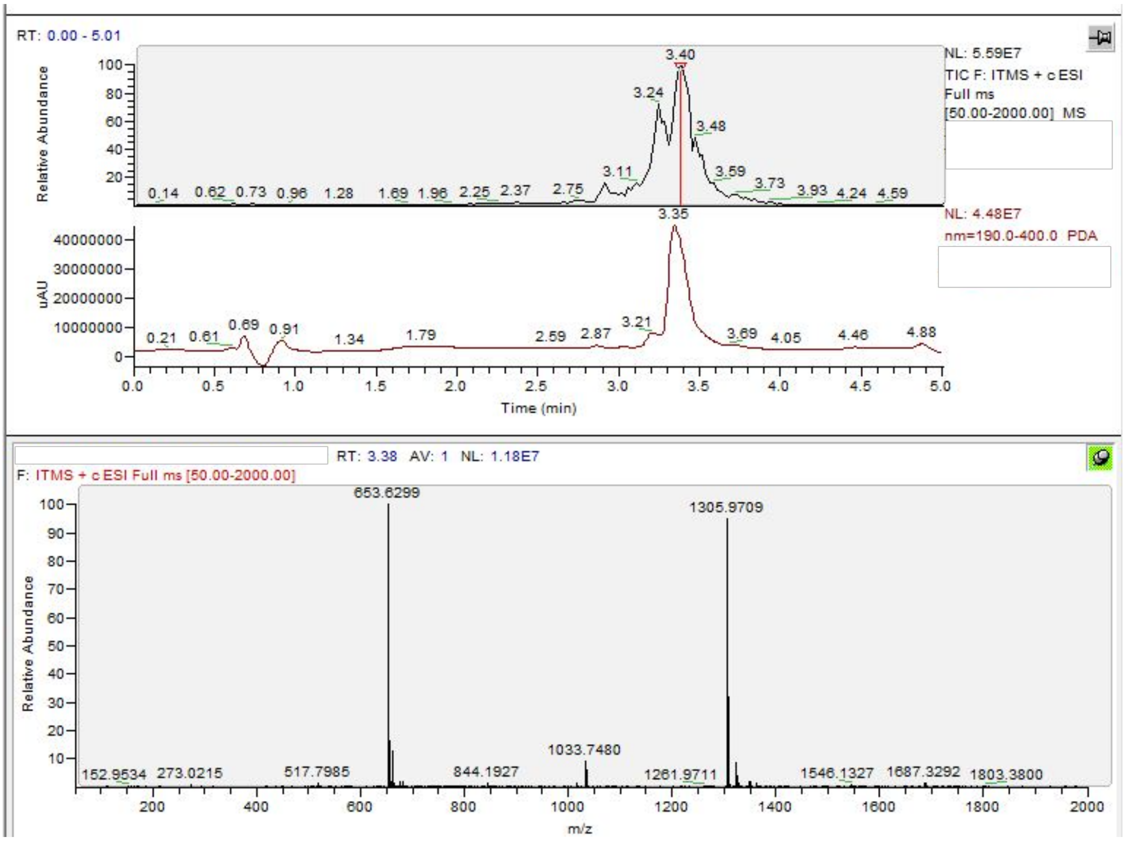

Figure S126. LC-MS trace of post column purified compound **Peracetyl-α-O-Rhamnose-Lysine-α-Fmoc-ε-Peg8-Biotin (36)**. Top - TIC trace. Bottom - selected mass spectrum from TIC highlighted by red bar (retention time 3.38). Middle - UV chromatogram.

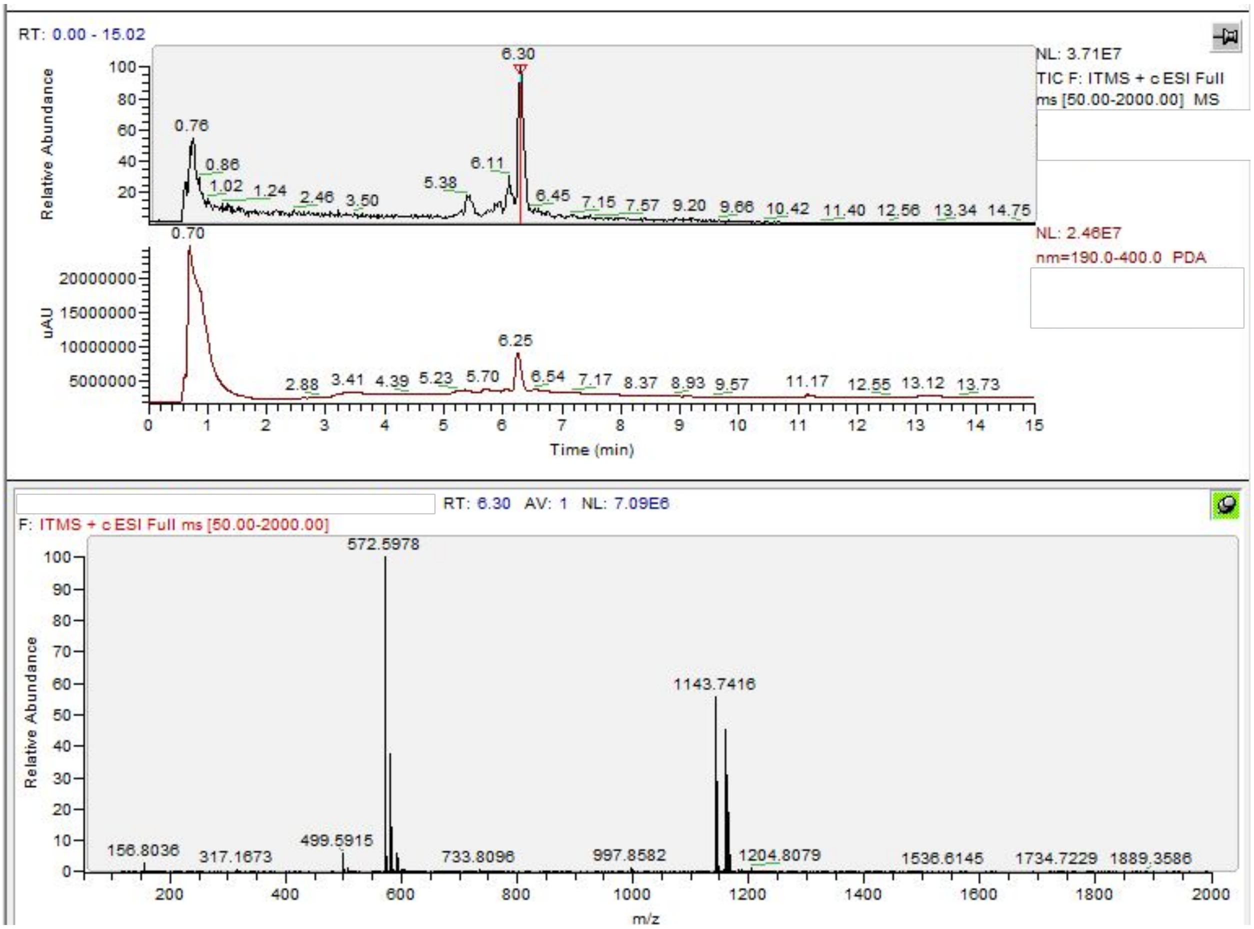

Figure S127. LC-MS trace of post column purified compound **Biotin-SO_2F-cARM (12)**. Top - TIC trace. Bottom - selected mass spectrum from TIC highlighted by red bar (retention time 6.30). Middle - UV chromatogram.

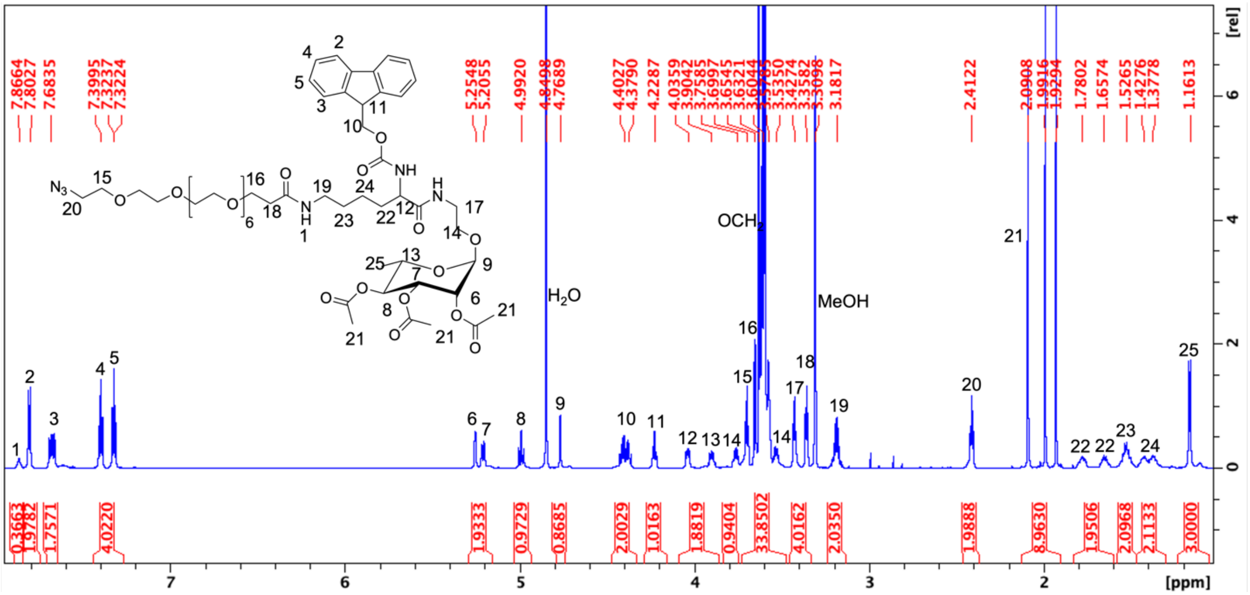

Figure S128. ^{1}H-NMR of **Peracetyl-α-O-Rhamnose-Lysine-α-Fmoc-ε-Peg8-N_3 (37)**, MeOD.

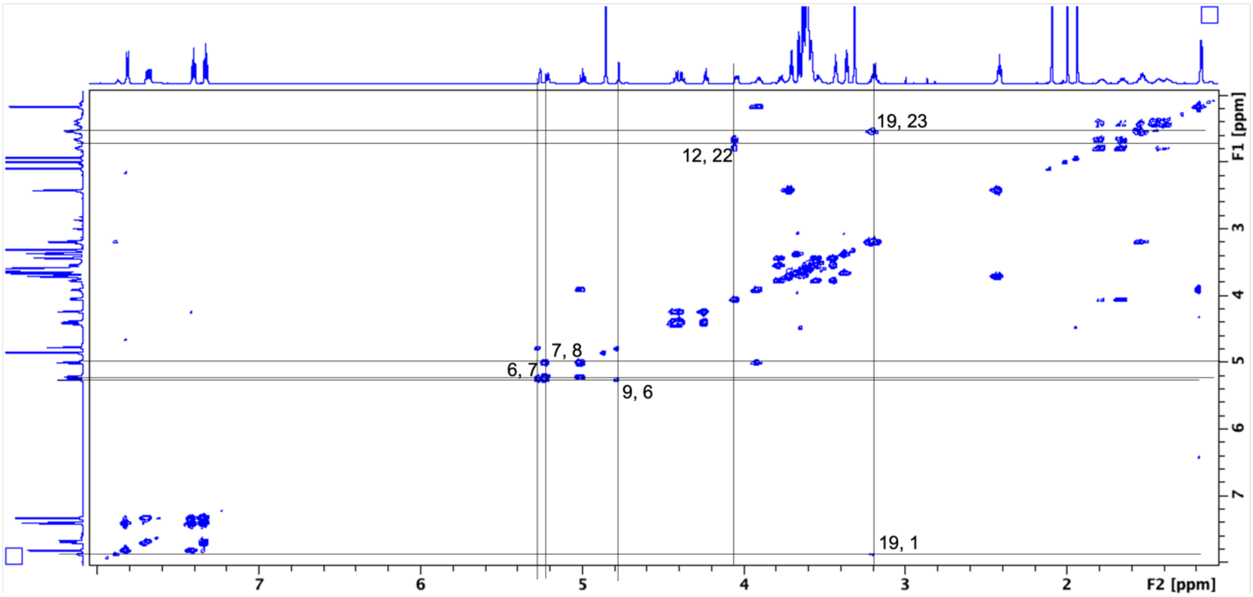

Figure S129. ^{1}H^{1}H-COSY NMR of **Peracetyl-α-O-Rhamnose-Lysine-α-Fmoc-ε-Peg8-N_3 (37)**, MeOD.

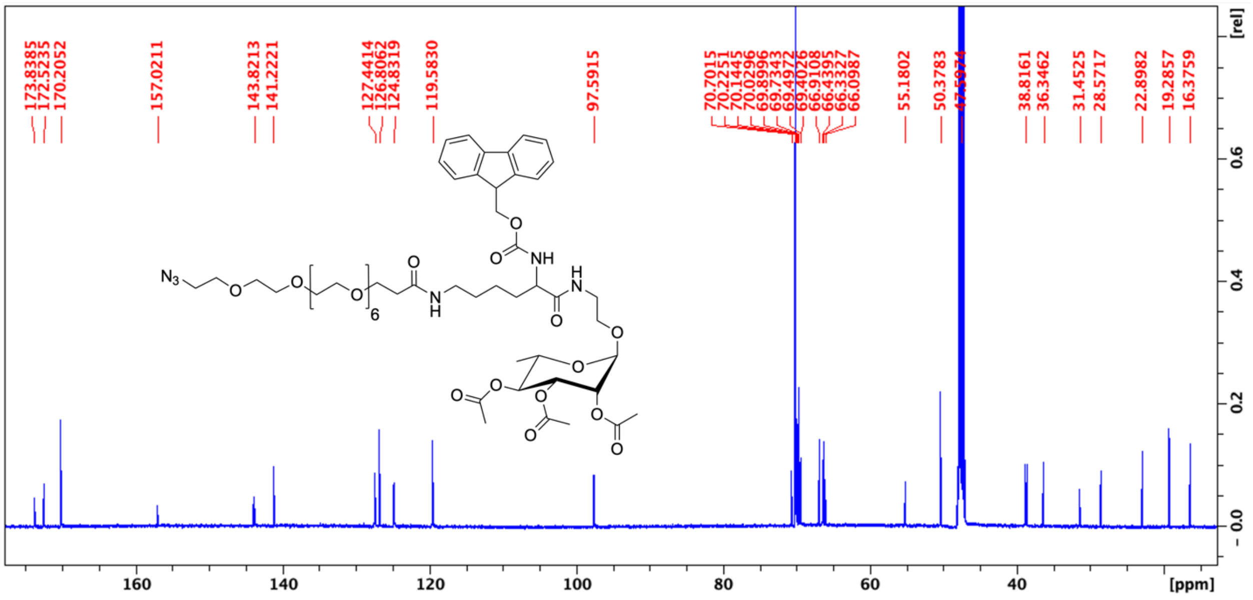

Figure S130. ^{13}C-NMR of **<u>Peracetyl-α-O-Rhamnose-Lysine-α-Fmoc-ε-Peg8-N$_3$ (37)</u>**, MeOD.

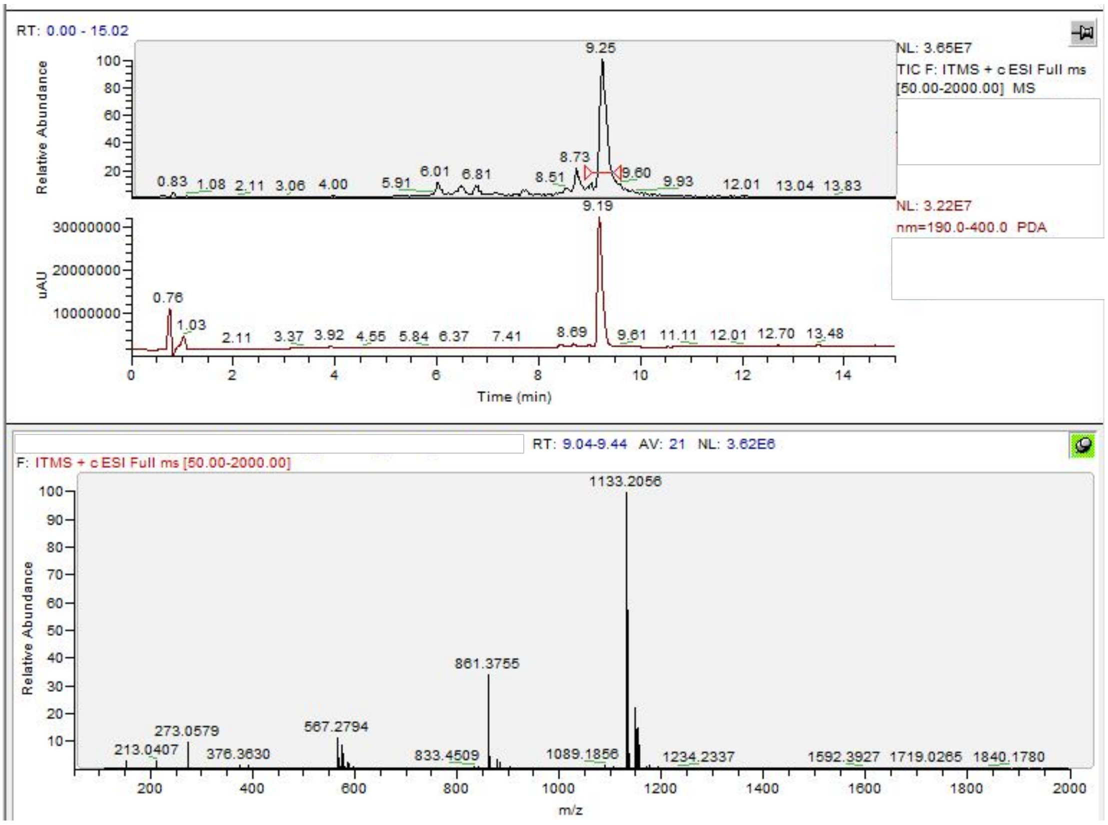

Figure S131. LC-MS trace of post column purified compound **<u>Peracetyl-α-O-Rhamnose-Lysine-α-Fmoc-ε-Peg8-N$_3$ (37)</u>**. Top - TIC trace. Bottom - selected mass spectrum from TIC highlighted by red bar (retention time 9.04 – 9.44). Middle - UV chromatogram.

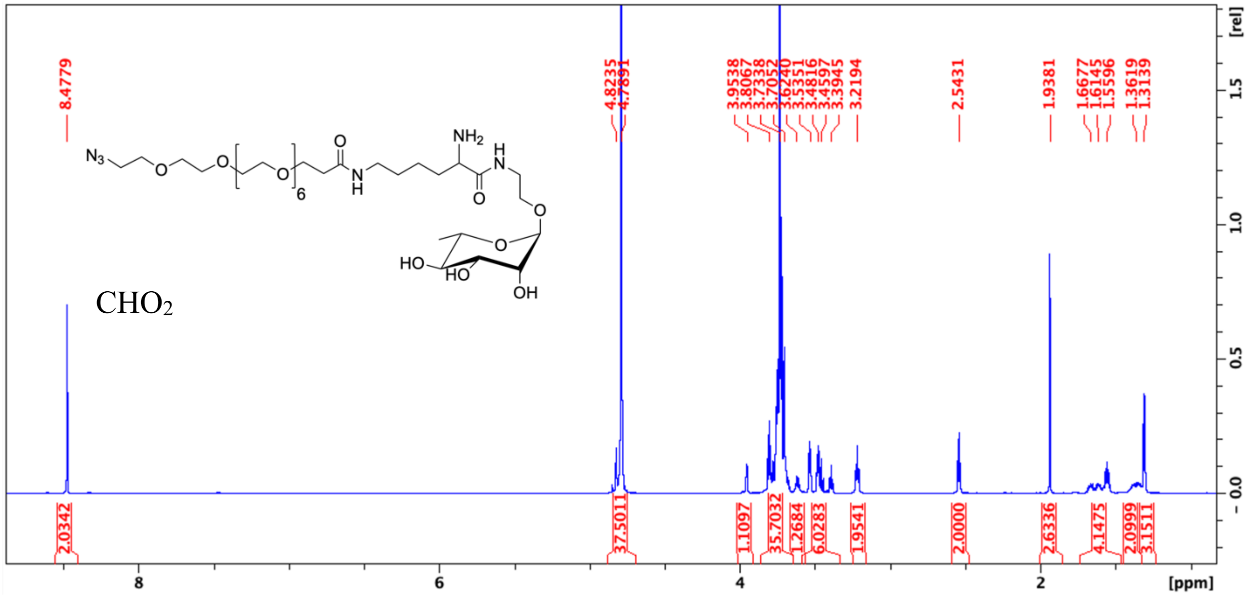

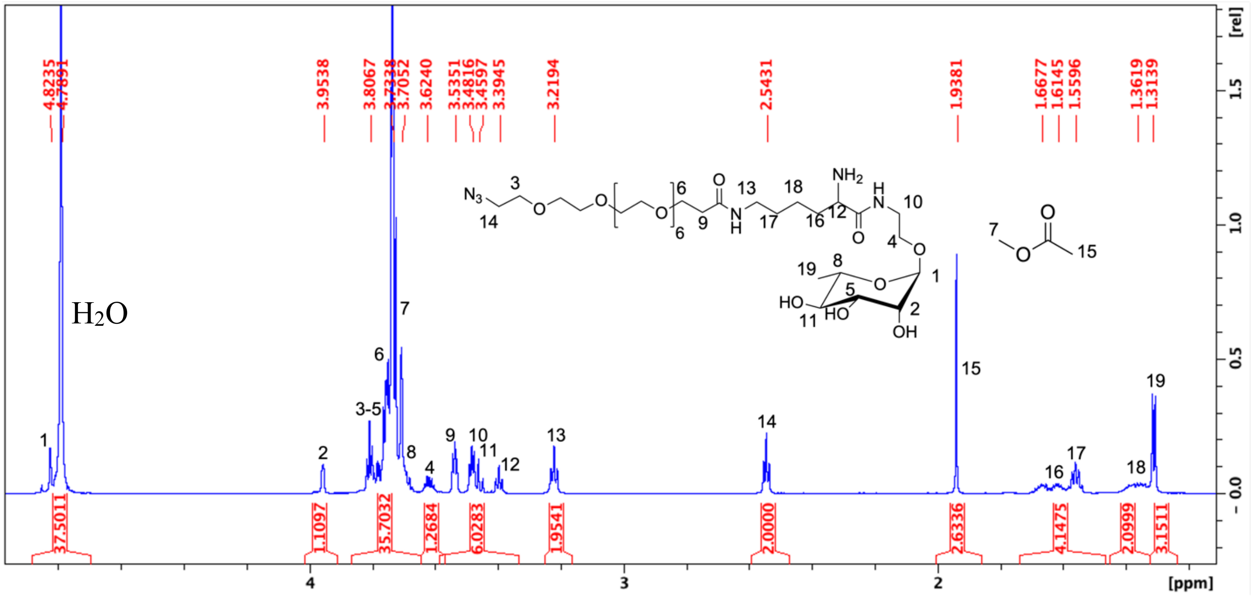

Figure S132. ^{1}H-NMR of **Peracetyl-α-O-Rhamnose-Lysine-α-NH$_2$-ε-Peg8-N$_3$ (38)**, D$_2$O. Top is full spectrum, bottom is narrow range.

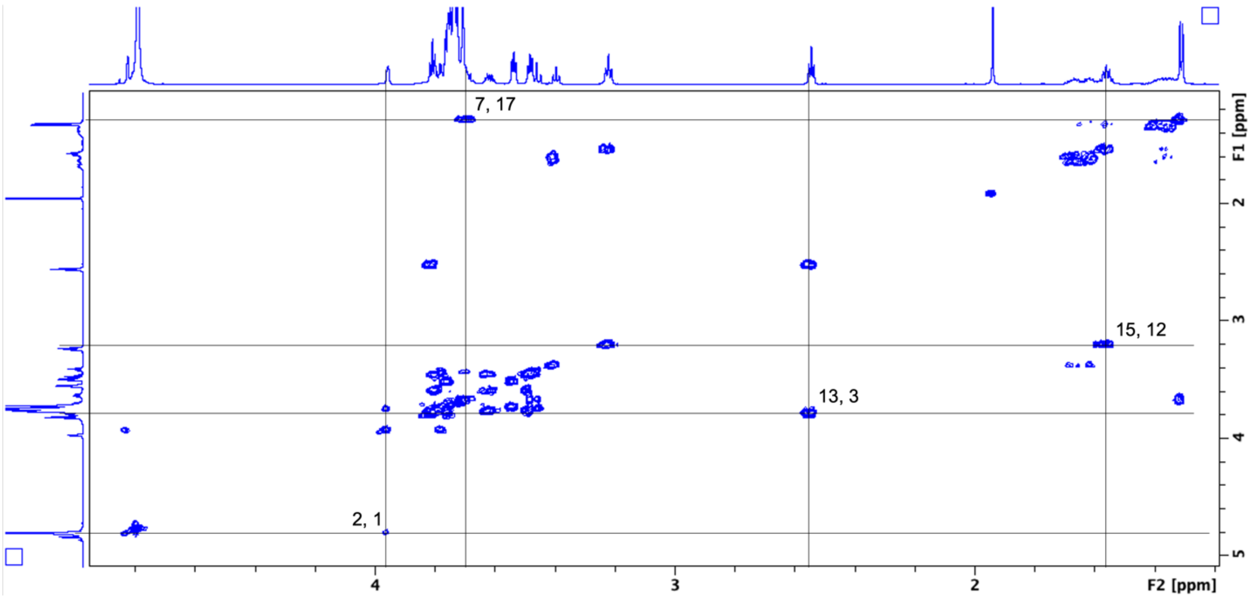

Figure S133. ^{1}H^{1}H-COSY NMR of **<u>Peracetyl-α-O-Rhamnose-Lysine-α-NH$_2$-ε-Peg8-N$_3$ (38)</u>**, D_2O.

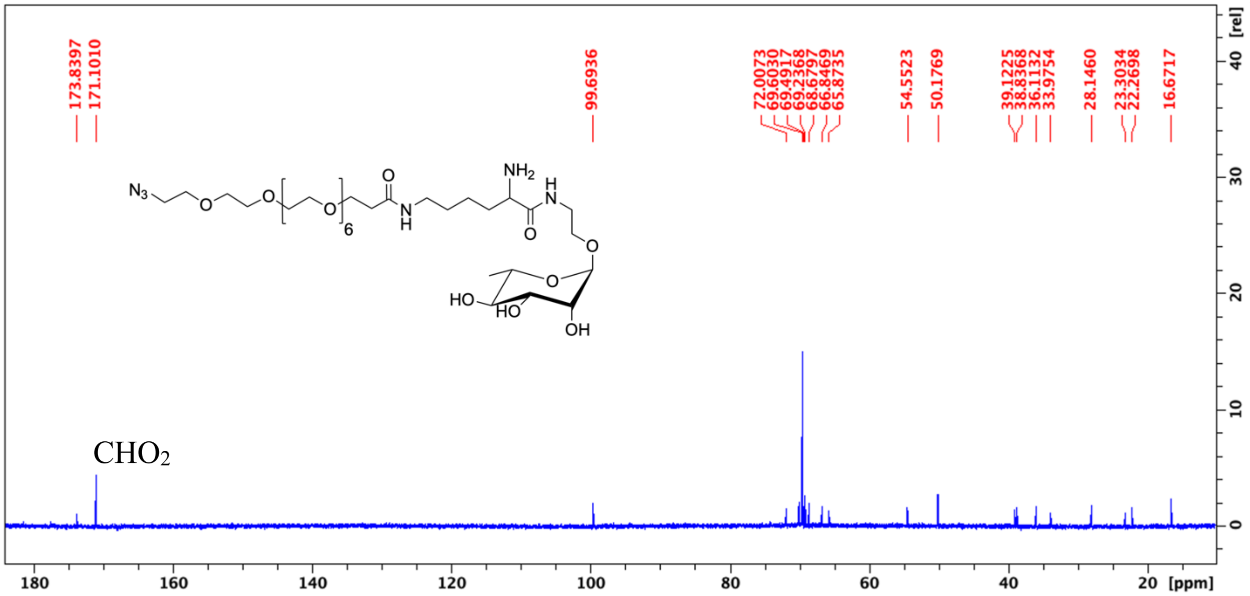

Figure S134. ^{13}C-NMR of **<u>Peracetyl-α-O-Rhamnose-Lysine-α-NH$_2$-ε-Peg8-N$_3$ (38)</u>**, D_2O.

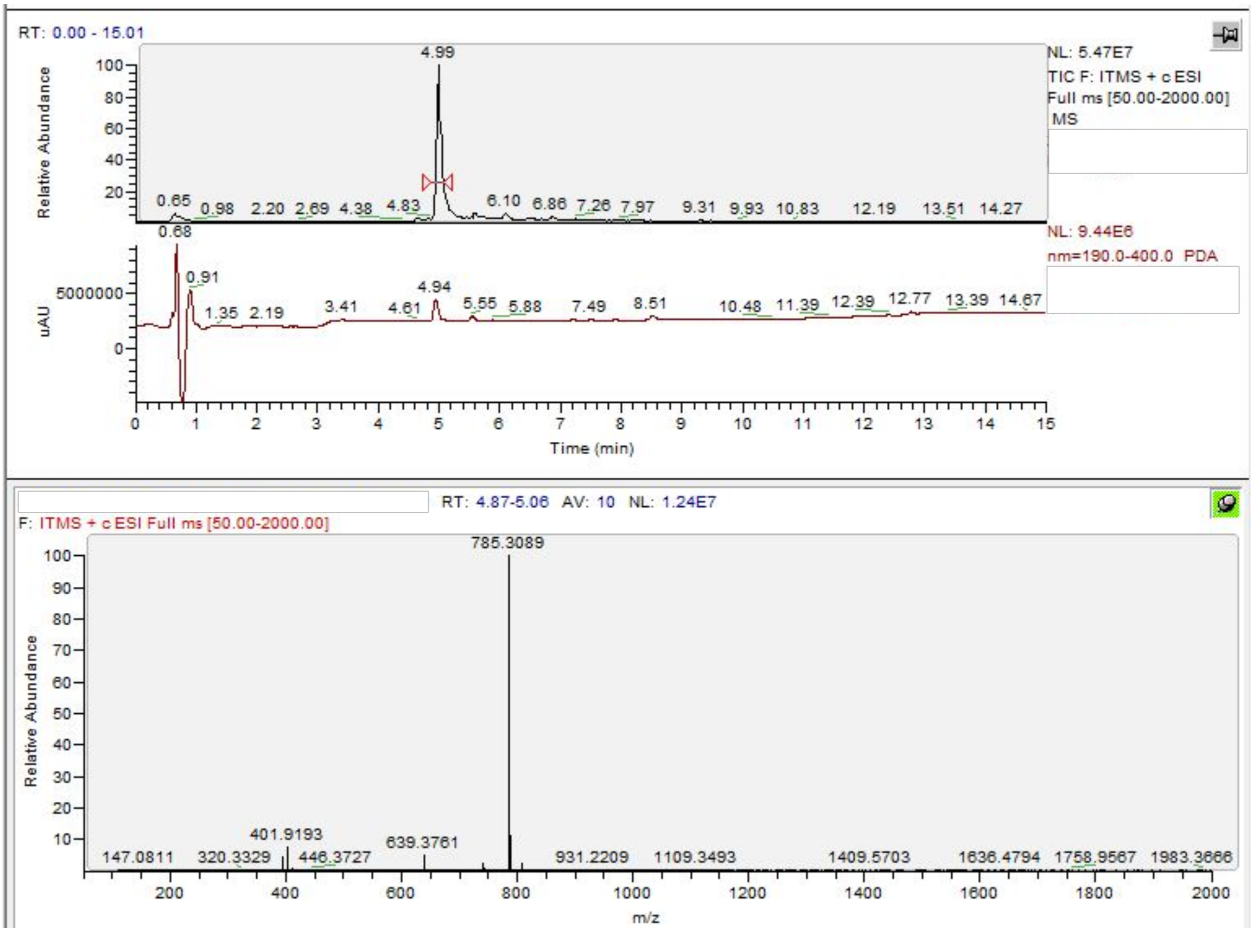

Figure S135. LC-MS trace of post column purified compound **Peracetyl-α-O-Rhamnose-Lysine-α-NH_2-ε-Peg8-N_3 (38)**. Top - TIC trace. Bottom - selected mass spectrum from TIC highlighted by red bar (retention time 4.87 – 5.06). Middle - UV chromatogram.

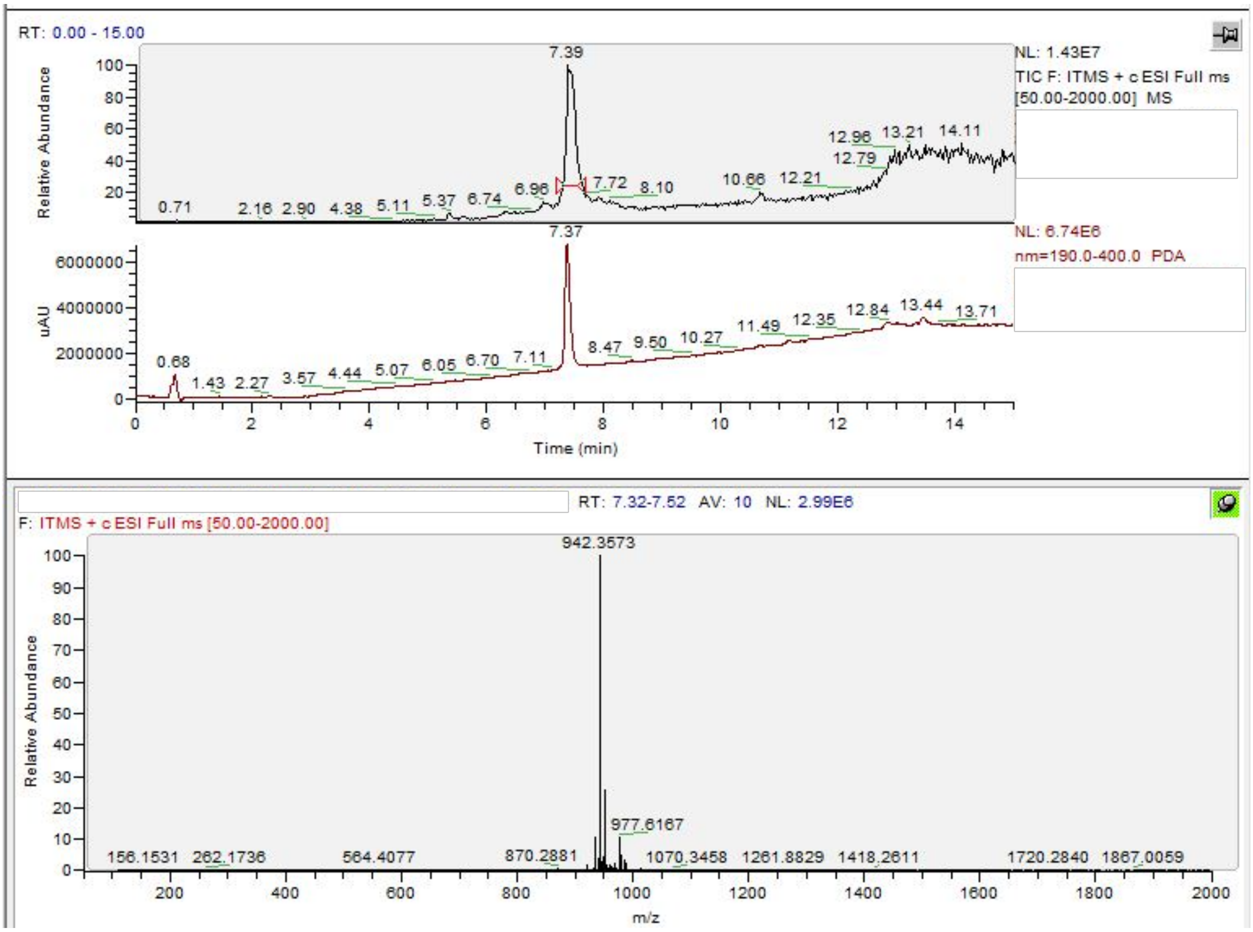

Figure S136. LC-MS trace of post column purified compound A647-SO_2F-cARM (13). Top - TIC trace. Bottom - selected mass spectrum from TIC highlighted by red bar (retention time 5.02 – 5.20). Middle - UV chromatogram.

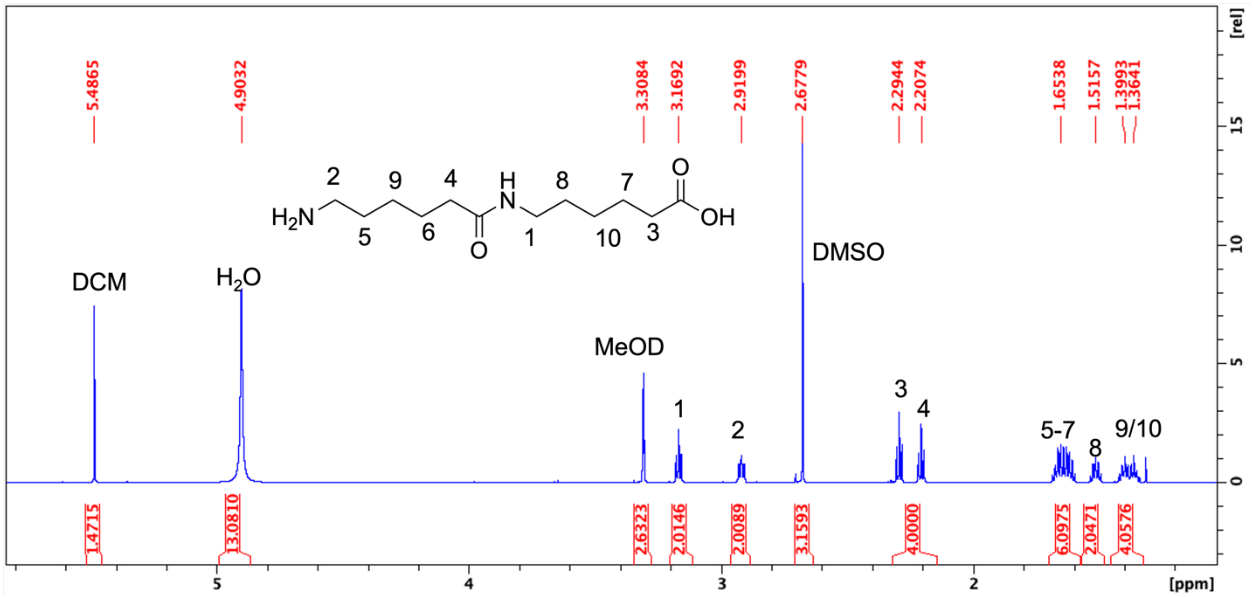

Figure S137. ^{1}H-NMR of **NH$_2$-(Hexanoic)$_2$-Carboxylic Acid (39)**, MeOD.

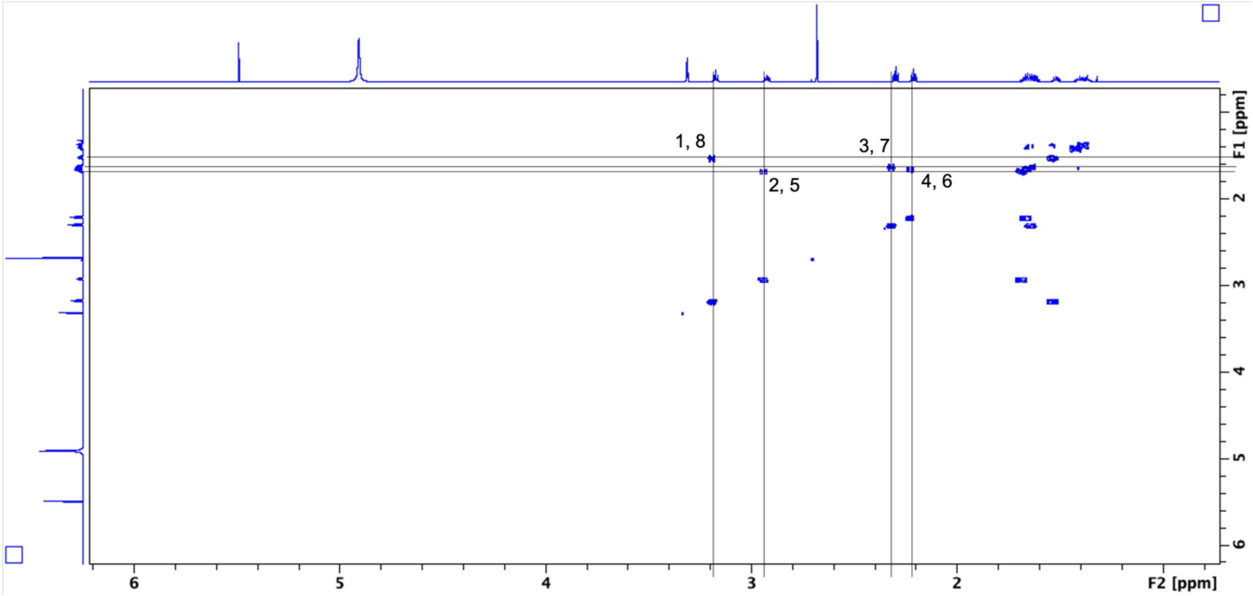

Figure S138. ^{1}H^{1}H-COSY NMR of **NH$_2$-(Hexanoic)$_2$-Carboxylic Acid (39)**, MeOD.

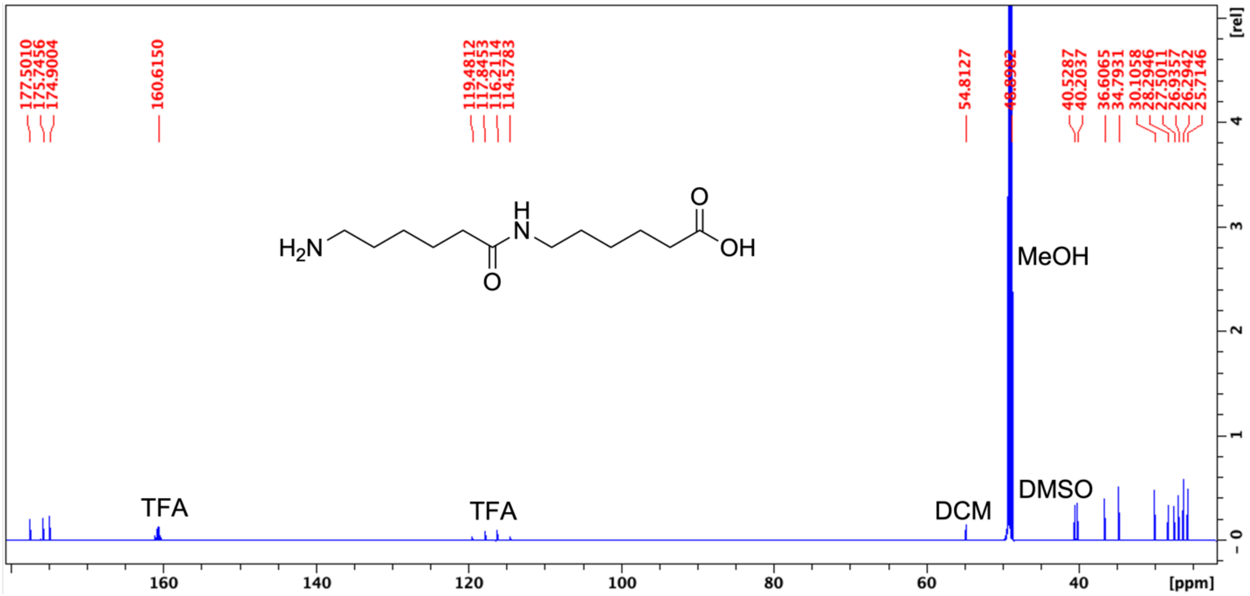

Figure S139. ^{13}C-NMR of **<u>NH$_2$-(Hexanoic)$_2$-Carboxylic Acid (39)</u>**, MeOD.

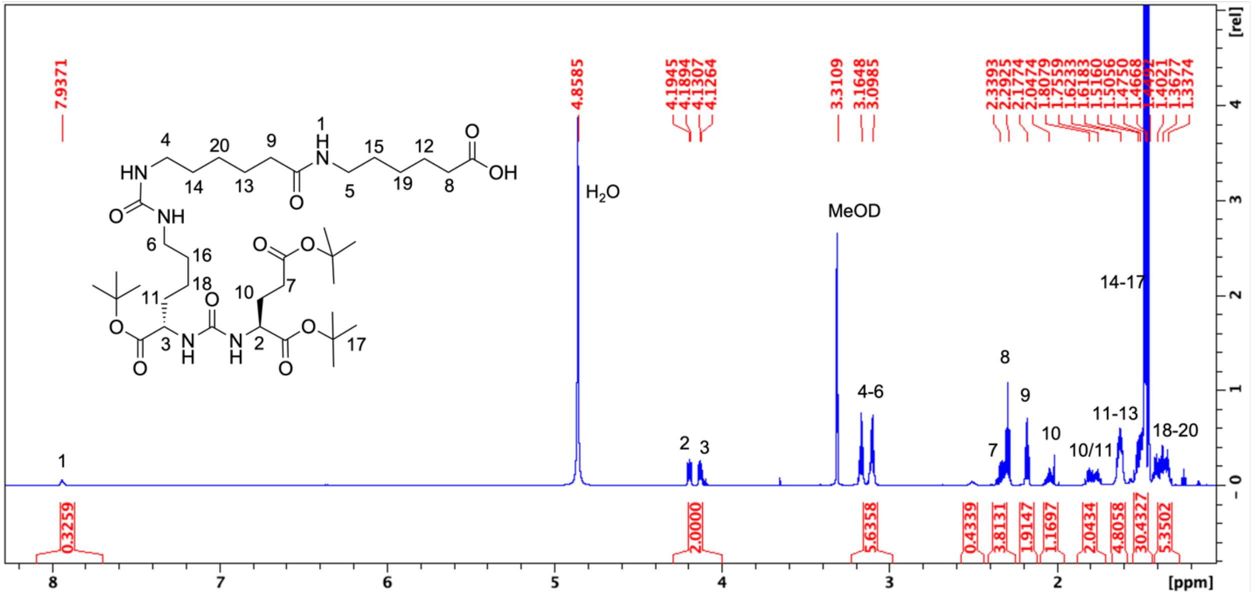

Figure S140. ^{1}H-NMR of **<u>Tri-t-butyl-GUL-Alkyl-COOH (40)</u>**, MeOD.

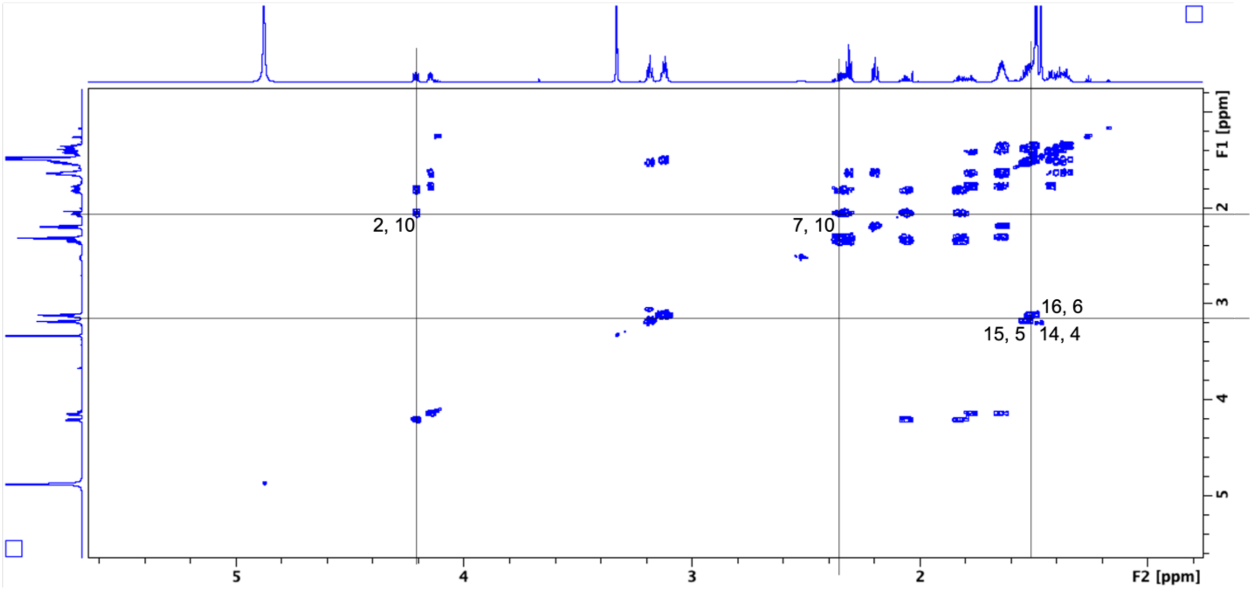

Figure S141. $^1H^1H$-COSY NMR of **<u>Tri-t-butyl-GUL-Alkyl-COOH (40)</u>**, MeOD.

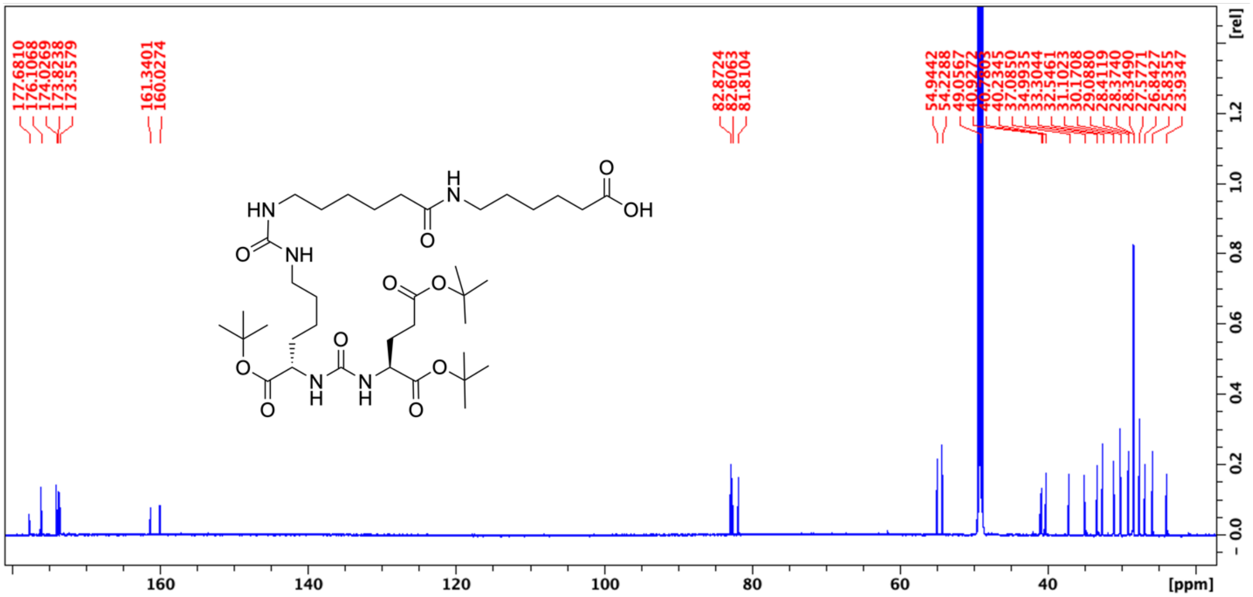

Figure S142. ^{13}C-NMR of **<u>Tri-t-butyl-GUL-Alkyl-COOH (40)</u>**, MeOD.

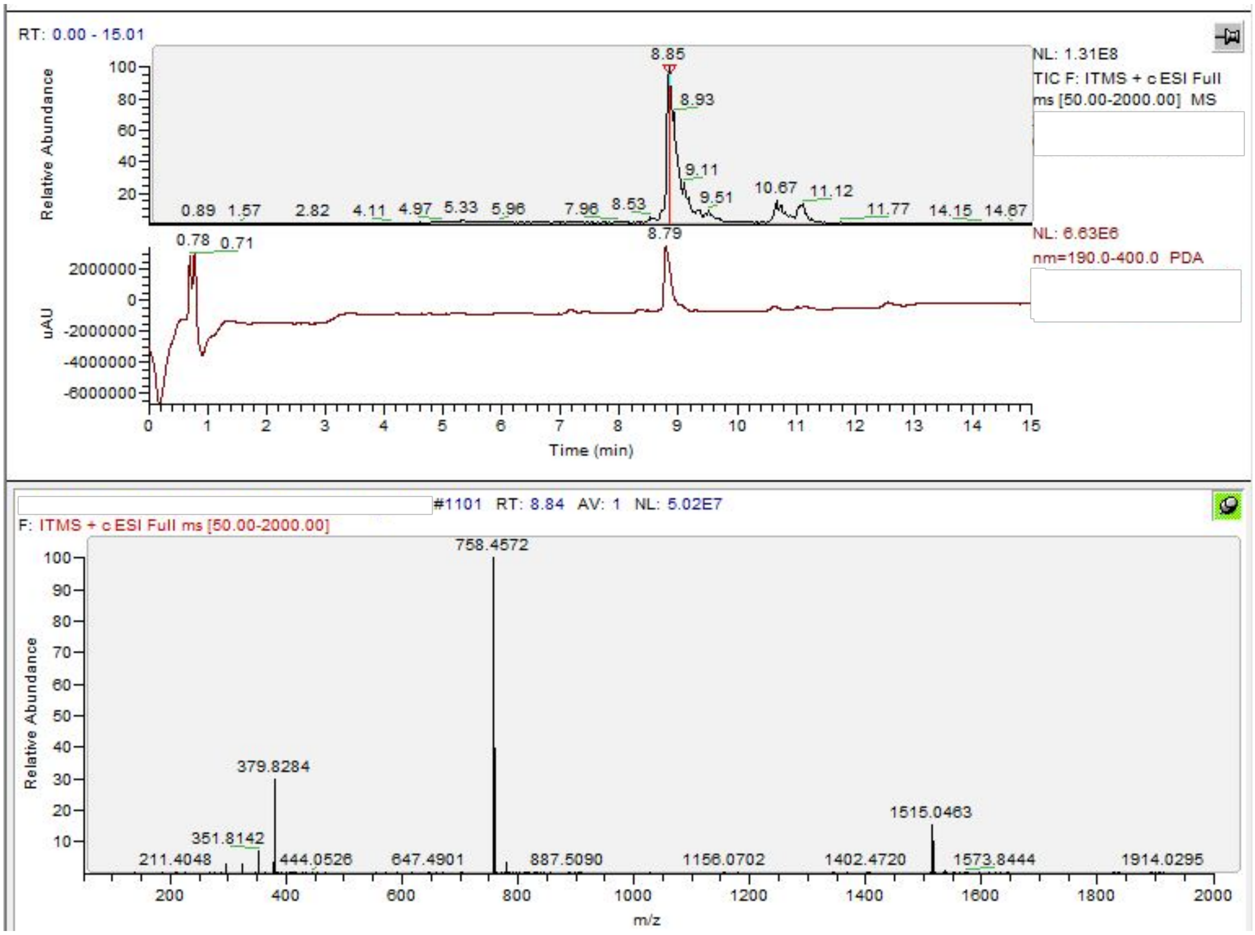

Figure S143. LC-MS trace of post column purified compound **Tri-t-butyl-GUL-Alkyl-COOH (40)**. Top - TIC trace. Bottom - selected mass spectrum from TIC highlighted by red bar (retention time 8.84). Middle - UV chromatogram.

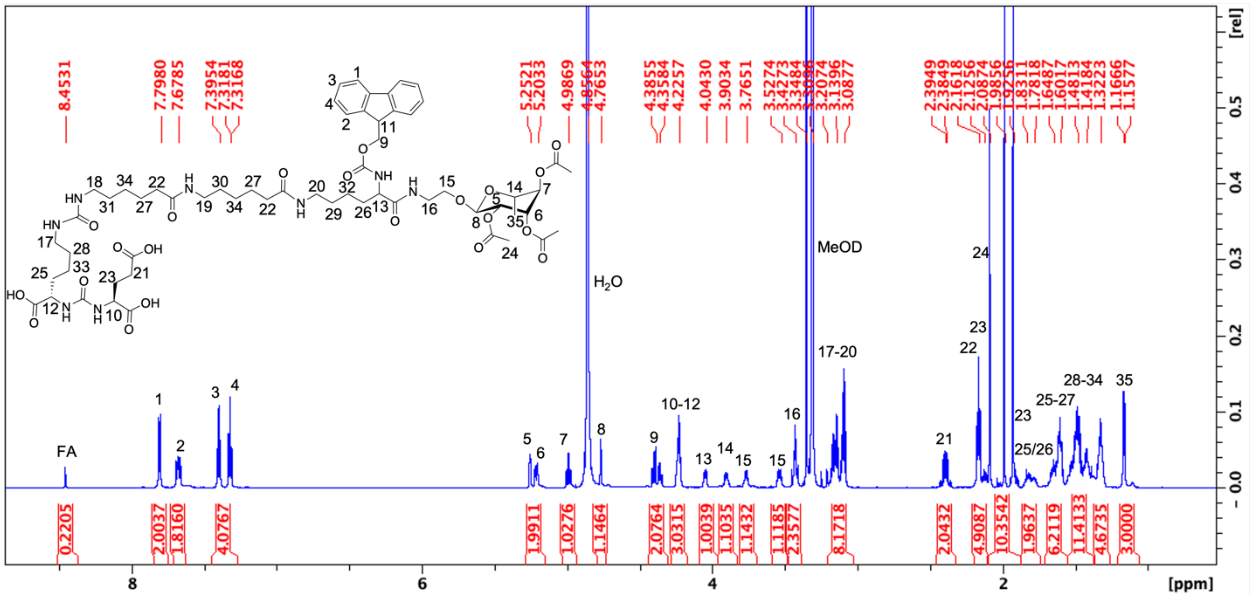

Figure S144. ^{1}H-NMR of **GUL-Alkyl-NHFmoc-α-O-Rhamnose (41)**, MeOD.

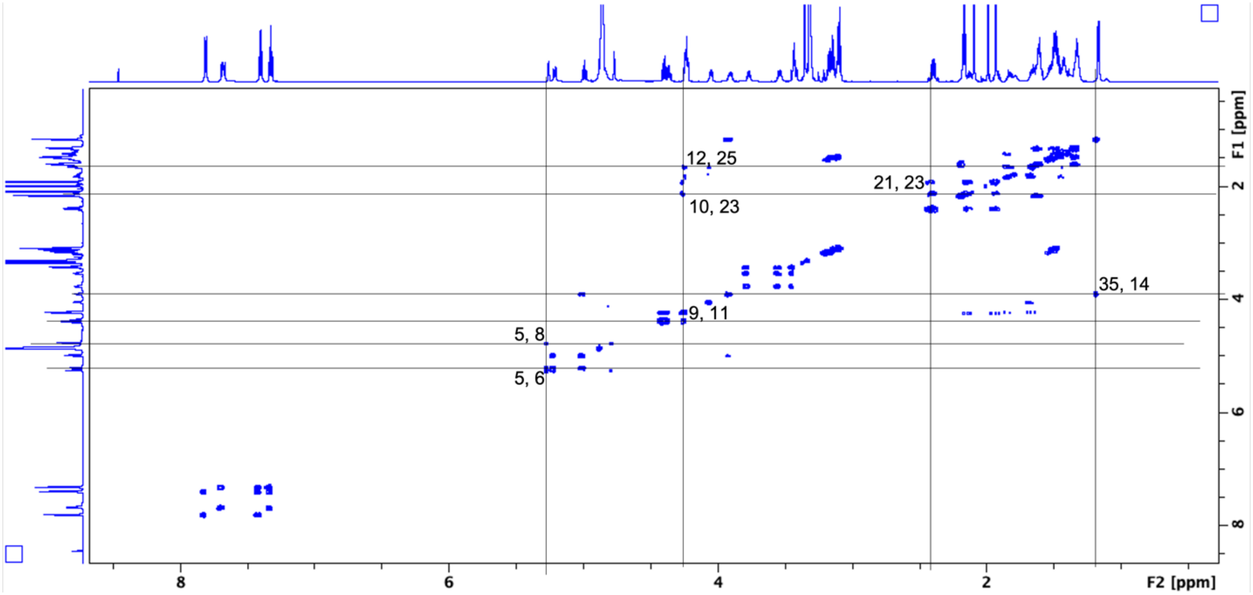

Figure S145. ^{1}H^{1}H-COSY NMR of **GUL-Alkyl-NHFmoc-α-O-Rhamnose (41)**, MeOD.

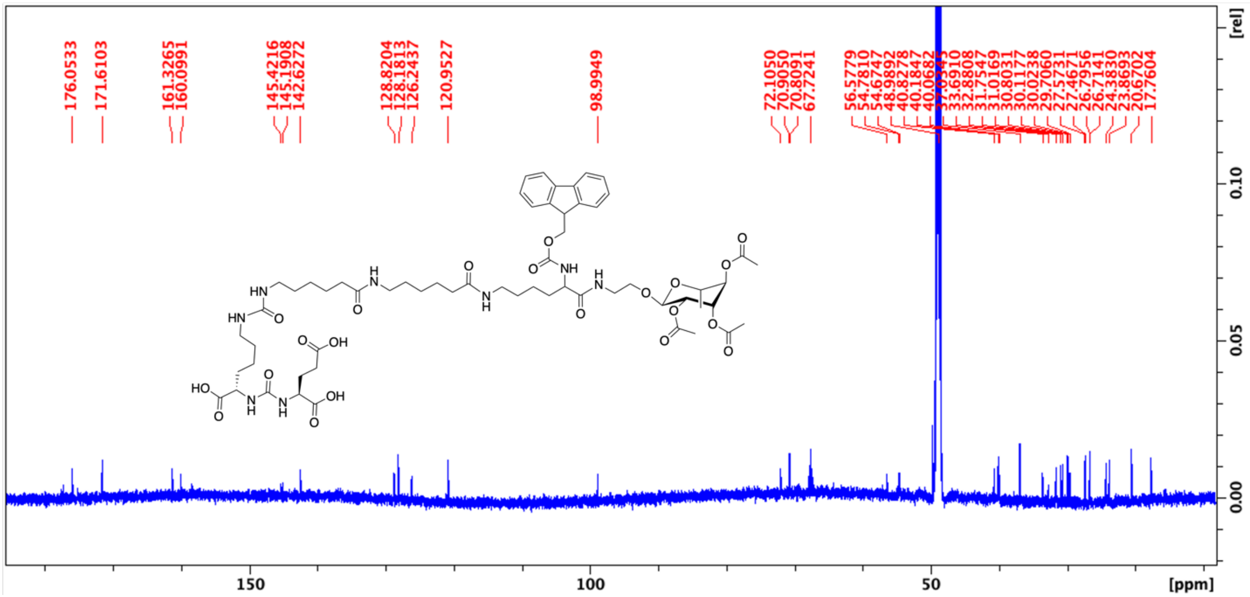

Figure S146. ^{13}C-NMR of **GUL-Alkyl-NHFmoc-α-O-Rhamnose (41)**, MeOD.

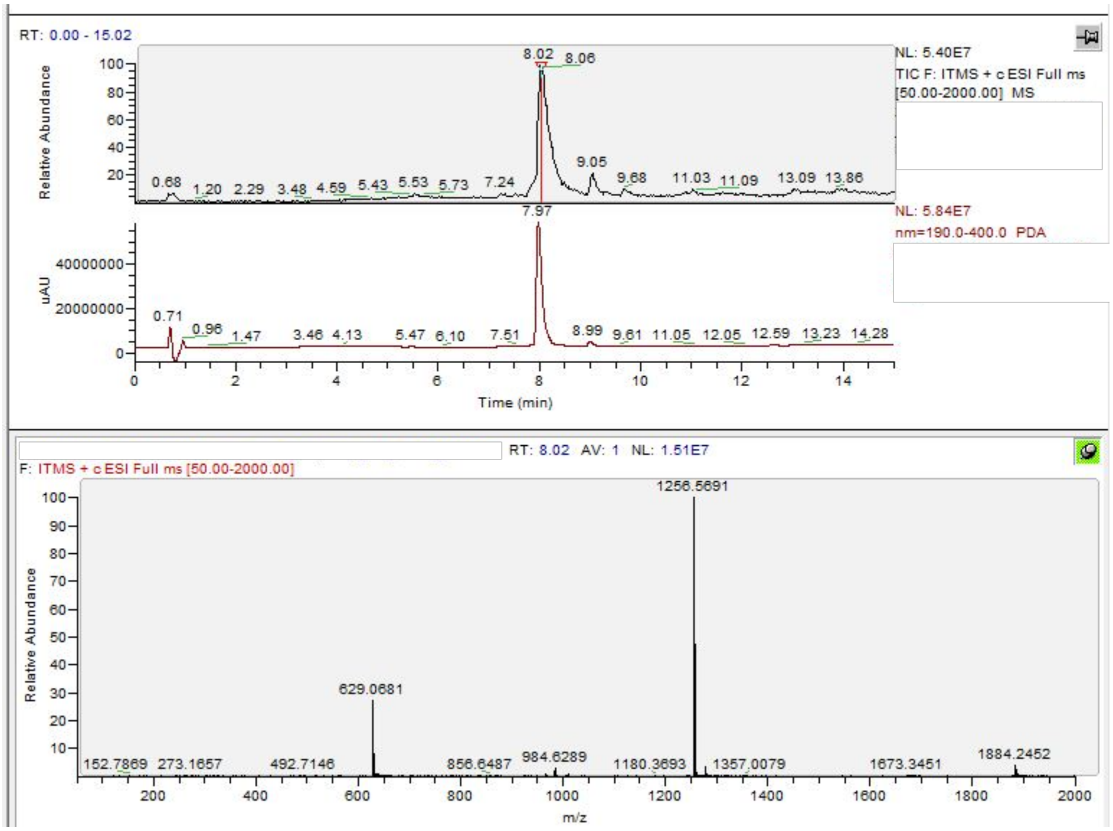

Figure S147. LC-MS trace of post column purified compound **GUL-Alkyl-NHFmoc-α-O-Rhamnose (41)**. Top - TIC trace. Bottom - selected mass spectrum from TIC highlighted by red bar (retention time 8.02). Middle - UV chromatogram.

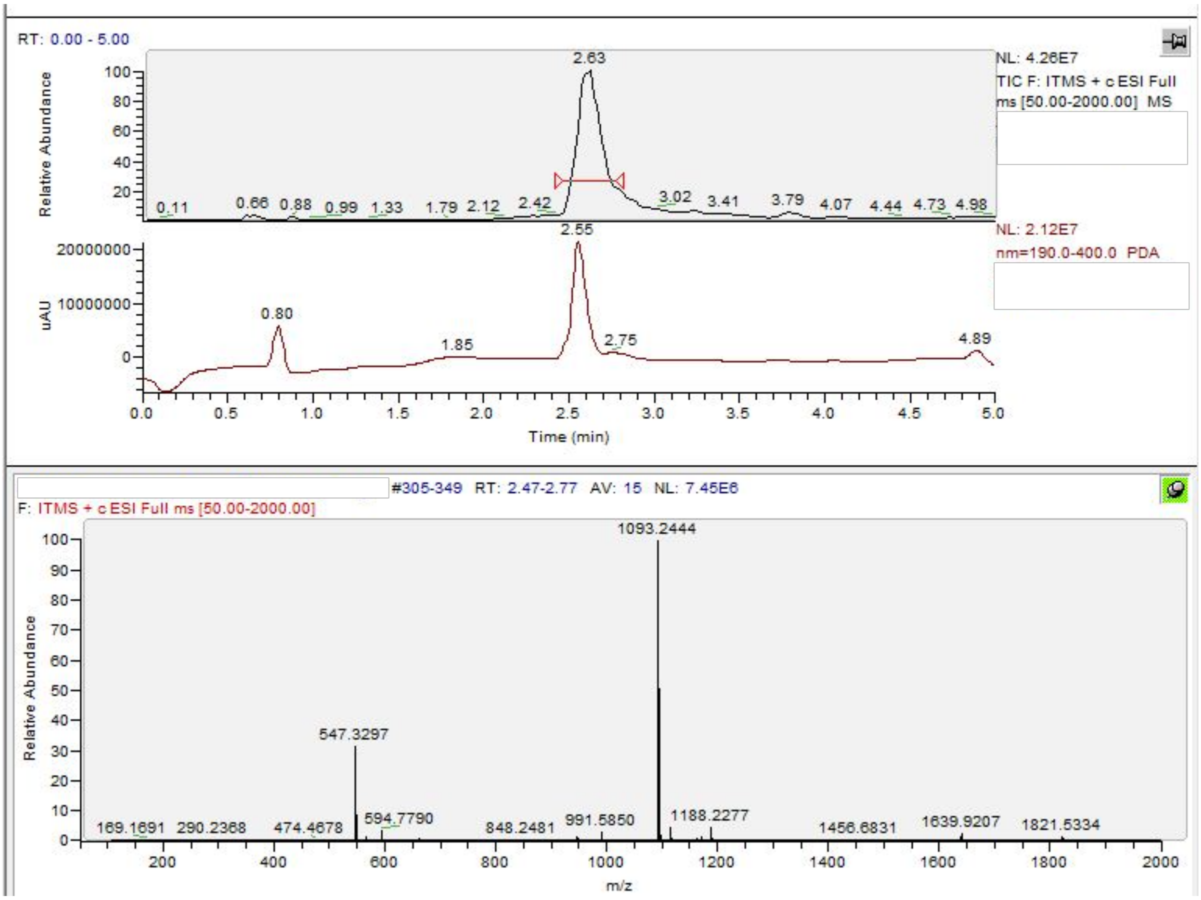

Figure S148. LC-MS trace of post column purified compound **GUL-SO_2F-cARM (14)**. Top - TIC trace. Bottom - selected mass spectrum from TIC highlighted by red bar (retention time 2.47 – 2.77). Middle - UV chromatogram.

References

(1) Kapcan, E.; Lake, B.; Yang, Z.; Zhang, A.; Miller, M. S.; Rullo, A. F. Covalent Stabilization of Antibody Recruitment Enhances Immune Recognition of Cancer Targets. *Biochemistry* **2021**, *60*, 1447–1458. https://doi.org/10.1021/acs.biochem.1c00127.

(2) Lake, B.; Serniuck, N.; Kapcan, E.; Wang, A.; Rullo, A. F. Covalent Immune Recruiters: Tools to Gain Chemical Control Over Immune Recognition. *ACS Chem. Biol.* **2020**, *15* (4), 1089–1095. https://doi.org/10.1021/acschembio.0c00112.

(3) Oyelaran, O.; Mcshane, L. M.; Dodd, L.; Gildersleeve, J. C. Profiling Human Serum Antibodies with a Carbohydrate Antigen Microarray. *J. Proteome Res.* **2009**, *8*, 4301–4310. https://doi.org/10.1021/pr900515y.

(4) Sheridan, R. T. C.; Hudon, J.; Hank, J. A.; Sondel, P. M.; Kiessling, L. L. Rhamnose Glycoconjugates for the Recruitment of Endogenous Anti-Carbohydrate Antibodies to Tumor Cells. *ChemBioChem* **2014**, *15* (10), 1393–1398. https://doi.org/10.1002/cbic.201402019.

(5) Sterlow, J. M. A Perspective on the Kinetics of Covalent and Irreversible Inhibition. *SLAS Discov.* **2017**, *22* (1), 3–20.
